# Heterobenzyl Chlorides as Linchpins for C–H Arylation via Sequential C–H Chlorination/Cross-Electrophile Coupling

**DOI:** 10.1021/acscatal.5c08221

**Published:** 2026-02-03

**Authors:** Jack T. Floreancig, Marco A. Lopez, Allison R. Dick, Luana Cardinale, Nicole C. Goodwin, Darren L. Poole, Shannon S. Stahl

**Affiliations:** † Department of Chemistry, 5228 University of Wisconsin−Madison, 1101 University Avenue, Madison, Wisconsin 53706, United States; ⊥ Chemistry Department, Wheaton College, 501 College Avenue, Wheaton, Illinois 60187, United States; § GSK, 1250 South Collegeville Road, Collegeville, Pennsylvania 19426, United States; ∥ Molecular Modalities Capabilities, 113460 GSK Medicines Research Centre, Stevenage SG1 2NY, U.K.

**Keywords:** homogeneous catalysis, cross-coupling, nickel, pyridine, reductive

## Abstract

Synthetic methods that use C­(sp^3^)–H bonds in carbon–carbon cross-coupling reactions are limited and often lack generality, particularly with substrates containing basic heterocycles. Here, we demonstrate the arylation of heterobenzylic C–H bonds by using heterobenzyl chlorides as linchpins that can undergo Ni-catalyzed cross-electrophile coupling with aryl iodides. The results show different reactivity for primary and secondary heterobenzyl chlorides and also show differences among secondary heterobenzyl chlorides at different positions on the heteroaromatic ring. The Ni-catalyzed conditions identified for each of these substrate classes ensure that the rate of heterobenzyl chloride activation complements the rate of aryl iodide activation. These methods are demonstrated with series of heterobenzyl chlorides and (hetero)­aryl iodides, providing a general strategy for C­(sp^3^)–H arylation.

## Graphical abstract

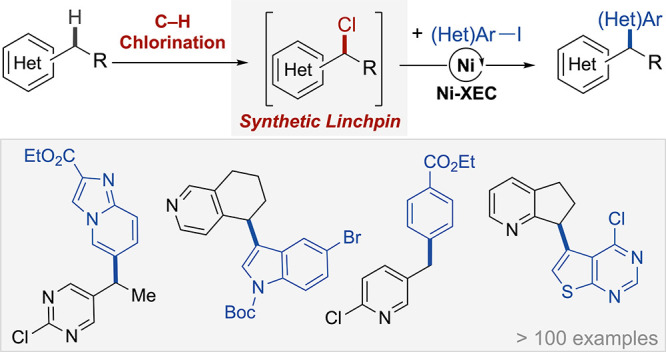

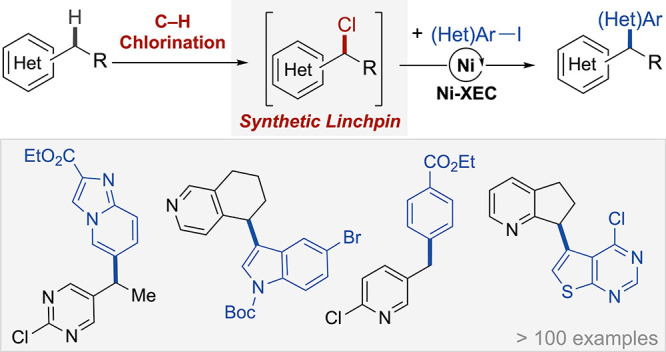

## Introduction

Cross-coupling reactions play a crucial role in the diversification of molecular building blocks in pharmaceutical and agrochemical discovery.^1^ While aryl and alkyl halides are the most common cross-coupling partners, recent efforts have shown how latent functional groups, such as alcohols, amines, and carboxylic acids,^2–5^ may be activated to access new sites for selective cross-coupling. Benzylic C–H bonds are even more prevalent in commercial building blocks than conventional functional groups, and they represent a strategic target for cross-coupling.^6–9^ Methods for direct coupling of benzylic C–H bonds with different partners, such as aryl halides,^10^ alcohols,^11,12^ boronic acids,^13^ and alkynes,^14^ have been developed; however, the reagents and conditions used in these reactions often restrict the scope of successful reactivity. For example, many of these methods rely on oxidants that have poor compatibility with basic or electron-rich functional groups. Pyridines and related aromatic heterocycles represent a persistent challenge that is especially problematic because of their prevalence in pharmaceuticals and other bioactive compounds. To address this challenge, we have begun pursuing sequential C–H functionalization/diversification strategies that could greatly expand the scope of compatible reactions and coupling partners. For example, halogenation of (hetero)­benzylic C–H bonds enables coupling with diverse heteroatom nucleophiles,^15–17^ including alcohols, amines, thiols, and carboxylic acids, many of which contain functional groups that would not be compatible with direct C–H cross coupling. C–C bond formation, which could find widespread utility in drug discovery and related applications (Figure 1A), is a notable void in this work. Here, we address this limitation by developing nickel-catalyzed cross-electrophile coupling (Ni-XEC) reactions with heterobenzyl chlorides (Figure 1B). This class of electrophiles has not previously been used in Ni-XEC. The results underscore reactivity challenges associated with heterocyclic core structures that are absent from previously reported Ni-XEC reactions of benzyl chlorides, while presenting a set of catalytic conditions equipped to overcome these challenges. The reactions are then showcased in sequential heterobenzylic C–H chlorination/Ni-XEC to highlight opportunities for efficient diversification of aromatic heterocycles.

**Figure 1:**
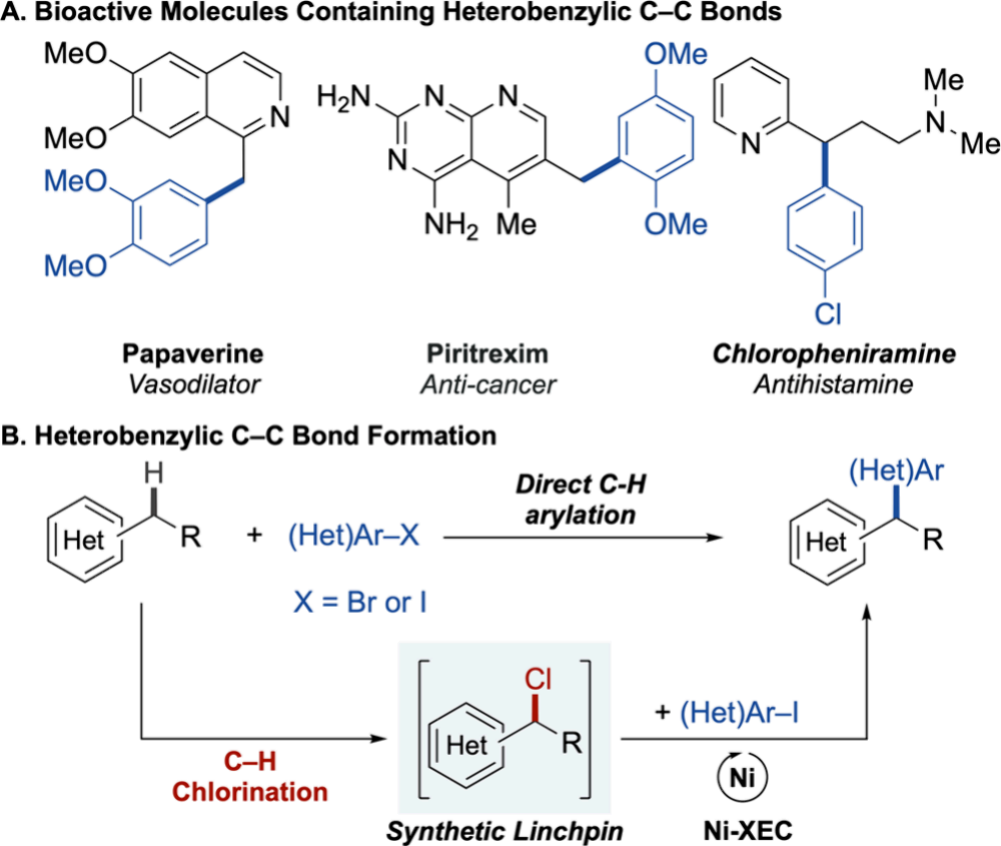
(A) Heteroaromatic small molecule drugs with heterobenzylic C–C bonds. (B) Use of heterobenzyl chloride linchpins to expand the scope and utility of heterobenzylic C–H arylation.

## Results and Discussion

The primary heterobenzyl chloride 2-chloro-4-(chloromethyl)­pyridine (**1a**) was selected for initial assessment of Ni-catalyzed arylation with 4-iodoethylbenzoate as the coupling partner. These substrates were tested under three previously reported Ni-XEC conditions that proceed effectively with benzyl chlorides (i.e., lacking the heteroatom in the ring).^18–20^ Two conditions that employ heterogeneous metal reductants (Zn^19^ and Mn^20^) afforded little to no yield of the desired cross-coupled product **1**. In contrast, conditions that use tetrakis­(dimethylamino)­ethylene (TDAE) as the reductant furnished the product in 93% yield (Figure 2A). This outcome likely arises from TDAE being a weaker reductant relative to metallic Zn and Mn (estimated potentials of −1.1, −1.5, and −1.6 V for TDAE, Zn, and Mn, respectively^21,22^ ), thereby minimizing deleterious side reactions. Further optimization of the reaction conditions, including changing the solvent to 1,4-dioxane, lowering the catalyst loading to 5 mol%, and using **1a** as the limiting reagent, led to near-quantitative yield of **1** (Figure 2B). These optimized conditions, designated Condition A, also led to near-quantitative yields of the regioisomeric products **2** and **3** (Figure 2B).

**Figure 2:**
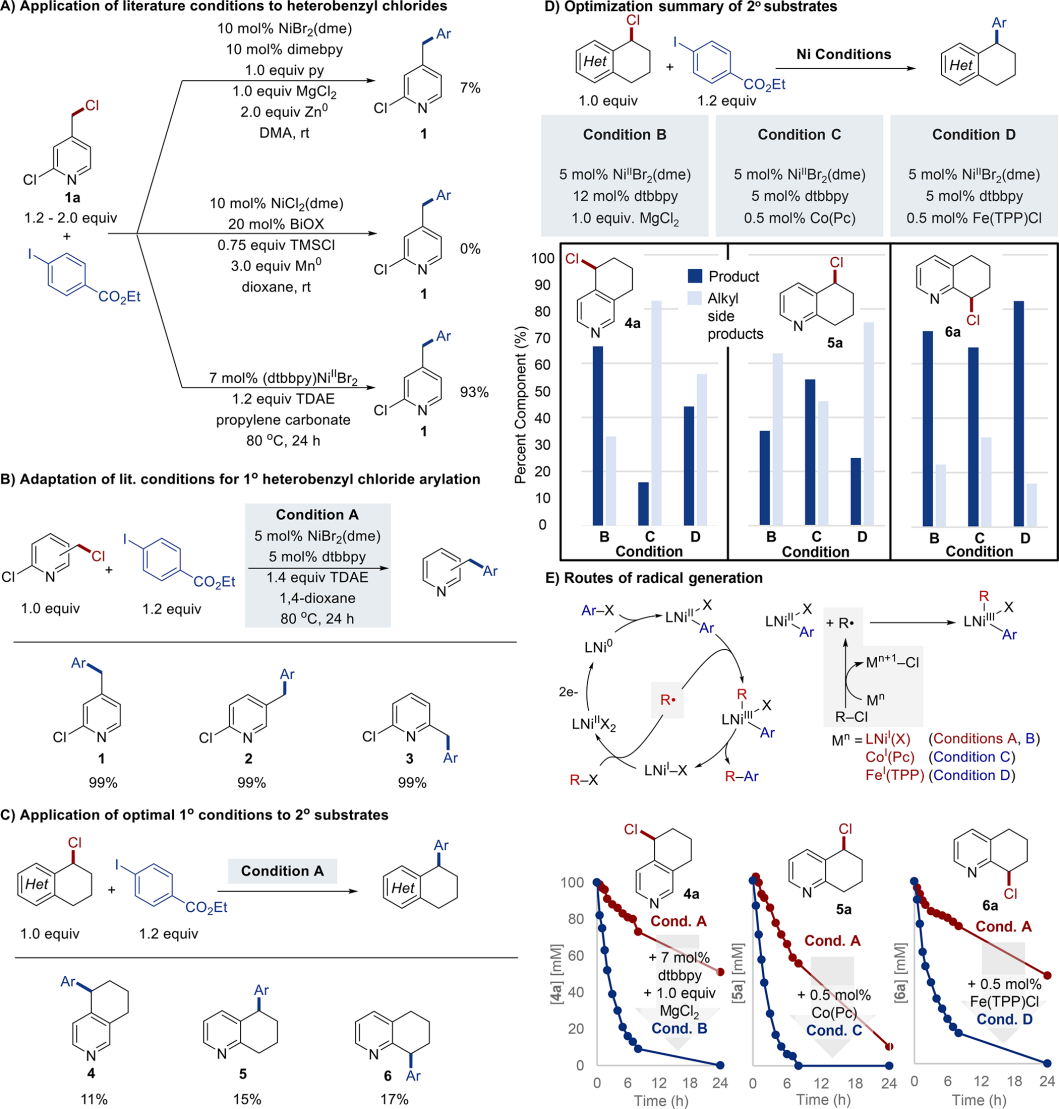
Optimization of heterobenzylic arylation. (A) Testing of three literature conditions in the arylation of **1a**. (B) Optimized conditions for arylation of primary heterobenzyl chloride isomers. (C) Ineffectiveness of Condition A with secondary heterobenzyl chlorides. (D) Three alternative conditions that are effective for secondary heterobenzyl chlorides. (E) Influence of additives on heterobenzyl chloride activation. Reactions in (A–D) were conducted with 0.1 mmol of **1a**–**6a** and 0.67 mL of 1,4-dioxane at 80 °C; other conditions are noted in the figure. ^1^H NMR yields are given (int. std. = mesitylene). Time course reactions in (E) were conducted at 0.3 mmol scale with 2.0 mL of 1,4-dioxane at 80 °C. Abbreviations: dme = 1,2-dimethoxyethane, dimebpy = 4,4′-dimethyl-2,2′-dipyridyl, BiOX = 2,2′-(cyclopropane-1,1-diyl)­bis­(3a,8a-dihydro-8H-indeno­[1,2-*d*]­oxazole), dtbbpy = 4,4′-di*tert*-butyl-2,2′-dipyridyl, TDAE = tetrakis­(dimethylamino)­ethylene, Pc = phthalocyanine, TPP = 5,10,15,20-tetraphenylporphyrin.

The excellent results obtained in reactions of **1a**–**3a** were not matched in reactions with secondary heterobenzyl chlorides (Figure 2C). The isomeric tetrahydroquinoline-derived heterobenzyl chlorides **4a**–**6a** afforded the corresponding arylation products **4**–**6** in yields of only 11–17%. To address this limitation, we screened other reaction conditions, varying the ancillary ligand, additives, and solvent (see Figures S2–S5 in the Supporting Information for details). Particular attention was given to two factors that could influence the reaction outcome: (i) the potential inhibitory effect of the pyridines and (ii) the relative rate of activation of the heterobenzyl chloride and aryl iodide coupling partners. Lewis acid additives were tested to offset the potential coordinating ability of pyridines, while cocatalysts were tested to modulate the relative rate of heterobenzyl chloride activation. The screening studies identified three new reaction conditions, designated Conditions B–D (Figure 2D). Condition B includes 1 equiv of MgCl_2_ as a Lewis acid and additional dtbbpy (12 mol%), and it proved the most effective with **4a**, affording a 67% yield of the XEC product **4**. The reduced steric profile of **4a**, which has no 2- or 6-substituent, may account for the beneficial influence of MgCl_2_ as a Lewis acid. Conditions C and D feature cobalt phthalocyanine (Co­(Pc)) and Fe­(TPP)Cl (TPP = tetraphenylporphyrin), respectively, as cocatalytic additives (0.5 mol%). Condition C proved most effective with **5a**, affording product **5** in 54% yield. All conditions led to good performance with **6a** (Figure 2D), but Condition D led to the highest yield of **6** (83%).

Analysis of side products formed under the different conditions shows how the variations influence substrate reactivity (Figures S7–S9), but perhaps the most notable observation is enhanced rate of the heterobenzyl chloride activation in each case relative to the original Condition A (Figure 2E). The rate enhancement with Condition B is attributed to attenuation of the inhibitory effect of the relatively unhindered pyridine, enabling the Ni catalyst to promote more effective activation of **4a**.^23^ Substrates **5a** and **6a** also undergo improved reactivity under Condition B, but use of the Co­(Pc) or Fe­(TPP) cocatalysts proved even more beneficial. Co­(Pc) is well established as a cocatalyst that promotes alkyl halide activation in Ni-XEC reactions,^24–28^ while Fe­(TPP)Cl has less precedent in this context. Fe porphyrins have been used previously to trap radicals and regulate their reaction rates in C­(sp^3^)–C­(sp^3^) coupling.^29,30^ Here, Fe­(TPP) promotes radical generation, playing a role similar to Co­(Pc) (see Figure S12 for CV studies supporting this reactivity).

The screening studies above show how even small variations in the substrate structure and/or position of the chloride relative to the heteroatom can have a significant influence on reactivity. Nonetheless, the set of complementary reaction conditions identified for the different substrate classes proved quite general in reactions with other substrates (Figure 3). Diverse primary chloromethyl-substituted pyridines (**1**–**3**, **7–15**) and other heterocycles, including pyrazine (**16**), quinazoline (**17**), and the tetrahydrobenzofuropyrimidine (**18**) react effectively under Condition A (Figure 3A). Substrates with both electron-withdrawing groups (**12–14**) and electron-donating groups (**7**, **9**) exhibit good reactivity. One exception is substrate **12**, which has a −CF_3_ group directly adjacent to the chloromethyl site, which proceeded in only 21% yield.

**Figure 3:**
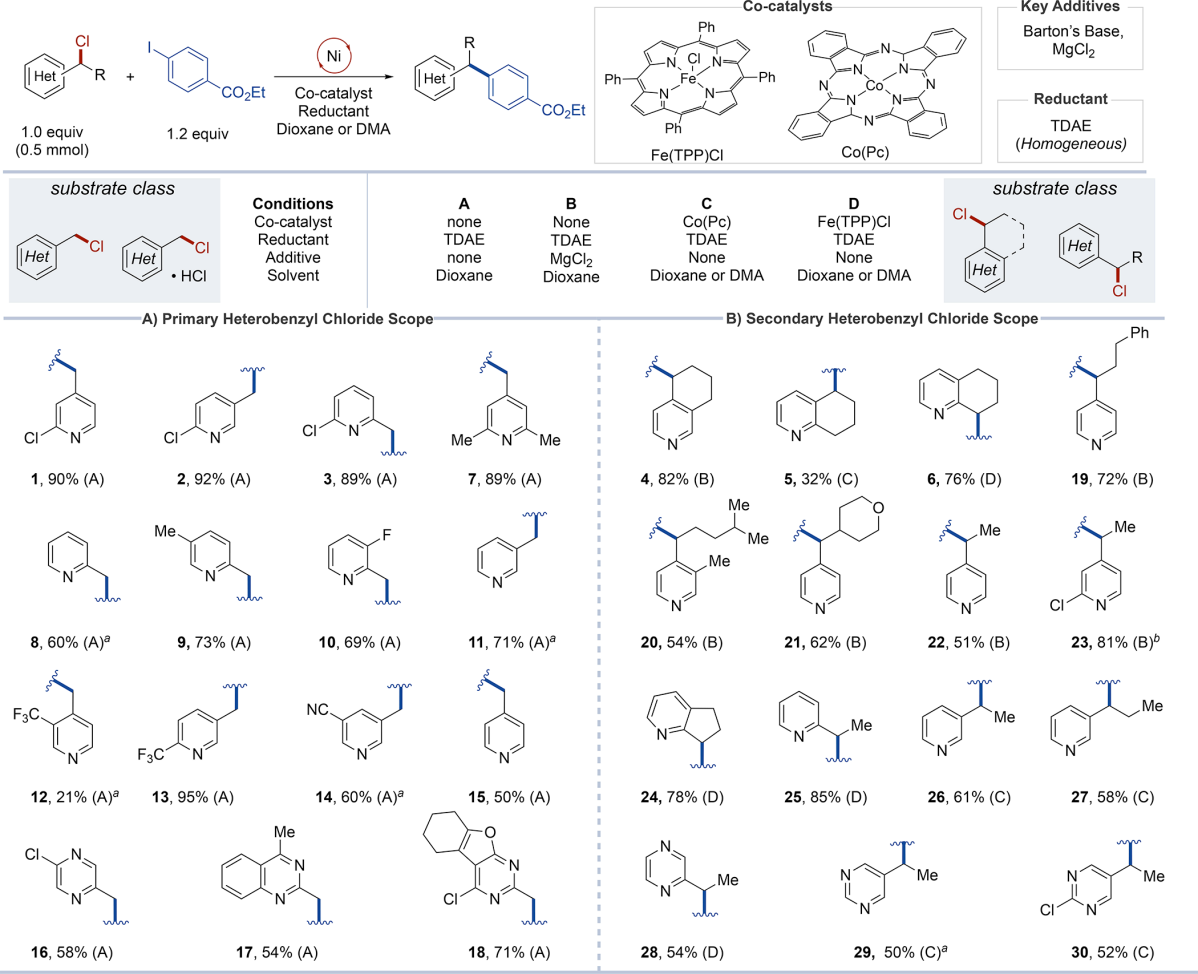
Scope of heterobenzylic arylation (0.5 mmol scale, solvent = DMA or dioxane, 80 °C, conditions A–D). All yields represent isolated yields. ^*a*^Benzyl chloride was employed as an HCl salt with 1 equiv of Barton’s base (2-*tert*-butyl-1,1,3,3-tetramethylguanidine). ^*b*^Reaction used Condition B without MgCl_2_.

Good results were also obtained with different secondary heterobenzyl chlorides, using Conditions B–D (Figure 3B). The preferred conditions generally align with the optimized conditions identified with the regiosomers **4a**–**6a**: 4-alkylated substrates proceed most effectively with Condition B (**4**, **19**–**23**), 3-alkylated substrates with Condition C (**5**, **26**, **27**), and 2-alkylated substrates with Condition D (**6**, **24**, **25**). Certain variations were also identified. For example, the formation of **23** in 81% yield did not require the inclusion of MgCl_2_ in Condition B, suggesting that substrates bearing a substituent adjacent to the pyridine nitrogen atom are less susceptible to poisoning of the Ni catalyst and can proceed without a Lewis acid. The conditions also support XEC with other classes of heterocycles, including pyrazine (**28**) and pyrimidines (**29**, **30**), and the conditions align with those observed with substituent positions on pyridines.

After demonstrating successful arylation of diverse heterobenzyl chlorides, efforts then turned to the chlorination/arylation of heterobenzylic C–H bonds (Figure 4). Prototypical heterobenzylic substrates with alkyl substituents in the 2-, 3-, and 4-positions were subjected to C–H chlorination^15,16^ to access **24a**, **30a**, and **4a**, respectively. Each of the heterobenzyl chlorides was subjected to the relevant Ni-XEC reaction conditions in a screening plate containing 30 different (hetero)­aryl iodides, curated to ensure the inclusion of substrates with medicinally relevant groups. The collection includes triazole (**1A**), pyridines (**1E**, **2D-E**, **4A-B**, **5A-C**), isoxazole (**3C**), indazole (**4C**), quinazoline (**3D**), indole (**5D**), quinolines (**3E-F**), thiazole (**4E**), substituted iodothienopyrimidine (**5E**), iodouracil (**2F**), and benzothiazole (**5F**), among others. The reactivity data show broad coverage, with hit rates (corresponding to a product:internal standard liquid chromatography area percent of >0.1) of 83% for **24a**, 63% for **30a**, and 47% for **4a**. Generally, better outcomes were observed with electron-deficient aryl iodides relative to electron-rich derivatives (e.g., compare **5A**, **1C**, **1B** versus **1A**, **2C**, **4F**). To validate the high-throughput screening data, three XEC reactions with each heterobenzyl chloride substrate were performed in vials to enable product isolation (Figure 4, bottom). The results in Figure 4 were performed with purified samples of the heterobenzyl chlorides. Attempts to conduct the reactions as a sequential process without purification of the heterobenzyl chlorides^16^ led to lower yields and reproducibility issues. Nonetheless, these screening results show how heterobenzylic C–H chlorination/diversification may be implemented in the design of medicinal chemistry libraries, starting from readily available building blocks.

**Figure 4:**
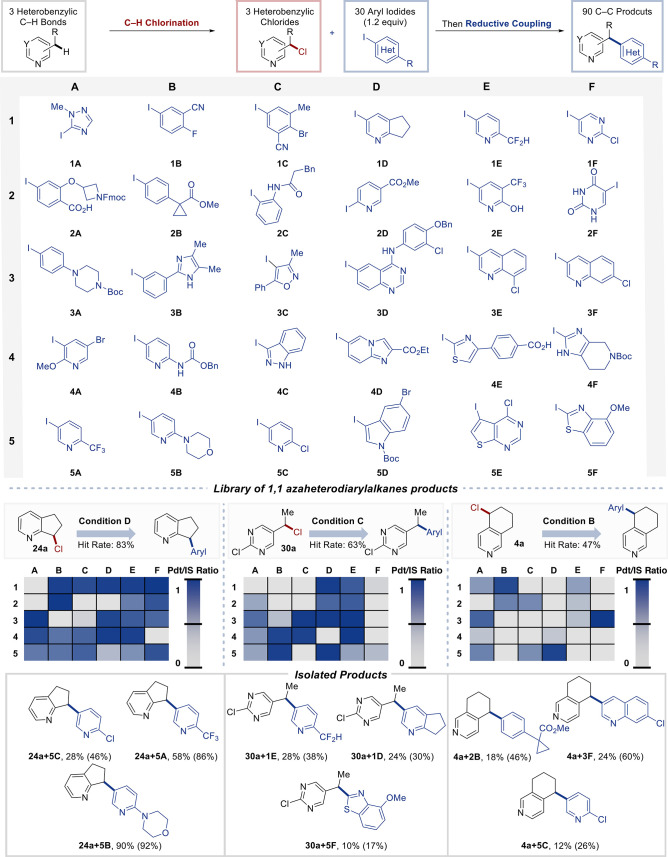
Scope of (hetero)­aryl iodides. Reactions were conducted on 10 μmol scale in dioxane at 80 °C using the conditions noted (B–D). Color scales represent the ratio of product absorbance to int. std. dibenzylaniline (1 equiv relative to benzyl chloride) absorbance at 210 nm. Hit rate is defined as >0.1 Pdt/Std Ratio. Isolated (and ^1^H NMR spectroscopic) yields are reported for selected products scaled up to 0.3 mmol.

## Conclusion

Overall, this work represents a notable expansion of Ni-catalyzed (hetero)­arylation reactions, providing methods that show good tolerance for heterocycles in both coupling partners. A small set of catalytic conditions has been identified that accommodates the intrinsically different heterobenzyl chloride reactivity, depending on its position on the heteroaromatic ring, and enables good reactivity at each site. More broadly, the results introduce a strategy for the use of heterobenzylic C­(sp^3^)–H bonds in cross coupling. Site-selective chlorination of the heterobenzylic C–H bonds is analogous to the emerging methods for activation of other latent functional groups, such as alcohols, amines, and carboxylic acids. Collectively, these results will support rapid diversification of pharmaceutical building blocks, thereby expanding accessible chemical space.

## Supplementary Material



## Notes

Experimental procedures, additional screening data, and compound characterization data (PDF)
