# Supplementary material for: Heterobenzyl Chlorides as Linchpins for C–H Arylation via Sequential C–H Chlorination/Cross-Electrophile Coupling
Source: ACS Catal. 2026 Feb 3;16(4):3081–7. doi: 10.1021/acscatal.5c08221 (PMC12930391; doi:10.1021/acscatal.5c08221)

Supporting Information

Heterobenzyl Chlorides as Linchpins for C–H Arylation via Sequential C–H Chlorination/Cross-Electrophile Coupling

Jack T. Floreancig,[a‡] Marco A. Lopez,[a‡] Allison R. Dick,[b] Luana Cardinale,[a] Nicole C. Goodwin,[c] Darren L. Poole,[d] and Shannon S. Stahl[a*]

[a] Department of Chemistry, University of Wisconsin−Madison, 1101 University Avenue, Madison, Wisconsin 53706, United States
[b] Chemistry Department, Wheaton College, 501 College Avenue, Wheaton, Illinois 60187, United States
[c] GSK, 1250 South Collegeville Road, Collegeville, Pennsylvania 19426 United States
[d] Molecular Modalities Capabilities, GSK Medicines Research Centre, Stevenage, SG1 2NY, UK

email: *stahl@chem.wisc.edu, ‡J.T.F. and M.A.L. contributed equally.

Table of Contents:

1. **General Considerations** S2
2. **General Procedure** S3
3. **Substrate Synthesis** S5
4. **Optimization of Reaction Conditions** S13
5. **Cyclic Voltammogram Experiments** S25
6. **Time Course Experiments** S27
7. **Assessment of Heteroaryl iodides** S28
8. **Product Synthesis and Characterization** S33
9. **References** S53
10. **Spectral Data** S54

1. General Considerations

All reagents were purchased from commercial sources and used without further purification. Anhydrous *N*,*N*-dimethylformamide (DMF, 99.9%), anhydrous *N*,*N*-dimethylacetamide (DMA, 99.9%), 1,3-dimethyl-2-imidazolidinone (DMI, 98%), and 1,4-dioxane (99.8%) were purchased from Sigma-Aldrich. Solvents were opened and stored in a nitrogen-filled glovebox and used as is. $NiBr_2$•dme and $NiCl_2$•dme were purchased from Sigma-Aldrich. 5,10,15,20-tetraphenyl-21*H*,23*H*-porphine iron(III) chloride (Fe(TPP)Cl) and cobalt(II) phthalocyanine (Co(Pc)) were purchased from Sigma-Aldrich or Alfa Aesar. Tetrakis(dimethylamino)ethylene (TDAE), zinc powder (-325 mesh, 97%), and manganese powder (-325 mesh) were purchased from Sigma-Aldrich and stored in the glove box. Ligands used in reaction optimizations and scope investigations were purchased from Sigma-Aldrich, Ambeed, or Alfa Aesar. Heterobenzyl chlorides **16a-24a**, **26a,** and **29a** were purchased from Ambeed. Substrate **30a** was purchased from Key Organics. Preparations and characterizations of synthesized starting materials are described in *Section 3*.

All cyclic voltammetry (CV) experiments were performed using a Pine WaveNow PGstat. The CV experiments were carried out in a three-electrode cell configuration with a glassy carbon (GC) working electrode (3 mm diameter), and a platinum wire counter electrode (~1.0 cm, spiral wire). The working electrode potentials were measured versus $Ag/AgNO_3$ reference electrode (internal solution, 0.1 M Bu_4NPF_6 and 0.01 M $AgNO_3$ in DMA). The redox potential of ferrocene/ferrocenium (Fc/Fc^+) was measured (under the same experimental conditions) and used to provide an internal reference. The potential values were then adjusted relative to Fc/Fc^+, and electrochemical studies in organic solvents were recorded accordingly. The GC working electrode was polished with alumina powder (5 µm) before each experiment.

Proton (^1H), carbon (^{13}C), and fluorine (^{19}F) nuclear magnetic resonance (NMR) spectra were recorded on a Bruker Avance 400 MHz spectrometer at 25 °C (^1H 400.1 MHz, ^{13}C 100.6 MHz, ^{19}F 376.5 MHz), Bruker Avance 500 MHz spectrometer at 25 °C (^1H 500.1 MHz, ^{13}C 125.7 MHz, ^{19}F 470.6 MHz), or a Bruker Avance 600 MHz spectrometer at 25 °C (^1H 600.1 MHz, ^{13}C 151.1 MHz). All spectra and chemical shifts are reported in parts per million (ppm). NMR spectra are referenced to residual solvent $CHCl_3$ at 7.26 ppm (^1H) and $CDCl_3$ at 77.16 ppm (^{13}C). All ^{19}F NMR spectra were absolutely referenced to their respective solvent peaks in the ^1H NMR spectrum. UPLC-MS analysis was conducted on a Waters-Acquity High resolution mass spectrometer. High resolution mass spectrometry data were obtained on a Thermo Q Exactive™ Plus by the mass spectrometry facility staff at the University of Wisconsin-Madison. Automatic normal phase column chromatography was performed using reusable 25g Sfär Silica HC D cartridges on a Biotage Isolera®. Preparative thin-layer chromatography was performed using 20 cm x 20 cm glass-backed silica preparative thin-layer chromatography plates (1000 um, layer thickness) from Miles Scientific.

2. General Procedures for Heterobenzyl Chloride Arylation

Reductive Arylation of Primary 2-, 3-, and 4-Alkyl Substituted Heterobenzyl Chlorides

General Procedure A: On the benchtop, an oven-dried 24 mL borosilicate vial was charged with $NiBr_2$•dme (7.7 mg, 5 mol%), dtbbpy (6.71 mg, 5 mol%) (dtbbpy = 4,4'-di(*tert*-butyl)-2,2'-bipyridine), heterobenzylic chloride (0.5 mmol, 1 equiv), ethyl 4-iodobenzoate (0.6 mmol, 1.2 equiv), and a Teflon stir bar. The vial was sparged with N_2 then transferred into a purging glovebox under N_2. In the glove box, the vial was charged with 1,4-dioxane (3.33 mL, 0.15 M), Barton's base if the substrate was employed as its HCl salt (101 µL, 0.5 mmol, 1 equiv), and TDAE (163 µL, 0.7 mmol, 1.4 equiv). The reaction vial was then sealed, removed from the glovebox, and set to stir at 750 rpm on an aluminum block at 80 ºC for 24 h.

Work Up: The crude reaction mixture was filtered through a 40 micron disposable polyethylene frit, washed with ethyl acetate, and concentrated on the rotovap. The obtained residue was diluted with ethyl acetate (10 mL), transferred to a separatory funnel, and washed with saturated $NaHCO_3$ (aq) (3 x 10 mL), dried over sodium sulfate, and concentrated on the rotovap. The obtained residue was purified by flash column chromatography (see Section 8 for details).

Reductive Arylation of Secondary 4-Alkyl-Substituted Heterobenzyl Chlorides

General Procedure B: On the benchtop, an oven-dried 6-dram borosilicate vial was charged with heterobenzylic chloride (0.5 mmol, 1 equiv), ethyl 4-iodobenzoate (0.6 mmol, 1.2 equiv), and a Teflon stir bar. $NiBr_2$•dme (15.4 mg, 0.05 mmol) and dtbbpy (32.2 mg, 0.12 mmol) were weighed into a secondary 2-dram vial with a cross-shaped stir bar. Both vials were sparged with N_2 then transferred into a purging glovebox under N_2. In the glovebox, 1,4-dioxane (6.5 mL, 7 mM) was added to the vial containing $NiBr_2$•dme and dtbbpy and stirred for 10 min to form a blue-green catalyst stock solution. To the vial containing benzylic chloride, aryl iodide, and the Teflon stir bar was added $MgCl_2$ (48 mg, 1 equiv), the catalyst stock solution (3.5 mL), and TDAE (163 µL, 0.7 mmol, 1.4 equiv). The reaction vial was then sealed, removed from the glovebox, and set to stir at 750 rpm on an aluminum block at the 80 °C for 16 h or 24 h (see Section 8 for details).

Work Up: The crude reaction mixture was filtered through a 40 micron disposable polyethylene frit, washed with ethyl acetate, and concentrated on the rotovap. The obtained residue was diluted with ethyl acetate (10 mL), transferred to a separatory funnel, and washed with saturated $NaHCO_3$ (aq) (3 x 10 mL), dried over sodium sulfate, and concentrated on the rotovap. The obtained residue was purified by flash column chromatography (see Section 8 for details).

Reductive Arylation of Secondary 3-Alkyl-Substituted Heterobenzyl Chlorides

General Procedure C: On the benchtop, an oven-dried 6-dram borosilicate vial was charged with heterobenzylic chloride (0.5 mmol, 1 equiv), ethyl 4-iodobenzoate (0.6 mmol, 1.2 equiv), and a Teflon stir bar. $NiBr_2$•dme (15.4 mg, 0.05 mmol), Co(Pc) (2.8 mg, 5 µmol), and dtbbpy (13.4 mg, 0.05 mmol) were weighed into a secondary 2-dram vial with a cross-shaped stir bar. Both vials were sparged with N_2 then transferred into a purging glovebox under N_2. In the glovebox, 1,4-dioxane (6.5 mL) was added to the vial containing $NiBr_2$•dme, Co(Pc), and dtbbpy and stirred for 10 min to from a deep purple stock solution. To the vial containing benzylic chloride, aryl iodide, and the Teflon stir bar was added the catalyst stock solution (3.5 mL) and TDAE (163 µL, 0.7 mmol, 1.4 equiv). The reaction vial was then sealed, removed from the glovebox, and set to stir at 750 rpm on an aluminum block at the specified temperature (see Section 8 for details) for 16 h.

Work Up: The crude reaction mixture was filtered through a 40 micron disposable polyethylene frit, washed with ethyl acetate, and concentrated on the rotovap. The obtained residue was diluted with ethyl acetate (10 mL), transferred to a separatory funnel, and washed with saturated $NaHCO_3$ (aq) (3 x 10 mL), dried over sodium sulfate, and concentrated on the rotovap. The obtained residue was purified by flash column chromatography (see Section 8 for details).

Reductive Arylation of Secondary 2-Alkyl-Substituted Heterobenzyl Chlorides

General Procedure D: On the benchtop, an oven-dried 6-dram borosilicate vial was charged with heterobenzylic chloride (0.5 mmol, 1 equiv), ethyl 4-iodobenzoate (0.6 mmol, 1.2 equiv), and a Teflon stir bar. $NiBr_2$•dme (15.4 mg, 0.05 mmol), Fe(TPP)Cl (3.5 mg, 5 µmol), and dtbbpy (13.4 mg, 0.05 mmol) were weighed into a secondary 2-dram vial with a cross-shaped stir bar. Both vials were sparged with N_2 then transferred into a purging glovebox under N_2. In the glovebox, 1,4-dioxane (6.5 mL) was added to the vial containing $NiBr_2$•dme, Fe(TPP)Cl, and dtbbpy and stirred for 10 min to from a dark brown catalyst stock solution. To the vial containing benzylic chloride, aryl iodide, and the Teflon stir bar was added the catalyst stock solution (3.5 mL) and TDAE (163 µL, 0.7 mmol, 1.4 equiv). The reaction vial was then sealed, removed from the glovebox, and set to stir at 750 rpm on an aluminum block at the specified temperature (see Section 8 for details) for 16 h.

Work Up: The crude reaction mixture was filtered through a 40 micron disposable polyethylene frit, washed with ethyl acetate, and concentrated on the rotovap. The obtained residue was diluted with ethyl acetate (10 mL), transferred to a separatory funnel, and washed with saturated $NaHCO_3$ (aq) (3 x 10 mL), dried over sodium sulfate, and concentrated on the rotovap. The obtained residue was purified by flash column chromatography (see Section 8 for details).

General Procedure E: On the benchtop, an oven-dried 8-mL borosilicate vial was charged with $NiCl_2$•dme (11.0 mg, 0.05 mmol, 10 mol%), dttbpy (13.4 mg, 0.05 mmol, 10 mol%), heterobenzylic chloride (0.5 mmol, 1 equiv), ethyl 4-iodobenzoate (0.6 mmol, 1.2 equiv), and a crossed shaped Teflon stir bar. The vial was sparged with N_2 then transferred into a purging glovebox under N_2. In the glove box, the vial was charged with LiCl (21.2 mg, 0.5 mmol, 1 equiv) and DMA (2.5 mL, 0.2 M) and allowed to stir for 10 min. Then, magnesium powder (24.3 mg, 1.0 mmol, 2 equiv) was added to the vial. The reaction vial was then sealed, removed from the glovebox, and set to stir at 1000 rpm on an aluminum block at the specified temperature (see Section 8 for details) for 16 h.

Work Up: The crude reaction mixture was filtered through a 40 micron disposable polyethylene frit, washed with ethyl acetate, and concentrated on the rotovap. The obtained residue was diluted with ethyl acetate (10 mL), transferred to a separatory funnel, and washed with 5% LiCl (aq) (10 mL), saturated $NaHCO_3$ (10 mL), and brine (10 mL), dried over sodium sulfate, and concentrated on the rotovap. The obtained residue was purified by flash column chromatography (see Section 8 for details).

3. Substrate Synthesis and Characterization

General Procedure F: C–H Chlorination of 4-Alkyl Substituted Pyridines

On the benchtop, a disposable 24 mL glass vial was charged with *N*-chlorosuccinimide (1.2 equiv, 1.2 mmol), Li_2CO_3 (1.1 equiv), 1.1 mmol), 4-(dimethylamino)pyridine (10 mol%, 0.1 mmol), C–H substrate (1 equiv, 1 mmol), anhydrous acetonitrile (0.1 M, 10 mL), a Teflon stir bar, and trifluoromethanesulfonyl chloride (TfCl, 10 or 40 mol%, 0.1 or 0.4 mmol). The reaction vial was then sealed and set to stir at 750 rpm on an aluminum block at the specified temperature (see each substrate below for details). After 4 or 16 h the reaction was allowed to cool, filtered through a pad of celite, and concentrated via rotary evaporation. The obtained residue was diluted with ethyl acetate (10 mL), transferred to a separatory funnel, and washed with saturated $NaHCO_3$ (aq) (10 mL). The organic layer was collected, dried over Na_2SO_4, and concentrated via rotary evaporation. The obtained residue was purified by flash column chromatography on silica gel (see below for details).

General Procedure G: C–H Chlorination of 2-Alkyl Substituted Pyridines

An oven-dried 100 mL round-bottom flask containing a magnetic stir bar was charged with 2.0 g of C–H substrate (1.0 equiv, 15 mmol), 40 mL of anhydrous DCM (0.3 M), and 4.2 g of trichloroisocyanuric acid (1.2 equiv, 18 mmol). The flask was fitted with a Findenser™ and refluxed under air. After 16 h, the reaction was allowed to cool, filtered through a pad of celite, and concentrated on the rotovap. The obtained residue was diluted with ethyl acetate (20 mL), transferred to a separatory funnel, and washed with saturated $NaHCO_3$ (aq) (20 mL). The organic layer was collected, dried over Na_2SO_4, and concentrated via rotary evaporation. The obtained residue was purified by flash column chromatography on silica gel (see below for details).

General Procedure H: C–H Chlorination of 3-Alkyl Substituted Pyridines

On the benchtop, *N*-(*tert*-butyl)-*N*-chloro-3,5-bis(trifluoromethyl)benzenesulfonamide (7.675 g, 20 mmol, 4.0 equiv) and heterobenzylic substrate (5.00 mmol, 1.0 equiv) were added to a 250-mL round bottom flask. The flask was then moved to a purging glove box under N_2 atmosphere. Cs_2CO_3 (2.444 g, 7.5 mmol, 1.5 equiv) and α,α,α-trifluorotoluene (25 mL) were added to the reaction flask. The flask was sealed with a septum, secured with electrical tape in the glove box and taken out to a photochemical setup. The reaction was carried out under irradiation of two blue LED lamps (~450 nm) with stirring for 20 h. When the reaction finished, mesitylene (70 µL, 0.50 mmol) was added as internal standard. After ^1H NMR analysis, the crude reaction was filtered through a silica plug using acetonitrile, evaporated under vacuum, and purified by column chromatography (see below for details).

Some heterobenzyl chloride substrates were synthesized by functional group interconversion, starting from the corresponding heterobenzylic ketone. Reduction to the alcohol followed by chlorination with $SOCl_2$ afforded the desired heterobenzylic chlorides. Yet other heterobenzyl chlorides were synthesized starting from the corresponding alcohol.

General Procedure I: $NaBH_4$ Reduction

To a solution of ketone (1.0 g, 1.0 equiv) dissolved in methanol (1.0 M) was added sodium borohydride (1.5 equiv) in small portions at 0 °C. The resulting mixture was allowed to stir at room temperature until complete by TLC analysis. When the starting material was consumed, the reaction was quenched with water (10 mL) and extracted with ethyl acetate (3 x 10 mL). The combined organic layer was washed with brine (10 mL), dried over sodium sulfate, filtered, and concentrated via rotary evaporation. The resulting residue was used without purification.

Heterobenzyl alcohols from commercial sources were chlorinated on a 1.0 g scale.

General Procedure J: Chlorination with $SOCl_2$
An oven-dried 25 mL round-bottom flask containing a magnetic stir bar was charged with heterobenzyl alcohol (1 equiv, made via General Procedure I or sourced commercially) and anhydrous dichloromethane (2.0 M). The flask was capped with a septum, placed under a positive pressure of nitrogen, and cooled to 0 ºC. Thionyl chloride (2 equiv) was added dropwise over a span of 15 min with stirring. After an additional 10 min, the reaction was allowed to warm to room temperature and stirred for an additional 2 h or until complete by TLC analysis. The reaction was then quenched with water (10 mL). The aqueous layer was neutralized with solid $NaHCO_3$, then extracted with diethyl ether (2 x 25 mL). The combined organic layers were dried with Na_2SO_4, filtered, and concentrated. The obtained residue was purified by column chromatography on silica gel (see below for details).

(4a) 5-chloro-5,6,7,8-tetrahydroisoquinoline

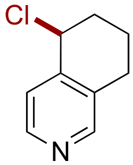

Reaction conducted following **Procedure F** using 5,6,7,8-tetrahydroisoquinoline (133.1 mg, 1.0 mmol, 1 equiv) and 10 mol% TfCl at 70 ºC for 16 h. Following workup, the obtained residue was purified by column chromatography on silica gel eluting with a solvent mixture of ethyl acetate:hexanes (40:60 (v:v)) to afford 119 mg (71% yield) of **4a** as a yellow oil.

NMR Spectroscopy of chloride:
^{1}H NMR (500 MHz, $CDCl_3$) δ 8.46 – 8.36 (m, 2H), 7.33 – 7.23 (m, 1H), 5.14 (t, *J* = 4.6 Hz, 1H), 2.93 – 2.83 (m, 1H), 2.80 – 2.69 (m, 1H), 2.30 – 2.10 (m, 3H), 1.93 – 1.84 (m, 1H).
^{13}C NMR (126 MHz, $CDCl_3$) δ 151.2, 147.5, 144.7, 132.0, 124.2, 55.8, 32.7, 25.9, 18.6.
HRMS (ESI) m/z: $[M+H]^+$ Calcd for $C_9H_{11}ClN$ 168.0575; found 168.0574.

(5a) 5-chloro-5,6,7,8-tetrahydroquinoline

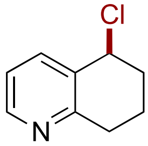

Reaction conducted following **Procedure I** using 7,8-dihydroquinolin-5(6*H*)-one (1.0 g, 6.8 mmol, 1 equiv) and **Procedure J**. Following workup, the obtained residue was purified by column chromatography on silica gel eluting with a solvent mixture of ethyl acetate:pentane (60:40 (v:v)) to afford 562 mg (58% yield) of **5a**.
Yield of benzyl alcohol: 85% (862 mg)
Yield of benzyl chloride: 58% (562 mg)

NMR Spectroscopy of chloride:
^{1}H NMR (500 MHz, $CDCl_3$) δ 8.44 – 8.39 (m, 2H), 7.30 (d, J = 5.2 Hz, 1H), 5.16 (t, J = 4.6 Hz, 1H), 2.91 (dt, J = 17.0, 5.2 Hz, 1H), 2.76 (ddd, J = 16.8, 9.3, 6.0 Hz, 2H), 2.34 – 2.22 (m, 2H), 2.22 – 2.10 (m, 1H), 1.96 – 1.87 (m, 1H).
^{13}C NMR (126 MHz, $CDCl_3$) δ 151.1, 147.5, 144.7, 132.0, 124.1, 55.8, 32.6, 25.9, 18.6.
HRMS (ESI) m/z: $[M+H]^+$ Calcd for $C_9H_{10}ClN$, 168.0575; Found 168.0573.

(6a) 8-chloro-5,6,7,8-tetrahydroquinoline

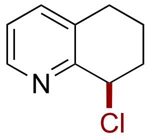

Reaction conducted following **Procedure G** using 5,6,7,8-tetrahydroquinoline (2.0 g, 15 mmol, 1 equiv). Following workup, the obtained residue was purified by column chromatography on silica gel eluting with a solvent mixture of ethyl acetate:pentane (30:70 (v:v)) to afford 2.1 g (83% yield) of **6a**. Spectral data matched those reported in the literature.[1]

NMR Spectroscopy of chloride:
^{1}H NMR (500 MHz, $CDCl_3$) δ 8.54 – 8.45 (m, 1H), 7.43 (dd, J = 7.8, 1.6 Hz, 1H), 7.14 (dd, J = 7.8, 4.6 Hz, 1H), 5.30 (d, J = 3.5 Hz, 1H), 2.94 – 2.84 (m, 1H), 2.84 – 2.69 (m, 1H), 2.43 – 2.27 (m, 1H), 2.31 – 2.17 (m, 2H), 1.90 (d, J = 5.3 Hz, 1H).
^{13}C NMR (126 MHz, $CDCl_3$) δ 154.7, 148.0, 137.7, 132.3, 123.4, 59.1, 32.6, 28.2, 17.6.

(16a) 2-chloro-5-(chloromethyl)pyrazine

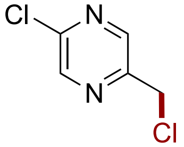

Reaction conducted following **Procedure J** using (5-chloropyrazin-2-yl) methanol (1.0 g, 6.9 mmol, 1 equiv). Following workup, the obtained residue was used without purification to afford 145 mg (89% yield) of **16a** as a yellow oil. Deviation from Procedure J is the use of 6 equiv of $SOCl_2$.

NMR Spectroscopy of chloride:
^{1}H NMR (500 MHz, $CDCl_3$) δ 8.55 (d, *J* = 1.5 Hz, 1H), 8.53 (d, *J* = 1.5 Hz, 1H), 4.69 (s, 2H).
^{13}C NMR (126 MHz, $CDCl_3$) δ 150.5, 149.1, 144.2, 143.6, 43.2.
HRMS (ESI) m/z: $[M+H]^+$ Calcd for $C_5H_4Cl_2N_2$, 162.9824; Found 162.9824.

(19a) 4-(1-chloro-3-phenylpropyl)pyridine

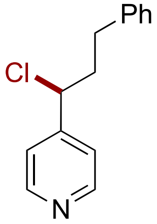

Reaction conducted following **Procedure F** using 4-(3-phenylpropyl)pyridine (197.2 mg, 1.0 mmol, 1 equiv) and 40 mol% TfCl at 70 ºC for 4 h. Following workup, the obtained residue was purified by column chromatography on silica gel eluting with a solvent mixture of ethyl acetate:hexanes (70:30 (v:v)) to afford 83 mg (36% yield) of **19a** as a yellow oil.

NMR Spectroscopy of chloride:
^{1}H NMR (500 MHz, $CDCl_3$) δ 8.71 – 8.55 (m, 2H), 7.37 – 7.17 (m, 7H), 4.76 (dd, J = 8.8, 5.4 Hz, 1H), 2.90 – 2.73 (m, 2H), 2.45 – 2.37 (m, 1H), 2.35 – 2.26 (m, 1H).
^{13}C NMR (126 MHz, $CDCl_3$) δ 150.3, 150.1, 140.1, 128.7, 128.5, 126.4, 121.8, 60.6, 41.0, 32.7.
HRMS (ESI) m/z: $[M+H]^+$ Calcd for $C_{14}H_{14}ClN$, 232.0888; Found 232.0887.

(20a) 3-methyl-4-(4-methylpentyl)pyridine

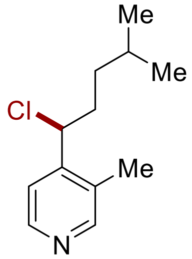

Reaction conducted following **Procedure F** using 3-methyl-4-(4-methylpentyl)pyridine (177.3 mg, 1.0 mmol, 1 equiv) and 10 mol% TfCl at 70 ºC for 4 h, except with the exclusion of DMAP. Following workup, the obtained residue was purified by column chromatography on silica gel eluting with a solvent mixture of ethyl acetate:hexanes (70:30 (v:v)) to afford 182 mg (86% yield) of **20a** as a yellow oil.

NMR Spectroscopy of chloride:
^{1}H NMR (500 MHz, $CDCl_3$) δ 8.47 (d, J = 5.2 Hz, 1H), 8.41 (s, 1H), 7.37 (d, J = 5.2 Hz, 1H), 4.98 (dd, J = 8.3, 6.0 Hz, 1H), 2.36 (s, 3H), 2.12 – 2.05 (m, 1H), 2.03 – 1.95 (m, 1H), 1.58 (dt, J = 13.3, 6.6 Hz, 1H), 1.46 – 1.38 (m, 1H), 1.23 – 1.12 (m, 1H), 0.89 (dd, J = 6.6, 1.4 Hz, 6H).
^{13}C NMR (126 MHz, $CDCl_3$) δ 151.5, 148.5, 148.4, 130.2, 120.0, 58.3, 36.3, 36.1, 27.8, 22.7, 22.5, 16.0.
HRMS (ESI) m/z: $[M+H]^+$ Calcd for $C_{12}H_{19}ClN$ 212.1201; found 212.1199.

(21a) 4-(chloro(tetrahydro-2*H*-pyran-4-yl)methyl)pyridine

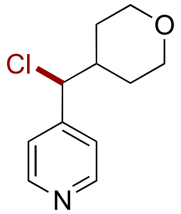

Reaction conducted following **Procedure F** using 4-((tetrahydro-2*H*-pyran-4-yl)methyl)pyridine (177.1 mg, 1.0 mmol, 1 equiv) and 40 mol% TfCl at 90 ºC for 4 h, except with the exclusion of DMAP. Following workup, the obtained residue was purified by column chromatography on silica gel eluting with a solvent mixture of ethyl acetate:hexanes (70:30 (v:v)) to afford 110.0 mg (52% yield) of **21a** as a clear oil.

NMR Spectroscopy of chloride:
^{1}H NMR (400 MHz, $CDCl_3$) δ 8.56 (dd, J = 6.0, 1.7 Hz, 2H), 7.22 (dd, J = 6.0, 1.7 Hz, 2H), 4.50 (d, J = 8.2 Hz, 1H), 4.08 – 4.00 (dd, J = 11.7, 3.5 Hz, 1H), 3.92 (dd, J = 11.5, 2.9 Hz, 1H), 3.32 (td, J = 12.0, 2.2 Hz, 1H), 3.22 – 3.20 (td, , J = 11.9, 2.2 Hz, 1H), 2.19 – 1.90 (m, 2H), 1.60 – 0.97 (m, 3H).
^{13}C NMR (101 MHz, $CDCl_3$) δ 150.3, 148.5, 122.5, 67.7, 67.2, 66.5, 43.0, 30.4, 30.0.
HRMS (ESI) m/z: $[M+H]^+$ Calcd for $C_{11}H_{14}ClNO$, 212.0837; Found 212.0835.

(22a) 4-(1-chloroethyl)pyridine

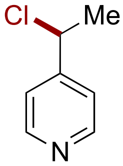

Reaction conducted following **Procedure J** using 1-(pyridin-4-yl)ethan-1-ol (1.0 g, 8.1 mmol, 1 equiv) Following workup, the obtained residue was purified by column chromatography on silica gel eluting with a solvent mixture of ethyl acetate:hexanes (70:30 (v:v)) to afford 118 mg (83% yield) of **22a** as a yellow oil.

NMR Spectroscopy of chloride:
^{1}H NMR (500 MHz, $CDCl_3$) δ 8.61 (d, J = 6.2 Hz, 2H), 7.33 (d, J = 6.2 Hz, 2H), 5.00 (q, J = 6.9 Hz, 1H), 1.82 (d, J = 6.9 Hz, 3H).
^{13}C NMR (126 MHz, $CDCl_3$) δ 151.3, 150.2, 121.3, 56.4, 26.1.
The spectra are in agreement with previous literature reports.[2]

(23a) 2-chloro-4-(1-chloroethyl)pyridine

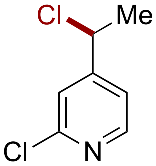

Reaction conducted following **Procedure I** using 1-(2-chloropyridin-4-yl)ethan-1-one (1.0 g, 6.4 mmol, 1 equiv) and **Procedure J**. Following workup, the obtained residue was purified by column chromatography on silica gel eluting with a solvent mixture of ethyl acetate:pentane (20:80 (v:v)) to afford 735 mg (66% yield) of **23a**.
Yield of benzyl alcohol: quantitative (1.0 g)
Yield of benzyl chloride: 66% (735 mg)

NMR Spectroscopy of chloride:
^{1}H NMR (500 MHz, $CDCl_3$) δ 8.39 (s, 1H), 7.38 (s, 1H), 7.27 (s, 1H), 4.96 (q, J = 6.7 Hz, 1H), 1.82 (d, J = 6.7 Hz, 3H).
^{13}C NMR (126 MHz, $CDCl_3$) δ 154.5, 152.1, 150.3, 122.1, 120.3, 55.6, 26.1.
HRMS (ESI) m/z: $[M+H]^+$ Calcd for $C_7H_7Cl_2N$, 176.0028; Found 176.0029.

(24a) 7-chloro-6,7-dihydro-5*H*-cyclopenta[*b*]pyridine

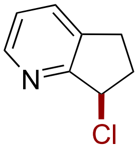

Reaction conducted following **Procedure G** using 6,7-dihydro-5*H*-cyclopenta[*b*]pyridine (2.0 g, 16.8 mmol, 1 equiv). Following workup, the obtained residue was purified by column chromatography on silica gel eluting with a solvent mixture of ethyl acetate:pentane (30:70 (v:v)) to afford 1.8 g (69% yield) of **24a**.

NMR Spectroscopy:

^{1}H NMR (500 MHz, $CDCl_3$) δ 8.55 – 8.48 (m, 1H), 7.67 – 7.53 (m, 1H), 7.17 (td, J = 7.1, 4.6 Hz, 1H), 5.35 (td, J = 7.1, 2.9 Hz, 1H), 3.20 (qt, J = 9.0, 7.2 Hz, 1H), 3.08 – 2.85 (m, 1H), 2.79 – 2.57 (m, 1H), 2.42 (dtt, J = 14.0, 7.8, 3.1 Hz, 1H).
^{13}C NMR (126 MHz, $CDCl_3$) δ 162.3, 149.1, 136.7, 133.3, 123.4, 62.1, 34.9, 28.4.
HRMS (ESI) m/z: $[M+H]^+$ Calcd for C_8H_8ClN, 154.0418; Found 154.0416.

(25a) 2-(1-chloroethyl)pyridine

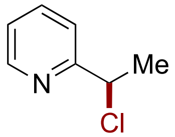

Reaction conducted following **Procedure J** using 1-(pyridin-2-yl)ethan-1-ol (1.0 g, 8.1 mmol, 1 equiv), 10 mol% TfCl, at 70 ºC for 4 h. Following workup, the obtained residue was purified by column chromatography on silica gel eluting with a solvent mixture of ethyl acetate:hexanes (30:70 (v:v)) to afford 120 mg (85% yield) of **25a** as a yellow oil.

NMR Spectroscopy of chloride:
^{1}H NMR (500 MHz, $CDCl_3$) δ 8.59 (ddd, J = 4.8, 1.8, 0.9 Hz, 1H), 7.73 (td, J = 7.7, 1.8 Hz, 1H), 7.51 (dt, J = 7.9, 1.1 Hz, 1H), 7.24 (ddd, J = 7.5, 4.8, 1.1 Hz, 1H), 5.17 (q, J = 6.9 Hz, 1H), 1.90 (d, J = 6.8 Hz, 3H).
^{13}C NMR (126 MHz, $CDCl_3$) δ 161.0, 149.2, 137.2, 123.1, 121.3, 59.1, 25.1.
HRMS (ESI) m/z: $[M+H]^+$ Calcd for C_7H_8ClN, 142.0418; Found 142.0418.

(26a) 3-(1-chloroethyl)pyridine

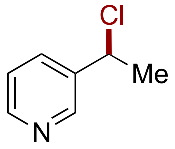

Reaction conducted following **Procedure J** using 1-(pyridin-3-yl)ethan-1-ol (1.0 g, 8.1 mmol, 1 equiv), 10 mol% TfCl, at 70 ºC for 4 h. Following workup, the obtained residue was purified by column chromatography on silica gel eluting with a solvent mixture of ethyl acetate:hexanes (70:30 (v:v)) to afford 127 mg (90% yield) of **26a** as a yellow oil. The spectra are in agreement with previous literature reports.[3]

NMR Spectroscopy of chloride:
^{1}H NMR (500 MHz, $CDCl_3$) δ 8.64 (d, J = 2.4 Hz, 1H), 8.55 (dd, J = 4.8, 1.6 Hz, 1H), 7.80 – 7.75 (m, 1H), 7.30 (ddd, J = 7.9, 4.7, 0.9 Hz, 1H), 5.10 (q, J = 6.8 Hz, 1H), 1.87 (d, J = 6.9 Hz, 3H).
^{13}C NMR (126 MHz, $CDCl_3$) δ 149.7, 148.2, 138.5, 134.2, 123.7, 55.9, 26.5.
HRMS (ESI) m/z: $[M+H]^+$ Calcd for $[C_7H_9ClN]^+$: 142.0418, Found: 142.0419.

(27a) 3-(1-chloropropyl)pyridine

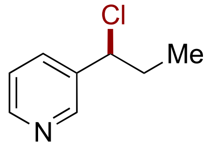

Reaction conducted following **Procedure I** using 1-(pyridin-3-yl)propan-1-one (1.0 g, 7.4 mmol, 1 equiv) and **Procedure J**. Following workup, the obtained residue was purified by column chromatography on silica gel eluting with a solvent mixture of ethyl acetate:pentane (60:40 (v:v)) to afford 678 mg (62% yield) of **27a**.
Yield of benzyl alcohol: 95% (964 mg)
Yield of benzyl chloride: 62% (678 mg)

NMR Spectroscopy for chloride:
^{1}H NMR (400 MHz, $CDCl_3$) δ 8.66 – 8.50 (m, 2H), 7.74 (dt, J = 8.0, 1.9 Hz, 1H), 7.35 – 7.28 (m, 1H), 4.80 (dd, J = 8.0, 6.4 Hz, 1H), 2.24 – 1.93 (m, 2H), 1.02 (t, J = 7.3 Hz, 3H).
^{13}C NMR (101 MHz, $CDCl_3$) δ 149.7, 148.6, 137.3, 134.6, 123.7, 62.5, 33.2, 11.6.
HRMS (ESI) m/z: $[M+H]^+$ Calcd for $C_8H_{10}ClN$, 156.0574; Found 156.0575.

(28a) 2-(1-chloroethyl)pyrazine

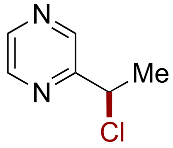

Reaction conducted following **Procedure I** using 1-(pyrazin-2-yl)ethan-1-one (1.0 g, 8.2 mmol, 1 equiv) and **Procedure J**. Following workup, the obtained residue was purified by column chromatography on silica gel eluting with a solvent mixture of ethyl acetate:pentane (40:60 (v:v)) to afford 81 mg (63% yield) of **28a**. Spectral data match those reported in the literature.[4]
Yield of benzyl alcohol: 90% (112 mg)
Yield of benzyl chloride: 63% (81 mg)

NMR Spectroscopy of chloride:
^{1}H NMR (500 MHz, $CDCl_3$) δ 8.78 (d, J = 1.5 Hz, 1H), 8.63 – 8.44 (m, 2H), 5.18 (q, J = 6.9 Hz, 1H), 1.92 (d, J = 6.9 Hz, 3H).
^{13}C NMR ($CDCl_3$, 126 MHz): δ 156.3, 144.1, 143.9, 143.4, 56.1, 24.3.

(29a) 5-(1-chloroethyl)pyrimidine

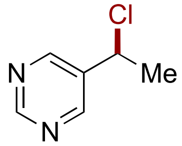

Reaction conducted following **Procedure I** using 1-(pyrimidin-5-yl)ethan-1-one (1.0 g, 8.2 mmol, 1 equiv) and **Procedure J**. The reaction mixture resulting from **Procedure J** was directly evaporated under a stream of dry nitrogen and then washed with ether to give a brown solid of **29a** as its HCl salt. 45 mg (32% yield) of **29a**.
Yield of benzyl alcohol: 98% (121 mg)
Yield of benzyl chloride: 32% (45 mg)

NMR Spectroscopy:
^{1}H NMR (500 MHz, DMSO-d_6): δ 9.15 (s, 1H), 8.79 (s, 2H), 5.06 (q, J = 6.9 Hz, 1H), 1.88 (d, J = 6.9 Hz, 3H) ppm.
^{13}C NMR (126 MHz, DMSO-d_6) δ 158.5, 156.0, 136.7, 54.3, 25.4.
HRMS (ESI) m/z: $[M+H]^+$ Calcd for $[C_6H_8ClN_2]^+$: 143.0370, Found: 143.0369.

(30a) 2-chloro-5-(1-chloroethyl)pyrimidine

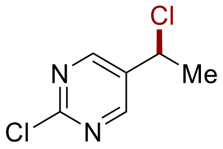

Reaction conducted following **Procedure H** using 2-chloro-5-ethylpyrimidine (713 mg, 5.0 mmol, 1 equiv). The reaction mixture resulting from **Procedure H** was purified by column chromatography on silica gel eluting with a solvent mixture of ethyl acetate:pentane (20:80 (v:v)) to afford 449 mg (63% yield) of **30a**.

NMR Spectroscopy:
^{1}H NMR ($CDCl_3$, 500 MHz): δ 8.67 (s, 2H), 5.07 (q, J = 6.9 Hz, 1H), 1.88 (d, J = 6.9 Hz, 3H).
^{13}C NMR ($CDCl_3$, 126 MHz): δ 160.9, 157.9, 134.9, 52.0, 25.8.
The spectra are in agreement with previous literature reports.[3]

4. Optimization of Reaction Conditions

General Procedure for Optimization of Reaction Parameters (0.1 mmol scale)

On the benchtop, an oven-dried 1-dram borosilicate vial was charged with heterobenzylic chloride (1 equiv), ethyl 4-iodobenzoate (1.2 equiv), and a Teflon stir bar. The Ni pre-catalyst and appropriate ligand were weighed into a secondary 2-dram vial with a cross-shaped stir bar. Both vials were sparged with N_2 and then transferred into a purging glovebox under N_2. In the glovebox, solvent (1.3 mL) was added to the vial containing Ni and ligand and stirred for 10 min to form a stock solution. To the vial containing heterobenzylic chloride, aryl halide, and the Teflon stir bar were added salt additive, the catalyst stock solution (0.7 mL), and reductant. The reaction vial was then sealed, removed from the glovebox, and set to stir at 750 rpm on an aluminum block at the specified temperature for 16 h. See the optimization tables below for additional details.

Work Up: After 24 h, an aliquot (3 µL) of the reaction was filtered into an HPLC collection block (Analytical Sales and Services) using MeCN (300 µL) giving a concentration of ~1.0 mM. It was then analyzed utilizing UPLC-MS (Waters-Acquity) analysis. Yields were determined as a percentage of product relative to all known species derived from the heterobenzyl chloride.

General Procedure for HTE Optimization

On the bench top, to individual 2-dram vials fitted with cross-shaped stir bars were added the Ni pre-catalyst (0.06 mmol), appropriate nitrogen-donor ligand (0.01 mmol), heterobenzylic chloride (1.2 mmol), and aryl iodide (1.4 mmol). These vials were then transferred into a nitrogen-filled glove box and solvent was added (Ni: 2.4 mL, ligand: 0.4 mL, HetBn-Cl: 1.2 mL, Ar-I: 1.2 mL) to give separate stock solutions of each reaction component. In the glove box, to separate 2-dram vials fitted with a cross-shaped stir bar were added the appropriate additive (0.4 mmol for stoichiometric salt additives or 2.0 µmol for Co or Fe co-catalysts) and reductant (1.7 mmol), followed by solvent (additive: 1.6 mL, reductant: 1.2 mL) to give separate stock solutions of these reaction components. These stock solutions were stirred for 15 min. To a 96-well optimization block (Analytical Sales and Services) with 1 mL glass vial inserts (Analytical Sales and Services) fitted with stainless-steel stir bars (V&P scientific) in a nitrogen- filled glove box, were dispensed appropriate quantities of the stock solutions in the following order: heterobenzyl chloride (10 µL), aryl iodide (10 µL), Ni pre-catalyst (20 µL), ligand (20 µL), additive (40 µL), and reductant (10 µL). The concentrations of the stock solutions were chosen so that each stock solution was dispensed to give a total volume of ~110 µL in each HTE well. The plate was then sealed with a screwdriver and placed in a zip lock bag inside the glove box. The plate was then removed from the glove box and agitated on a tumble stirrer (V&P Scientific) at the specified temperature for 24 h. After 24 h, the plate was removed from heat and allowed to cool to room temperature before being opened to air. A quench solution of 0.1 M AcOH in MeCN (0.6 mL) was then added to each well and the plate put back onto the tumble stirrer for 10 min. An aliquot (20 µL) was then taken and filtered directly into an HPLC collection block (Analytical Sales and Services) and diluted to 1.0 mM with MeCN. Yields were then determined either as a percentage of product relative to all known species derived from the heterobenzyl chloride or a compound/std ratio. For analysis using internal standard, the quench solution was prepared containing *N*,*N*-dibenzylaniline as a standard equimolar to heterobenzyl chloride. The data was then visualized on Tableau® or in Excel.

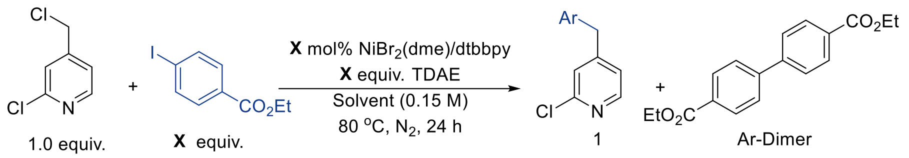

Conditions a-g	1	Ar-Dimer	Substrate
a. 7 mol% $NiBr_2$•dme/dtbbpy, 1.0 equiv Ar-I, 1.2 equiv TDAE, propylene carbonate, 80 °C, 16 h, N_2	93	6	As drawn
b. 7 mol% $NiBr_2$•dme/dtbbpy, **1.2 equiv Ar-I**, 1.4 equiv TDAE, propylene carbonate, 80 °C, 16 h, N_2	80	6	As drawn
c. 7 mol% $NiBr_2$•dme/dtbbpy, 1.2 equiv Ar-I, 1.4 equiv TDAE, **DMA**, 80 °C, 16 h, N_2	70	3	As drawn
d. 7 mol% $NiBr_2$•dme/dtbbpy, 1.2 equiv Ar-I, 1.4 equiv TDAE, **dioxane**, 80 °C, 16 h, N_2	99	13	As drawn
***e.* 5 mol% $NiBr_2$•dme/dtbbpy**, 1.2 equiv Ar-I, 1.4 equiv TDAE, dioxane, 80 °C, 16 h, N_2	99	11	As drawn
f. 5 mol% $NiBr_2$•dme/dtbbpy, 1.2 equiv Ar-I, 1.4 equiv TDAE, dioxane, 80 °C, 16 h, N_2	99*	12	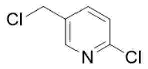
g. 5 mol% $NiBr_2$•dme/dtbbpy, 1.2 equiv Ar-I, 1.4 equiv TDAE, dioxane, 80 °C, 16 h, N_2	99*	12	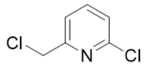

Figure S1. Adaptation of literature conditions for primary heterobenzyl chloride substrates. Reactions were run on a 0.1 mmol scale. Yields were calculated using ^{1}H NMR spectroscopy with mesitylene as an internal standard. *These entries utilize the benzyl chloride shown in the rightmost column.

Discussion: Simple adjustments to the solvent and Ni/ligand loading from literature reported conditions[5] gave optimal yields for all three model primary heterobenzyl chlorides.

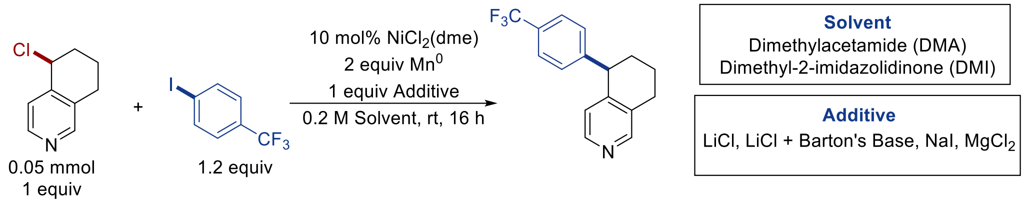

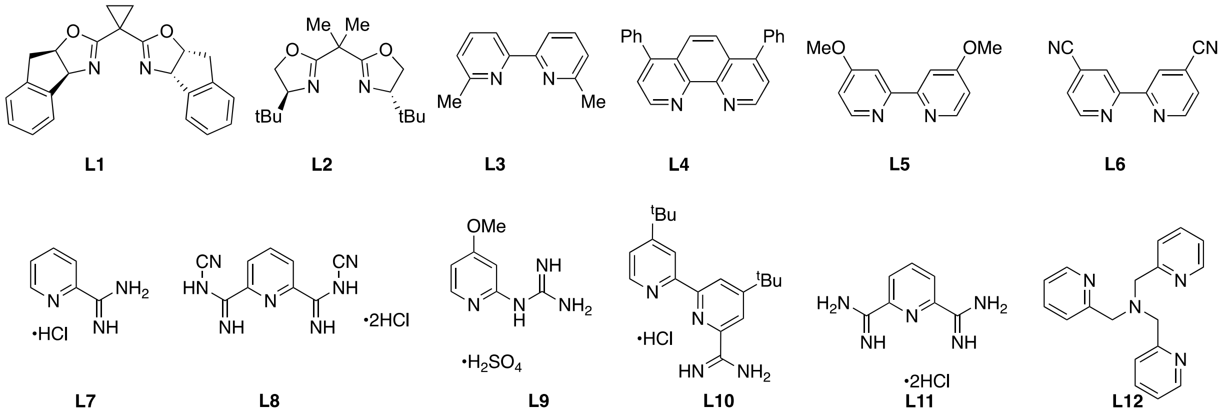

Solvent	Additive	L1	L2	L3	L4	L5	L6	L7	L8	L9	L10	L11	L12
DMA	LiCl	22	0	11	0	0	0	0	0	0	0	0	0
	LiCl+BB	16	0	6	0	0	0	0	0	0	0	0	0
	MgCl2	8	4	8	4	5	0	0	0	4	0	0	0
	NaI	0	0	0	0	0	0	0	0	0	0	0	0
DMI	LiCl	23	0	8	0	0	0	0	0	0	0	0	0
	LiCl+BB	11	0	5	0	0	0	0	0	0	0	0	0
	MgCl2	0	0	9	8	0	0	0	0	5	0	0	0
	NaI	0	0	0	0	0	0	0	0	0	0	0	0

Ligand

Sum of Conversion: 25.09, 40.00, 60.00, 80.00, 100.00

Sum of Calibrated yield: 0 – 100

Figure S2. Initial HTE screening for ligands, additives, and solvents for reductive coupling of the secondary heterobenzyl chloride 5-chloro-5,6,7,8-tetrahydroisoquinoline (**4a**) and 1-iodo-4-(trifluoromethyl)benzene. Reactions were run on a 0.05 mmol scale. Yields are reported as area percent of product relative to all known species derived from **4a** as determined by UV-visible spectroscopy.

Discussion: The results reported in **Figure S2** show the reductive coupling reaction outcome when employing different ligands, additives, and solvents. From this HTE data, LiCl, bisoxazoline ligand (**L1**), and DMA were selected for initial optimization studies as this combination afforded high yields of the desired product **4**. Heterobenzyl chloride reduction and heterobenzyl chloride dimerization, along with unreacted aryl iodide, accounted for the remaining mass balance.

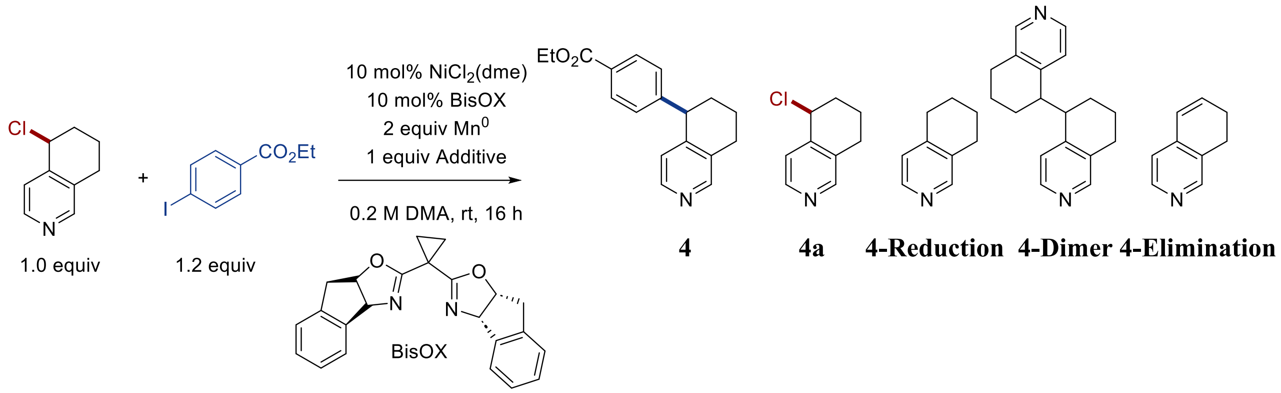

Additive	4	4a	4-Reduction	4-Dimer	4-Elimination
TBACl	8	0	52	40	0
KCl	12	0	35	53	0
LiBr	15	0	60	25	0
TBABr	7	0	42	51	0
KBr	16	0	49	35	0
TBAI	3	0	53	44	0

Figure S3. Additive screening for reductive coupling of 5-chloro-5,6,7,8-tetrahydroisoquinoline (**4a**) and 4-iodoethylbenzoate. Reactions were run on a 0.1 mmol scale. Yields are reported as area percent of product relative to all known species derived from **4a** as determined by UV-visible spectroscopy.

Discussion: The results reported in **Figure S3** show the reductive coupling reaction outcome when employing additives. However, no significant improvements in reactivity were observed when compared to results in **Figure S2.**

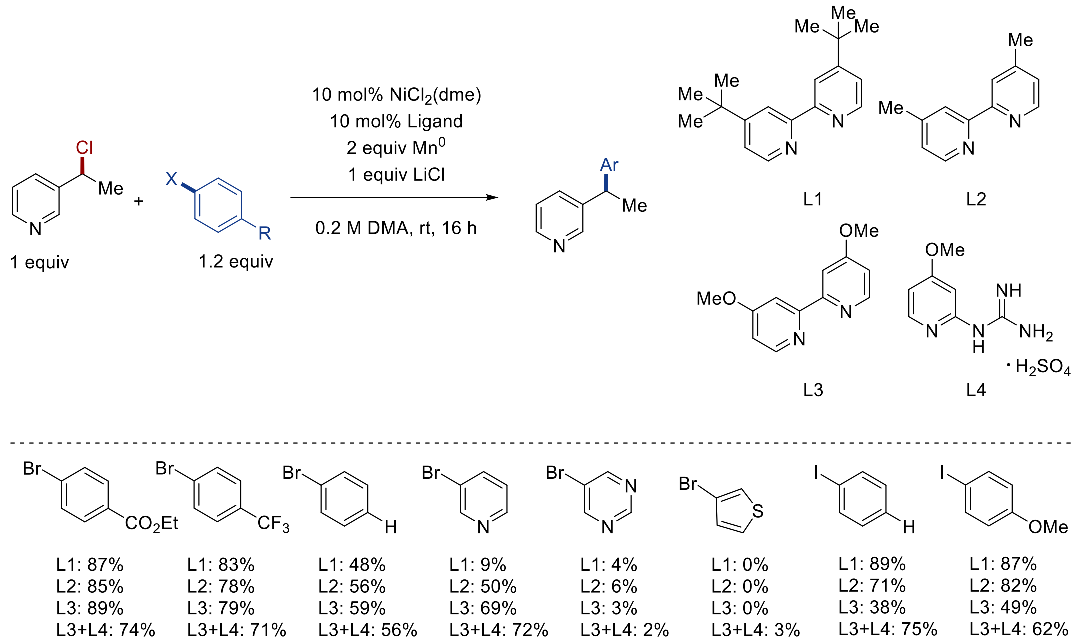

Figure S4. Aryl electrophile screening for reductive coupling of 3-(1-chloroethyl)pyridine (**26a**). Reactions were run on a 0.1 mmol scale. Yields are reported as area percent of product relative to all known species derived from **26a** as determined by UV-visible spectroscopy.

Discussion: The results reported in **Figure S4** show the reductive coupling reaction outcome when employing various aryl electrophiles. A clear electronic trend is visible within the aryl bromides with the yield decreasing as the arenes become more electron rich. This trend is not present with the aryl iodides tested and even electron rich 4-iodoanisole is high yielding.

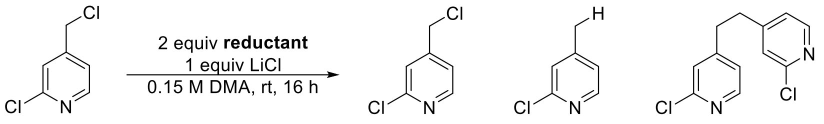

1a **1a** **1-Reduction** **1-Dimer**

Reductant	1a	1-Reduction	1-Dimer
Zn	98	2	0
Mn	97	3	0
TDAE*	99	0	0

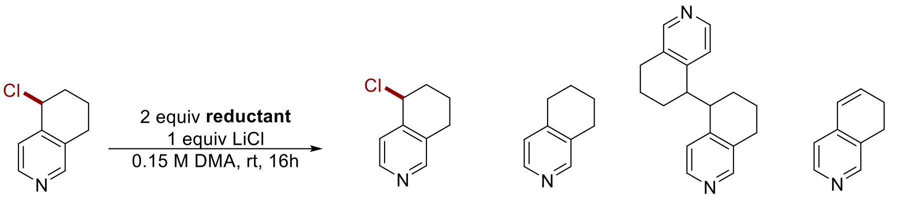

4a **4a** **4-Reduction** **4-Dimer** **4-Elimination**

Reductant	4	4a	4-Reduction	4-Dimer	4-Elimination
Zn	0	29	34	37	0
Mn	0	70	30	0	0
TDAE*	0	99	0	0	0

Figure S5. Evaluation of heterobenzyl chloride consumption by Zn^0, Mn^0, and TDAE in the absence of Ni catalyst. Reactions were run on a 0.1 mmol scale. Yields are reported as area percent of product relative to all known species derived from **4a** as determined by UV-visible spectroscopy. *Reaction included no LiCl.

Discussion: The results reported in **Figure S5** show that Zn^0 and Mn^0 can directly activate and contribute to the consumption of **4a** but do not readily reduce primary substrate **1a**. These data supported further optimization with TDAE as the reductant.

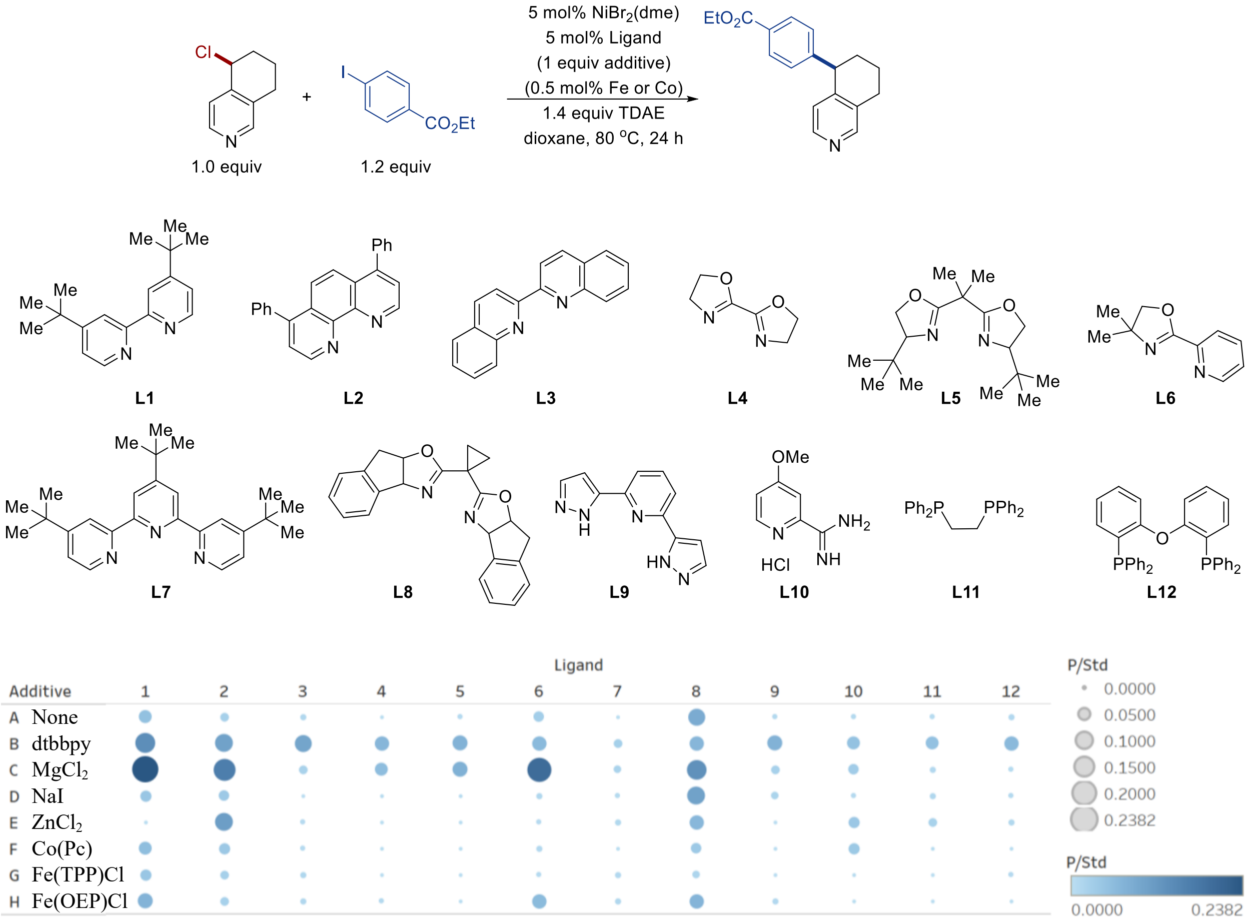

Figure S6. Ligand and additive screen using TDAE as the reductant for reductive coupling of 5-chloro-5,6,7,8-tetrahydroisoquinoline (**4a**) and ethyl 4-iodobenzoate. Reactions were run in a 96 well HTE plate on a 10 µmol scale. Yields are reported as a ratio of the peak area of the product compared to the peak area of dibenzylaniline as an internal standard at 210 nm.

Discussion: The results reported in **Figure S6** show that the best ligand and additive combination to couple 4-substituted secondary heterobenzyl chlorides and aryl iodides is dtbbpy (L1) and $MgCl_2$ (Well C1). We also note that inclusion of 2 equiv dtbbpy gave higher yields than a 1:1 ratio of Ni:dtbbpy (Well B1) for this 4-substituted secondary heterobenzyl chloride substrate.

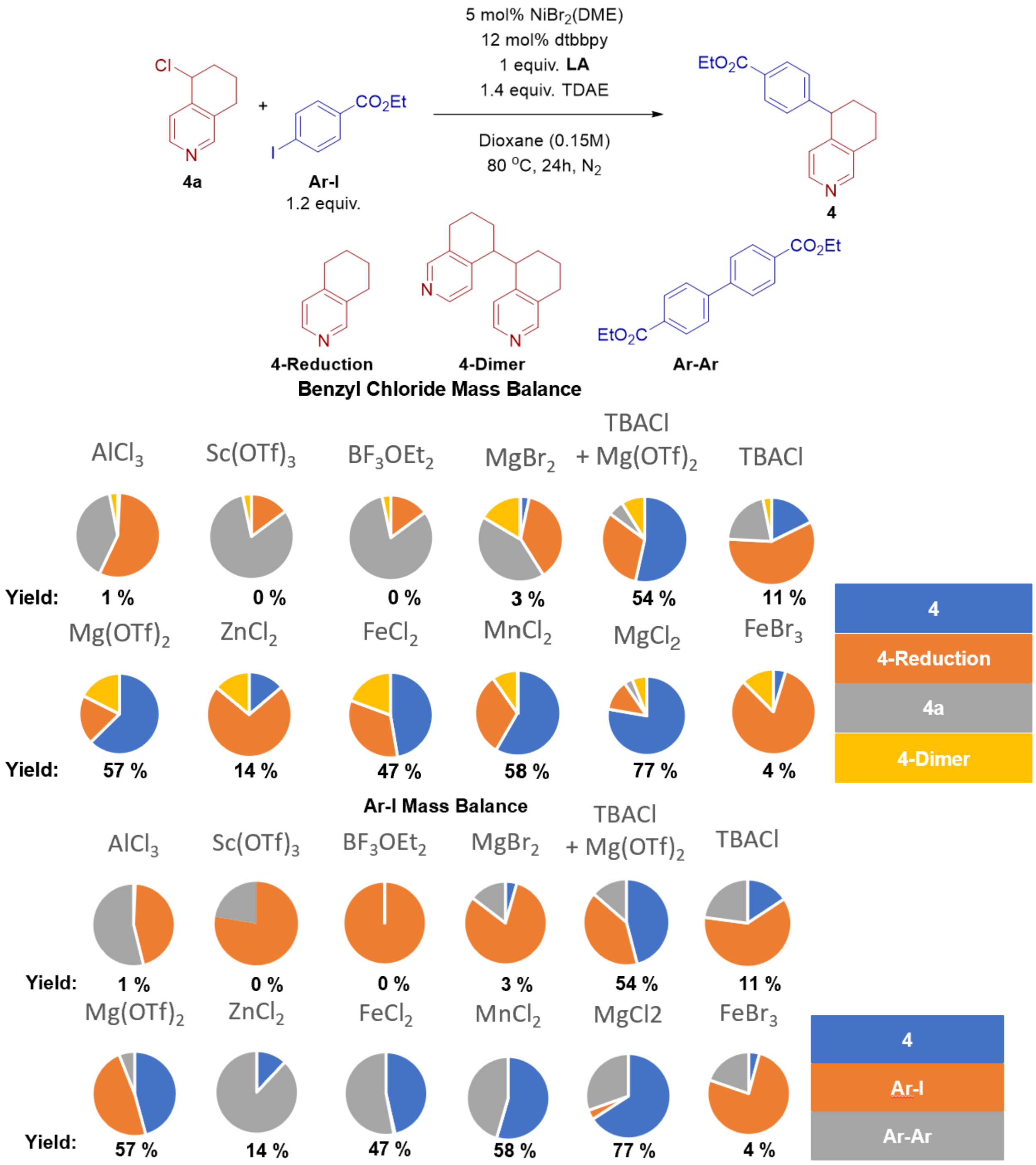

Figure S7. Lewis acid screen using TDAE as the reductant for reductive coupling of 5-chloro-5,6,7,8-tetrahydroisoquinoline (**4a**) and 4-iodoethylbenzoate. Plots show the mass balance with respect to either coupling partner (Top BnCl, Bottom Ar-I). Reactions were run on 0.1 mmol scale. Yields are reported as a ratio of the peak area of the product compared to the peak area of dibenzylaniline as an internal standard at 210 nm. TBACl = Tetrabutylammonium chloride.

Discussion: The results reported in **Figure S7** show the best Lewis acid to be $MgCl_2$ with other notable examples $FeCl_2$ and $MnCl_2$ both of which also improved yield. The inclusion of $ZnCl_2$ led to significant

homodimerization of the Ar-I and reduction of the BnCl. Stronger Lewis acids such as BF_3OEt_2 and $Sc(OTf)_3$ inhibited reactivity leading to poor conversion of both coupling partners.

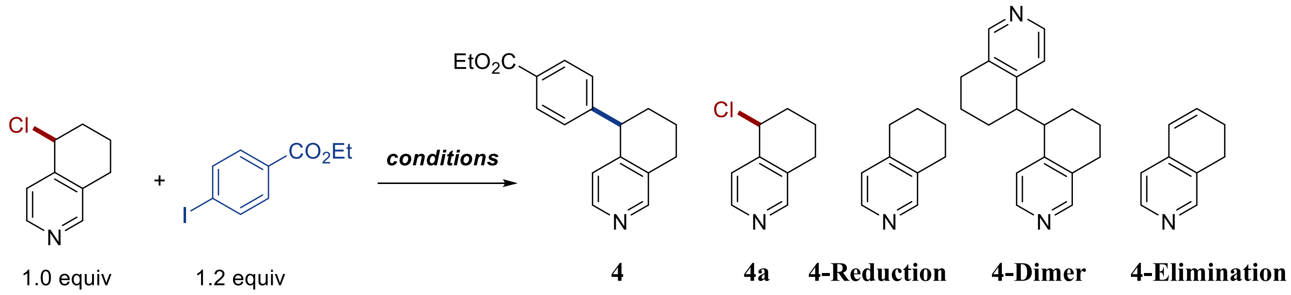

Conditions	4	4a	4-Reduction	4-Dimer	4-Elimination
5 mol% $NiBr_2$•dme<br>12 mol% dtbbpy<br>1.4 eq TDAE<br>1,4-dioxane, 80 ºC, 24 h, N_2	55	0	21	22	4
5 mol% $NiBr_2$•dme/dtbbpy<br>1 equiv $MgCl_2$, 1.4 equiv TDAE<br>1,4-dioxane, 80 ºC, 24 h, N_2	67	0	24	9	0
5 mol% $NiBr_2$•dme<br>12 mol% dtbbpy<br>1 equiv $MgCl_2$, 1.4 equiv TDAE<br>1,4-dioxane, 80 ºC, 24 h, N_2	80	0	16	2	2

Figure S8. Validation of the best conditions identified using HTE (**Figure S5**). Reactions were run on a 0.1 mmol scale. Yields are reported as area percent of product relative to all known species derived from **4a** as determined by UV-visible spectroscopy.

Discussion: The results observed in **Figure S6** were successfully validated showing that for a 4-substituted secondary heterobenzylic chloride, increasing the ligand loading and adding $MgCl_2$ both increase product formation resulting in the highest yield when used together.

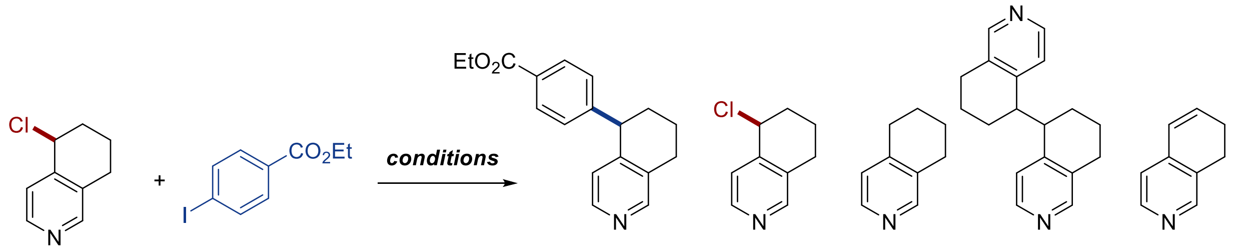

4 **4a** **4-Reduction** **4-Dimer** **4-Elimination**

Conditions A-D	4	4a	4-Reduction	4-Dimer	4-Elimination
A. 5 mol% $NiBr_2$•dme, 5 mol% dtbbpy, 1.4 eq TDAE, 1,4-dioxane, 80 ºC, 24 h, N_2	11	41	38	10	0
B. 5 mol% $NiBr_2$•dme, 12 mol% dtbbpy, 1 equiv $MgCl_2$, 1.4 equiv TDAE, 1,4-dioxane, 80 ºC, 24 h, N_2	80	0	16	2	2
C. 5 mol% $NiBr_2$•dme/dtbbpy, 0.5 mol% Co(Pc), 1.4 equiv TDAE, 1,4-dioxane, 80 ºC, 24 h, N_2	16	0	25	20	38
D. 5 mol% $NiBr_2$•dme/dtbbpy, 0.5 mol% Fe(TPP)Cl, 1.4 equiv TDAE, 1,4-dioxane, 80 ºC, 24 h, N_2	44	0	27	29	0

Figure S9. Evaluation of reductive coupling of 5-chloro-5,6,7,8-tetrahydroisoquinoline (**2a**) and ethyl 4-iodobenzoate under the three optimized conditions reported in the manuscript. Reactions were run on a 0.1 mmol scale. Yields are reported as area percent of product relative to all known species derived from **1a** as determined by UV-visible spectroscopy.

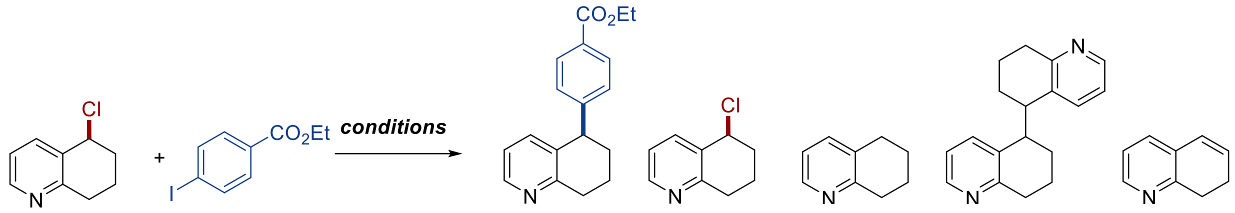

1.0 equiv 1.2 equiv **5** **5a** **5-Reduction** **5-Dimer** **5-Elimination**

Conditions A-D	5	5a	5-Reduction	5-Dimer	5-Elimination
A.<br>5 mol% $NiBr_2$•dme<br>5 mol% dtbbpy<br>1.4 eq TDAE<br>1,4-dioxane, 80 ºC, 24 h, N_2	15	10	47	24	4
B.<br>5 mol% $NiBr_2$•dme<br>12 mol% dtbbpy<br>1 equiv $MgCl_2$, 1.4 equiv TDAE<br>1,4-dioxane, 80 ºC, 24 h, N_2	35	15	13	52	0
C.<br>5 mol% $NiBr_2$•dme/dtbbpy<br>0.5 mol% Co(Pc), 1.4 equiv TDAE<br>1,4-dioxane, 80 ºC, 24 h, N_2	54	0	4	17	6
D.<br>5 mol% $NiBr_2$•dme/dtbbpy<br>0.5 mol% Fe(TPP)Cl, 1.4 equiv TDAE<br>1,4-dioxane, 80 ºC, 24 h, N_2	25	0	14	61	0

Figure S10. Evaluation of reductive coupling of 5-chloro-5,6,7,8-tetrahydroquinoline (**5a**) and ethyl 4-iodobenzoate under the three optimized conditions reported in the manuscript. Reactions were run on a 0.1 mmol scale. Yields are reported as area percent of product relative to all known species derived from **5a** as determined by UV-visible spectroscopy.

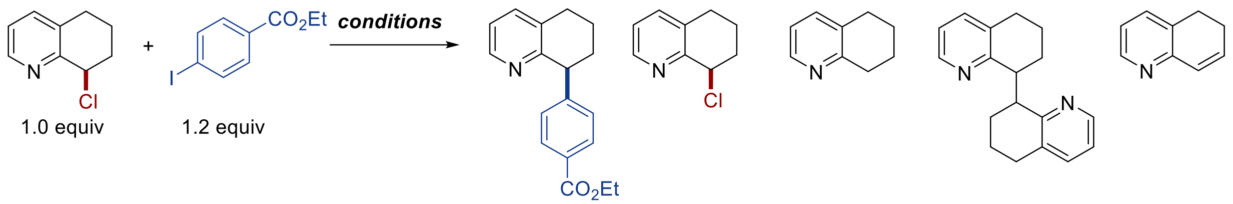

6 **6a** **6-Reduction** **6-Dimer** **6-Elimination**

Conditions A-D	6	6a	6-Reduction	6-Dimer	6-Elimination
A.<br>5 mol% $NiBr_2$•dme<br>5 mol% dtbbpy<br>1.4 eq TDAE<br>1,4-dioxane, 80 ºC, 24 h, N_2	17	43	30	10	0
B.<br>5 mol% $NiBr_2$•dme<br>12 mol% dtbbpy<br>1 equiv $MgCl_2$, 1.4 equiv TDAE<br>1,4-dioxane, 80 ºC, 24 h, N_2	72	10	13	5	0
C.<br>5 mol% $NiBr_2$•dme/dtbbpy<br>0.5 mol% Co(Pc), 1.4 equiv TDAE<br>1,4-dioxane, 80 ºC, 24 h, N_2	67	0	25	8	0
D.<br>5 mol% $NiBr_2$•dme/dtbbpy<br>0.5 mol% Fe(TPP)Cl, 1.4 equiv TDAE<br>1,4-dioxane, 80 ºC, 24 h, N_2	83	0	6	11	0

Figure S11. Evaluation of reductive coupling of 8-chloro-5,6,7,8-tetrahydroquinoline (**6a**) and ethyl 4-iodobenzoate under the three optimized conditions reported in the manuscript. Reactions were run on a 0.1 mmol scale. Yields are reported as area percent of product relative to all known species derived from **6a** as determined by UV-visible spectroscopy.

5. Cyclic Voltammogram Experiments

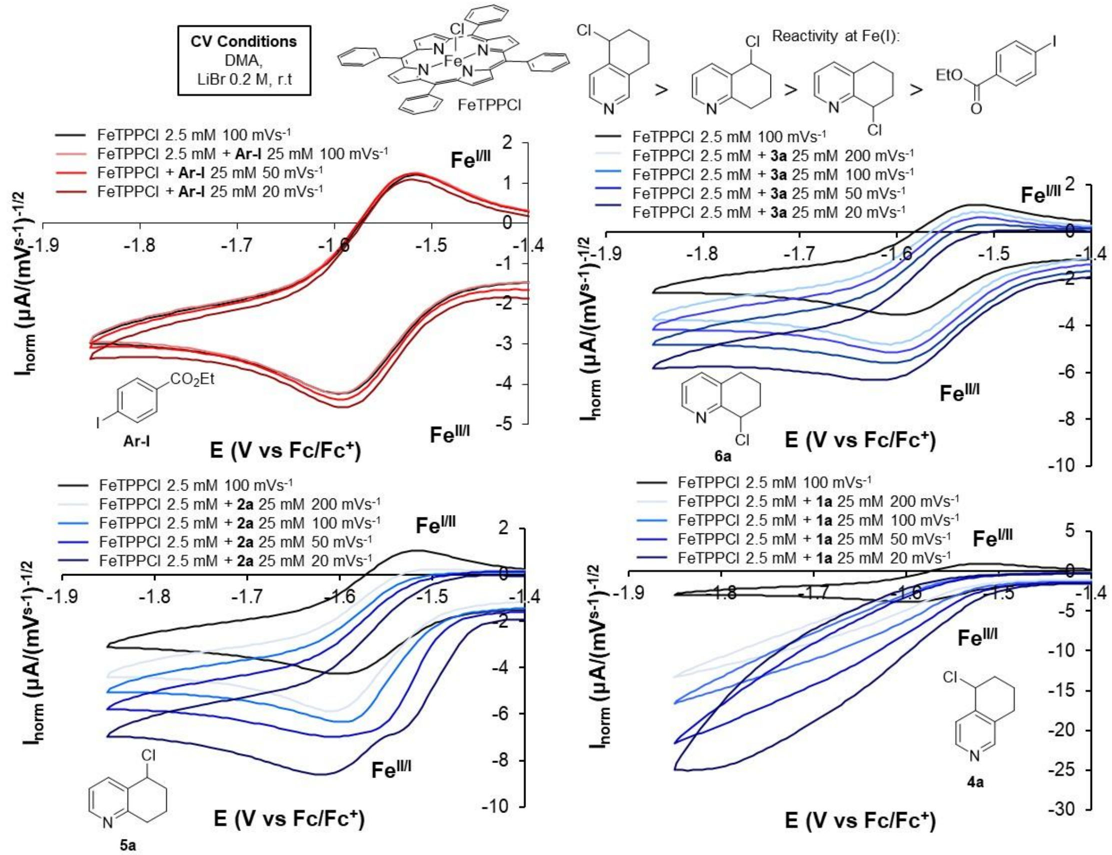

Figure S12. Cyclic voltammograms of model secondary heterobenzylic chlorides in the presence of 10 mol% Fe(TPP)Cl at varying scan rates. The CV experiments were carried out in a three-electrode cell configuration with a glassy carbon (GC) working electrode (3 mm diameter), and a platinum wire counter electrode (~1.0 cm, spiral wire).

Discussion: The results reported in **Figure S12** show that Fe(TPP)Cl, after reduction, is competent for activation of the heterobenzyl chlorides (evidenced by the current enhancement) but does not activate the Ar-I (no change in the CV upon addition of substrate).

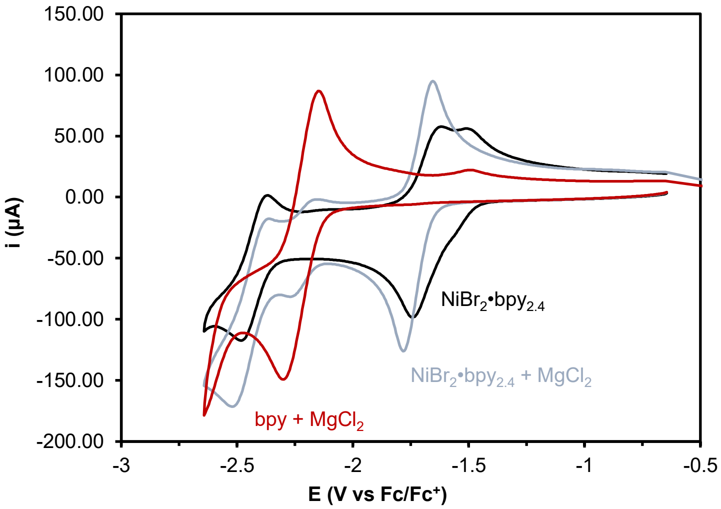

Figure S13. Cyclic voltammograms of $NiBr_2$(dme) with and without $MgCl_2$. All traces use a scan rate of 100 mV/s and DMA as the solvent with TBA•PF_6 (0.1 M) as the electrolyte. Black trace is 5 mM $NiBr_2$(dme) and 12 mM bpy (bpy = 2,2'-bipyridine). Gray trace is 5 mM $NiBr_2$(dme), 12 mM bpy, and 20 mM $MgCl_2$. Red trace is 12 mM bpy and 20 mM $MgCl_2$. We note that bpy was used for these studies instead of dtbbpy because of more clear redox features compared to dtbbpy.

Discussion: The results reported in **Figure S13** show that the Ni speciation undergoes a significant change upon the addition of $MgCl_2$. It appears that $MgCl_2$ causes the formation of a new Ni species that undergoes reduction at a more reducing potential. Furthermore, a signal that can be attributed to a bpy-$MgCl_2$ adduct is observed in both the red and gray traces.

6. Procedure for Time Course Reaction Monitoring

General Procedure for time course reactions: On the benchtop, an oven-dried 50-mL Schlenk tube was charged with heterobenzylic chloride (0.5 mmol, 1 equiv), ethyl 4-iodobenzoate (0.6 mmol, 1.2 equiv), 1,3,5-trimethoxybenzene (as internal standard, 0.5 mmol, 1 equiv) and a cross-shaped stir bar. $NiBr_2$(dme) (0.025 mmol, 5 mol%) and dtbbpy (0.025 mmol, 5 mol%) were weighed into a secondary 2-dram vial with a cross-shaped stir bar. Both the vial and Schlenk tube were sparged with N_2 then transferred into a purging glovebox under N_2. In the glovebox, 1,4-dioxane (6.5 mL) was added to the vial containing $NiBr_2$(dme) and dtbbpy and stirred for 10 min to from a blue-green catalyst stock solution. To the Schlenk tube containing heterobenzylic chloride, aryl iodide, and the cross-shaped stir bar was added either $MgCl_2$ (0.5 mmol, 1 equiv), Co(Pc) (0.0025 mmol, 0.5 mol%), Fe(TPP)Cl (0.0025 mmol, 0.5 mol%), or no additive, the catalyst stock solution (3.5 mL), and TDAE (0.7 mmol, 1.4 equiv). The Schlenk tube was then sealed with a rubber septum, removed from the glovebox, and set to stir at 750 rpm in an oil bath at 80 ºC. Under a positive pressure of nitrogen, at time intervals of 30 min or 1 h, aliquots (0.1 mL) were removed via syringe and filtered through Celite into an NMR tube with $CDCl_3$ for analysis by ^{1}H NMR spectroscopy.

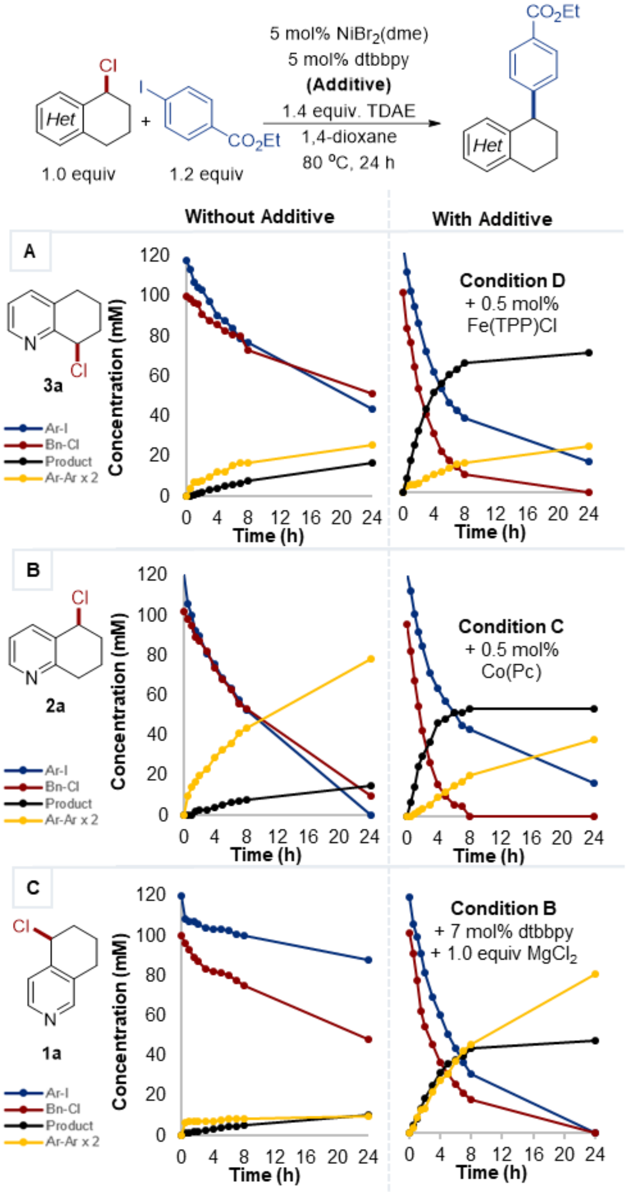

Figure S14. Time course studies with and without key additives.

7. HTE Assessment of Heteroaryl Iodides

General Procedure: On the bench top, to a 6-dram vial fitted with a cross-shaped stir bar were added $NiBr_2$•dme (5.8 mg, 0.019 mmol), dtbbpy (2.7 mg, 0.01 mmol), and the appropriate additive [$MgCl_2$ (11.9 mg, 0.13 mmol), Co(Pc) (1.1 mg, 0.002 mmol), or Fe(TPP)Cl (1.4 mg, 0.002 mmol)]. To separate individual 6-dram vials were added heterobenzylic chloride (0.38 mmol) and aryl iodides (0.07 mmol). These vials were then transferred into a nitrogen-filled glove box and 1,4-dioxane was added to give a solution 5 times the concentration needed for the reaction (this allows 20 μL of each solution to be added to give a 100 μL reaction volume). The catalyst stock solution was stirred for 15 min. To a 96-well optimization block (Analytical Sales and Services) with 1-mL glass vial inserts (Analytical Sales and Services) fitted with stainless-steel stir bars (V&P scientific) in a nitrogen-filled glove box, were dispensed 20 μL of the stock solutions in the following order: heterobenzyl chloride, aryl iodide, catalyst stock solution. The reductant was then directly added neat to each well (3.2 μL). The plate was then sealed with a screwdriver and agitated on a tumble stirrer (V&P Scientific) at 80 ºC for 16 h. The block was then diluted to 50 mM with MeCN (0.1 mL) containing dibenzylaniline equimolar to the theoretical yield of cross-coupled product (0.01 mmol) and an aliquot (2 μL) was filtered and taken into an HPLC collection block (Analytical Sales and Services). The HPLC collection block was then further diluted with MeCN to 1 mM and analyzed using UPLC-MS (Waters-Acquity) analysis. Yields were determined as percent of product relative internal standard. Data was then visualized on excel or Tableau®.

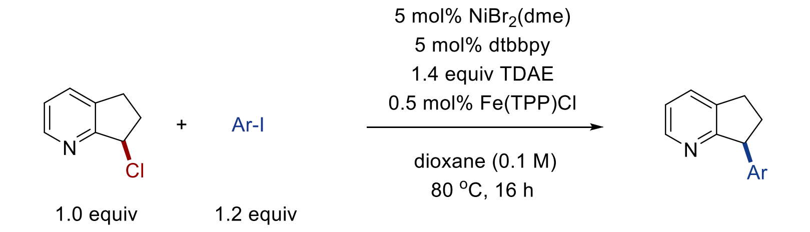

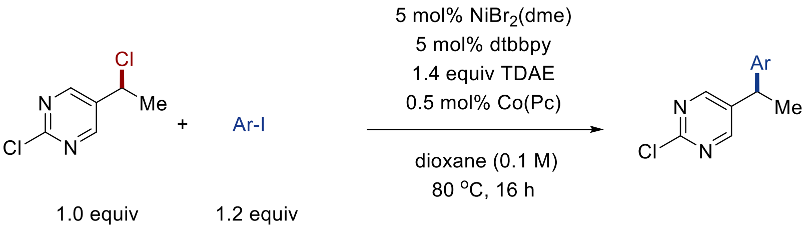

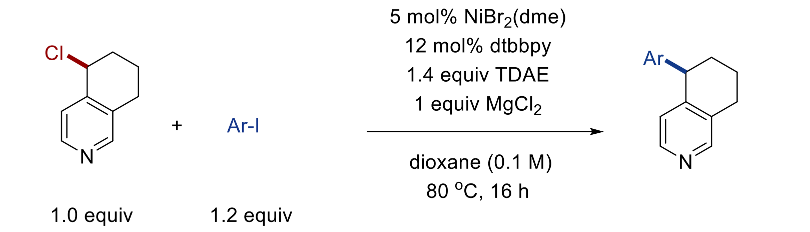

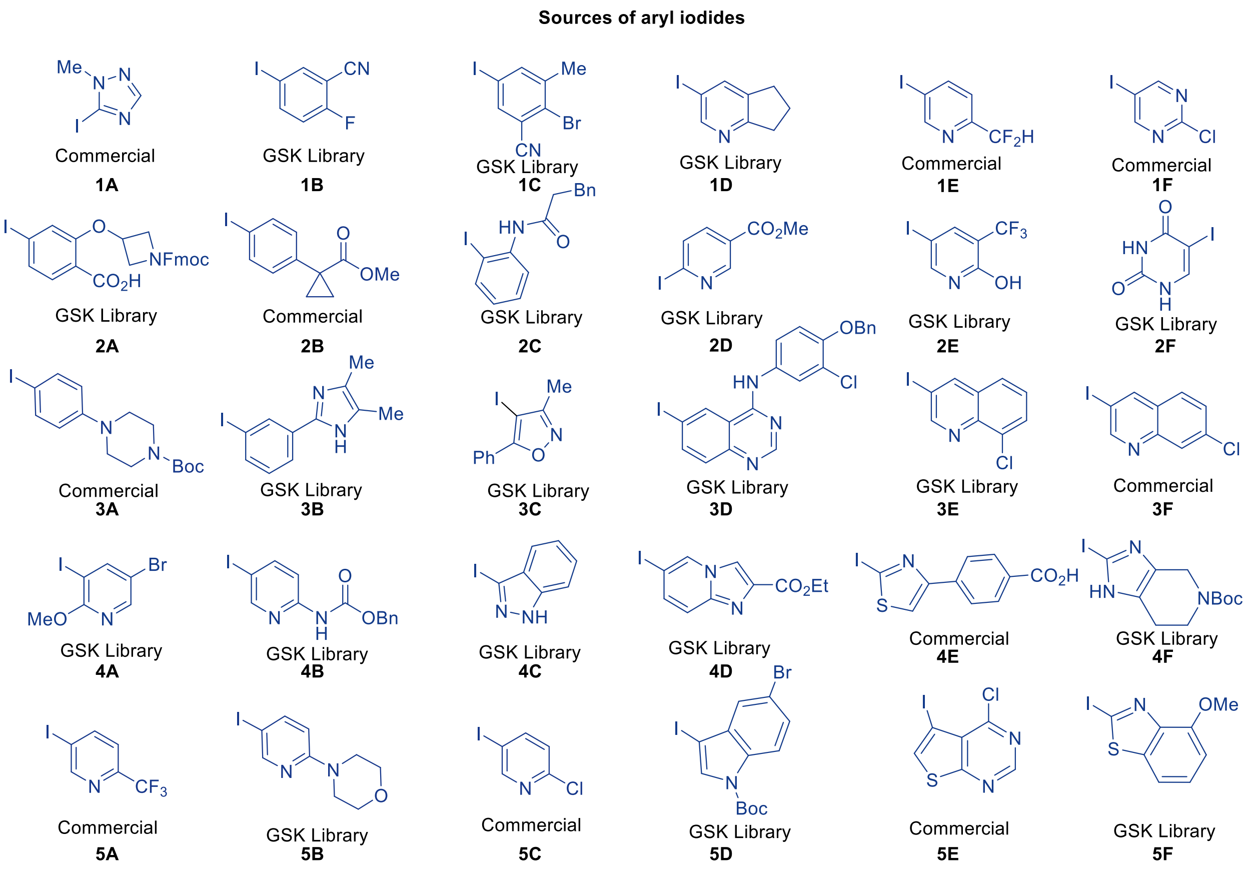

Figure S15. Structures of the aryl iodides screened for cross-coupling with secondary heterobenzyl chlorides and the sources of each aryl iodide.

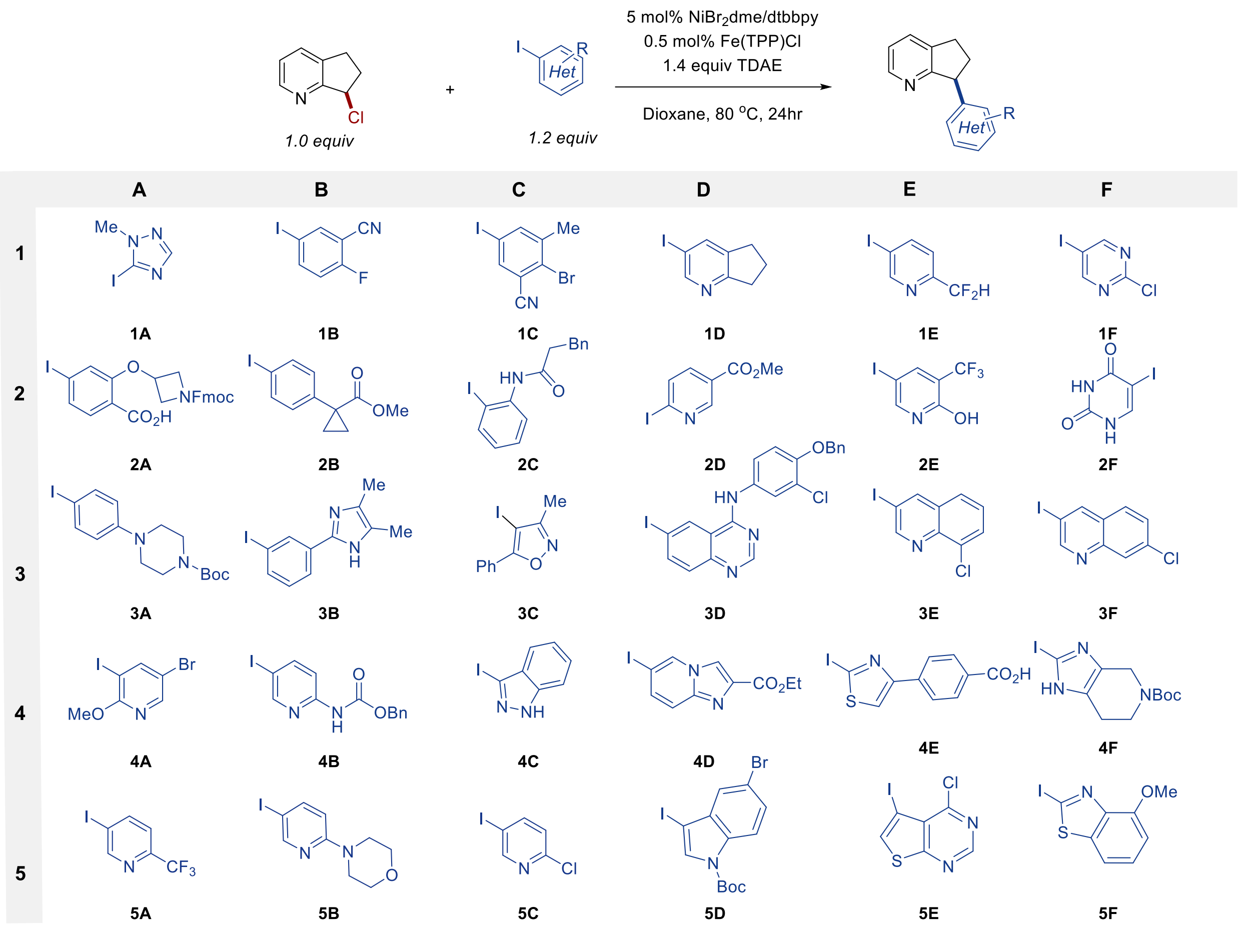

	A	B	C	D	E	F
1	0	95	88	95	95	99
2	0	99	0	0	57	83
3	99	0	92	92	79	71
4	88	63	73	97	95	0
5	60	63	75	20	46	84

Pdt/IS Ratio (scale: 0–1)

Figure S16. Evaluation of aryl iodide coupling using 7-chloro-6,7-dihydro-5*H*-cyclopenta[*b*]pyridine (**24a**) and various aryl iodides. Reactions were run on a 0.03 mmol scale. Yields were determined as percent of product relative internal standard as determined by UV-visible spectroscopy.

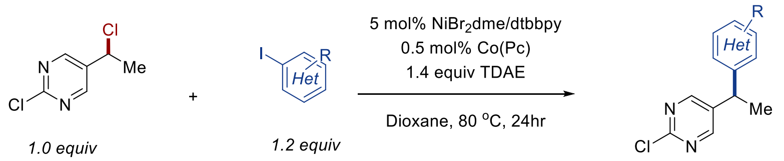

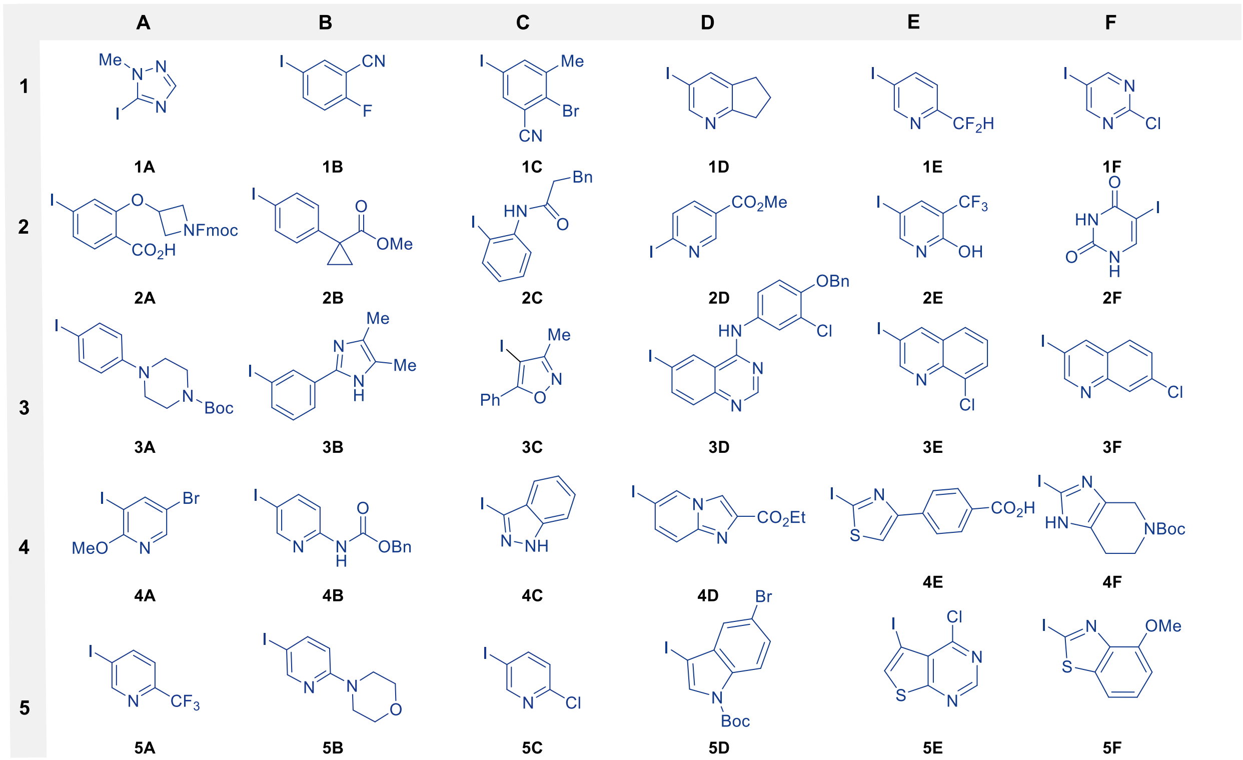

	A	B	C	D	E	F
1	0	0	0	92	75	4
2	30	0	0	68	74	0
3	64	17	93	93	94	0
4	40	88	89	0	94	0
5	32	90	0	93	32	18

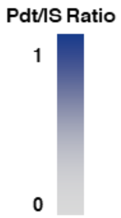

Figure S17. Evaluation of aryl iodide coupling using 2-chloro-5-(1-chloroethyl)pyrimidine (**30a**) and various aryl iodides. Reactions were run on a 0.03 mmol scale. Yields were determined as percent of product relative internal standard as determined by UV-visible spectroscopy.

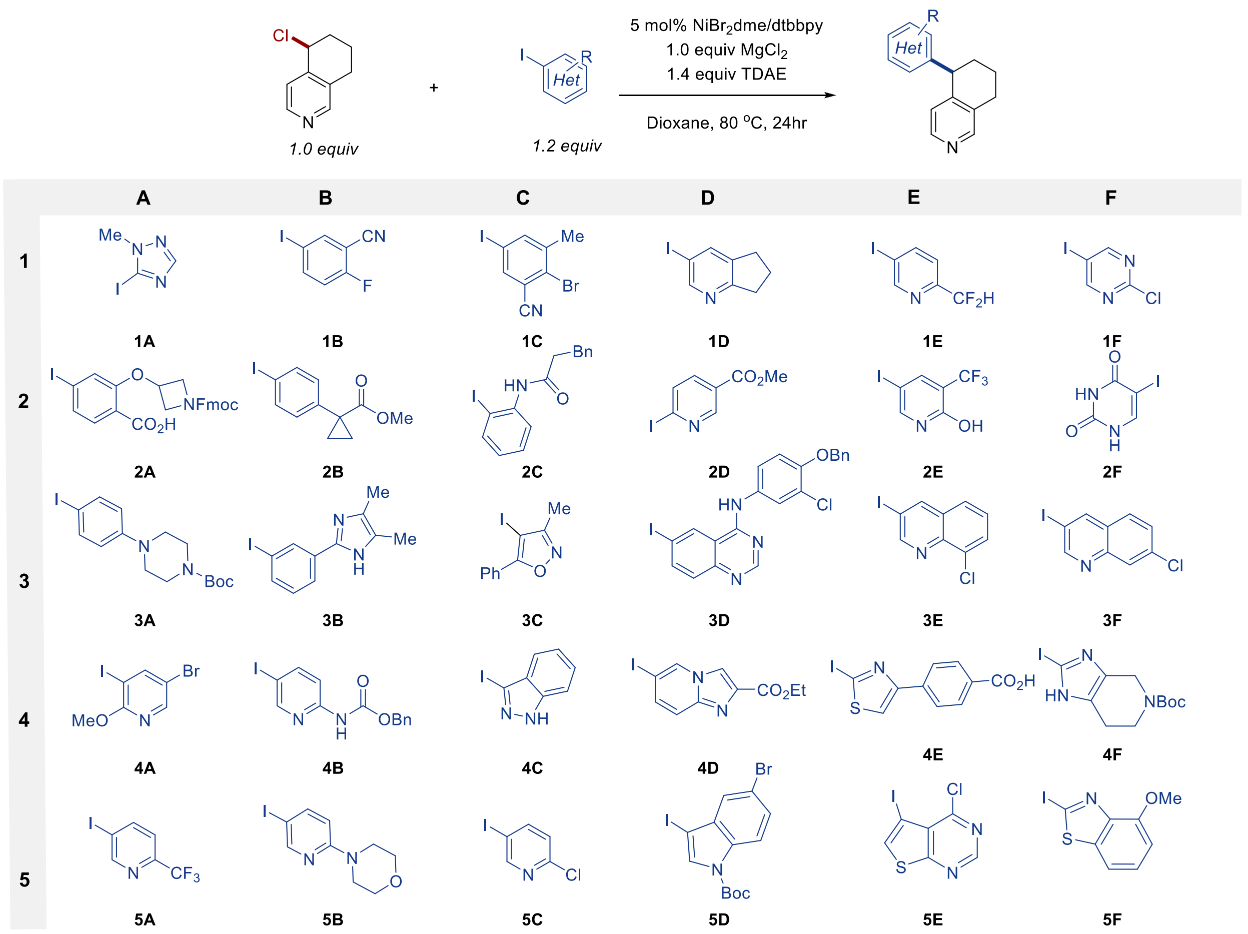

	A	B	C	D	E	F
1	34	75	0	0	31	0
2	0	46	42	0	14	0
3	62	0	0	0	14	83
4	12	0	0	10	0	0
5	46	0	40	78	0	0

Pdt/IS Ratio (scale: 0 to 1)

Figure S18. Evaluation of aryl iodide coupling using 5-chloro-5,6,7,8-tetrahydroisoquinoline (**4a**) and various aryl iodides. Reactions were run on a 0.03 mmol scale. Yields were determined as percent of product relative internal standard as determined by UV-visible spectroscopy.

8. Product Synthesis and Characterization

(1) ethyl 4-((2-chloropyridin-4-yl)methyl)benzoate

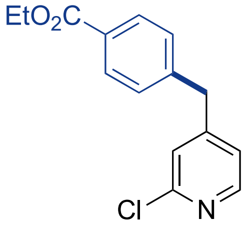

Reaction conducted following **Procedure A** using 2-chloro-4-(chloromethyl)pyridine (116.0 mg, 0.5 mmol, 1 equiv). Deviation from procedure is the exclusion of Barton's base. Following workup, the obtained residue was purified by column chromatography on silica gel eluting with a solvent mixture of ethyl acetate:hexanes (25:75 (v:v)) to afford 124.8 mg (90% yield) of **1** as a yellow oil.

^{1}H NMR (500 MHz, $CDCl_3$) δ 8.28 (d, *J* = 5.1 Hz, 1H), 8.01 (d, *J* = 8.3 Hz, 2H), 7.24 (d, *J* = 8.3 Hz, 2H), 7.12 (s, 1H), 7.01 (d, *J* = 5.3 Hz, 1H), 4.38 (q, *J* = 7.1 Hz, 2H), 4.01 (s, 2H), 1.39 (t, *J* = 7.1 Hz, 3H).
^{13}C NMR (126 MHz, $CDCl_3$) δ 166.4, 152.6, 152.1, 149.9, 143.1, 130.3, 129.6, 129.2, 124.6, 123.0, 61.2, 41.0, 14.5.
HRMS (ESI) m/z: $[M+H]^+$ Calcd for $C_{15}H_{15}ClNO_2$, 276.0786; Found 276.0787

(2) ethyl 4-((6-chloropyridin-3-yl)methyl)benzoate

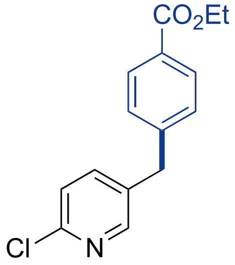

Reaction conducted following **Procedure A** using 2-chloro-5-(chloromethyl)pyridine (80.5 mg, 0.5 mmol, 1 equiv). Deviation from procedure is the exclusion of Barton's base. Following workup, the obtained residue was purified by column chromatography on silica gel eluting with a solvent mixture of ethyl acetate:hexanes (25:75 (v:v)) to afford 126.5 mg (92% yield) of **2** as a yellow oil.

^{1}H NMR (500 MHz, $CDCl_3$) δ 8.27 (d, *J* = 2.4 Hz, 1H), 7.99 (d, *J* = 7.9 Hz, 2H), 7.40 (dd, *J* = 8.2, 2.5 Hz, 1H), 7.23 (m, 3H), 4.37 (q, *J* = 7.1 Hz, 2H), 4.01 (s, 2H), 1.38 (t, *J* = 7.1 Hz, 3H).
^{13}C NMR (126 MHz, $CDCl_3$) δ 166.4, 150.0, 149.9, 144.4, 139.3, 134.7, 130.4, 129.3, 128.9, 124.4, 61.1, 38.3, 14.5.
HRMS (ESI) m/z: $[M+H]^+$ Calcd for $C_{15}H_{15}ClNO_2$, 276.0786; Found 276.0784

(3) ethyl 4-((6-chloropyridin-2-yl)methyl)benzoate

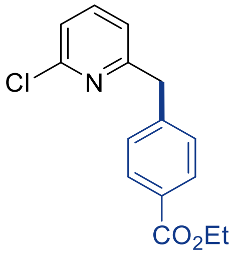

Reaction conducted following **Procedure A** using 2-chloro-6-(chloromethyl)pyridine (80.5 mg, 0.5 mmol, 1 equiv). Deviation from procedure is the exclusion of Barton's base. Following workup, the obtained residue was purified by column chromatography on silica gel eluting with a solvent mixture of ethyl acetate:hexanes (25:75 (v:v)) to afford 122.7 mg (89% yield) of **3** as a yellow oil.

^{1}H NMR (500 MHz, $CDCl_3$) δ 7.98 (dt, J = 8.2, 1.9 Hz, 2H), 7.54 (t, J = 7.7 Hz, 1H), 7.33 (d, J = 8.3 Hz, 2H), 7.18 (d, J = 7.9 Hz, 1H), 6.98 (d, J = 7.6 Hz, 1H), 4.36 (q, J = 7.1 Hz, 2H), 4.18 (s, 2H), 1.38 (t, J = 7.1 Hz, 3H).
^{13}C NMR (126 MHz, $CDCl_3$) δ 166.6, 161.3, 151.1, 143.9, 139.3, 130.1, 129.3, 129.2, 122.2, 121.7, 61.0, 44.3, 14.5.

(4) Ethyl 4-(5,6,7,8-tetrahydroisoquinolin-5-yl)benzoate

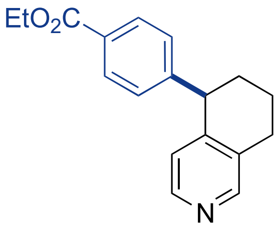

Reaction conducted following **Procedure B** using 5-chloro-5,6,7,8-tetrahydroisoquinoline (83.5 mg, 0.5 mmol, 1equiv). Following workup, the obtained residue was purified by column chromatography on silica gel eluting with a solvent mixture of ethyl acetate:hexanes (70:30 (v:v)) to afford 115.3 mg (82% yield) of **4** as a clear oil.

^{1}H NMR (500 MHz, $CDCl_3$) δ 8.39 (s, 1H), 8.20 (d, J = 5.1 Hz, 1H), 7.96 (d, J = 8.3 Hz, 2H), 7.13 (d, J = 8.3 Hz, 2H), 6.66 (d, J = 5.1 Hz, 1H), 4.35 (q, J = 7.1 Hz, 2H), 4.09 (td, J = 6.6, 1.6 Hz, 1H), 2.85 (m, 2H), 2.27 – 2.09 (m, 1H), 2.00 – 1.90 (m, 1H), 1.90 – 1.69 (m, 2H), 1.36 (t, J = 7.1 Hz, 3H).
^{13}C NMR (126 MHz, $CDCl_3$) δ 166.5, 150.7, 147.5, 147.0, 133.2, 129.9, 129.0, 128.8, 124.4, 61.0, 45.3, 32.5, 26.6, 20.9, 14.4.
HRMS (ESI) m/z: $[M+H]^+$ Calcd for $C_{18}H_{20}NO_2$, 282.1489; Found 282.1487

(5) ethyl 4-(5,6,7,8-tetrahydroquinolin-5-yl)benzoate

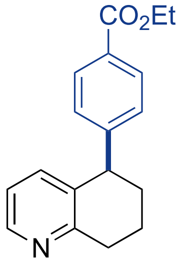

Reaction conducted following **Procedure C** using 5-chloro-5,6,7,8-tetrahydroquinoline (83.5 mg, 0.5 mmol, 1 equiv). Following workup, the obtained residue was purified by column chromatography on silica gel eluting with a solvent mixture of ethyl acetate:hexanes (60:75 (v:v)) to afford 45.0 mg (32% yield) of **5** as a clear oil.

^{1}H NMR (500 MHz, $CDCl_3$) δ 8.42 – 8.41 (d, *J* = 4.7 Hz, 1H), 7.98 (d, *J* = 8.3 Hz, 2H), 7.16 (d, *J* = 8.2 Hz, 2H), 7.13 – 7.07 (m, 1H), 7.00 (m, 1H), 4.37 (q, *J* = 7.1 Hz, 2H), 4.18 (t, *J* = 7.0 Hz, 1H), 3.15 – 2.99 (m, 2H), 2.29 – 2.14 (m, 1H), 2.10 – 1.97 (m, 1H), 1.88 (m, 2H), 1.38 (t, *J* = 7.1 Hz, 3H).
^{13}C NMR (126 MHz, $CDCl_3$) δ 166.6, 157.6, 151.4, 147.5, 138.1, 134.5, 130.0, 129.0, 128.9, 121.4, 61.1, 45.5, 32.7, 21.1, 14.5.
HRMS (ESI) m/z: $[M+H]^+$ Calcd for $C_{18}H_{20}NO_2$, 282.1489; Found 282.1484

(6) ethyl-4-(5,6,7,8-tetrahydroquinolin-8-yl)benzoate

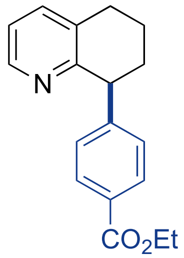

Reaction conducted following **Procedure D** using 8-chloro-5,6,7,8-tetrahydroquinoline pyridine (83.5 mg, 0.5 mmol, 1 equiv). Following workup, the obtained residue was purified by column chromatography on silica gel eluting with a solvent mixture of ethyl acetate:hexanes (30:70 (v:v)) to afford 106.9 mg (76% yield) of **6** as a clear oil.

^{1}H NMR (500 MHz, $CDCl_3$) δ 8.38 (d, *J* = 3.6 Hz, 1H), 7.94 (d, *J* = 8.3 Hz, 2H), 7.46 (d, *J* = 7.6 Hz, 1H), 7.16 – 7.00 (m, 3H), 4.39 – 4.30 (m, 3H), 2.98 – 2.76 (m, 2H), 2.33 – 2.19 (m, 1H), 1.97 (m, 1H), 1.90 – 1.81 (m, 1H), 1.81 – 1.71 (m, 1H), 1.36 (t, *J* = 7.1 Hz, 3H).
^{13}C NMR (126 MHz, $CDCl_3$) δ 166.8, 157.9, 151.9, 147.7, 137.2, 133.3, 129.8, 128.8, 128.4, 121.7, 60.8, 48.0, 33.0, 29.1, 19.8, 14.5.
HRMS (ESI) m/z: $[M+H]^+$ Calcd for $C_{18}H_{20}NO_2$, 282.1489; Found 282.1486

(7) ethyl 4-((2,6-dimethylpyridin-4-yl)methyl)benzoate

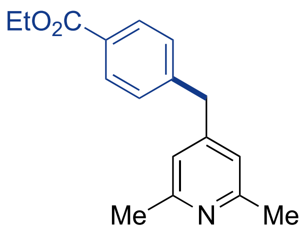

Reaction conducted following **Procedure A** using 4-(chloromethyl)-2,6-dimethylpyridine (77.5 mg, 0.5 mmol, 1 equiv). Deviation from procedure is the exclusion of Barton's base. Following workup, the obtained residue was purified by column chromatography on silica gel eluting with a solvent mixture of ethyl acetate:hexanes (25:75 (v:v)) to afford 119.8 mg (89% yield) of **7** as a clear oil.

^{1}H NMR (500 MHz, $CDCl_3$) δ 7.98 (d, J = 8.2 Hz, 2H), 7.23 (d, J = 8.3 Hz, 2H), 6.76 (s, 2H), 4.37 (q, J = 7.1 Hz, 2H), 3.92 (s, 2H), 2.47 (s, 6H), 1.38 (t, J = 7.1 Hz, 3H).
^{13}C NMR (126 MHz, $CDCl_3$) δ 166.6, 158.1, 149.7, 144.6, 130.1, 129.2, 129.1, 120.9, 61.1, 41.3, 24.5, 14.5.
HRMS (ESI) m/z: $[M+H]^+$ Calcd for $C_{17}H_{20}NO_2$, 270.1489; Found 270.1489

(8) ethyl 4-(pyridin-2-ylmethyl)benzoate

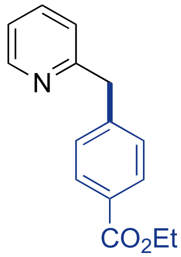

Reaction conducted following **Procedure A** using 2-(chloromethyl)pyridine hydrochloride (82 mg, 0.5 mmol, 1 equiv). Following workup, the obtained residue was purified by column chromatography on silica gel eluting with a solvent mixture of ethyl acetate:hexanes (25:75 (v:v)) to afford 144.7 mg (60% yield) of **8** as a clear oil.

^{1}H NMR: (500 MHz, $CDCl_3$) δ 8.55 (d, J = 4.2 Hz, 1H), 7.98 (d, J = 8.3 Hz, 2H), 7.59 (td, J = 7.6, 1.7 Hz, 1H), 7.34 – 7.30 (m, 2H), 7.17 – 7.06 (m, 2H), 4.35 (q, J = 7.1 Hz, 2H), 4.21 (s, 2H), 1.37 (t, J = 7.1 Hz, 3H).
^{13}C NMR: (126 MHz, $CDCl_3$) δ 166.5, 160.1, 149.5, 144.7, 136.7, 129.9, 129.1, 128.8, 123.2, 121.5, 60.8, 44.6, 14.3.
HRMS (ESI) m/z: $[M+H]^+$ Calcd for $C_{15}H_{16}NO_2$, 242.1176; Found 242.1176

(9) ethyl 4-((5-methylpyridin-2-yl)methyl)benzoate

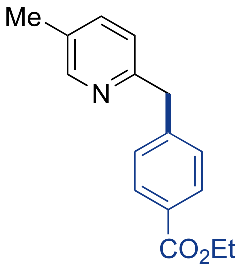

Reaction conducted following **Procedure A** using 2-(chloromethyl)-5-methylpyridine (70.5 mg, 0.5 mmol, 1 equiv). Deviation from procedure is the exclusion of Barton's base. Following workup, the obtained residue was purified by column chromatography on silica gel eluting with a solvent mixture of ethyl acetate:hexanes (25:75 (v:v)) to afford 93.1 mg (73% yield) of **9** as a clear oil.

^{1}H NMR (500 MHz, $CDCl_3$) δ 8.38 (q, *J* = 1.1 Hz, 1H), 7.99 – 7.95 (m, 2H), 7.44 – 7.37 (m, 1H), 7.36 – 7.30 (m, 2H), 7.00 (d, *J* = 7.7 Hz, 1H), 4.35 (q, *J* = 7.1 Hz, 2H), 4.16 (s, 2H), 2.29 (s, 3H), 1.37 (t, *J* = 7.1 Hz, 3H).
^{13}C NMR (126 MHz, $CDCl_3$) δ 166.7, 157.3, 150.0, 145.2, 137.4, 131.0, 130.0, 128.8, 122.8, 61.0, 44.3, 18.2, 14.5.
HRMS (ESI) m/z: $[M+H]^+$ Calcd for $C_{16}H_{18}NO_2$, 256.1332; Found 256.1330

(10) ethyl 4-((3-fluoropyridin-2-yl)methyl)benzoate

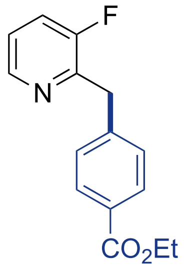

Reaction conducted following **Procedure A** using 2-(chloromethyl)-3-fluoropyridine (72.5 mg, 0.5 mmol, 1 equiv). Deviation from procedure is the exclusion of Barton's base. Following workup, the obtained residue was purified by column chromatography on silica gel eluting with a solvent mixture of ethyl acetate:hexanes (25:75 (v:v)) to afford 89.5 mg (69% yield) of **10** as a clear oil.

^{1}H NMR (500 MHz, $CDCl_3$) δ 8.36 (d, *J* = 4.6, 1H), 8.03 – 7.90 (m, 2H), 7.42 – 7.32 (m, 3H), 7.18 (m, 1H), 4.34 (q, *J* = 7.1 Hz, 2H), 4.25 (d, *J* = 2.6 Hz, 2H), 1.36 (t, *J* = 7.1 Hz, 3H).
^{13}C NMR (126 MHz, $CDCl_3$) δ 166.6, 157.8 (d, *J* = 257.0 Hz), 148.4 (d, *J* = 15.2 Hz), 145.4 (d, *J* = 5.5 Hz), 143.7, 130.0, 129.1, 129.0, 123.3 (d, *J* = 3.6 Hz), 123.2 (d, *J* = 19.1 Hz), 61.0, 38.3 (d, *J* = 2.1 Hz), 14.5.
^{19}F NMR (377 MHz, $CDCl_3$) δ –124.3.
HRMS (ESI) m/z: $[M+H]^+$ Calcd for $C_{15}H_{15}FNO_2$, 260.1081; Found 260.1078

(11) ethyl 4-(pyridin-3-ylmethyl)benzoate

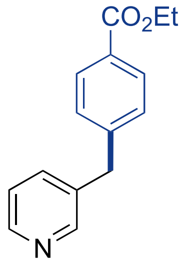

Reaction conducted following **Procedure A** using 3-(chloromethyl)pyridine hydrochloride (82.0 mg, 0.5 mmol, 1 equiv). Following workup, the obtained residue was purified by column chromatography on silica gel eluting with a solvent mixture of ethyl acetate:hexanes (25:75 (v:v)) to afford 85.6 mg (71% yield) of **11** as a clear oil.

^{1}H NMR (500 MHz, $CDCl_3$) δ 8.44 (s, 2H), 8.00 – 7.79 (m, 2H), 7.37 (d, *J* = 7.8 Hz, 1H), 7.17 (m, 3H), 4.29 (q, *J* = 7.1 Hz, 2H), 3.96 (s, 2H), 1.31 (t, *J* = 7.1 Hz, 3H).
^{13}C NMR (126 MHz, $CDCl_3$) δ 166.5, 145.1, 136.5, 130.1, 129.0, 129.0, 61.0, 39.2, 14.5.
HRMS (ESI) m/z: $[M+H]^+$ Calcd for $C_{15}H_{16}NO_2$, 242.1176; Found 242.1172

(12) ethyl 4-((3-(trifluoromethyl)pyridin-4-yl)methyl)benzoate

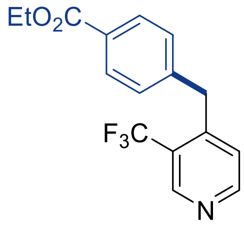

Reaction conducted following **Procedure A** using 4-(chloromethyl)-3-(trifluoromethyl)pyridine hydrochloride (116.0 mg, 0.5 mmol, 1 equiv). Following workup, the obtained residue was purified by column chromatography on silica gel eluting with a solvent mixture of ethyl acetate:hexanes (25:75 (v:v)) to afford 32.5 mg (21% yield) of **12** as a clear oil.

^{1}H NMR (500 MHz, $CDCl_3$) δ 8.89 (s, 1H), 8.64 (d, *J* = 5.1 Hz, 1H), 8.02 (d, *J* = 8.3 Hz, 2H), 7.22 (d, *J* = 8.3 Hz, 2H), 7.04 (d, *J* = 5.2 Hz, 1H), 4.38 (q, *J* = 7.1 Hz, 2H), 4.23 (s, 2H), 1.39 (t, *J* = 7.1 Hz, 3H).
^{13}C NMR (126 MHz, $CDCl_3$) δ 166.4, 152.9, 148.3, 146.9 (q, *J* = 6.3 Hz), 142.5, 130.2, 129.3, 125.6, 125.2, 123.8 (q, *J* = 275.3 Hz), 122.8, 61.1, 37.4 (d, *J* = 2.2 Hz), 14.3.
^{19}F NMR (377 MHz, $CDCl_3$) δ –59.9.
HRMS (ESI) m/z: $[M+H]^+$ Calcd for $C_{16}H_{15}F_3NO_2$, 310.1049; Found 310.1051

(13) ethyl 4-((6-(trifluoromethyl)pyridin-3-yl)methyl)benzoate

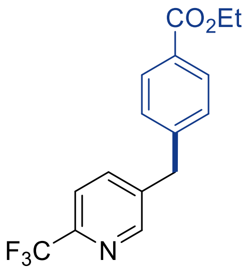

Reaction conducted following **Procedure A** using 5-(chloromethyl)-2-(trifluoromethyl)pyridine (97.5 mg, 0.5 mmol, 1 equiv). Deviation from procedure is the exclusion of Barton's base. Following workup, the obtained residue was purified by column chromatography on silica gel eluting with a solvent mixture of ethyl acetate:hexanes (25:75 (v:v)) to afford 147 mg (95% yield) of **13** as a clear oil.

^{1}H NMR (500 MHz, $CDCl_3$) δ 8.61 (s, 1H), 8.00 (d, J = 8.3 Hz, 2H), 7.61 (s, 2H), 7.24 (d, J = 8.3 Hz, 2H), 4.37 (q, J = 7.1 Hz, 3H), 4.11 (s, 3H), 1.38 (t, J = 7.1 Hz, 4H).
^{13}C NMR (126 MHz, $CDCl_3$) δ 166.4, 150.5, 146.7 (q, *J* = 34.8 Hz), 143.8, 139.2, 137.6, 130.3, 129.5, 129.0, 121.7 (q, *J* = 273.9 Hz), 120.5 (q, *J* = 2.7 Hz), 61.2, 38.9, 14.5.
^{19}F NMR (377 MHz, CDCl3) δ –67.8.
HRMS (ESI) m/z: $[M+H]^+$ Calcd for $C_{16}H_{15}F_3NO_2$, 310.1049; Found 310.1049

(14) ethyl 4-((5-cyanopyridin-3-yl)methyl)benzoate

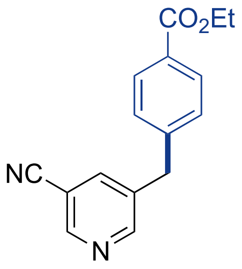

Reaction conducted following **Procedure A** using 5-(chloromethyl)nicotinonitrile hydrochloride (94.5 mg, 0.5 mmol, 1 equiv). Following workup, the obtained residue was purified by column chromatography on silica gel eluting with a solvent mixture of ethyl acetate:hexanes (25:75 (v:v)) to afford 79.8 mg (60% yield) of **14** as a clear oil.

^{1}H NMR (500 MHz, $CDCl_3$) δ 8.75 (d, *J* = 2.0 Hz, 1H), 8.69 (d, *J* = 2.2 Hz, 1H), 8.02 (d, *J* = 8.3 Hz, 2H), 7.70 (t, *J* = 2.0 Hz, 1H), 7.23 (d, *J* = 8.0 Hz, 2H), 4.38 (q, *J* = 7.2 Hz, 2H), 4.09 (s, 2H), 1.39 (t, *J* = 7.1 Hz, 3H).
^{13}C NMR (126 MHz, $CDCl_3$) δ 166.3, 153.6, 150.6, 143.1, 139.3, 136.5, 130.5, 129.7, 129.1, 116.5, 110.2, 61.2, 38.7, 14.5.
HRMS (ESI) m/z: $[M+H]^+$ Calcd for $C_{16}H_{15}N_2O_2$, 267.1128; Found 267.1128

(15) ethyl 4-(pyridin-4-ylmethyl)benzoate

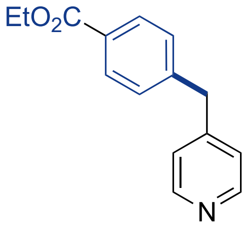

Reaction conducted following **Procedure A** using 3-(chloromethyl)pyridine hydrochloride (82.0 mg, 0.5 mmol, 1 equiv). Following workup, the obtained residue was purified by column chromatography on silica gel eluting with a solvent mixture of ethyl acetate:hexanes (25:75 (v:v)) to afford 60.3 mg (50% yield) of **15** as a clear oil.

^{1}H NMR (500 MHz, $CDCl_3$) δ 8.50 (d, J = 4.4 Hz, 2H), 7.99 (d, J = 8.4 Hz, 2H), 7.24 (d, J = 7.9 Hz, 2H), 7.09 – 7.08 (d, J = 4.9 Hz, 2H), 4.42 – 4.29 (q, J = 7.2 Hz, 2H), 4.01 (s, 2H), 1.41 – 1.36 t, J = 7.2 Hz, 3H).
^{13}C NMR (126 MHz, $CDCl_3$) δ 166.5, 150.1, 149.2, 144.1, 130.2, 129.2, 129.2, 124.3, 61.1, 41.3, 14.5.
HRMS (ESI) m/z: $[M+H]^+$ Calcd for $C_{15}H_{16}NO_2$, 242.1176; Found 242.1176

(16) ethyl 4-((5-chloropyrazin-2-yl)methyl)benzoate

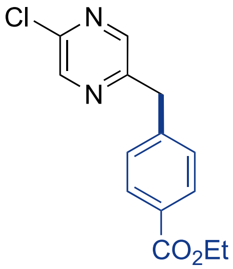

Reaction conducted following **Procedure A** using 2-chloro-5-(chloromethyl)pyrazine (81.0 mg, 0.5 mmol, 1 equiv). Deviation from procedure is the exclusion of Barton's base. Following workup, the obtained residue was purified by column chromatography on silica gel eluting with a solvent mixture of ethyl acetate:hexanes (25:75 (v:v)) to afford 160.1 mg (58% yield) of **16** as a brown oil.

^{1}H NMR (500 MHz, $CDCl_3$) δ 8.52 (d, J = 1.4 Hz, 1H), 8.24 (d, J = 1.5 Hz, 1H), 8.00 (d, J = 8.3 Hz, 2H), 7.32 (d, J = 8.3 Hz, 2H), 4.37 (q, J = 7.1 Hz, 2H), 4.20 (s, 2H), 1.38 (t, J = 7.1 Hz, 3H).
^{13}C NMR (126 MHz, $CDCl_3$) δ 166.4, 153.8, 147.7, 144.3, 143.6, 142.9, 130.3, 129.5, 129.1, 61.1, 41.0, 14.5.
HRMS (ESI) m/z: $[M+H]^+$ Calcd for $C_{14}H_{14}ClN_2O_2$, 277.0738; Found 277.0739

(17) ethyl 4-((4-methylquinazolin-2-yl)methyl)benzoate

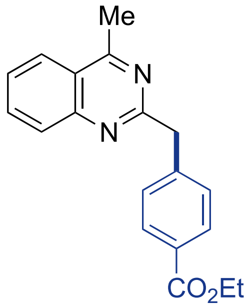

Reaction conducted following **Procedure A** using 2-(chloromethyl)-4-methylquinazoline (96.3 mg, 0.5 mmol, 1 equiv). Deviation from procedure is the exclusion of Barton's base. Following workup, the obtained residue was purified by column chromatography on silica gel eluting with a solvent mixture of ethyl acetate:hexanes (25:75 (v:v)) to afford 82.7 mg (54% yield) of **17** as a white solid.

^{1}H NMR (500 MHz, $CDCl_3$): δ 8.05 (m, 1H), 7.97 (m, 3H), 7.85 (ddd, *J* = 8.4, 7.0, 1.3 Hz, 1H), 7.58 (ddd, *J* = 8.3, 6.9, 1.3 Hz, 1H), 7.50 (d, *J* = 8.4 Hz, 2H), 4.45 (s, 2H), 4.34 (q, *J* = 7.1 Hz, 2H), 2.91 (s, 3H), 1.36 (t, *J* = 7.1 Hz, 3H).
^{13}C NMR (126 MHz, $CDCl_3$): δ 168.9, 166.8, 164.5, 150.1, 144.0, 133.8, 129.8, 129.4, 128.8, 127.2, 125.1, 122.7, 60.9, 46.3, 21.9, 14.5.
HRMS (ESI) m/z: $[M+H]^+$ Calcd for $C_{19}H_{19}N_2O_2$, 307.1441; Found 307.1438

(18) ethyl 4-((4-chloro-5,6,7,8-tetrahydrobenzofuro[2,3-*d*]pyrimidin-2-yl)methyl)benzoate

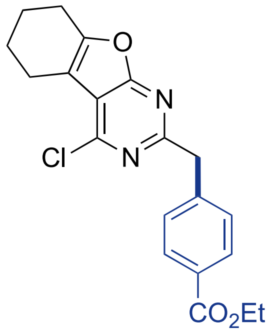

Reaction conducted following **Procedure A** using 4-chloro-2-(chloromethyl)-5,6,7,8-tetrahydrobenzofuro[2,3-*d*]pyrimidine (128.0 mg, 0.5 mmol, 1 equiv). Deviation from procedure is the exclusion of Barton's base. Following workup, the obtained residue was purified by column chromatography on silica gel eluting with a solvent mixture of ethyl acetate:hexanes (25:75 (v:v)) to afford 130.8 mg (71% yield) of **18** as a clear oil.

^{1}H NMR (500 MHz, $CDCl_3$): δ 7.98 (d, *J* = 8.3 Hz, 2H), 7.46 (d, *J* = 8.4 Hz, 2H), 4.39 – 4.33 (m, 4H), 2.80 (tt, *J* = 6.0, 2.1 Hz, 2H), 2.76 (tt, *J* = 6.3, 2.1 Hz, 2H), 1.98 – 1.92 (m, 2H), 1.90 – 1.83 (m, 2H), 1.38 (t, *J* = 7.1 Hz, 3H).
^{13}C NMR (126 MHz, $CDCl_3$): δ 167.1, 166.5, 163.0, 155.3, 151.2, 143.1, 129.8, 129.2, 128.9, 115.7, 111.4, 60.8, 45.2, 23.2, 22.2, 22.1, 21.0, 14.3.
HRMS (ESI) m/z: $[M+H]^+$ Calcd for $C_{20}H_{20}ClN_2O_3$, 371.1157; Found 371.1152

(19) ethyl 4-(3-phenyl-1-(pyridin-4-yl)propyl)benzoate

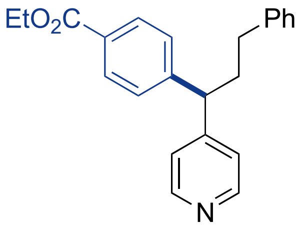

Reaction conducted following **Procedure B** using 4-(1-chloro-3-phenylpropyl)pyridine (115.5 mg, 0.5 mmol, 1 equiv). Following workup, the obtained residue was purified by column chromatography on silica gel eluting with a solvent mixture of ethyl acetate:hexanes (60:40 (v:v)) to afford 124.3 mg (72% yield) of **19** as a clear oil.

^{1}H NMR (500 MHz, $CDCl_3$) δ 8.58 – 8.44 (m, 2H), 8.00 (d, *J* = 8.4 Hz, 2H), 7.33 – 7.27 (m, 4H), 7.22 – 7.18 (m, 1H), 7.16 – 7.09 (m, 4H), 4.37 (q, *J* = 7.1 Hz, 2H), 3.94 (t, *J* = 7.7 Hz, 1H), 2.57 (m, 2H), 2.41 (m, 2H), 1.38 (t, *J* = 7.1 Hz, 3H).
^{13}C NMR 126 MHz, $CDCl_3$) δ 166.4, 152.9, 150.2, 148.0, 141.2, 130.2, 129.3, 128.6, 128.5, 128.1, 126.3, 123.2, 61.0, 50.0, 36.4, 33.8, 14.4.
HRMS (ESI) m/z: $[M+H]^+$ Calcd for $C_{23}H_{24}NO_2$, 346.1802; Found 346.1797

(20) ethyl 4-(4-methyl-1-(3-methylpyridin-4-yl)pentyl)benzoate

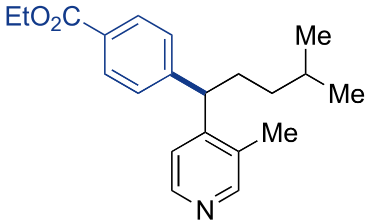

Reaction conducted following **Procedure B** using 4-(1-chloro-4-methylpentyl)-3-methylpyridine (105.6 mg, 0.5 mmol, 1 equiv). Following workup, the obtained residue was purified by column chromatography on silica gel eluting with a solvent mixture of ethyl acetate:hexanes (60:40 (v:v)) to afford 87.8 mg (54% yield) of **20** as a clear oil.

^{1}H NMR (500 MHz, $CDCl_3$) δ 8.44 (d, *J* = 5.1 Hz, 1H), 8.34 (s, 1H), 7.95 (d, *J* = 8.3 Hz, 2H), 7.24 – 7.18 (m, 3H), 4.35 (q, *J* = 7.1 Hz, 2H), 4.05 (t, *J* = 7.6 Hz, 1H), 2.21 (s, 3H), 2.07 – 1.96 (m, 2H), 1.56 (hept, *J* = 6.6 Hz, 1H), 1.37 (t, *J* = 7.1 Hz, 3H), 1.16 (m, 2H), 0.87 (dd, *J* = 9.1, 6.6 Hz, 6H).
^{13}C NMR (126 MHz, $CDCl_3$) δ 166.5, 151.4, 151.0, 148.3, 148.1, 132.0, 130.0, 129.0, 128.3, 121.5, 61.0, 47.2, 37.1, 33.3, 28.2, 22.7, 16.8, 14.5.
HRMS (ESI) m/z: $[M+H]^+$ Calcd for $C_{21}H_{28}NO_2$, 326.2115; Found 326.2111

(21) ethyl 4-(pyridin-4-yl(tetrahydro-2*H*-pyran-4-yl)methyl)benzoate

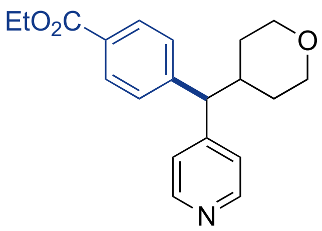

Reaction conducted following **Procedure B** using 4-(chloro(tetrahydro-2*H*-pyran-4-yl)methyl)pyridine (105.5 mg, 0.5 mmol, 1 equiv). Following workup, the obtained residue was purified by column chromatography on silica gel eluting with a solvent mixture of ethyl acetate:hexanes (60:40 (v:v)) to afford 100.8 mg (62% yield) of **21** as a clear oil.

^{1}H NMR (500 MHz, $CDCl_3$) δ 8.54 – 8.43 (m, 2H), 7.97 (d, *J* = 8.3 Hz, 2H), 7.33 (d, *J* = 8.3 Hz, 2H), 7.20 (d, *J* = 6.1 Hz, 2H), 4.34 (q, *J* = 7.1 Hz, 2H), 3.98 – 3.80 (m, 2H), 3.56 (d, *J* = 11.0 Hz, 1H), 3.36 (m, 2H), 2.36 (dt, *J* = 11.2, 3.7 Hz, 1H), 1.47 – 1.38 (m, 2H), 1.36 (t, *J* = 7.1 Hz, 3H), 1.31 – 1.22 (m, 2H).
^{13}C NMR (126 MHz, $CDCl_3$) δ 166.3, 151.3, 150.3, 146.6, 130.3, 129.5, 128.2, 123.5, 67.9, 61.1, 58.6, 38.4, 32.0, 14.4.
HRMS (ESI) m/z: $[M+H]^+$ Calcd for $C_{20}H_{24}NO_3$, 326.1751; Found 326.1749

(22) Ethyl 4-(1-(pyridin-4-yl)ethyl)benzoate

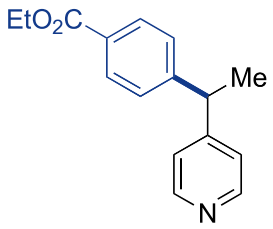

Reaction conducted following **Procedure B** using 4-(1-chloroethyl)pyridine (70.8 mg, 0.5 mmol, 1equiv). Following workup, the obtained residue was purified by column chromatography on silica gel eluting with a solvent mixture of ethyl acetate:hexanes (70:40 (v:v)) to afford 65.1 mg (49% yield) of **22** as a clear oil.

^{1}H NMR (500 MHz, $CDCl_3$) δ 8.50 (d, *J* = 6.1 Hz, 2H), 7.98 (d, *J* = 8.3 Hz, 2H), 7.26 (d, *J* = 8.2 Hz, 2H), 7.12 (d, *J* = 6.2 Hz, 2H), 4.36 (q, *J* = 7.1 Hz, 2H), 4.17 (q, *J* = 7.2 Hz, 1H), 1.66 (d, *J* = 7.2 Hz, 3H), 1.38 (t, *J* = 7.1 Hz, 3H).
^{13}C NMR (126 MHz, $CDCl_3$) δ 166.5, 154.4, 150.0, 149.6, 130.1, 129.2, 127.8, 123.1, 61.1, 44.4, 21.0, 14.5.
HRMS (ESI) m/z: $[M+H]^+$ Calcd for $C_{16}H_{18}NO_2$, 256.1332; Found 256.1333

(23) Ethyl 4-(1-(2-chloropyridin-4-yl)ethyl)benzoate

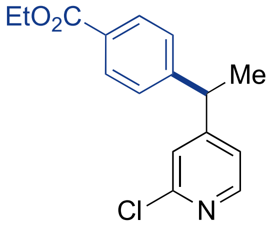

Reaction conducted following **Procedure B** using 2-chloro-4-(1-chloroethyl)pyridine (87.5 mg, 0.5 mmol, 1 equiv). Deviation from Procedure B was the exclusion of $MgCl_2$. Following workup, the obtained residue was purified by column chromatography on silica gel eluting with a solvent mixture of ethyl acetate:hexanes (30:70 (v:v)) to afford 117.1 mg (81 % yield) of **23** as a clear oil.

^{1}H NMR (500 MHz, $CDCl_3$) δ 8.27 (d, *J* = 5.2, 1H), 8.00 (d, *J* = 8.4 Hz, 2H), 7.25 (d, *J* = 8.3 Hz, 2H), 7.16 – 7.15 (m, 1H), 7.03 – 7.02 (m, 1H), 4.37 (q, *J* = 7.1 Hz, 2H), 4.16 (q, *J* = 7.2 Hz, 1H), 1.65 (d, *J* = 7.2 Hz, 3H), 1.38 (t, *J* = 7.1 Hz, 3H).
^{13}C NMR (126 MHz, $CDCl_3$) δ 166.4, 157.8, 152.1, 149.9, 148.70, 130.2, 129.5, 127.7, 123.4, 121.9, 61.1, 44.2, 20.9, 14.5.
HRMS (ESI m/z: $[M+H]^+$ Calcd for $C_{16}H_{17}ClNO_2$, 290.0942; Found 290.0936

(24) ethyl 4-(6,7-dihydro-5*H*-cyclopenta[*b*]pyridin-7-yl)benzoate

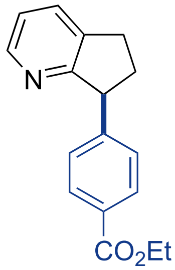

Reaction conducted following **Procedure D** using 7-chloro-6,7-dihydro-5*H*-cyclopenta[*b*]pyridine (76.8 mg, 0.5 mmol, 1 equiv). Following workup, the obtained residue was purified by column chromatography on silica gel eluting with a solvent mixture of ethyl acetate:hexanes (25:75 (v:v)) to afford 104.3 mg (78% yield) of **24** as a clear oil.

^{1}H NMR (500 MHz, $CDCl_3$) δ 8.41 (d, *J* = 5.0 Hz, 1H), 8.01 (d, *J* = 8.3 Hz, 2H), 7.62 (d, *J* = 7.6 Hz, 1H), 7.30 – 7.25 (d, *J* = 8.3 Hz, 2H), 7.13 (dd, *J* = 7.6, 4.9 Hz, 1H), 4.50 (t, *J* = 8.2 Hz, 1H), 4.38 (q, *J* = 7.1 Hz, 2H), 3.12 (m, 1H), 3.02 (m, 1H), 2.71 (m, 1H), 2.18 (m, 1H), 1.39 (t, *J* = 7.1 Hz, 3H).
^{13}C NMR (126 MHz, $CDCl_3$) δ 166.6, 165.9, 149.3, 148.2, 137.3, 132.6, 130.0, 128.8, 128.1, 121.7, 60.8, 51.8, 33.8, 29.4, 14.4.
HRMS (ESI) m/z: $[M+H]^+$ Calcd for $C_{17}H_{18}NO_2$, 268.1332; Found 268.1326

(25) ethyl 4-(1-(pyridin-2-yl)ethyl)benzoate

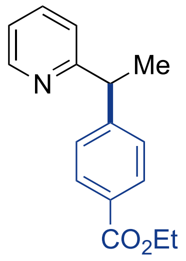

Reaction conducted following **Procedure D** using 2-(1-chloroethyl)pyridine (70.8 mg, 0.5 mmol, 1 equiv). Following workup, the obtained residue was purified by column chromatography on silica gel eluting with a solvent mixture of ethyl acetate:hexanes (25:75 (v:v)) to afford 108.5 mg (85% yield) of **25** as a clear oil.

^{1}H NMR (500 MHz, $CDCl_3$) δ 8.56 (d, *J* = 3.4 Hz, 1H), 7.97 (d, *J* = 8.4 Hz, 2H), 7.60 – 7.55 (m, 1H), 7.36 (d, *J* = 8.2 Hz, 2H), 7.15 – 7.09 (m, 2H), 4.38 – 4.31 (m 3H), 1.72 (d, *J* = 7.3 Hz, 3H), 1.36 (t, *J* = 7.1 Hz, 3H).
^{13}C NMR (126 MHz, $CDCl_3$) δ 166.7, 164.2, 150.4, 149.4, 136.7, 129.9, 128.8, 127.8, 122.3, 121.6, 60.9, 47.5, 20.7, 14.4.
HRMS (ESI) m/z: $[M+H]^+$ Calcd for $C_{16}H_{18}NO_2$, 256.1332; Found 256.1328

(26) ethyl 4-(1-(pyridin-3-yl)ethyl)benzoate

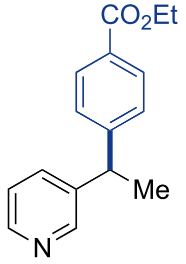

Reaction conducted following **Procedure C** using 3-(1-chloroethyl)pyridine (70.8 mg, 0.5 mmol, 1 equiv). Following workup, the obtained residue was purified by column chromatography on silica gel eluting with a solvent mixture of ethyl acetate:hexanes (25:75 (v:v)) to afford 77.9 mg (61% yield) of **26** as a clear oil.

^{1}H NMR (500 MHz, $CDCl_3$) δ 8.52 (d, *J* = 2.4 Hz, 1H), 8.46 (dd, *J* = 4.8, 1.7 Hz, 1H), 7.98 (d, *J* = 8.4 Hz, 2H), 7.47 (d, *J* = 8.0 Hz, 1H), 7.27 (d, *J* = 8.3 Hz, 2H), 7.21 (dd, *J* = 7.8, 4.8 Hz, 1H), 4.36 (q, *J* = 7.1 Hz, 2H), 4.23 (q, *J* = 7.2 Hz, 1H), 1.68 (d, *J* = 7.2 Hz, 3H), 1.37 (t, *J* = 7.1 Hz, 3H).
^{13}C NMR (126 MHz, $CDCl_3$) δ 166.5, 150.2, 149.4, 147.9, 140.9, 129.0, 127.7, 123.6, 61.0, 42.6, 21.5, 14.5.
HRMS (ESI) m/z: $[M+H]^+$ Calcd for $C_{16}H_{18}NO_2$, 256.1332; Found 256.1328

(27) ethyl 4-(1-(pyridin-3-yl)propyl)benzoate

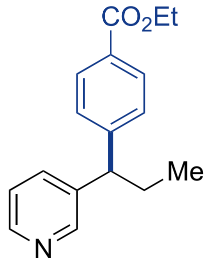

Reaction conducted following **Procedure C** using 3-(1-chloropropyl)pyridine (77.8 mg, 0.5 mmol, 1 equiv). Deviation from Procedure C running the reaction at 40 ºC. Following workup, the obtained residue was purified by column chromatography on silica gel eluting with a solvent mixture of ethyl acetate:hexanes (25:75 (v:v)) to afford 78.1 (58% yield) of **27** as a clear oil.

^{1}H NMR (500 MHz, $CDCl_3$) δ 8.53 (s, 1H), 8.45 (d, *J* = 3.2 Hz, 1H), 7.97 (d, *J* = 8.3 Hz, 2H), 7.49 (dt, *J* = 7.9, 2.0 Hz, 1H), 7.29 (d, *J* = 8.3 Hz, 2H), 7.21 (dd, *J* = 7.9, 4.8 Hz, 1H), 4.35 (q, *J* = 7.1 Hz, 2H), 3.87 (t, *J* = 7.8 Hz, 1H), 2.19 – 2.03 (m, 2H), 1.37 (t, *J* = 7.1 Hz, 3H), 0.91 (t, *J* = 7.3 Hz, 3H).
^{13}C NMR (126 MHz, $CDCl_3$) δ 166.5, 149.7, 149.1, 148.0, 139.8, 135.2, 130.1, 129.0, 128.0, 123.7, 61.0, 50.9, 28.3, 14.5, 12.7.
HRMS (ESI) m/z: $[M+H]^+$ Calcd for $C_{17}H_{20}NO_2$, 270.1489; Found 270.1488

(28) ethyl 4-(1-(pyrazin-2-yl)ethyl)benzoate

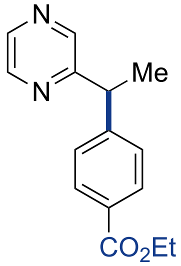

Reaction conducted following **Procedure D** using 2-(1-chloroethyl)pyrazine (71.0 mg, 0.5 mmol, 1 equiv). Following workup, the obtained residue was purified by column chromatography on silica gel eluting with a solvent mixture of ethyl acetate:hexanes (25:75 (v:v)) to afford 69.2 mg (54% yield) of **28** as a clear oil.

^{1}H NMR (500 MHz, $CDCl_3$) δ 8.51 (s, 1H), 8.45 (s, 1H), 8.39 (d, *J* = 2.5 Hz, 1H), 7.98 (d, *J* = 8.4 Hz, 2H), 7.36 (d, *J* = 8.3 Hz, 2H), 4.47 – 4.26 (m, 3H), 1.73 (d, *J* = 7.2 Hz, 3H), 1.36 (t, *J* = 7.1 Hz, 3H).
^{13}C NMR (126 MHz, $CDCl_3$) δ 166.5, 159.6, 149.0, 144.3, 144.2, 142.7, 130.1, 129.2, 127.8, 61.0, 45.2, 20.4, 14.4.
HRMS (ESI) m/z: $[M+H]^+$ Calcd for $C_{15}H_{17}N_2O_2$, 257.1285; Found 257.1279

(29) ethyl 4-(1-(pyrimidin-5-yl)ethyl)benzoate

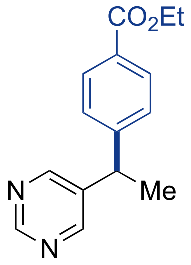

Reaction conducted following **Procedure C** using 5-(1-chloroethyl)pyrimidine (71.3 mg, 0.5 mmol, 1 equiv). Following workup, the obtained residue was purified by column chromatography on silica gel eluting with a solvent mixture of ethyl acetate:hexanes (25:75 (v:v)) to afford 64.0 mg (50% yield) of **29** as a clear oil.

^{1}H NMR (500 MHz, $CDCl_3$) δ 9.09 (s, 1H), 8.59 (s, 2H), 8.01 (d, J = 8.4 Hz, 2H), 7.28 (d, J = 8.2 Hz, 2H), 4.37 (q, J = 7.1 Hz, 2H), 4.22 (q, J = 7.3 Hz, 1H), 1.72 (d, J = 7.3 Hz, 3H), 1.38 (t, J = 7.1 Hz, 3H).
^{13}C NMR 126 MHz, $CDCl_3$) δ 166.2, 157.1, 156.1, 148.6, 138.3, 130.2, 129.4, 127.5, 61.1, 40.6, 21.0, 14.3.
HRMS (ESI) m/z: $[M+H]^+$ Calcd for $C_{15}H_{17}N_2O_2$, 257.1285; Found 257.1283

(30) ethyl 4-(1-(2-chloropyrimidin-5-yl)ethyl)benzoate

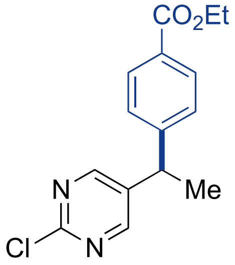

Reaction conducted following **Procedure C** using 2-chloro-5-(1-chloroethyl)pyrimidine (88.0 mg, 0.5 mmol, 1 equiv). Following workup, the obtained residue was purified by column chromatography on silica gel eluting with a solvent mixture of ethyl acetate:hexanes (25:75 (v:v)) to afford 150.8 mg (52% yield) of **30** as a clear oil.

^{1}H NMR (500 MHz, $CDCl_3$) δ 8.47 (s, 2H), 8.02 (d, J = 8.3 Hz, 2H), 7.26 (d, J = 8.1 Hz, 2H), 4.38 (q, J = 7.1 Hz, 2H), 4.23 (q, J = 7.2 Hz, 1H), 1.71 (d, J = 7.2 Hz, 3H), 1.39 (t, J = 7.1 Hz, 3H).
^{13}C NMR (126 MHz, $CDCl_3$) δ 166.2, 159.8, 158.9, 148.1, 137.1, 130.4, 129.7, 127.5, 61.2, 40.0, 21.2, 14.4.
HRMS (ESI) m/z: $[M+H]^+$ Calcd for $C_{15}H_{16}ClN_2O_2$, 291.0895; Found 291.0893

(31) 7-(6-chloropyridin-3-yl)-6,7-dihydro-5H-cyclopenta[b]pyridine

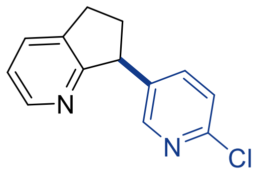

Reaction conducted following **Procedure D** using 7-chloro-6,7-dihydro-5H-cyclopenta[b]pyridine (76.8 mg, 0.5 mmol, 1 equiv). Following workup, the obtained residue was purified by column chromatography on silica gel eluting with a solvent mixture of ethyl acetate:hexanes (50:50 (v:v)) to afford 32.2 mg (28% yield) of **31** as a clear oil.

^{1}H NMR (500 MHz, $CDCl_3$) δ 8.37 (dd, *J* = 4.9, 1.5 Hz, 1H), 8.27 (d, *J* = 2.5 Hz, 1H), 7.60 (dd, *J* = 7.5, 1.4 Hz, 1H), 7.44 (dd, *J* = 8.2, 2.5 Hz, 1H), 7.25 (d, *J* = 8.2 Hz, 1H), 7.11 (ddd, *J* = 7.6, 5.0, 0.8 Hz, 1H), 4.41 (t, *J* = 8.4 Hz, 1H), 3.12 – 2.97 (m, 2H), 2.69 (dtd, *J* = 12.6, 8.2, 4.2 Hz, 1H), 2.08 (dq, *J* = 13.0, 8.4 Hz, 1H).
^{13}C NMR (126 MHz, $CDCl_3$) δ 164.8, 149.6, 149.5, 148.3, 138.4, 138.2, 137.2, 132.7, 124.2, 122.0, 48.4, 33.8, 29.3.
HRMS (ESI) m/z: $[M+H]^+$ Calcd for $C_{13}H_{11}ClN_2$, 231.0683; Found 231.0682

(32) 7-(6-(trifluoromethyl)pyridin-3-yl)-6,7-dihydro-5*H*-cyclopenta[*b*]pyridine

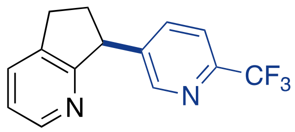

Reaction conducted following **Procedure D** using 7-chloro-6,7-dihydro-5H-cyclopenta[b]pyridine (76.8 mg, 0.5 mmol, 1 equiv). Following workup, the obtained residue was purified by column chromatography on silica gel eluting with a solvent mixture of ethyl acetate:hexanes (50:50 (v:v)) to afford 76.6 mg (58% yield) of **32** as a clear oil.

^{1}H NMR (500 MHz, $CDCl_3$) δ 8.61 (d, *J* = 2.1 Hz, 1H), 8.39 (dd, *J* = 4.9, 1.4 Hz, 1H), 7.68 – 7.59 (m, 3H), 7.14 (ddd, *J* = 7.6, 5.0, 0.8 Hz, 1H), 4.51 (t, *J* = 8.5 Hz, 1H), 3.18 – 2.96 (m, 2H), 2.74 (dtd, *J* = 12.7, 8.2, 4.2 Hz, 1H), 2.13 (dq, *J* = 13.0, 8.5 Hz, 1H).
^{13}C NMR (126 MHz, $CDCl_3$) δ 164.6, 150.1, 148.5, 146.5 (q, *J* = 34.6 Hz), 142.7, 137.2, 136.8, 132.8, 122.1, 121.7 (q, *J* = 273.8 Hz), 120.4 (q, *J* = 2.7 Hz), 49.1, 33.9, 29.4.
^{19}F NMR (377 MHz, $CDCl_3$) δ –67.8.
HRMS (ESI) m/z: $[M+H]^+$ Calcd for $C_{14}H_{12}F_3N_2$ 265.0947; Found 265.0945

(33) 4-(5-(5,6,7,8-tetrahydroisoquinolin-5-yl)pyridin-2-yl)morpholine

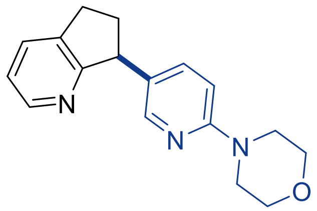

Reaction conducted following **Procedure D** using 7-chloro-6,7-dihydro-5H-cyclopenta[b]pyridine (46.1 mg, 0.3 mmol, 1 equiv). Following workup, the obtained residue was purified by column chromatography on silica gel eluting with a solvent gradient of ethyl acetate:hexanes (5:95 (v:v) – 60:40 (v:v)) to afford 76.0 mg (90% yield) of **33** as a clear oil.

^{1}H NMR (500 MHz, $CDCl_3$) δ 8.37 (d, *J* = 4.9 Hz, 1H), 8.08 (d, *J* = 2.5 Hz, 1H), 7.56 (dq, *J* = 7.5, 1.2 Hz, 1H), 7.30 (dd, *J* = 8.7, 2.5 Hz, 1H), 7.07 (dd, *J* = 7.7, 4.9 Hz, 1H), 6.61 (d, *J* = 8.7 Hz, 1H), 4.31 (t, *J* = 8.3 Hz, 1H), 3.81 (t, *J* = 5.0 Hz, 4H), 3.45 (dd, *J* = 5.8, 4.0 Hz, 4H), 3.07 – 3.02 (m, 1H), 2.99 – 2.92 (m, 1H), 2.62 (dtd, *J* = 12.7, 8.3, 4.3 Hz, 1H), 2.07 (dq, *J* = 12.9, 8.3 Hz, 1H).
^{13}C NMR (126 MHz, $CDCl_3$) δ 166.4, 158.8, 148.4, 147.6, 137.4, 137.2, 132.5, 129.3, 121.6, 107.4, 67.0, 48.6, 46.1, 34.1, 29.4.
HRMS (ESI) m/z: $[M+H]^+$ Calcd for $C_{17}H_{19}N_3O$ 282.1601; Found 282.1598

(34) 2-chloro-5-(1-(6-(difluoromethyl)pyridin-3-yl)ethyl)pyrimidine

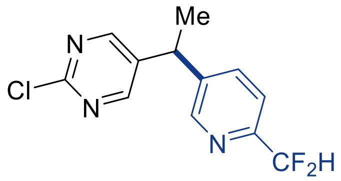

Reaction conducted following **Procedure C** using 2-chloro-5-(1-chloroethyl)pyrimidine (53.1 mg, 0.3 mmol, 1 equiv). Following workup, the obtained residue was purified by column chromatography on silica gel eluting with a solvent mixture of ethyl acetate:hexanes (50:50 (v:v)) to afford 22.7 mg (28% yield) of **33** as a clear oil.

^{1}H NMR (500 MHz, $CDCl_3$) δ 8.55 (d, *J* = 1.7 Hz, 1H), 8.49 (s, 2H), 7.63 (t, *J* = 1.8 Hz, 2H), 6.62 (t, *J* = 55.4 Hz, 1H), 4.27 (q, *J* = 7.3 Hz, 1H), 1.75 (d, *J* = 7.3 Hz, 3H).
^{13}C NMR (126 MHz, $CDCl_3$) δ 160.4, 158.9, 152.4 – 152.0 (t, *J* = 26.2 Hz), 149.0, 140.9, 140.9, 136.2, 136.2, 120.7 – 120.6 (t, *J* = 2.8 Hz), 115.8 – 112.0 (t, *J* = 241.0 Hz), 37.8, 21.2.
^{19}F NMR (377 MHz, $CDCl_3$) δ –115.7, –115.7.
HRMS (ESI) m/z: $[M+H]^+$ Calcd for $C_{12}H_{10}ClF_2N_3$ 270.0604; Found 270.0602

(35) 3-(1-(2-chloropyrimidin-5-yl)ethyl)-6,7-dihydro-5H-cyclopenta[b]pyridine

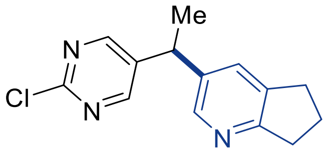

Reaction conducted following **Procedure C** using 2-chloro-5-(1-chloroethyl)pyrimidine (53.1 mg, 0.3 mmol, 1 equiv). Following workup, the obtained residue was purified by column chromatography on silica gel eluting with a solvent mixture of ethyl acetate:hexanes (50:50 (v:v)) to afford 18.7 mg (24% yield) of **34** as a clear oil.

^{1}H NMR (500 MHz, $CDCl_3$) δ 8.48 (s, 2H), 8.25 (d, *J* = 2.1 Hz, 1H), 7.31 – 7.27 (m, 1H), 4.18 (q, *J* = 7.3 Hz, 1H), 3.03 (t, *J* = 7.7 Hz, 2H), 2.92 (t, *J* = 7.5 Hz, 2H), 2.15 (p, *J* = 7.6 Hz, 2H), 1.70 (d, *J* = 7.3 Hz, 3H).
^{13}C NMR (126 MHz, CDCl3) δ 164.7, 159.9, 158.9, 145.9, 138.4, 137.0, 136.5, 131.7, 37.6, 33.7, 30.7, 23.5, 21.2.
HRMS (ESI) m/z: $[M+H]^+$ Calcd for $C_{14}H_{14}ClN_3$ 260.0949; Found 260.0946

(36) 2-(1-(2-chloropyrimidin-5-yl)ethyl)-4-methoxybenzo[d]thiazole

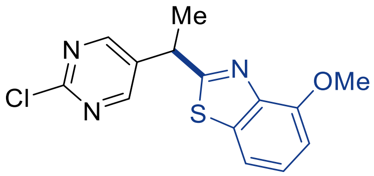

Reaction conducted following **Procedure C** using 2-chloro-5-(1-chloroethyl)pyrimidine (53.1 mg, 0.3 mmol, 1 equiv). Following workup, the obtained residue was purified by column chromatography on silica gel eluting with a solvent mixture of ethyl acetate:hexanes (50:50 (v:v)) to afford 9.5 mg (10% yield) of **36** as a clear oil.

^{1}H NMR (500 MHz, $CDCl_3$) δ 8.68 (s, 2H), 7.43 (dd, *J* = 8.1, 1.0 Hz, 1H), 7.34 (t, *J* = 8.1 Hz, 1H), 6.93 (dd, *J* = 8.0, 1.0 Hz, 1H), 4.69 (q, *J* = 7.2 Hz, 1H), 4.05 (s, 3H), 1.91 (d, *J* = 7.3 Hz, 3H).
^{13}C NMR (126 MHz, CDCl3) δ 171.1, 160.5, 159.0, 153.6, 143.2, 136.8, 135.1, 126.7, 113.8, 107.2, 56.2, 39.9, 21.3.
HRMS (ESI) m/z: $[M+H]^+$ Calcd for $C_{14}H_{12}ClN_3OS$ 306.0462; Found 306.0462

(37) methyl 1-(4-(5,6,7,8-tetrahydroisoquinolin-5-yl)phenyl)cyclopropane-1-carboxylate

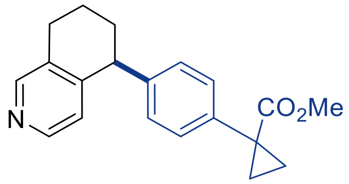

Reaction conducted following **Procedure B** using 5-chloro-5,6,7,8-tetrahydroisoquinoline (50.3 mg, 0.3 mmol, 1 equiv). Following workup, the obtained residue was purified by column chromatography on silica gel eluting with a solvent mixture of ethyl acetate:hexanes (75:25 (v:v)) to afford 16.6 mg (24% yield) of **35** as a clear oil.

^{1}H NMR (500 MHz, $CDCl_3$): δ 8.38 (s, 1H), 8.22 (d, *J* = 5.2 Hz, 1H), 7.29 – 7.20 (m, 2H), 7.05 – 6.92 (m, 2H), 6.78 (d, *J* = 5.1 Hz, 1H), 4.04 (dd, *J* = 8.2, 5.9 Hz, 1H), 3.63 (s, 3H), 2.93 – 2.80 (m, 2H), 2.21 – 2.13 (m, 1H), 1.96 (tdd, *J* = 13.4, 5.6, 2.4 Hz, 1H), 1.91 – 1.73 (m, 2H), 1.59 (t, *J* = 3.3 Hz, 2H), 1.20 – 1.16 (m, 2H).
^{13}C NMR (126 MHz, $CDCl_3$) δ 175.2, 150.1, 149.0, 146.4, 144.3, 137.9, 133.6, 130.7, 128.6, 124.8, 52.5, 45.0, 32.6, 28.8, 26.7, 20.9, 16.7, 16.7.
HRMS (ESI) m/z: $[M+H]^+$ Calcd for $C_{20}H_{21}NO_2$ 308.1645; Found 308.1642

(38) 7-chloro-3-(5,6,7,8-tetrahydroisoquinolin-5-yl)quinoline

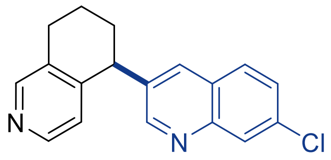

Reaction conducted following **Procedure B** using 5-chloro-5,6,7,8-tetrahydroisoquinoline (50.3 mg, 0.3 mmol, 1 equiv). Following workup, the obtained residue was purified by column chromatography on silica gel eluting with a solvent mixture of ethyl acetate:hexanes (75:25 (v:v)) to afford 21.2 mg (24% yield) of **36** as a clear oil.

^{1}H NMR (500 MHz, $CDCl_3$) δ 8.78 (d, *J* = 4.5 Hz, 1H), 8.50 (s, 1H), 8.26 (d, *J* = 5.3 Hz, 1H), 8.16 (d, *J* = 2.2 Hz, 1H), 7.95 (d, *J* = 9.1 Hz, 1H), 7.52 (dd, *J* = 9.1, 2.2 Hz, 1H), 6.84 (d, *J* = 4.5 Hz, 1H), 6.72 (d, *J* = 5.1 Hz, 1H), 4.83 (t, *J* = 6.7 Hz, 1H), 3.03 – 2.87 (m, 2H), 2.39 – 2.25 (m, 1H), 2.03 (dddd, *J* = 13.5, 8.8, 7.2, 3.2 Hz, 1H), 1.90 (ddtd, *J* = 16.1, 13.5, 10.4, 4.4 Hz, 2H).
^{13}C NMR (126 MHz, $CDCl_3$) δ 151.2, 151.2, 150.6, 149.3, 147.1, 146.8, 135.4, 133.8, 129.7, 128.0, 125.3, 124.8, 124.6, 122.0, 40.9, 30.7, 26.5, 20.5.
HRMS (ESI) m/z: $[M+H]^+$ Calcd for $C_{18}H_{15}ClN_2$ 295.0996; Found 295.0993

(39) 5-(6-chloropyridin-3-yl)-5,6,7,8-tetrahydroisoquinoline

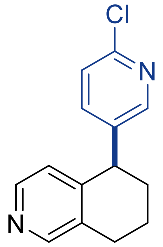

Reaction conducted following **Procedure B** using 5-chloro-5,6,7,8-tetrahydroisoquinoline (50.3 mg, 0.3 mmol, 1 equiv). Following workup, the obtained residue was purified by column chromatography on silica gel eluting with a solvent mixture of ethyl acetate:hexanes (75:25 (v:v)) to afford 8.5 mg (12% yield) of **39** as a clear oil.

^{1}H NMR (500 MHz, $CDCl_3$) δ 8.41 (s, 1H), 8.25 (d, *J* = 5.1 Hz, 1H), 8.20 (d, *J* = 2.5 Hz, 1H), 7.31 – 7.22 (m, 2H), 6.66 (d, *J* = 5.1 Hz, 1H), 4.13 – 4.00 (m, 1H), 2.94 – 2.81 (m, 2H), 2.24 – 2.15 (m, 1H), 2.00 – 1.89 (m, 1H), 1.89 – 1.75 (m, 2H).
^{13}C NMR (126 MHz, $CDCl_3$) δ 151.1, 150.0, 150.0, 147.3, 146.5, 140.0, 138.8, 133.3, 124.4, 124.1, 42.0, 32.6, 26.5, 20.8.
HRMS (ESI) m/z: $[M+H]^+$ Calcd for $C_{14}H_{13}ClN_2$ 245.0840; Found 245.0843

9. References

(1) Li, Z.; Wang, Y.; Fu, C.; Wang, X.; Wang, J. J.; Zhang, Y.; Zhou, D.; Zhao, Y.; Luo, L.; Ma, H.; Lu, W.; Zheng, J.; Zhang, X. Design, Synthesis, and Structure-Activity-Relationship of a Novel Series of CXCR4 Antagonists. *Eur. J. Med. Chem.* **2018**, *149*, 30–44. https://doi.org/10.1016/j.ejmech.2018.02.042.

(2) Liu, J.; Zhang, X.; Yi, H.; Liu, C.; Liu, R.; Zhang, H.; Zhuo, K.; Lei, A. Chloroacetate-Promoted Selective Oxidation of Heterobenzylic Methylenes under Copper Catalysis. *Angew. Chem. Int. Ed.* **2015**, *54*, 1261–1265. https://doi.org/10.1002/anie.201409580.

(3) Golden, D. L.; Flynn, K. M.; Aikonen, S.; Hanneman, C. M.; Kalyani, D.; Krska, S. W.; Paton, R. S.; Stahl, S. S. Radical Chlorination of Non-Resonant Heterobenzylic C–H Bonds and High-Throughput Diversification of Heterocycles. *Chem* **2024**, *10*, 1593–1605. https://doi.org/10.1016/j.chempr.2024.04.001.

(4) Wang, Y.; Huang, W.; Wang, C.; Qu, J.; Chen, Y. Nickel-Catalyzed Formal Aminocarbonylation of Secondary Benzyl Chlorides with Isocyanides. *Org. Lett.* **2020**, *22*, 4245–4249. https://doi.org/10.1021/acs.orglett.0c01284.

(5) Anka-Lufford, L. L.; Huihui, K. M. M.; Gower, N. J.; Ackerman, L. K. G.; Weix, D. J. Nickel-Catalyzed Cross-Electrophile Coupling with Organic Reductants in Non-Amide Solvents. *Chem. – Eur. J.* **2016**, *22*, 11564–11567. https://doi.org/10.1002/chem.201602668.

10. Spectral Data

^{1}H NMR (500 MHz, $CDCl_3$) of **4a**

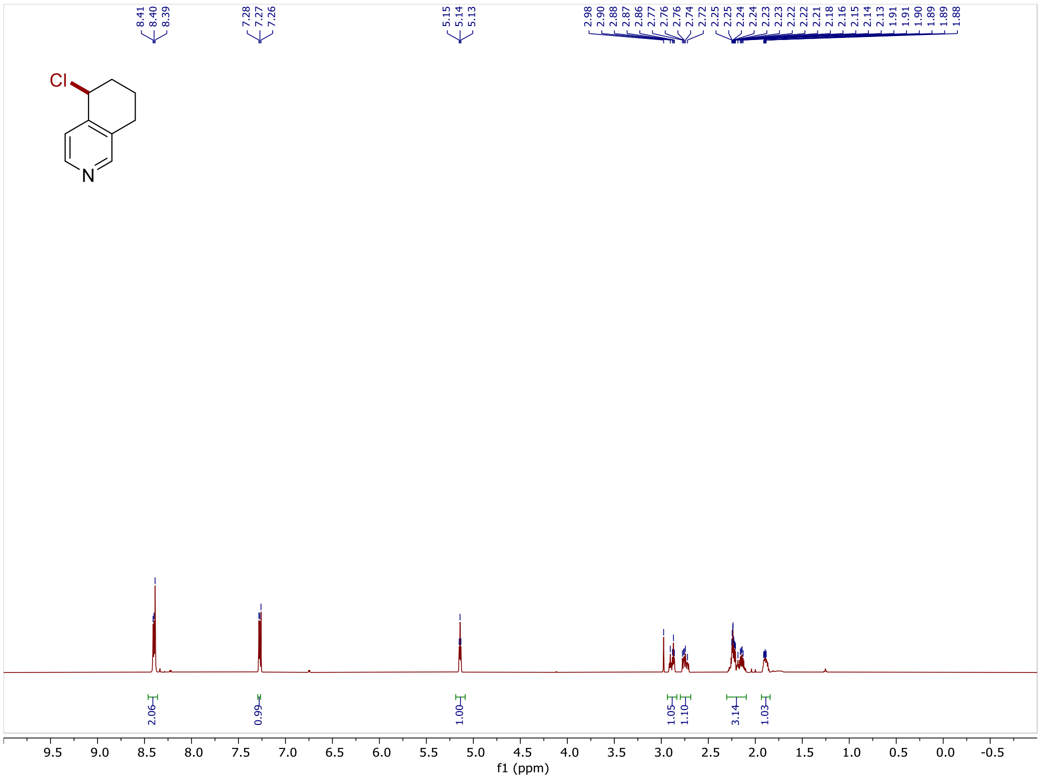

^{13}C NMR: (126 MHz, $CDCl_3$) **4a**

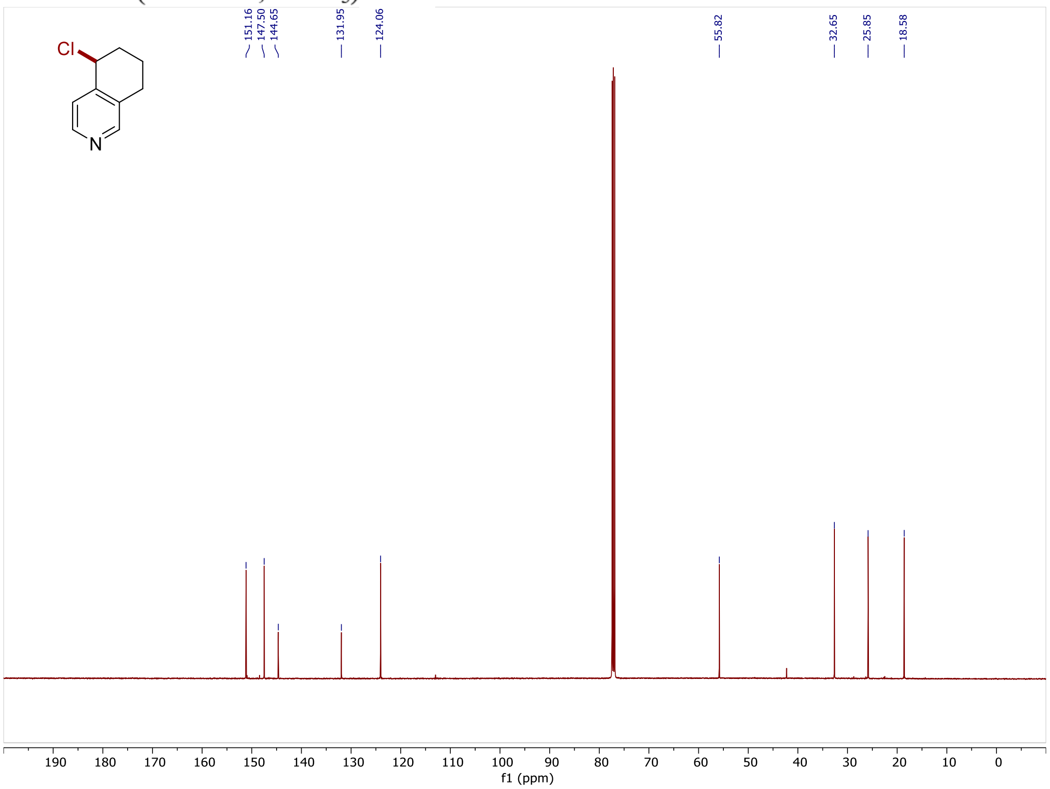

^{1}H NMR (500 MHz, $CDCl_3$) of **5a**

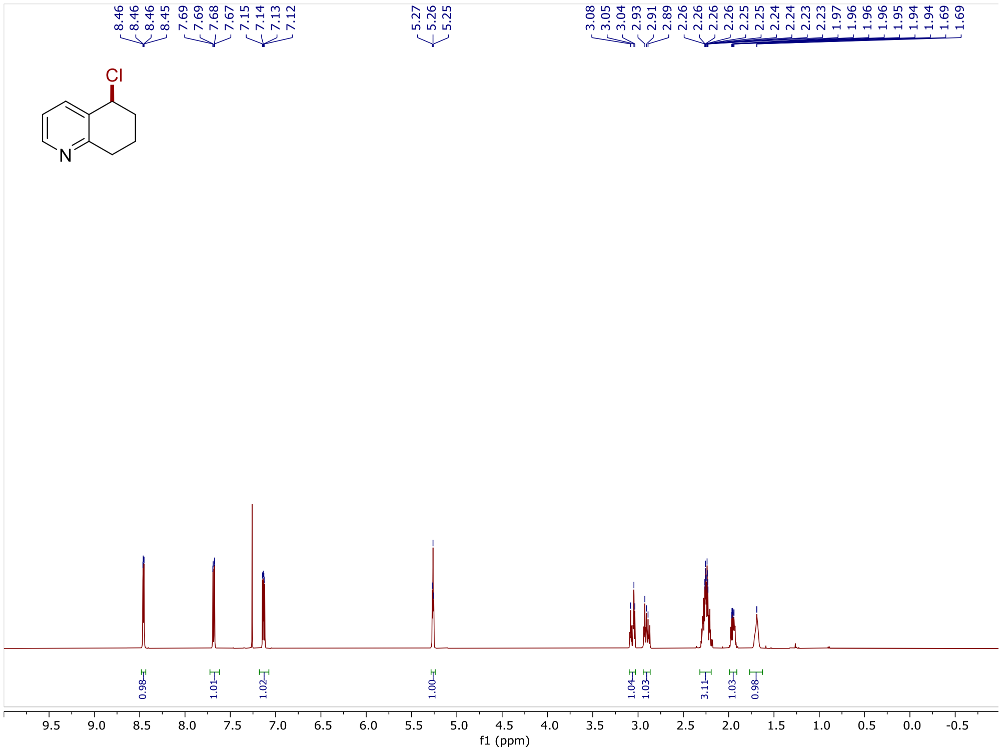

^{13}C NMR: (126 MHz, $CDCl_3$) of **5a**

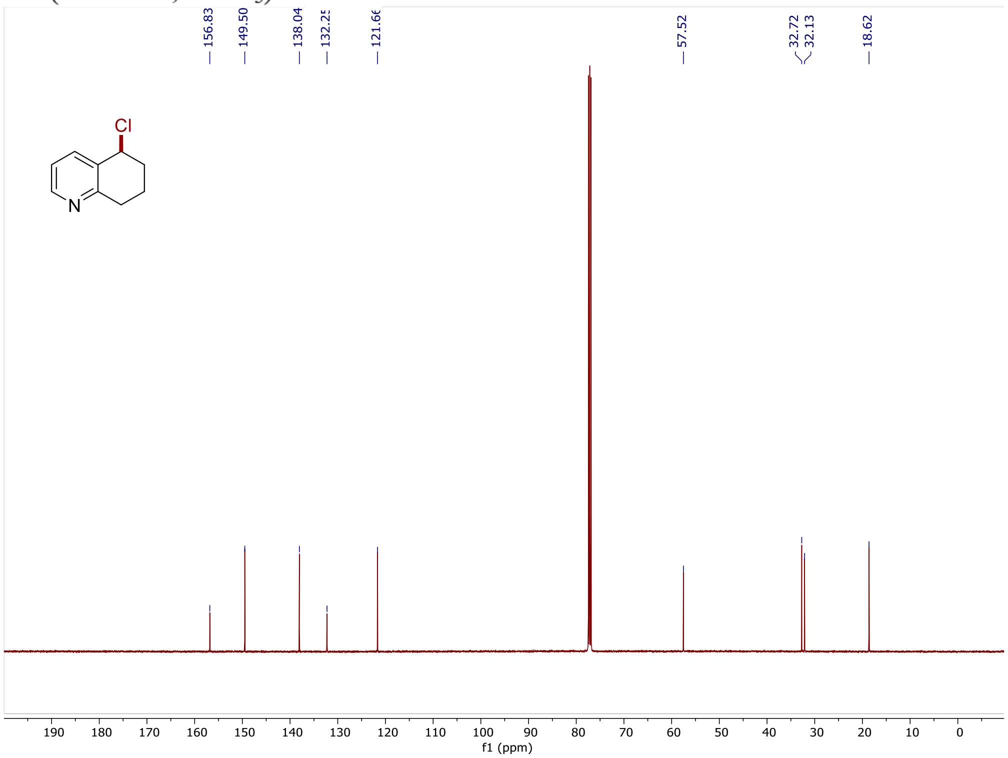

^{1}H NMR (500 MHz, $CDCl_3$) of **16a**

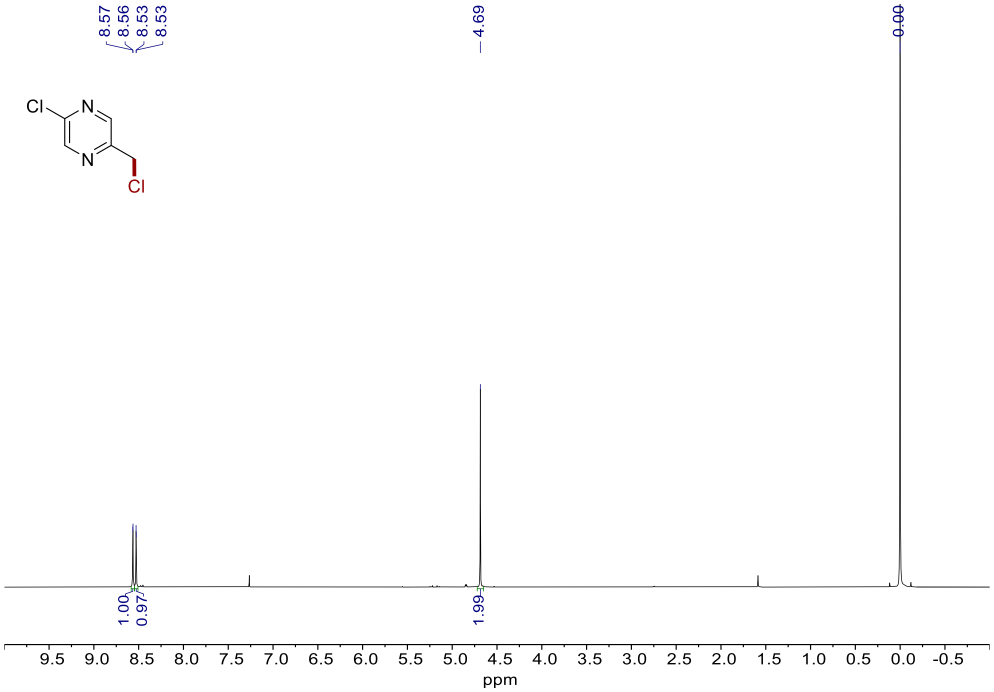

^{13}C NMR: (126 MHz, $CDCl_3$) of **16a**

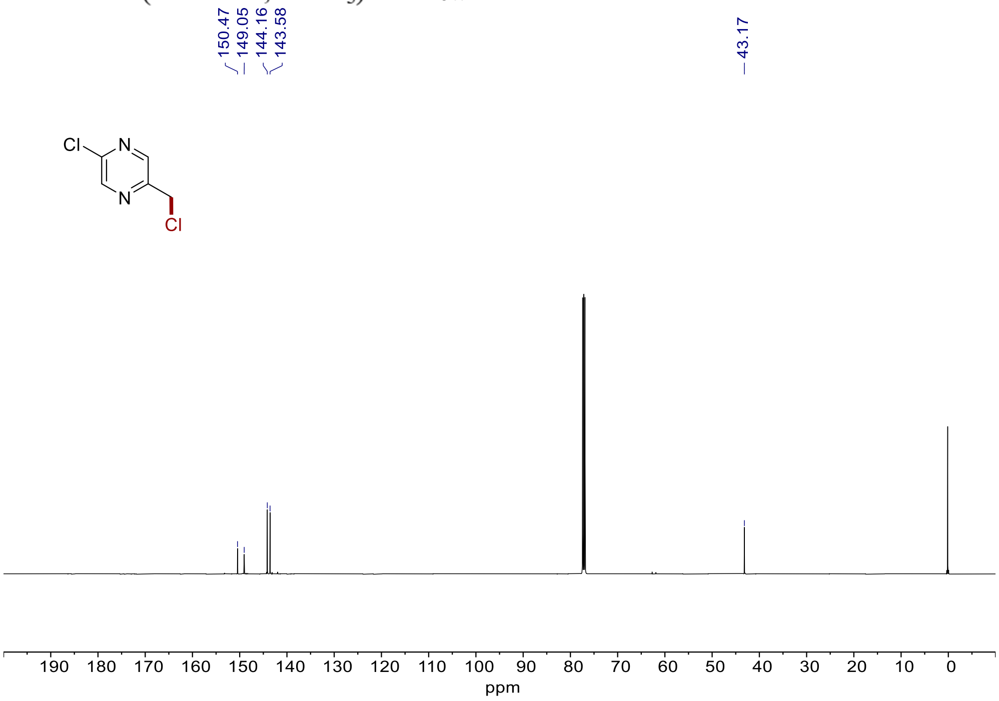

^{1}H NMR (500 MHz, $CDCl_3$) of **19a**

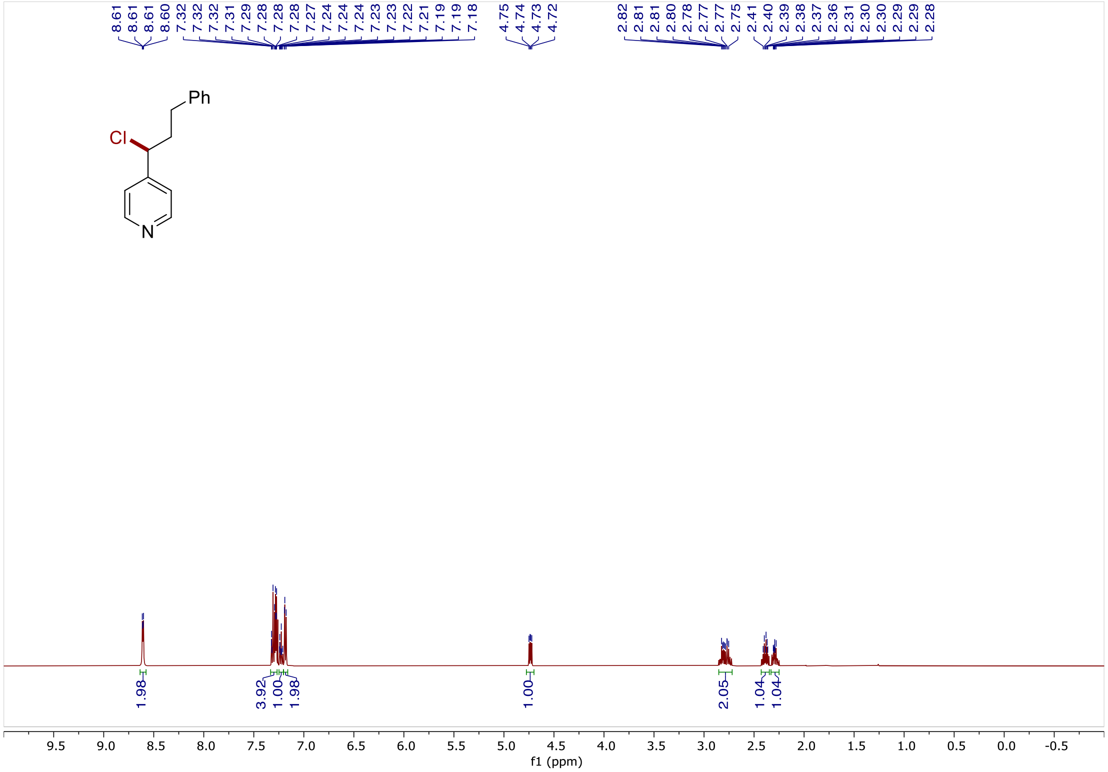

^{13}C NMR: (126 MHz, $CDCl_3$) of **19a**

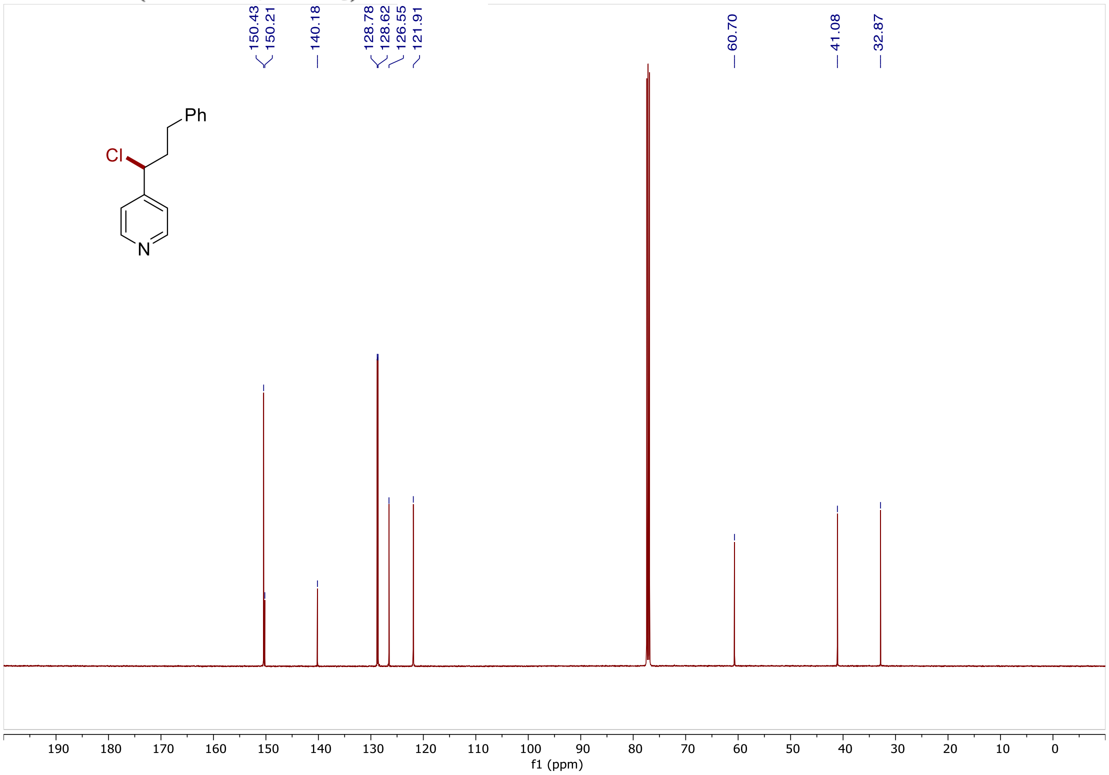

^{1}H NMR (500 MHz, $CDCl_3$) of **20a**

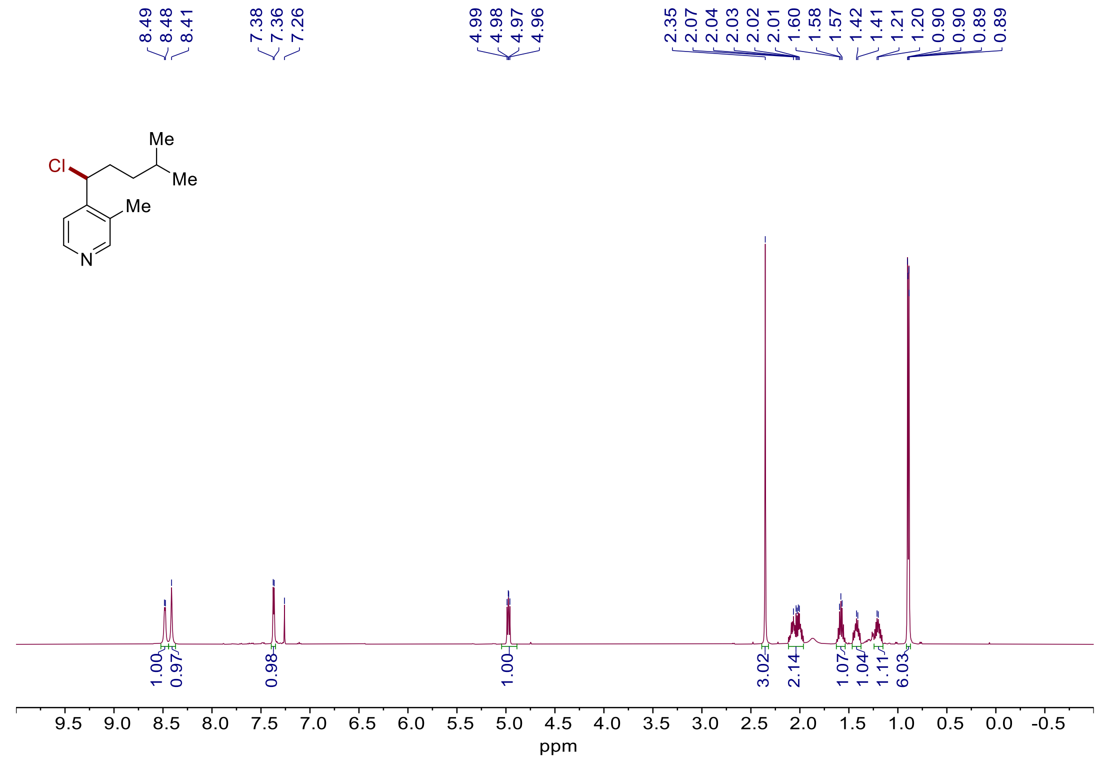

^{13}C NMR: (126 MHz, $CDCl_3$) of **20a**

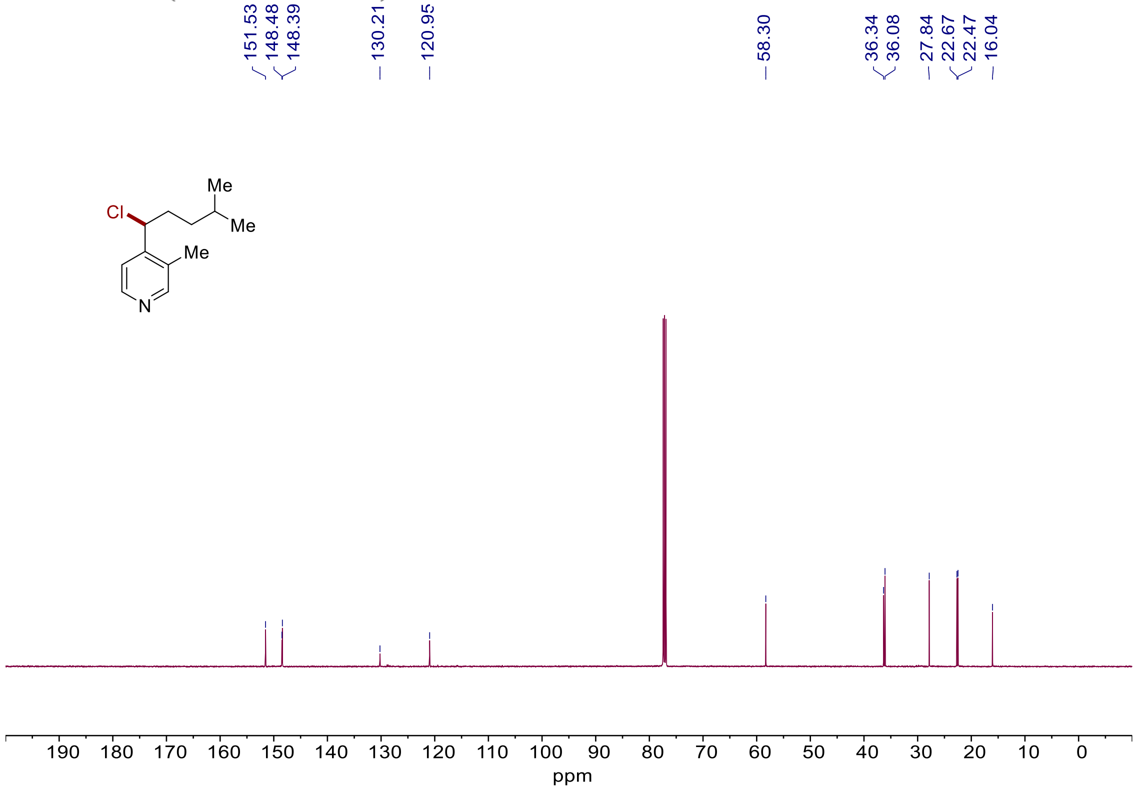

^{1}H NMR (500 MHz, $CDCl_3$) of **21a**

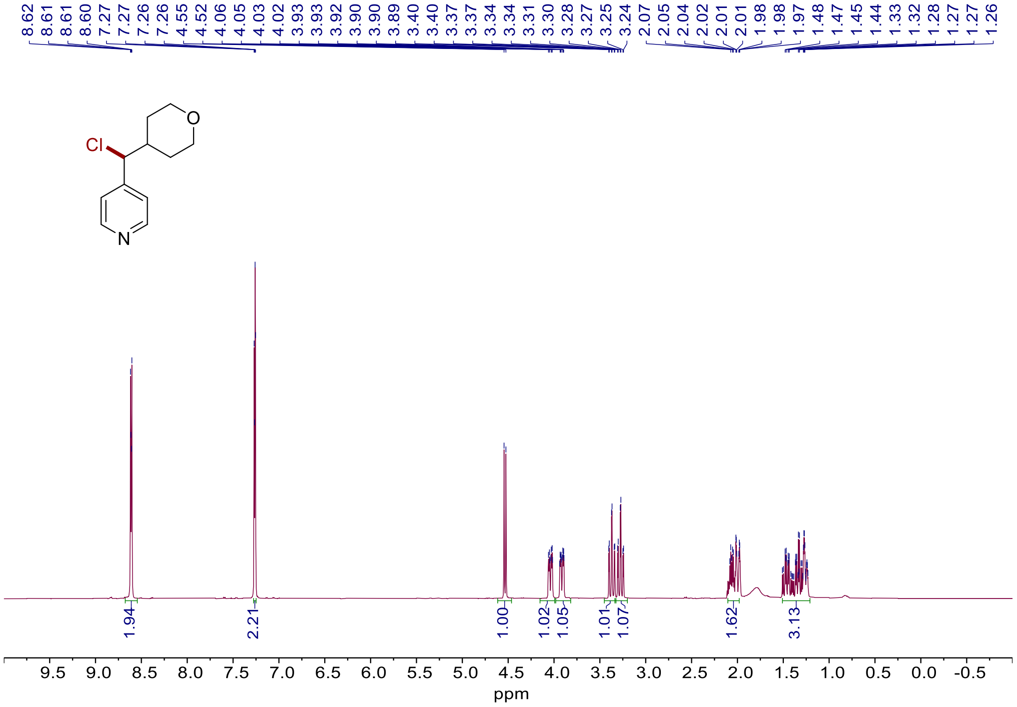

^{13}C NMR: (126 MHz, $CDCl_3$) of **21a**

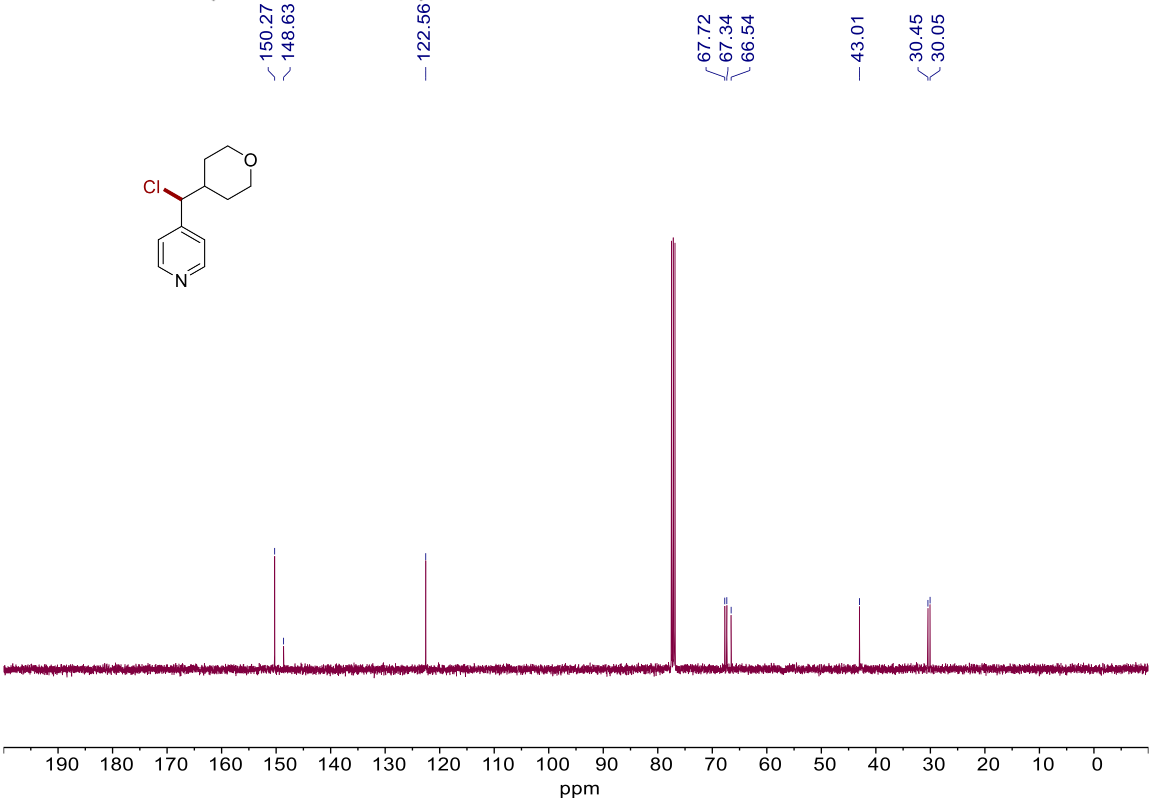

^{1}H NMR (500 MHz, $CDCl_3$) of **23a**

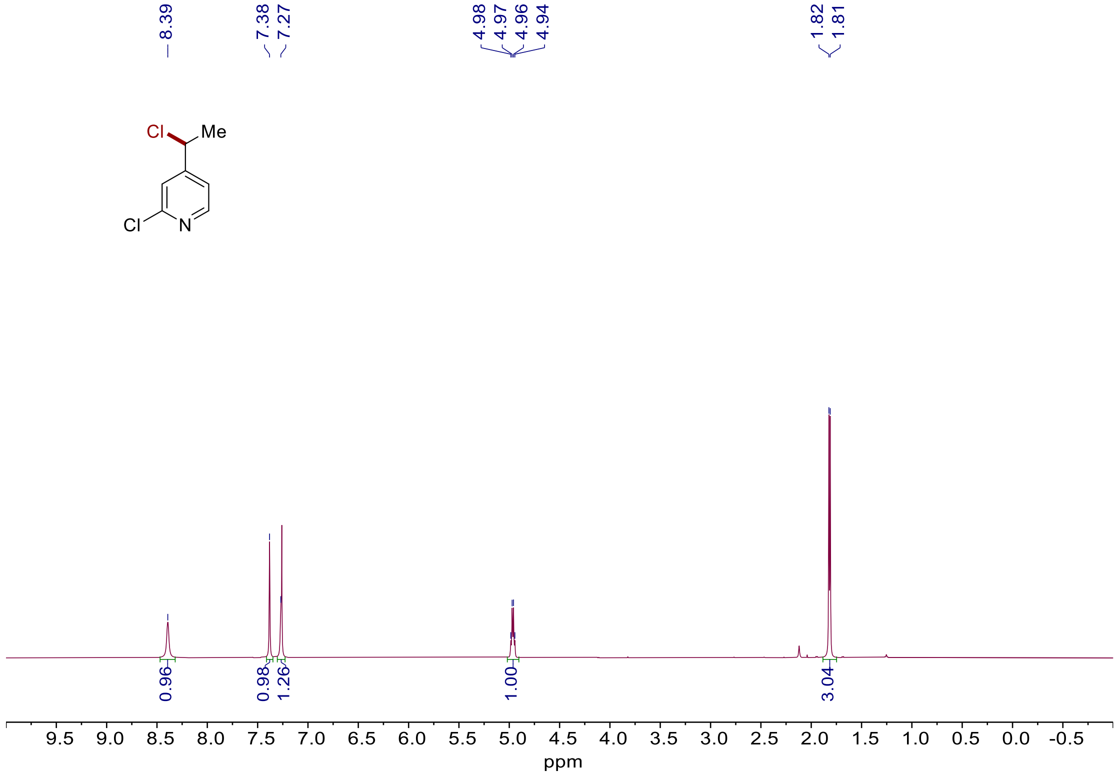

^{13}C NMR: (126 MHz, $CDCl_3$) of **23a**

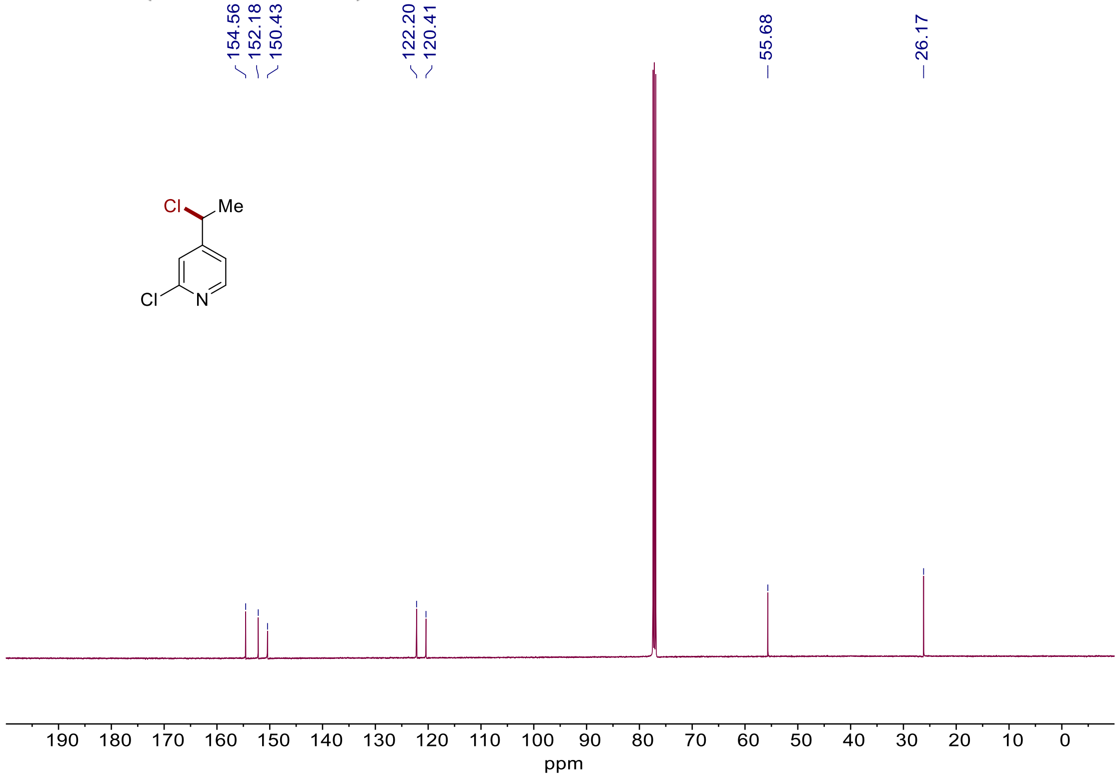

^{1}H NMR (500 MHz, $CDCl_3$) of **24a**

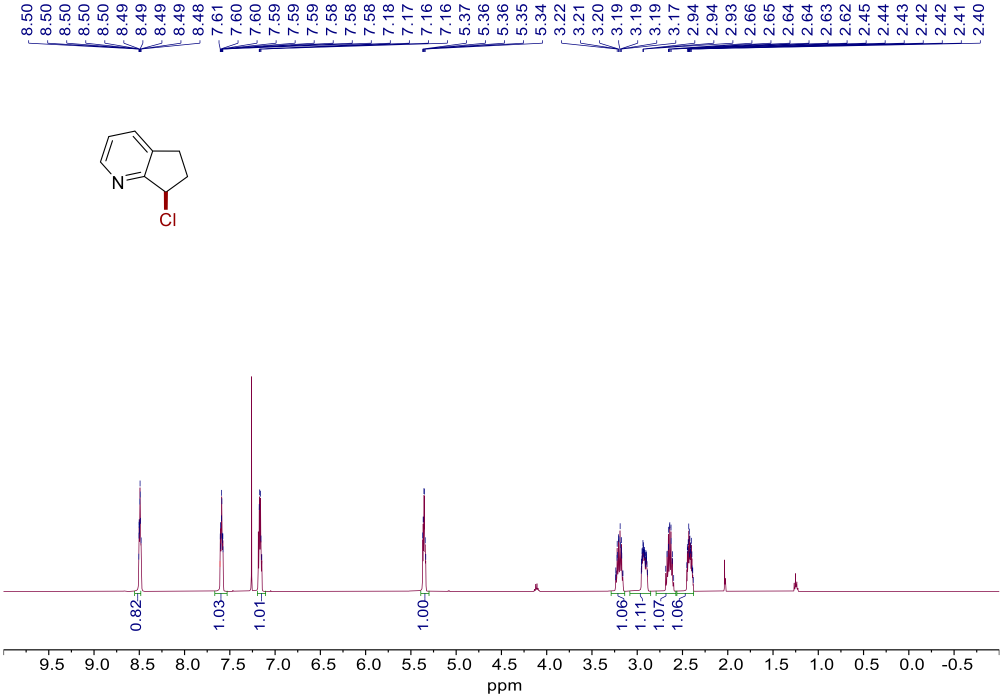

^{13}C NMR: (126 MHz, $CDCl_3$) of **24a**

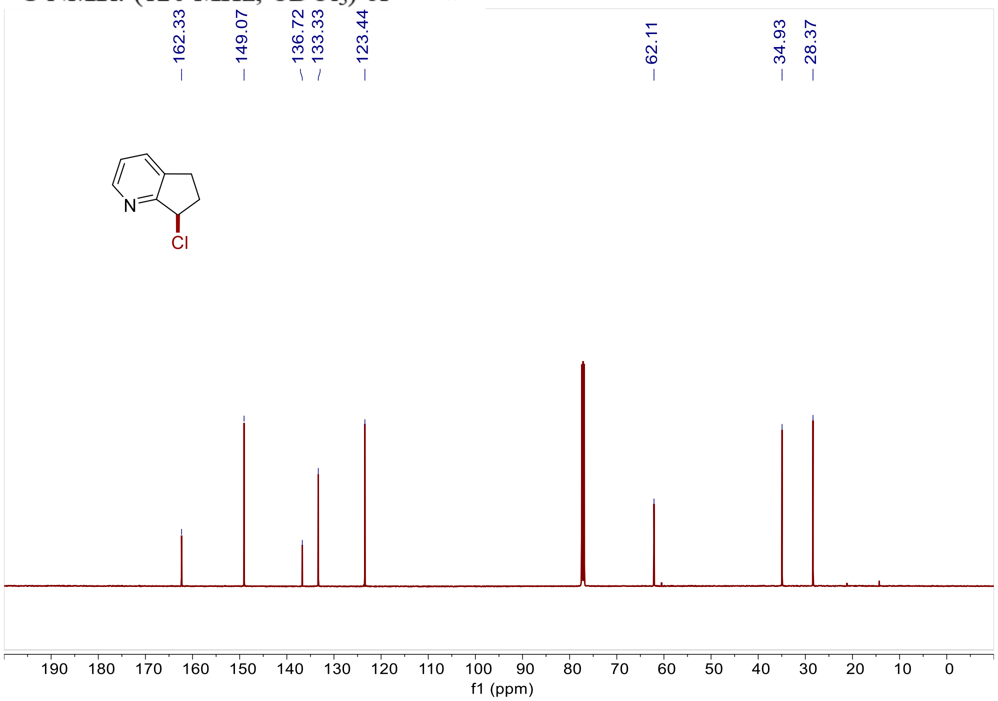

^{1}H NMR (500 MHz, $CDCl_3$) of **25a**

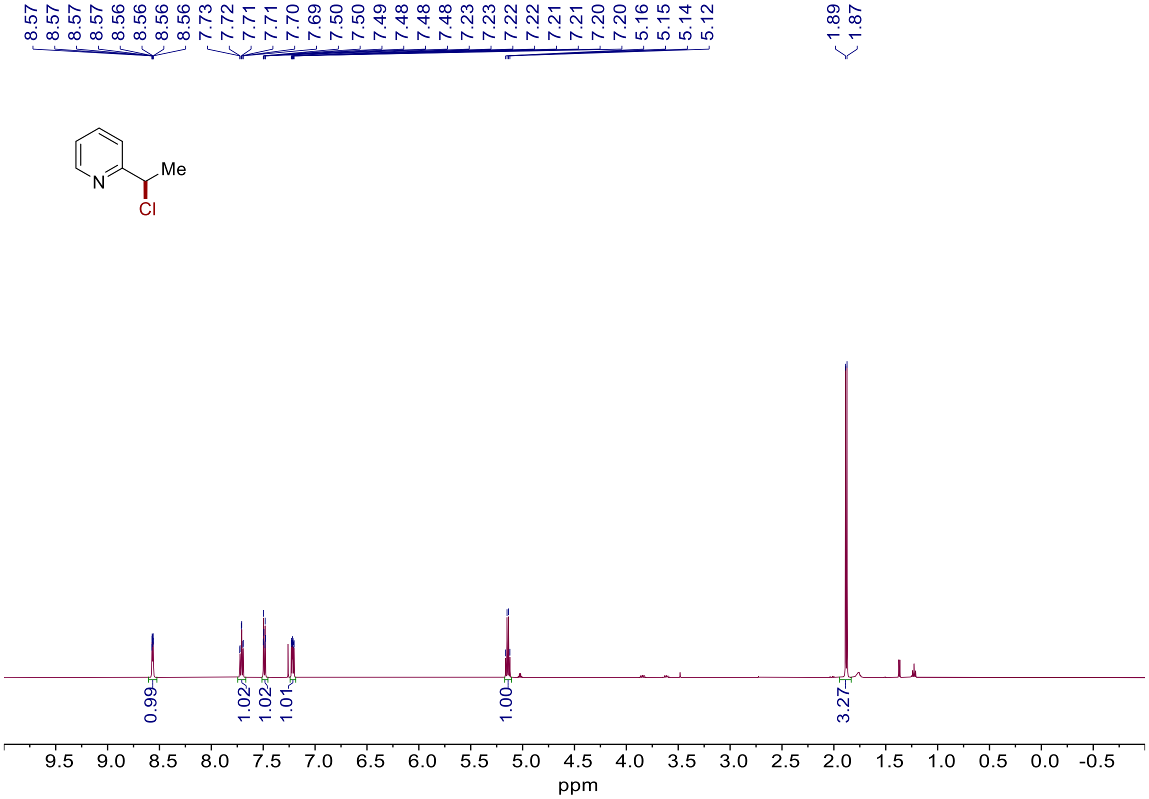

^{13}C NMR: (126 MHz, $CDCl_3$) of **25a**

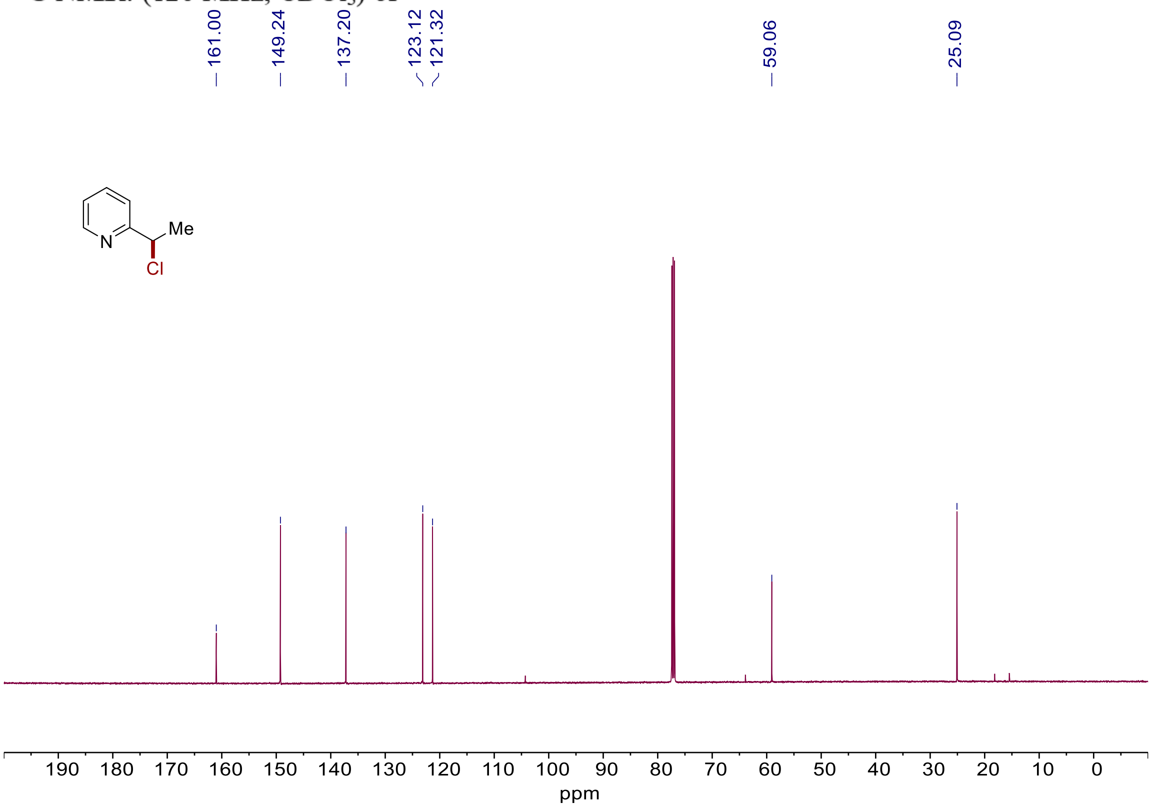

^{1}H NMR (500 MHz, $CDCl_3$) of **27a**

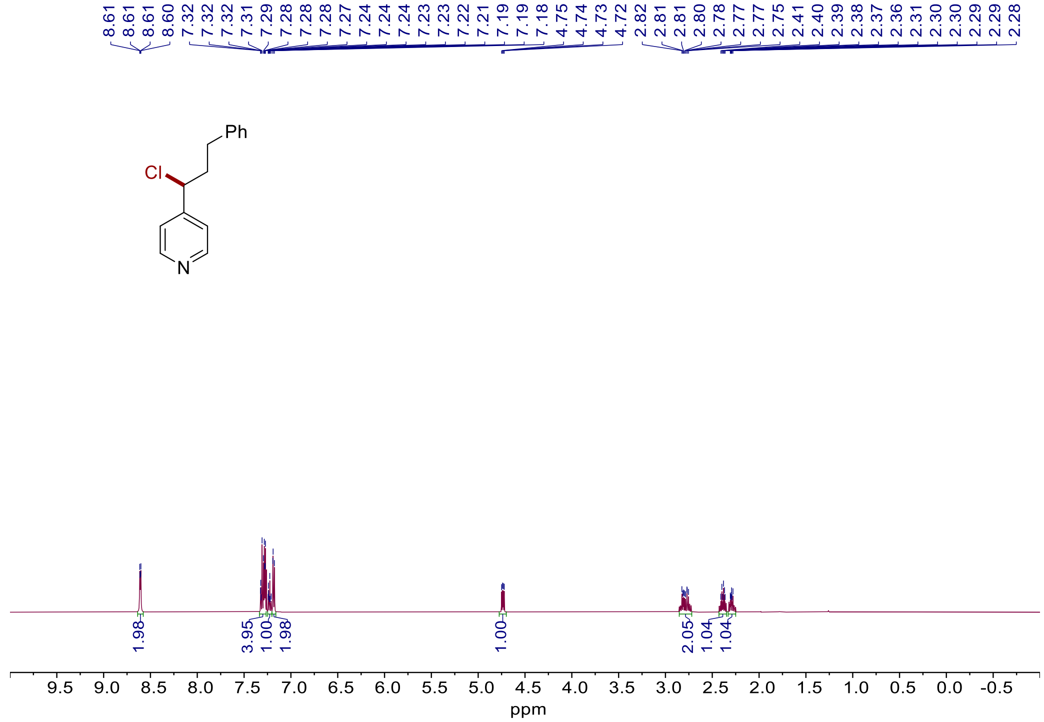

^{13}C NMR: (126 MHz, $CDCl_3$) of **27a**

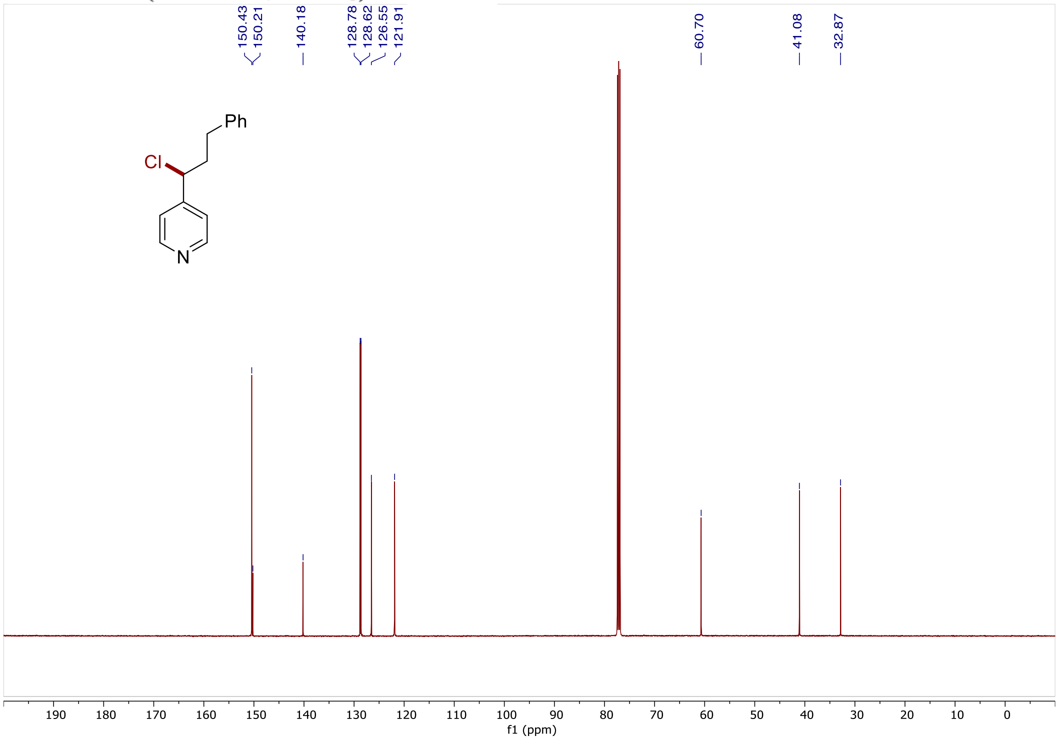

^{1}H NMR (500 MHz, $CDCl_3$) of **29a**

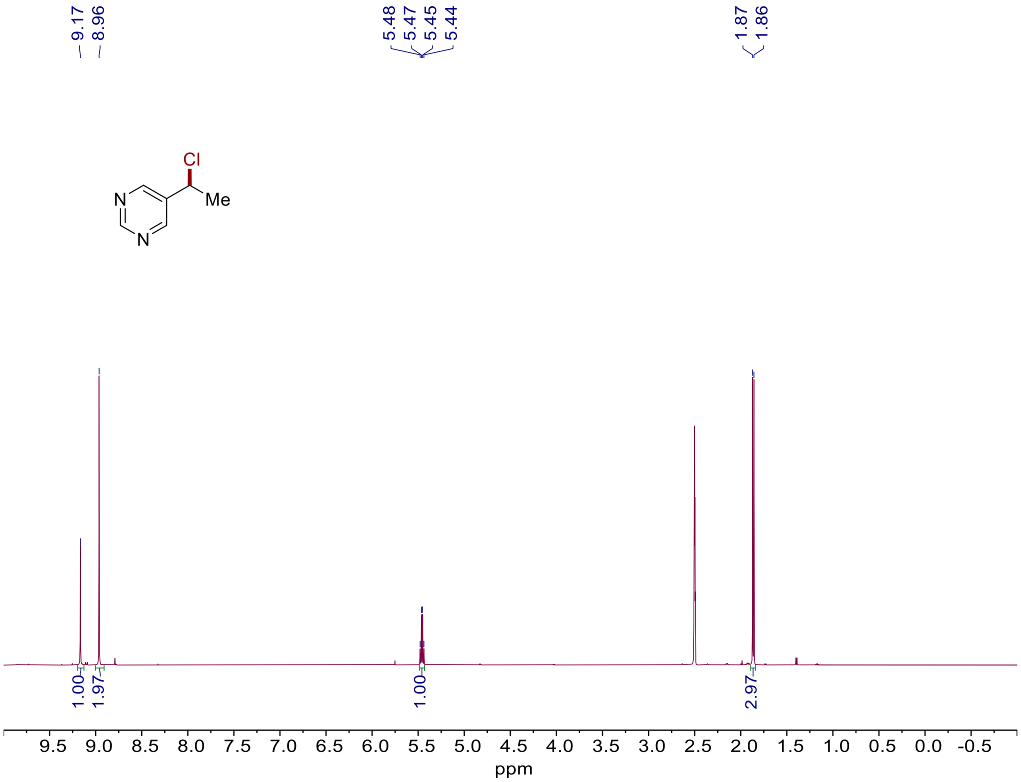

^{13}C NMR: (126 MHz, $CDCl_3$) of **29a**

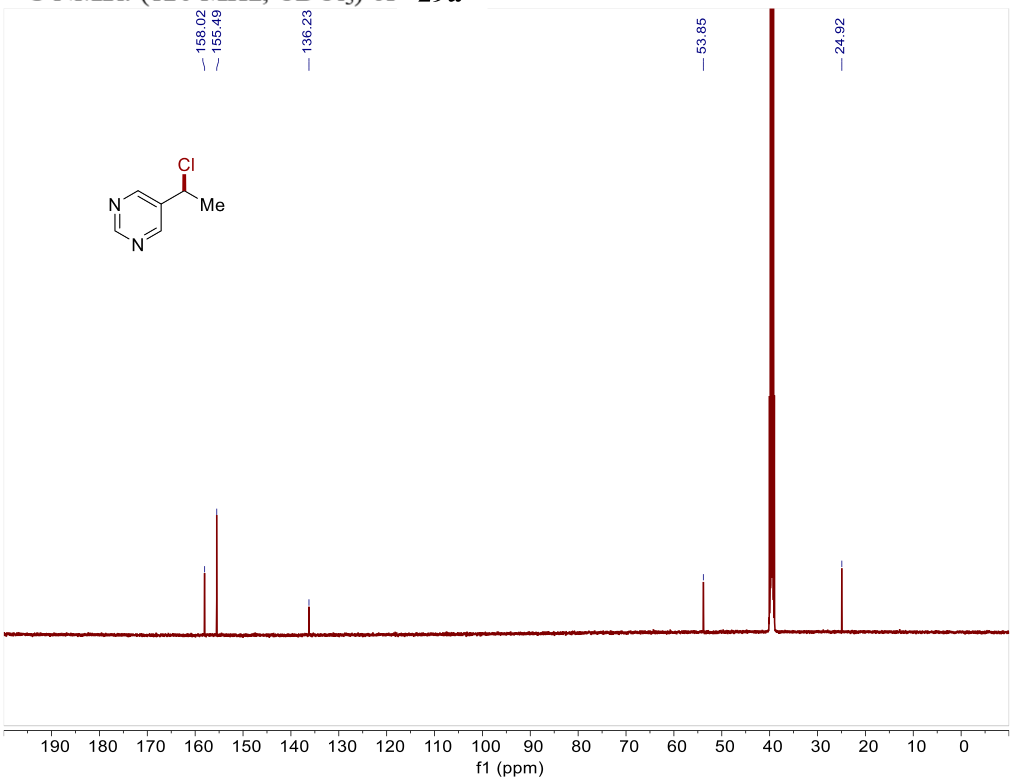

^{1}H NMR (500 MHz, $CDCl_3$) of **1**

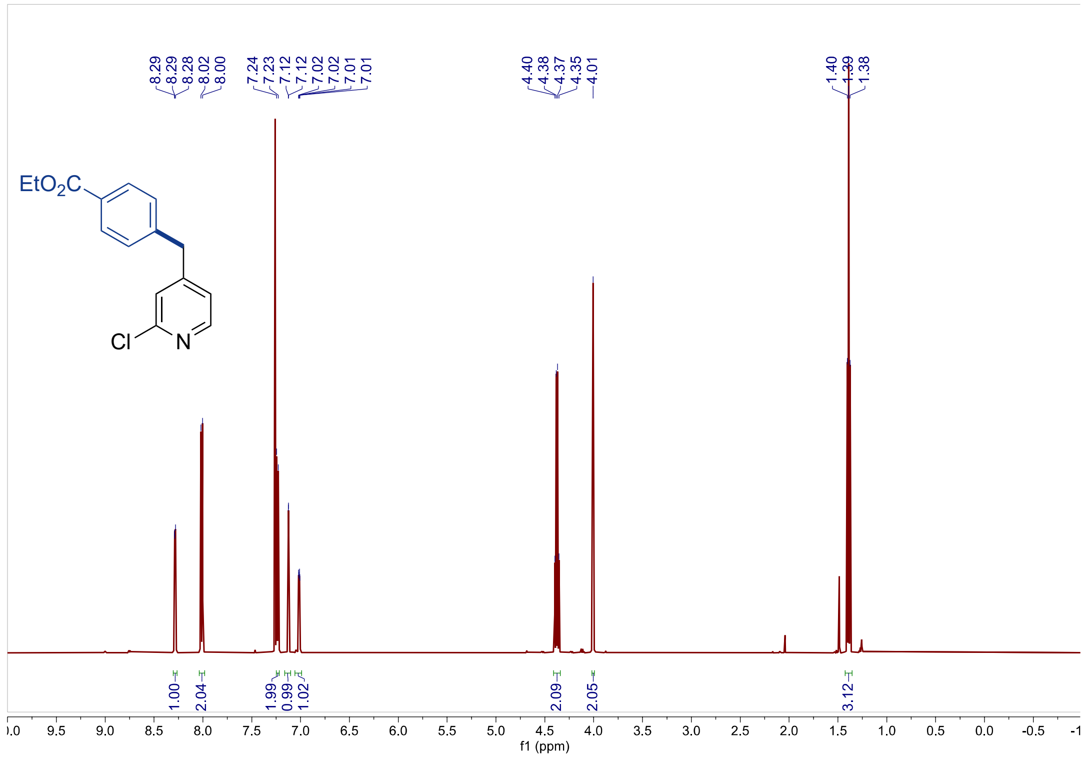

^{13}C NMR: (126 MHz, $CDCl_3$) of **1**

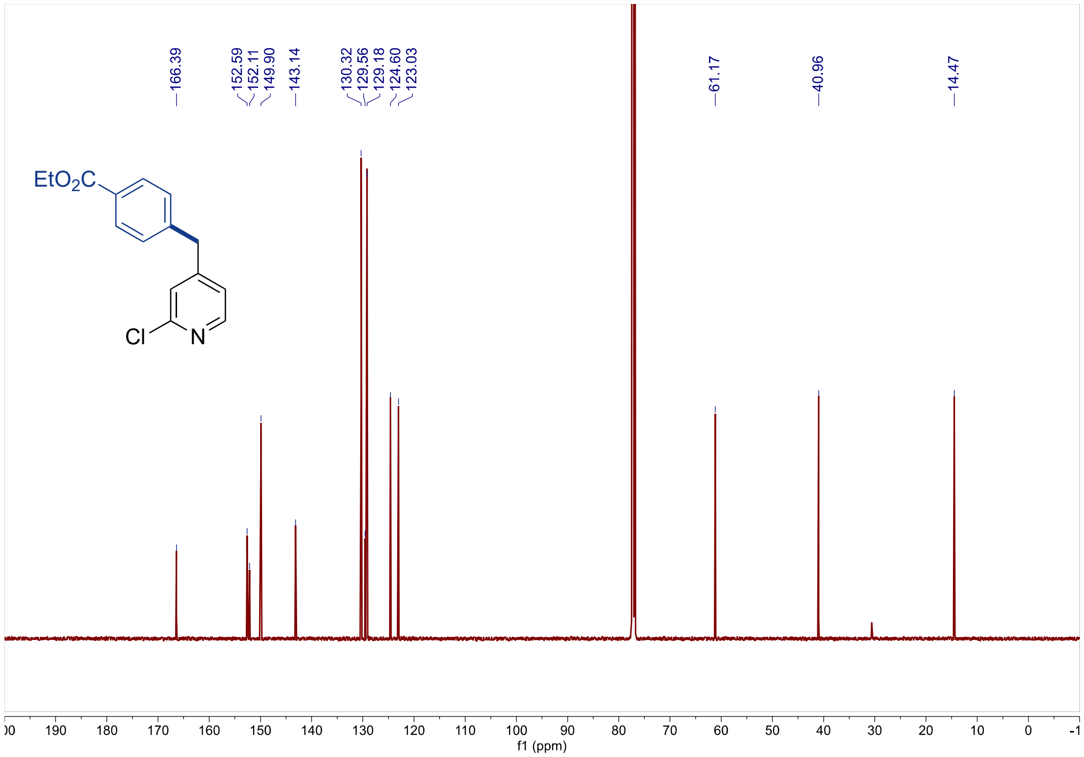

^{1}H NMR (500 MHz, $CDCl_3$) of **2**

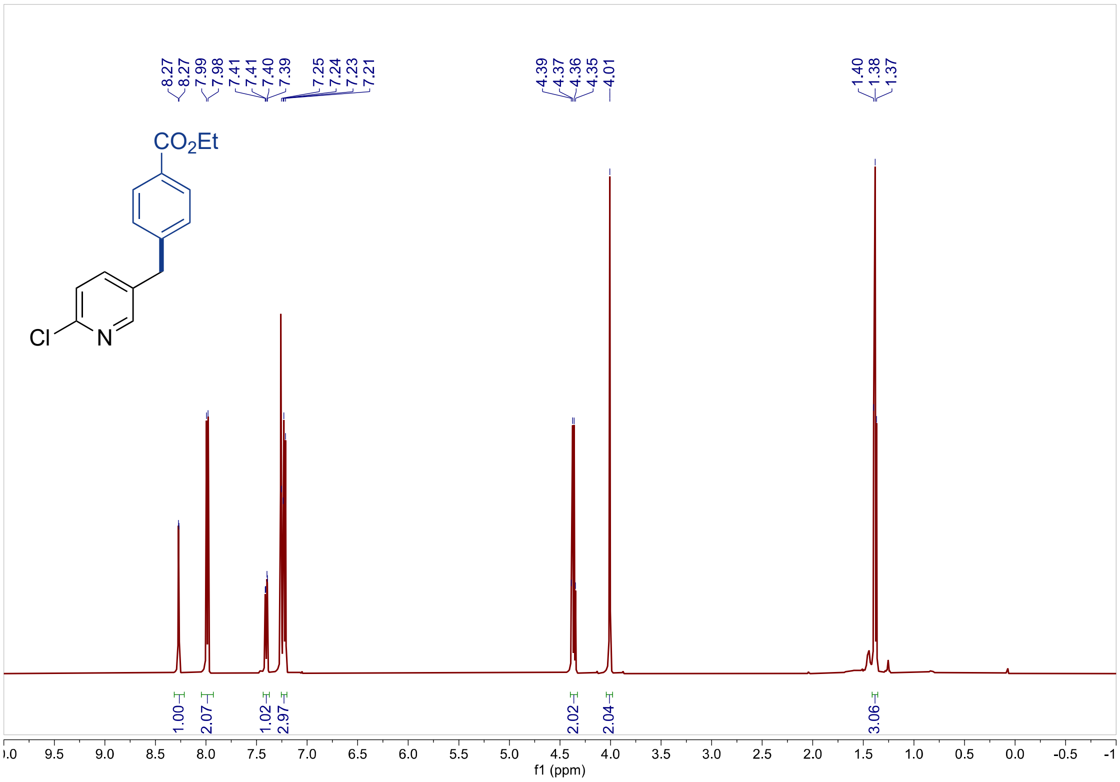

^{13}C NMR: (126 MHz, $CDCl_3$) of **2**

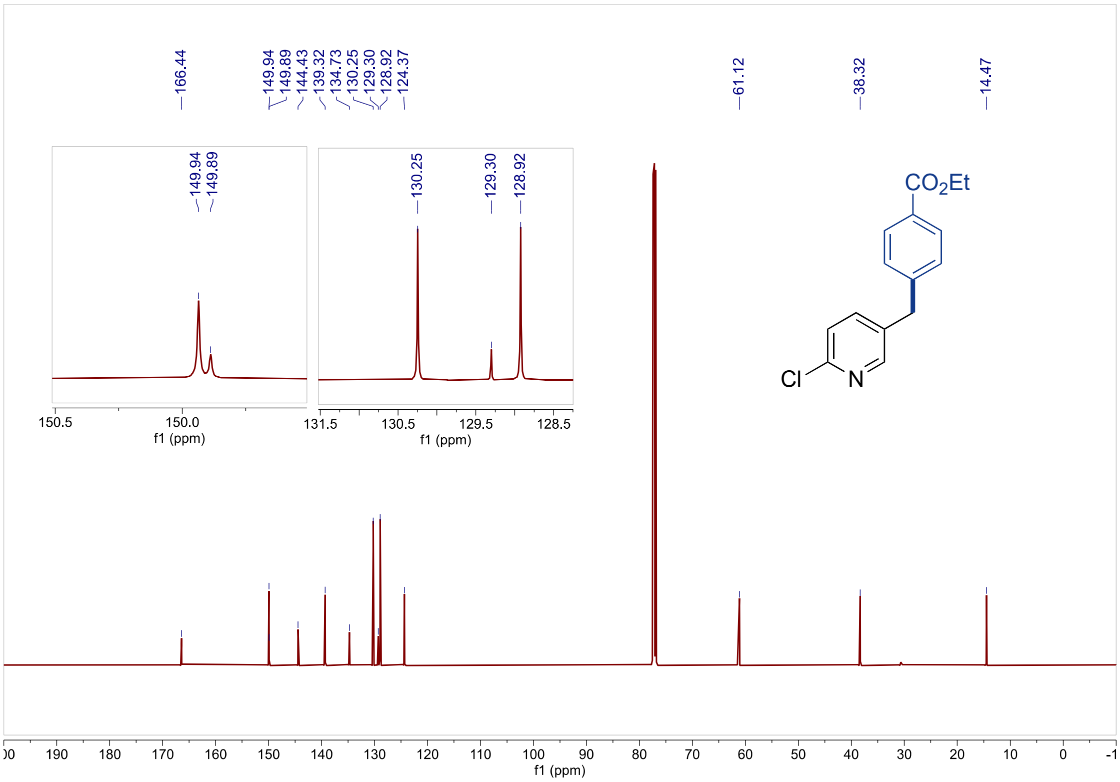

^{1}H NMR (500 MHz, $CDCl_3$) of **3**

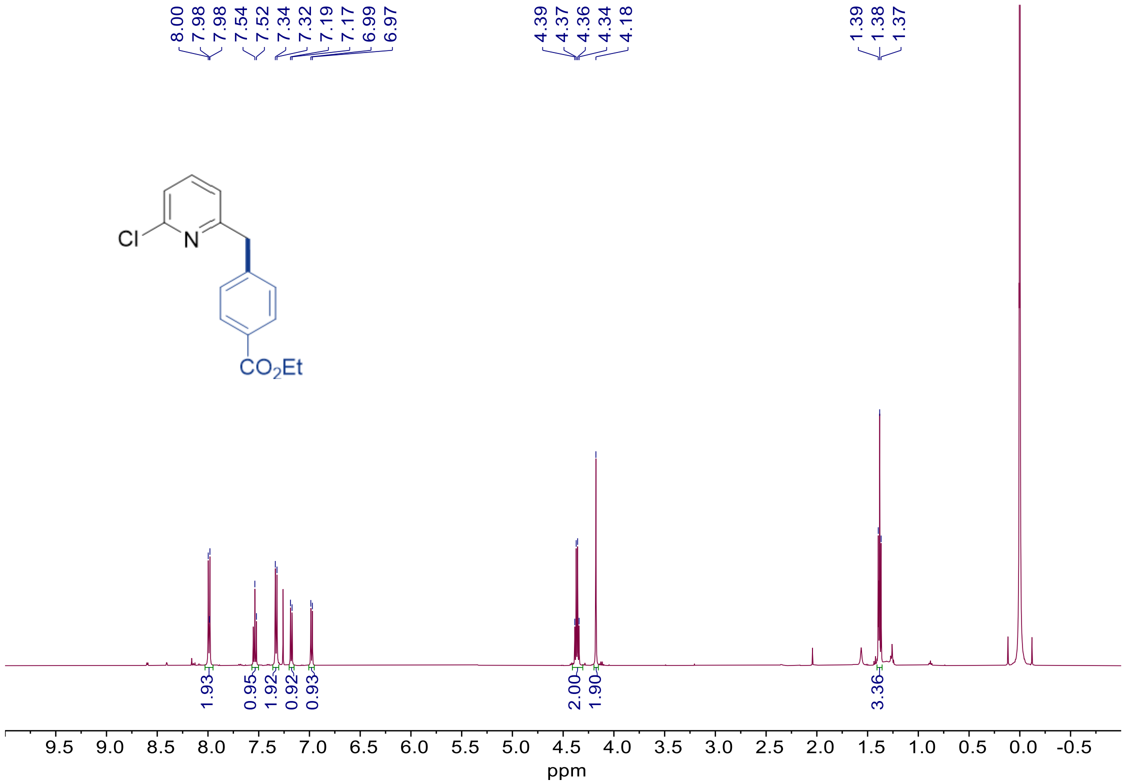

^{13}C NMR: (126 MHz, $CDCl_3$) of **3**

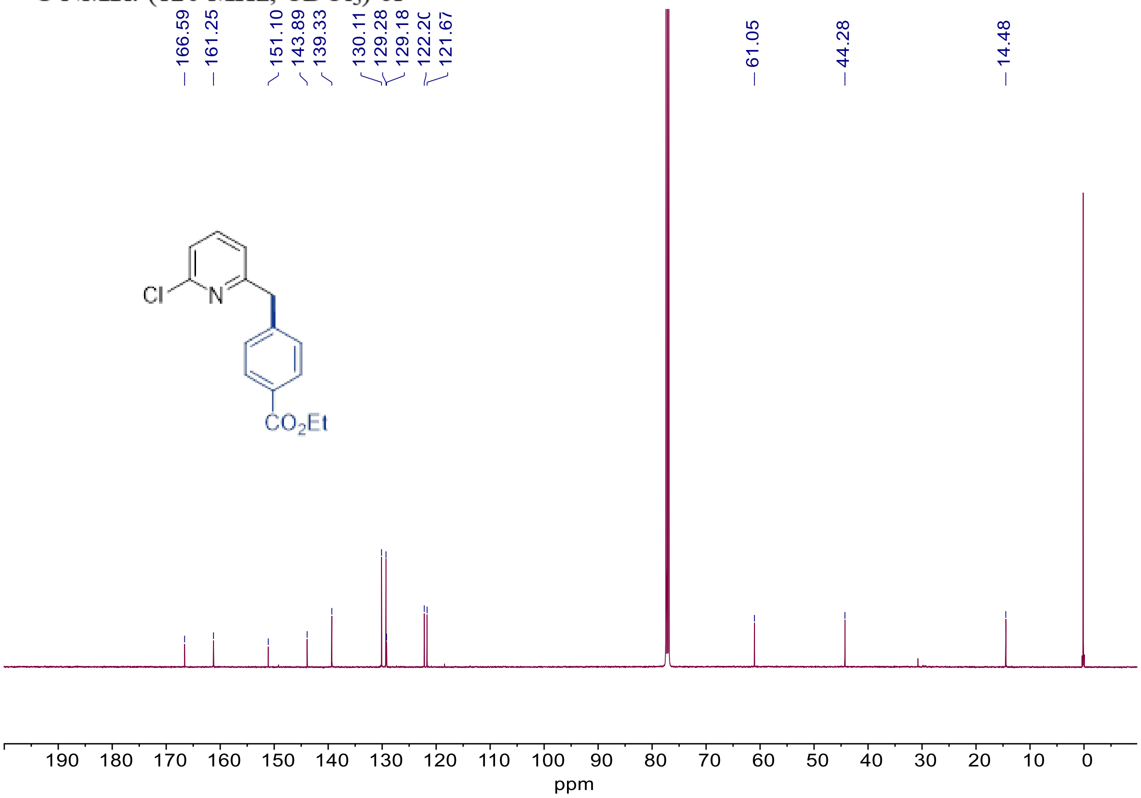

^{1}H NMR (500 MHz, $CDCl_3$) of **4**

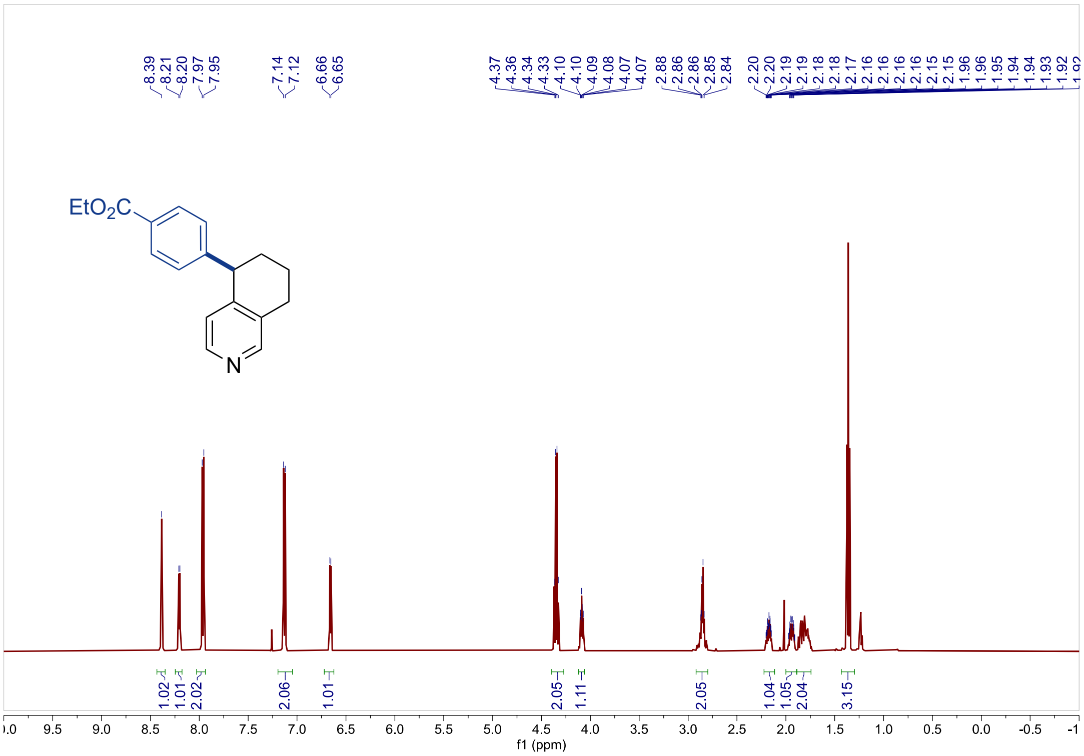

^{13}C NMR: (126 MHz, $CDCl_3$) of **4**

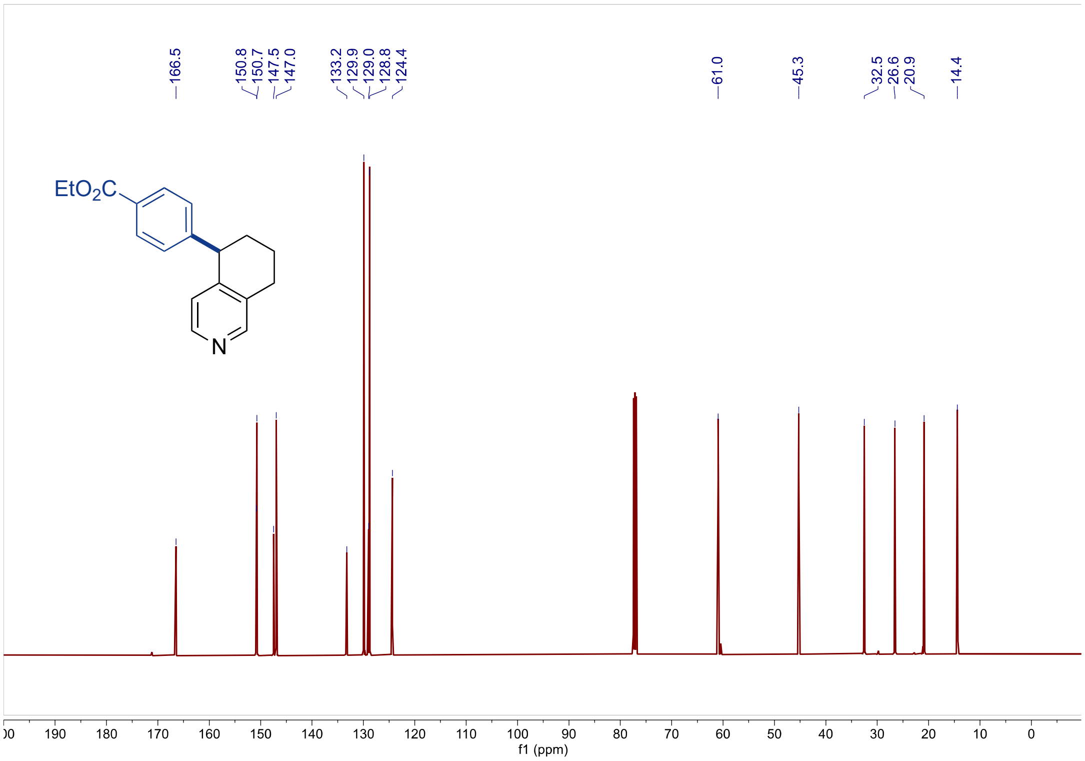

^{1}H NMR (500 MHz, $CDCl_3$) of **5**

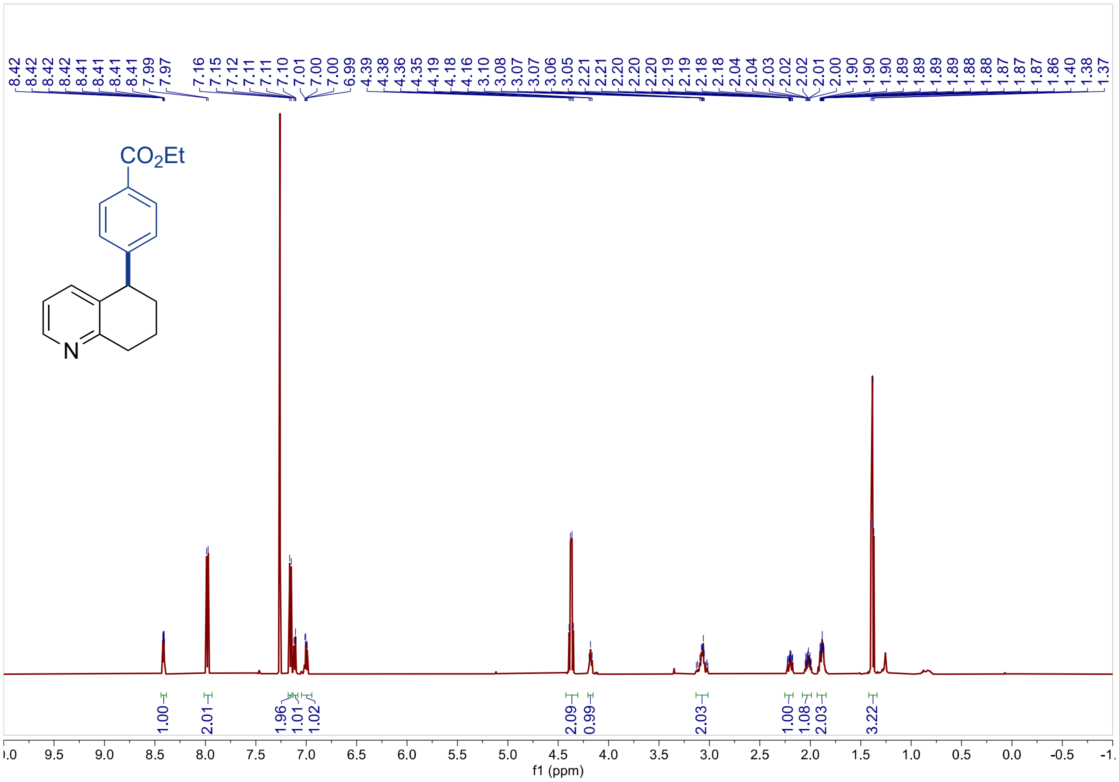

^{13}C NMR: (126 MHz, $CDCl_3$) of **5**

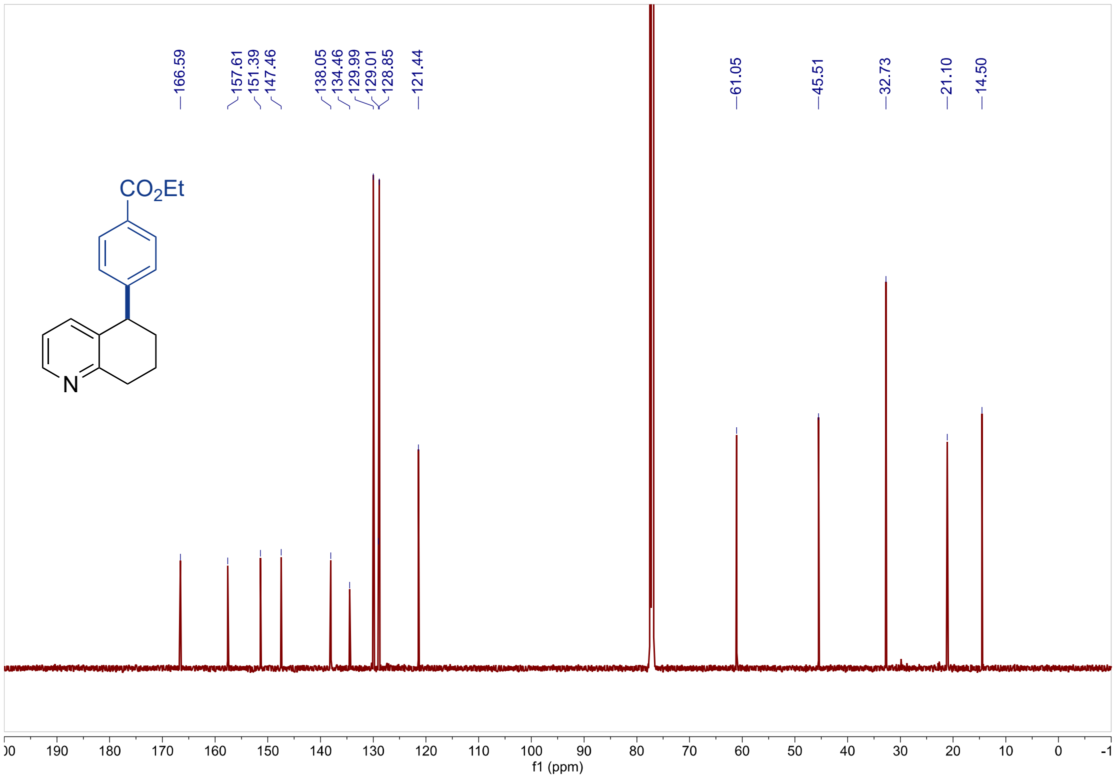

^{1}H NMR (500 MHz, $CDCl_3$) of **6**

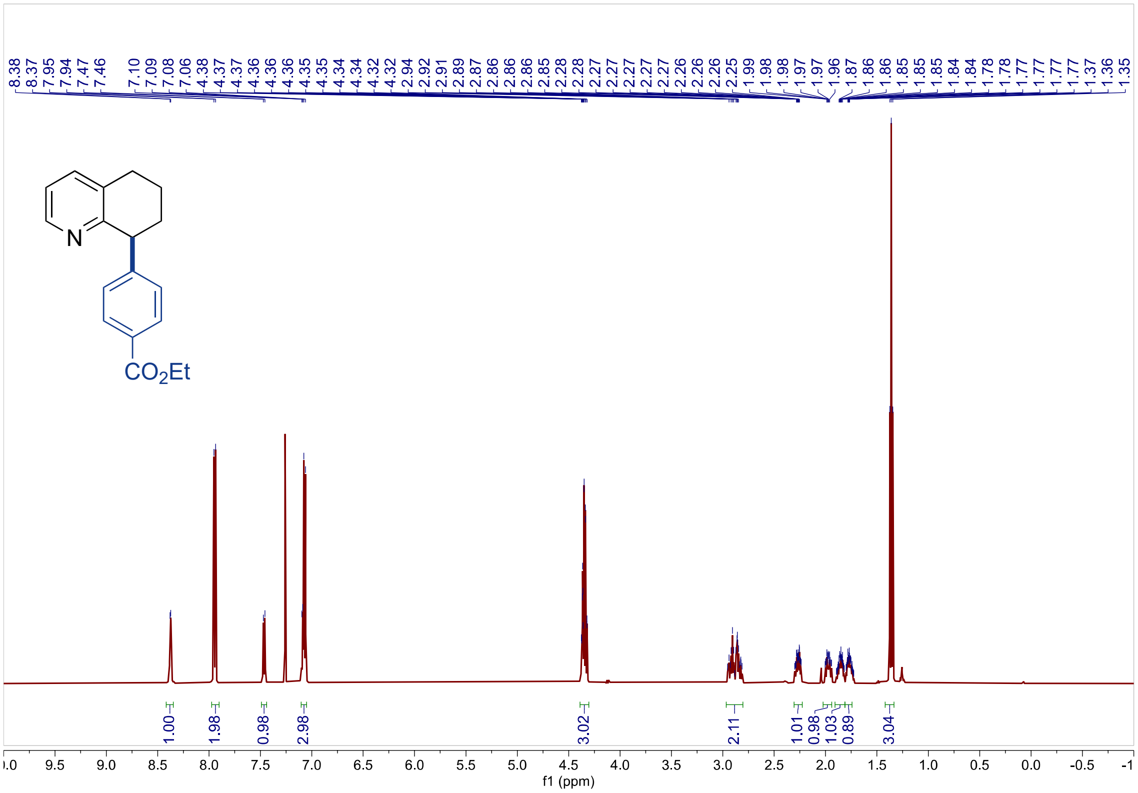

^{13}C NMR: (126 MHz, $CDCl_3$) of **6**

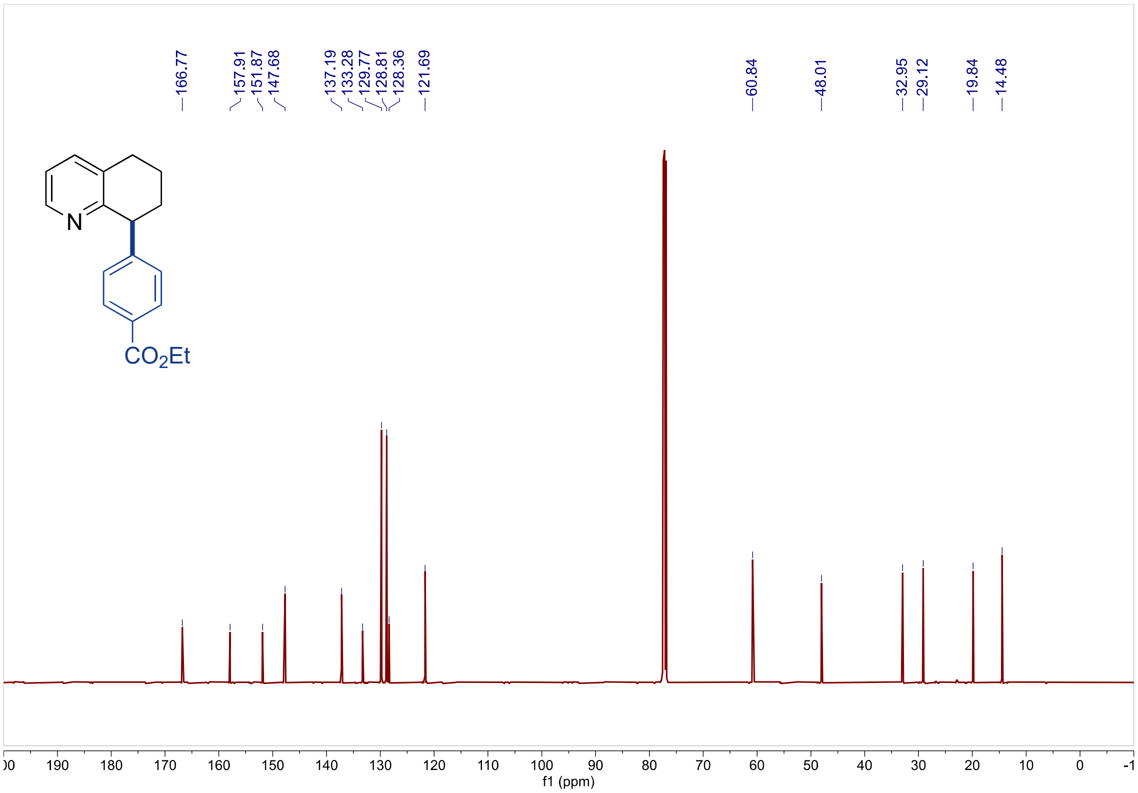

^{1}H NMR (500 MHz, $CDCl_3$) of **7**

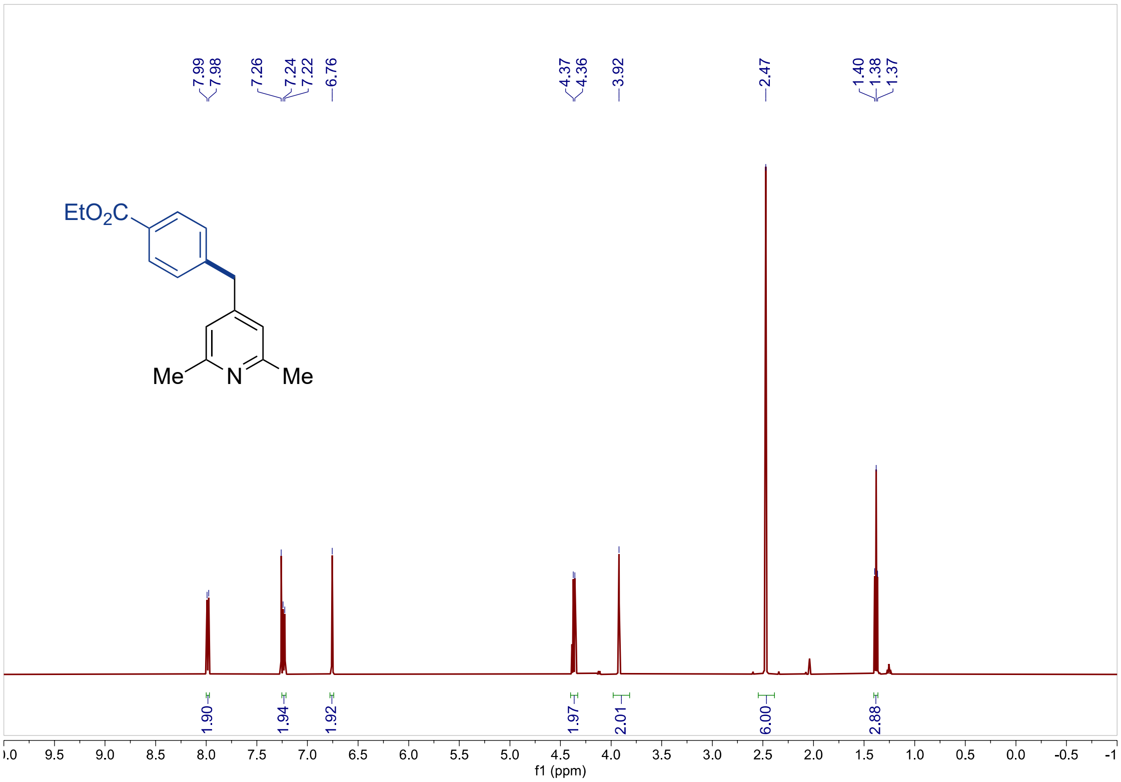

^{13}C NMR: (126 MHz, $CDCl_3$) of **7**

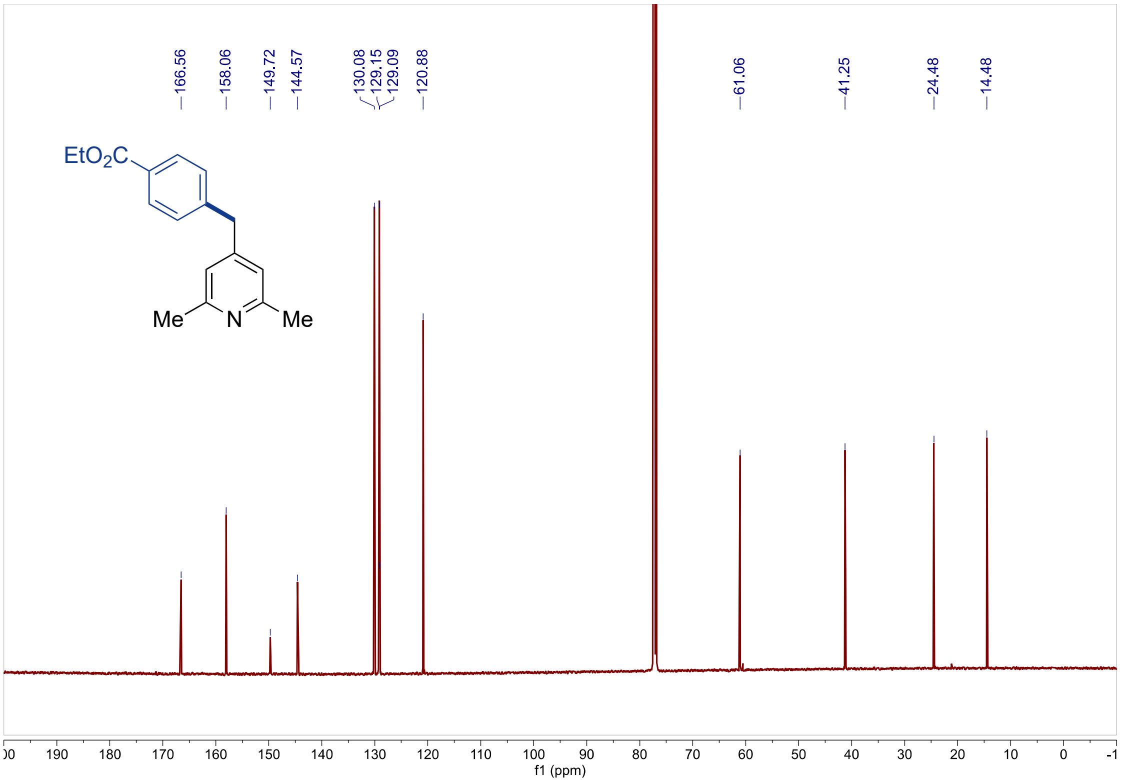

^{1}H NMR (500 MHz, $CDCl_3$) of **8**

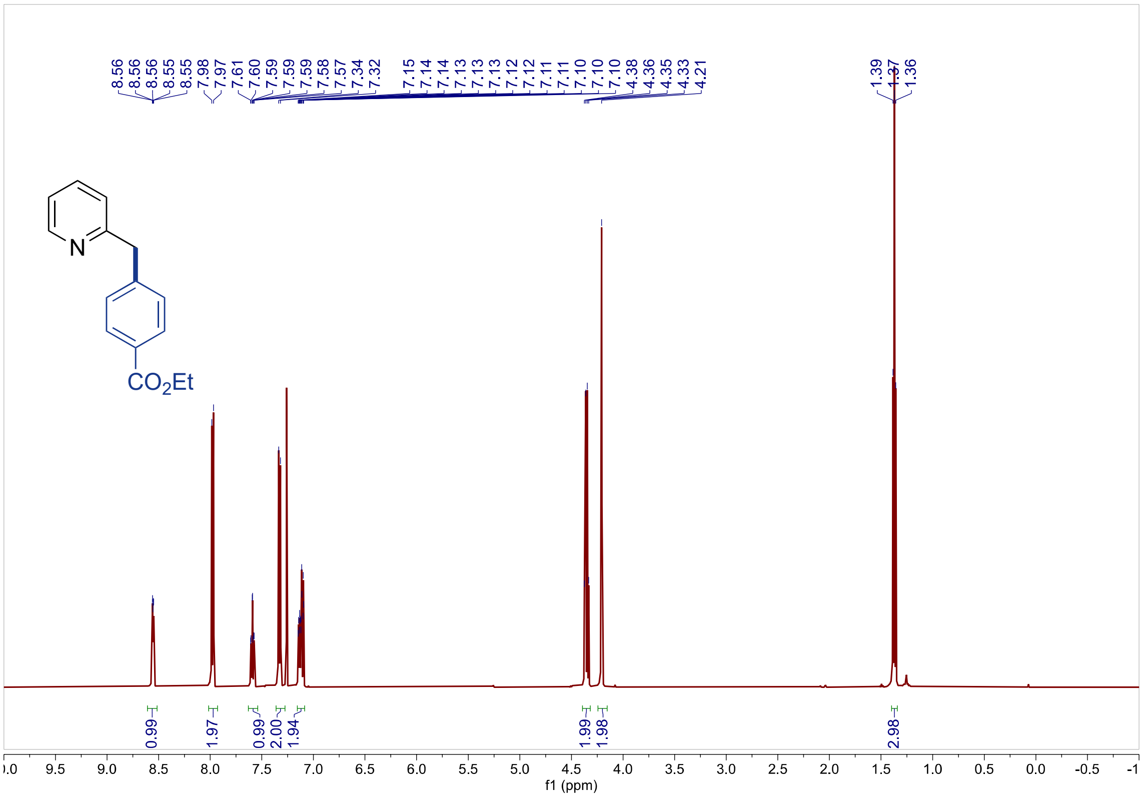

^{13}C NMR: (126 MHz, $CDCl_3$) of **8**

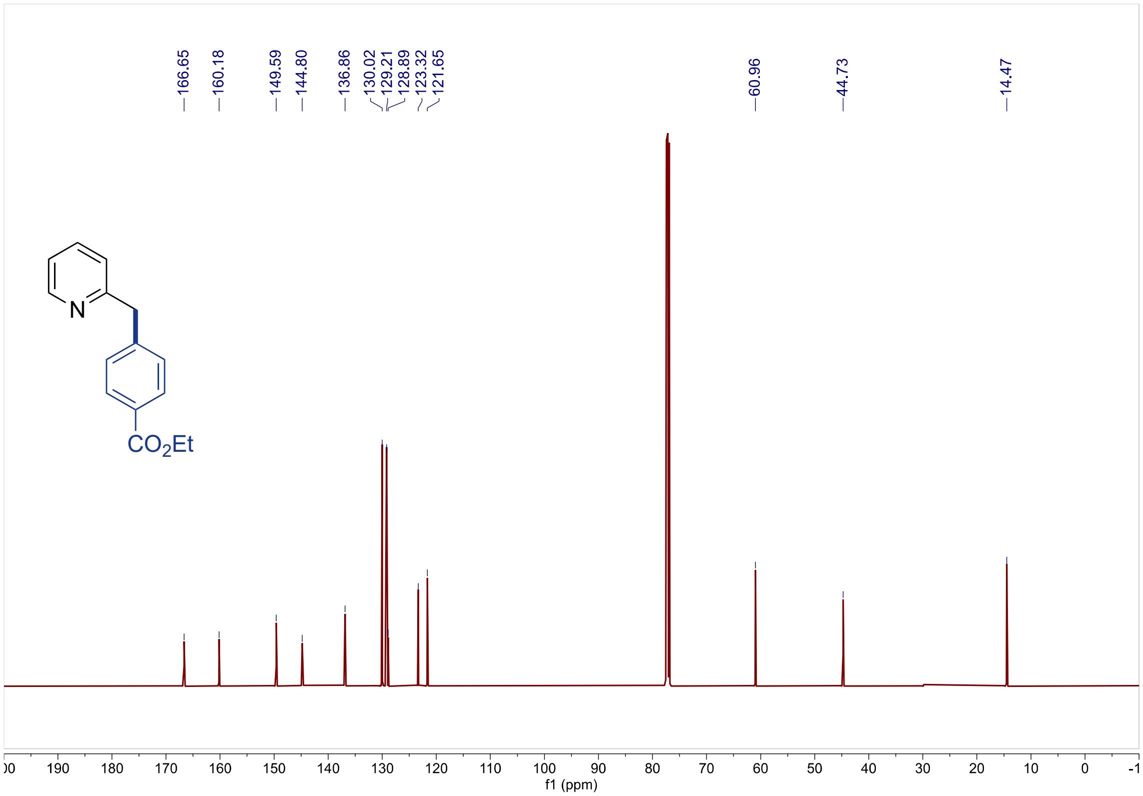

^{1}H NMR (500 MHz, $CDCl_3$) of **9**

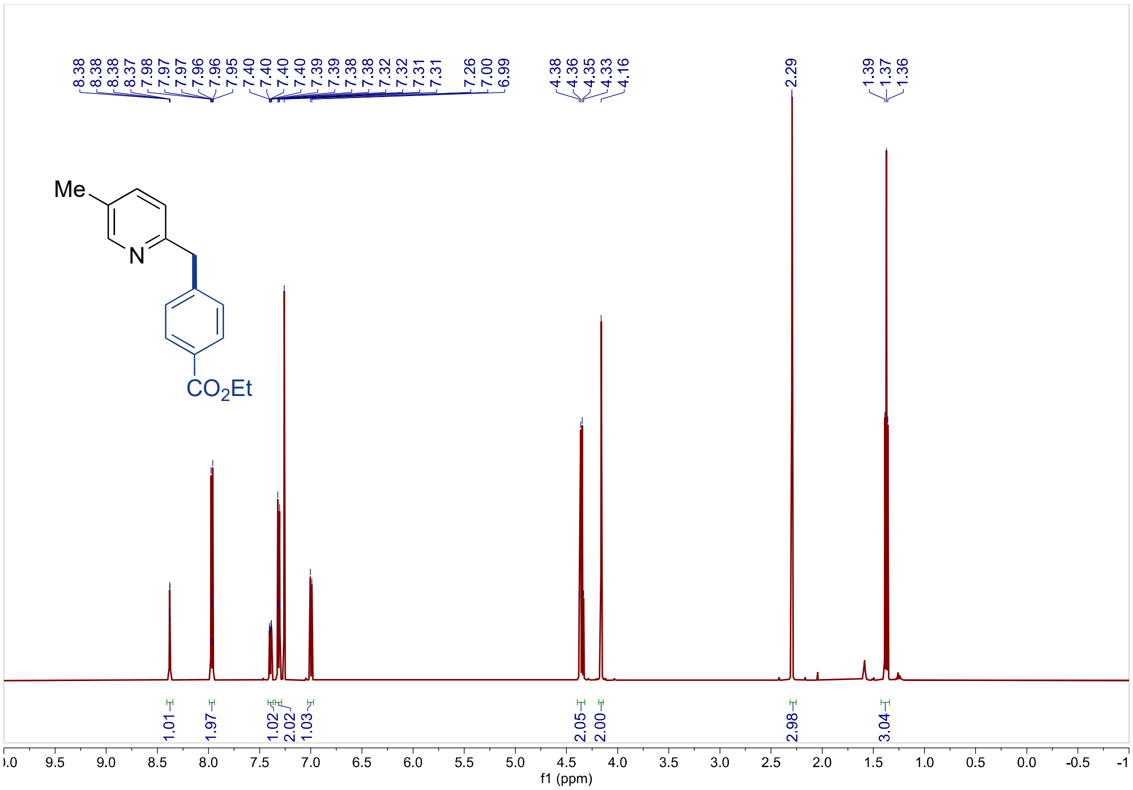

^{13}C NMR: (126 MHz, $CDCl_3$) of **9**

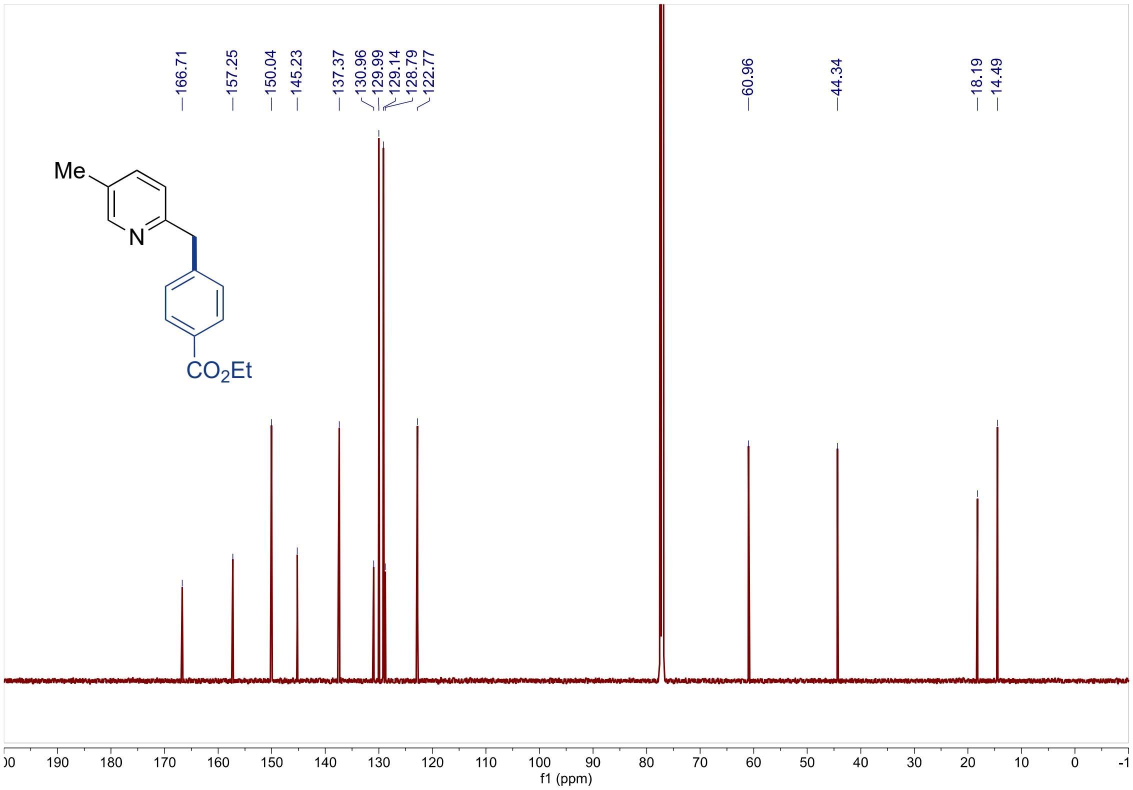

^{1}**H NMR** (500 MHz, $CDCl_3$) of **10**

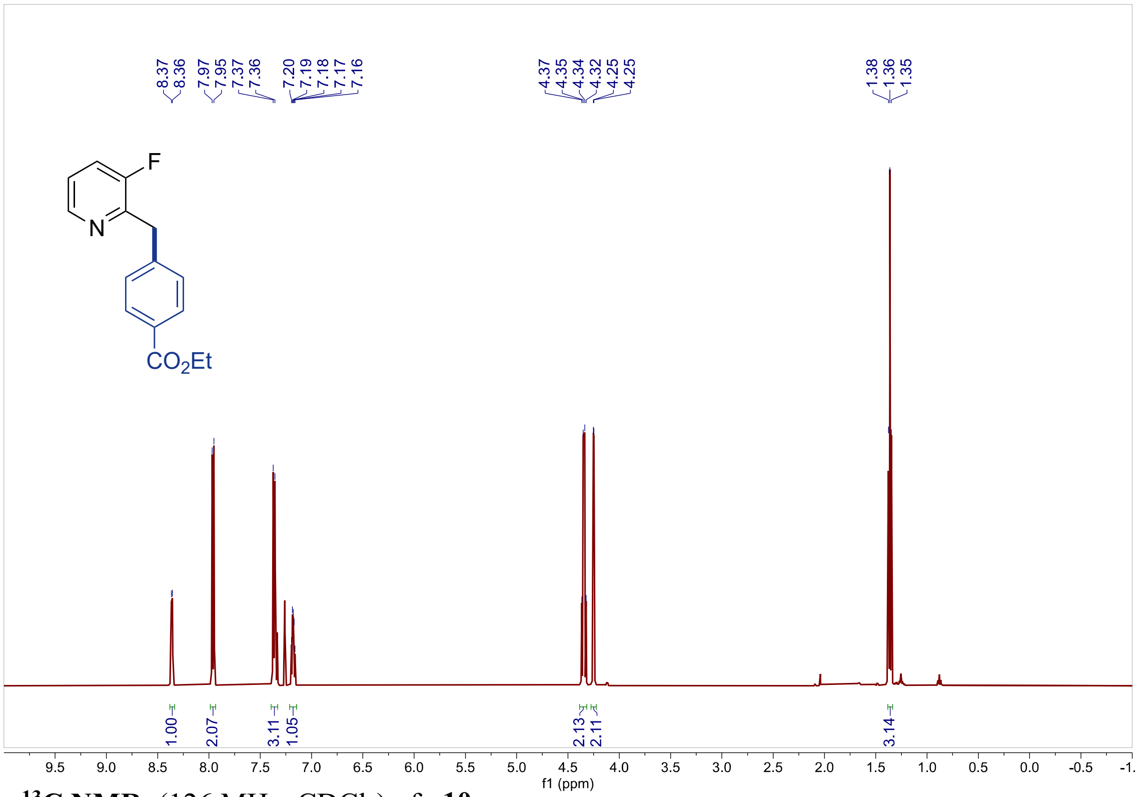

^{13}**C NMR**: (126 MHz, $CDCl_3$) of **10**

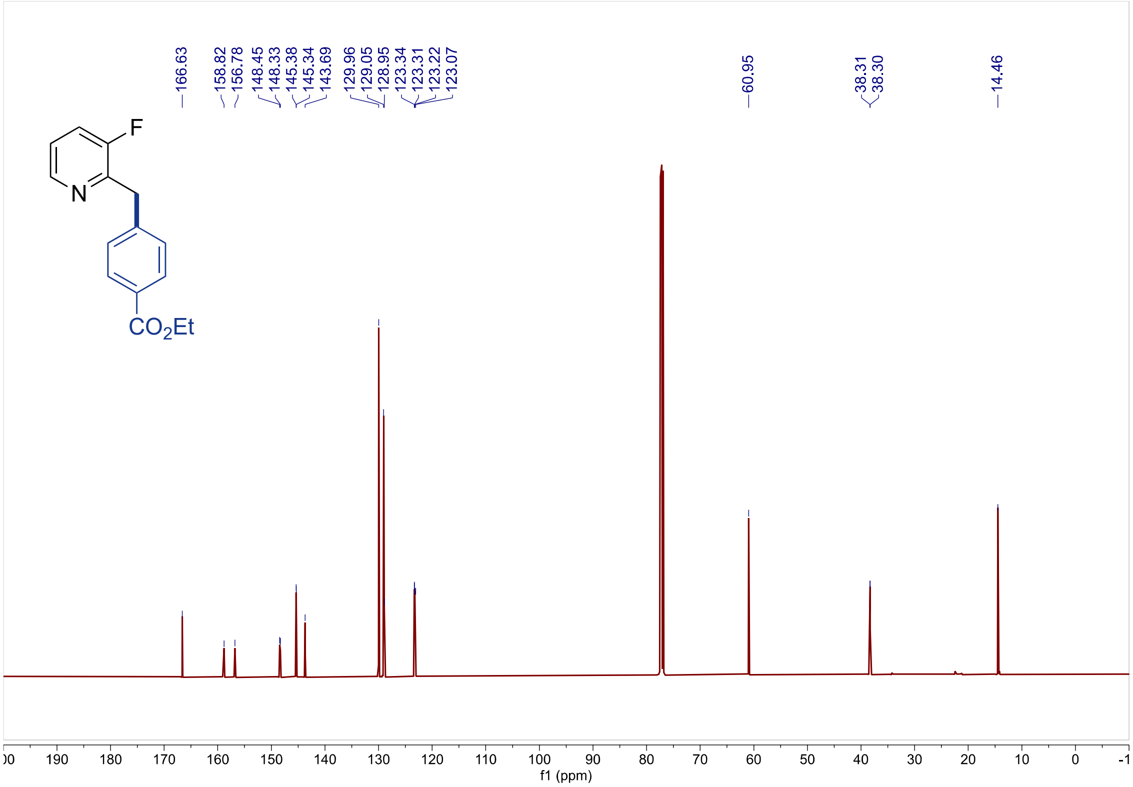

^{1}H NMR (500 MHz, $CDCl_3$) of **10**

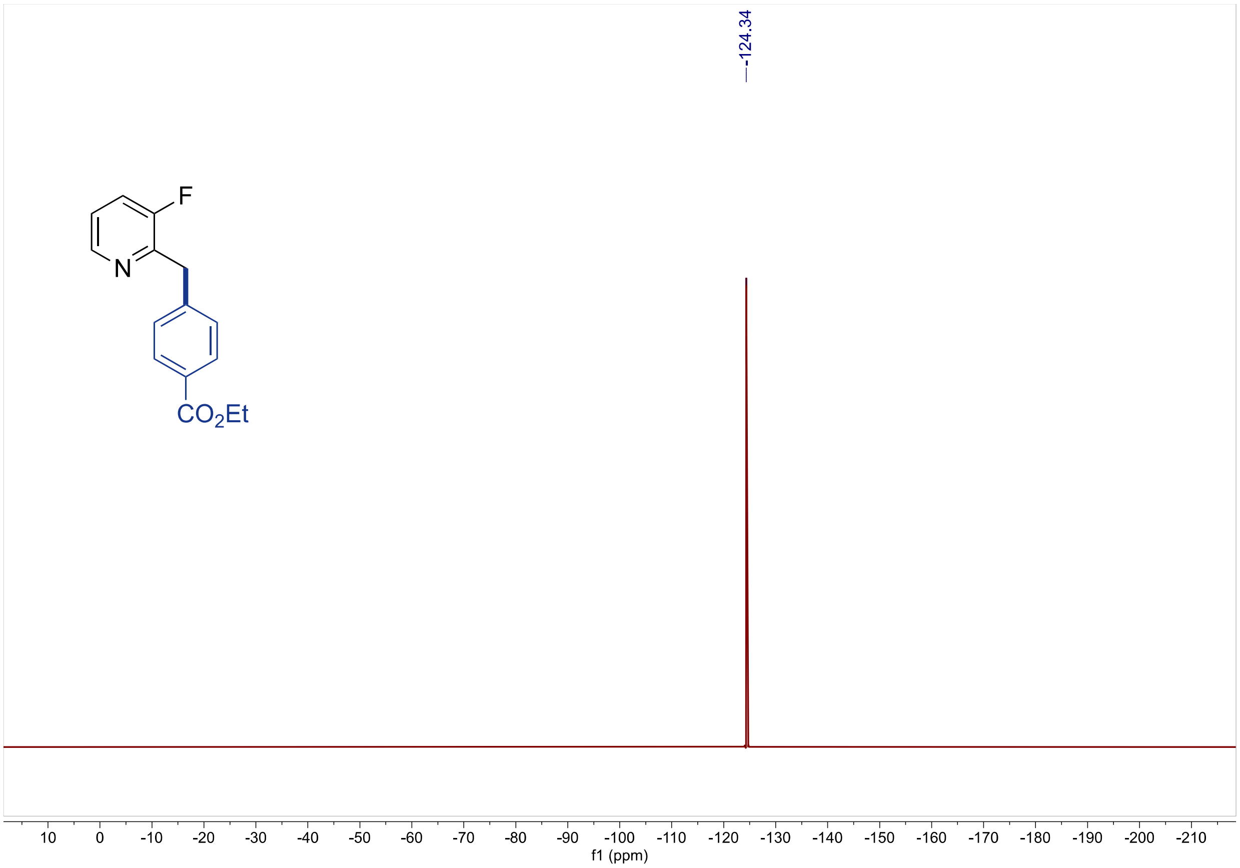

^{1}H NMR (500 MHz, $CDCl_3$) of **11**

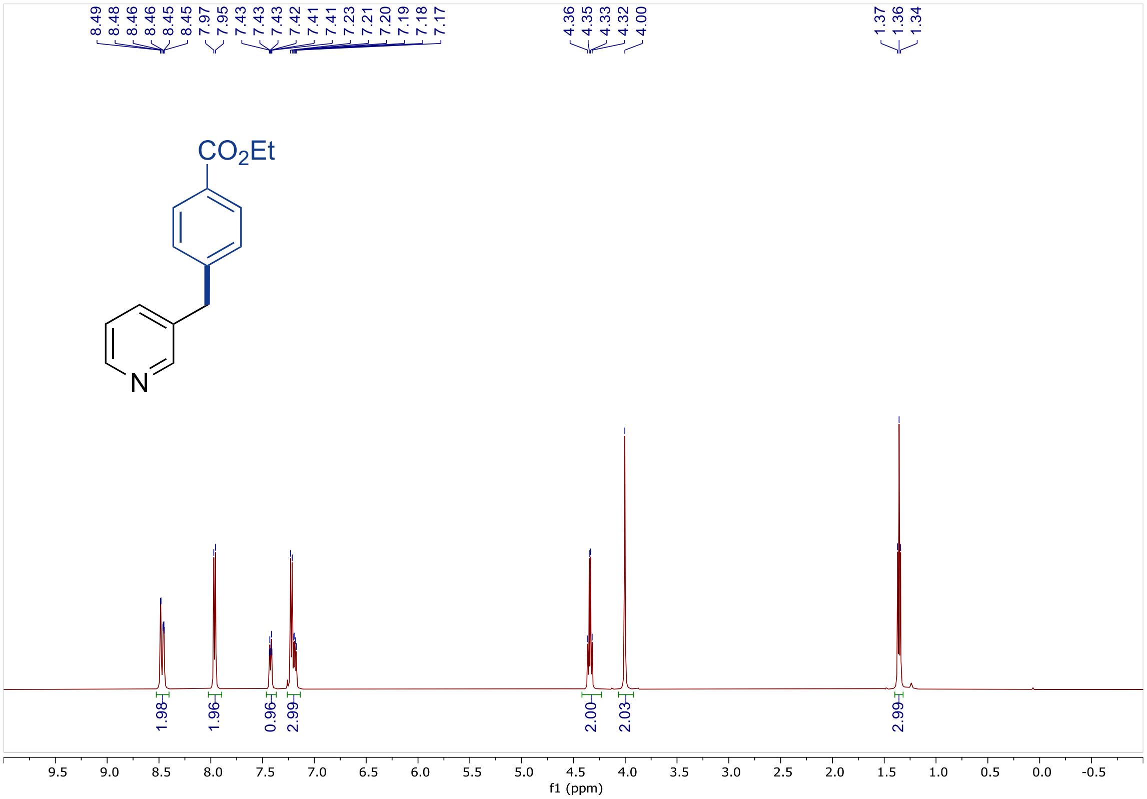

^{13}C NMR: (126 MHz, $CDCl_3$) of **11**

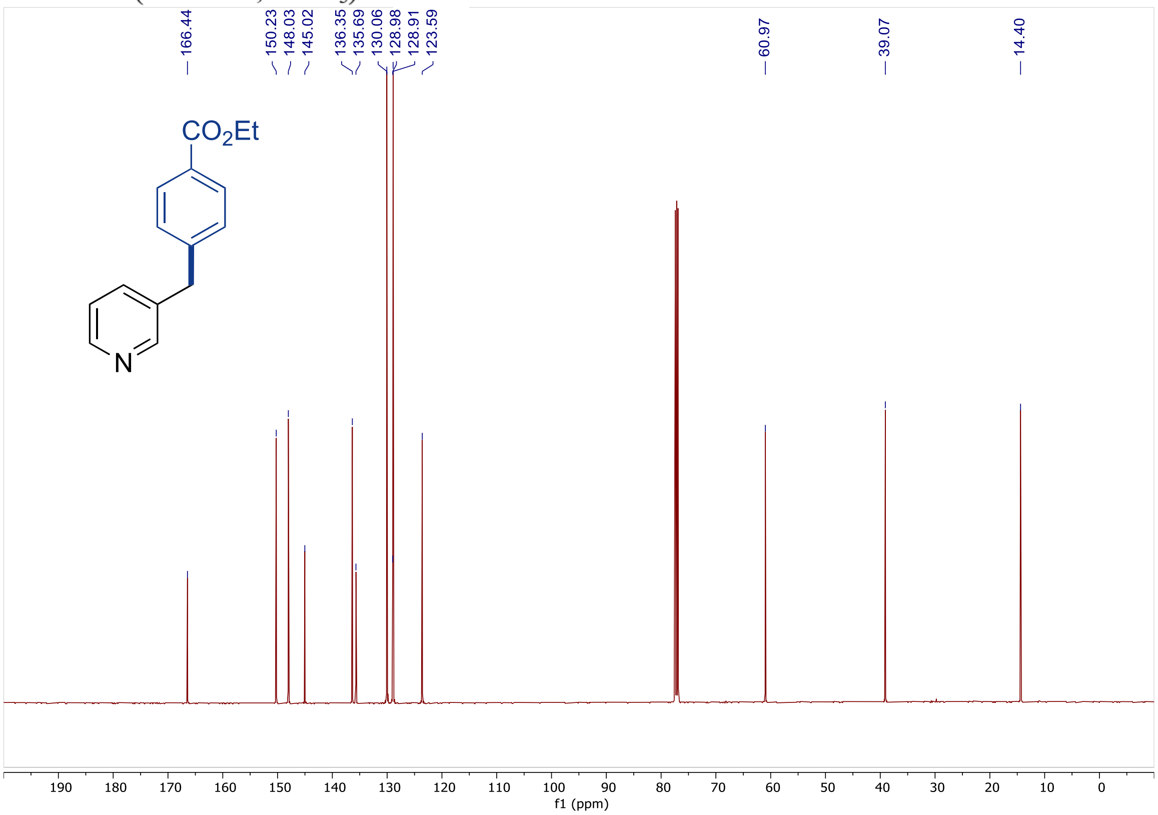

^{1}H NMR (500 MHz, $CDCl_3$) of **12**

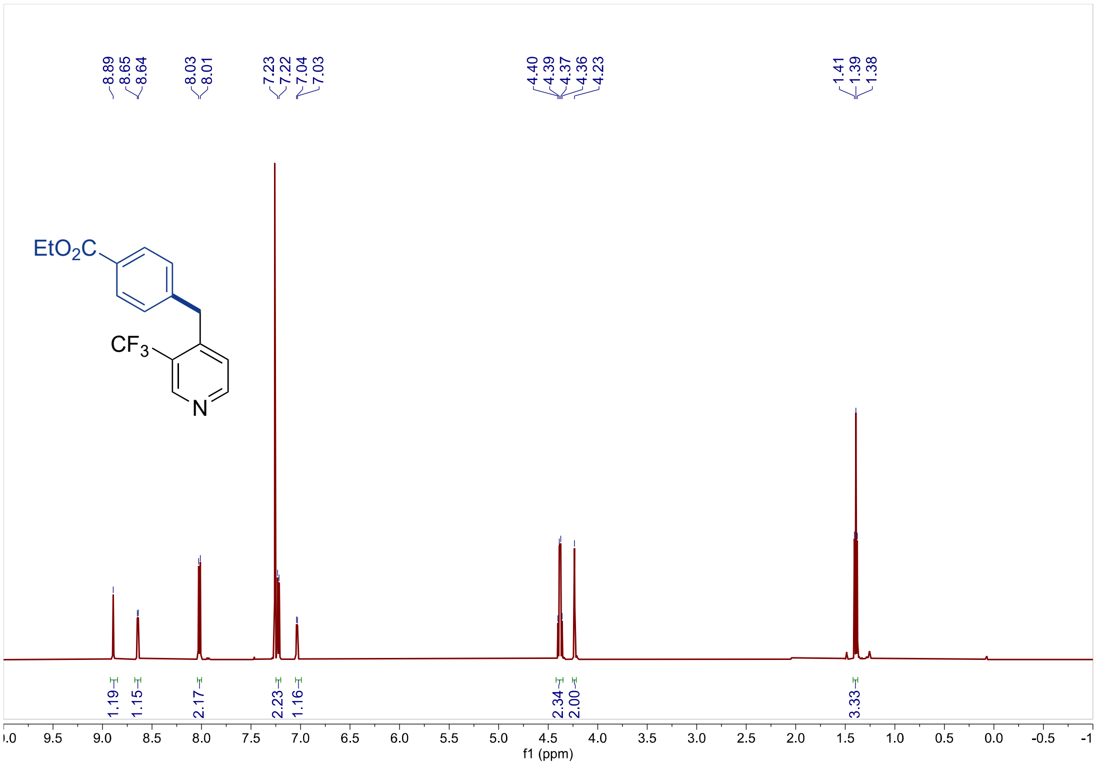

^{13}C NMR: (126 MHz, $CDCl_3$) of **12**

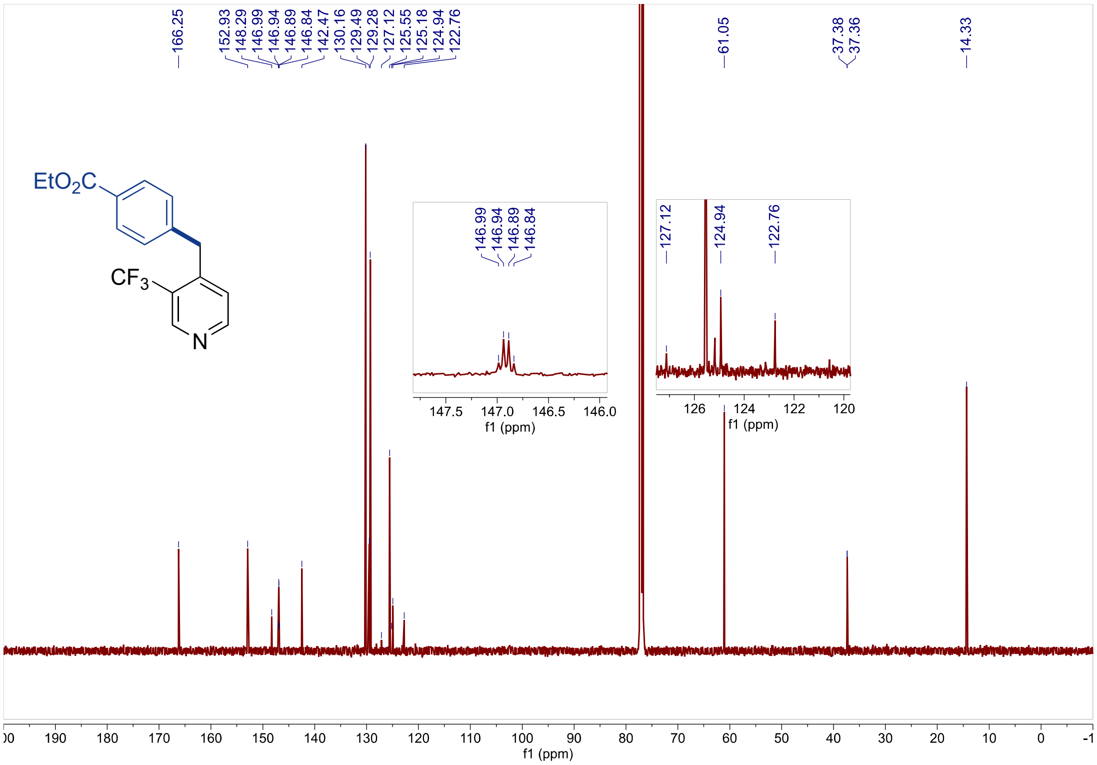

^{19}F NMR (377 MHz, $CDCl_3$) of **12**

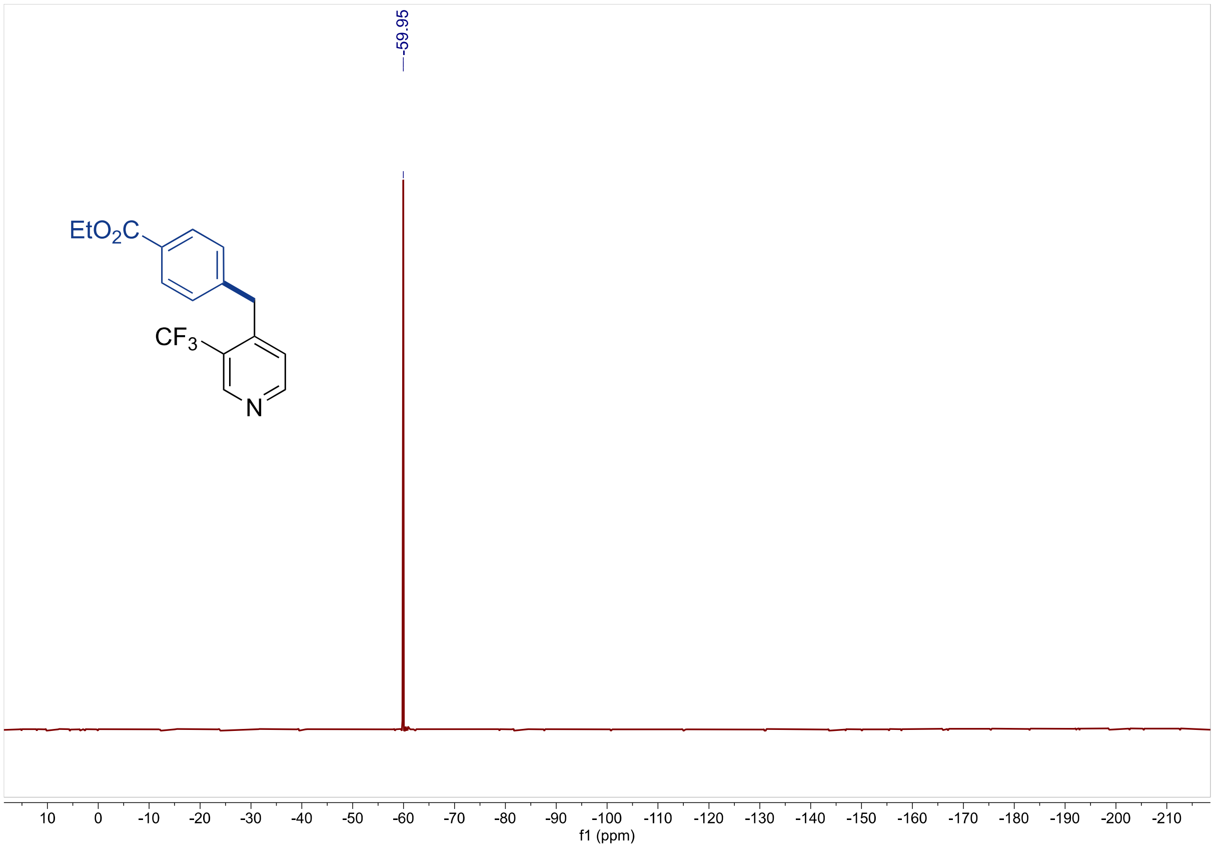

^{1}H NMR (500 MHz, $CDCl_3$) of **13**

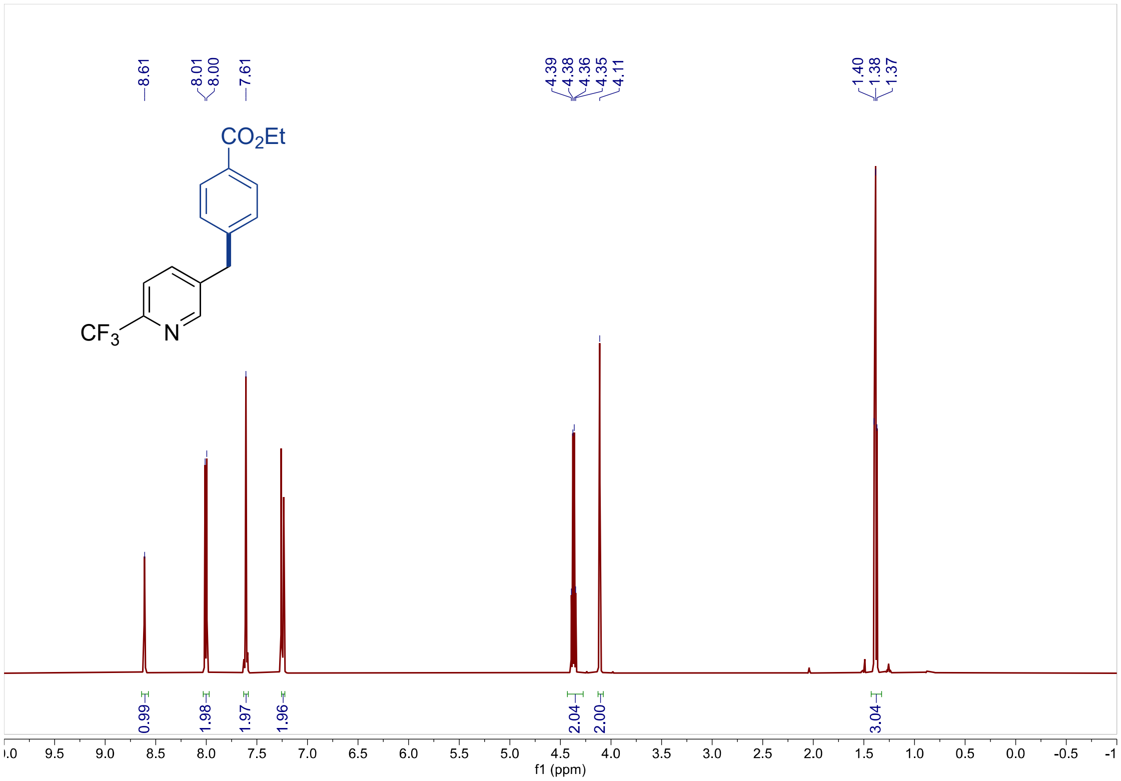

^{13}C NMR: (126 MHz, $CDCl_3$) of **13**

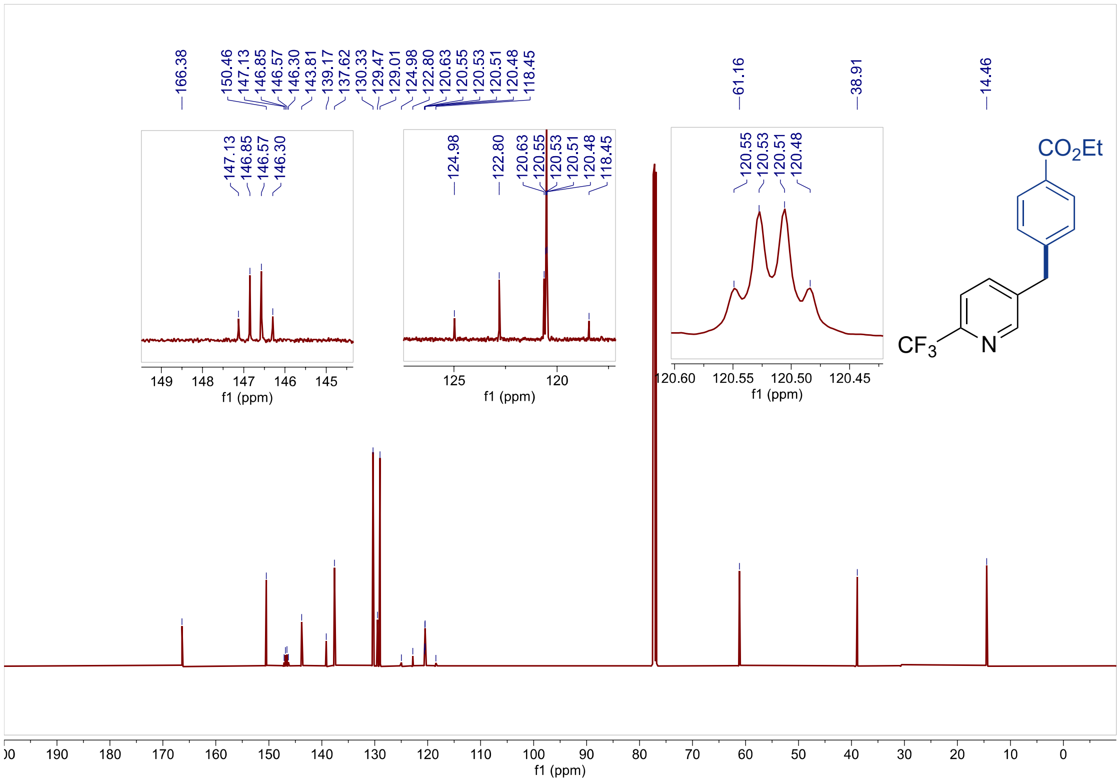

^{19}F NMR (377 MHz, $CDCl_3$) of **13**

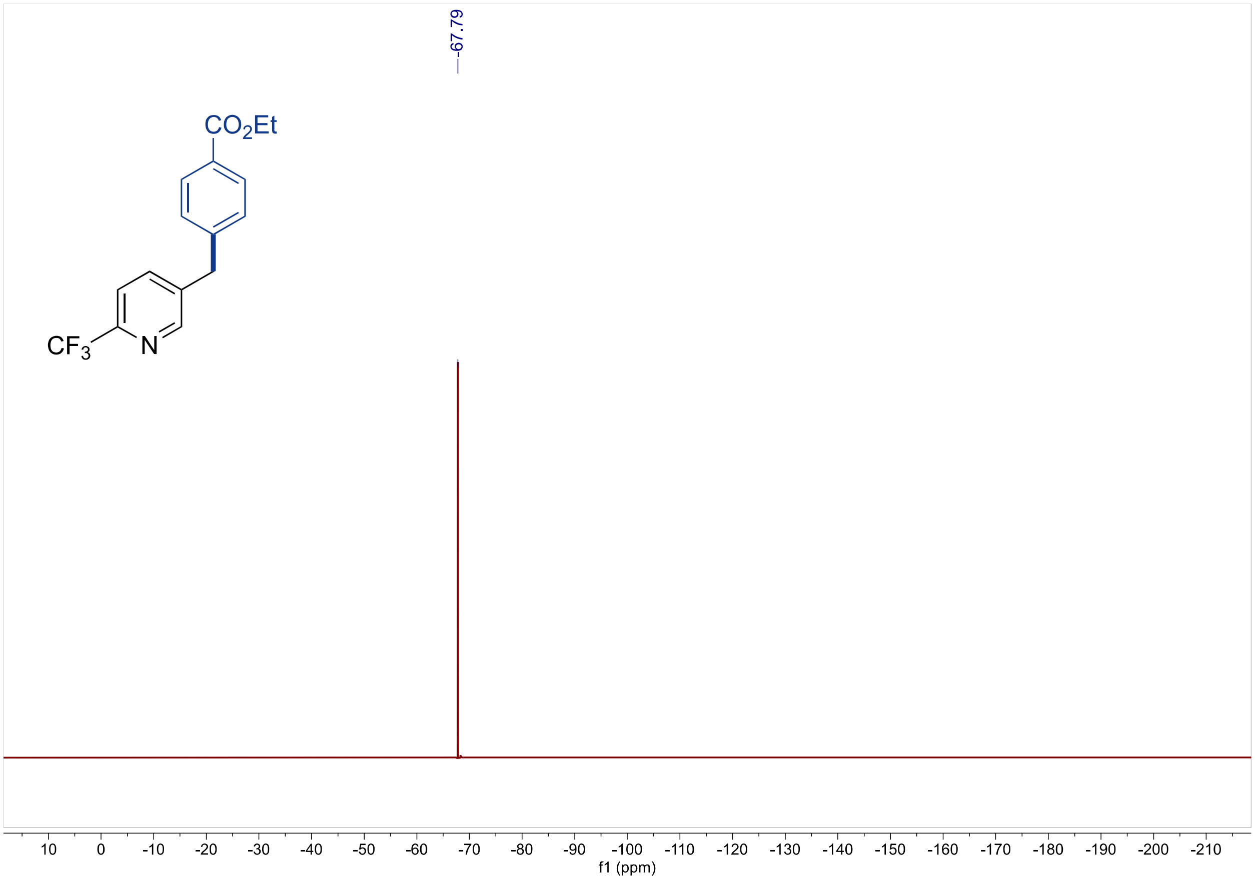

¹H NMR (500 MHz, $CDCl_3$) of **14**

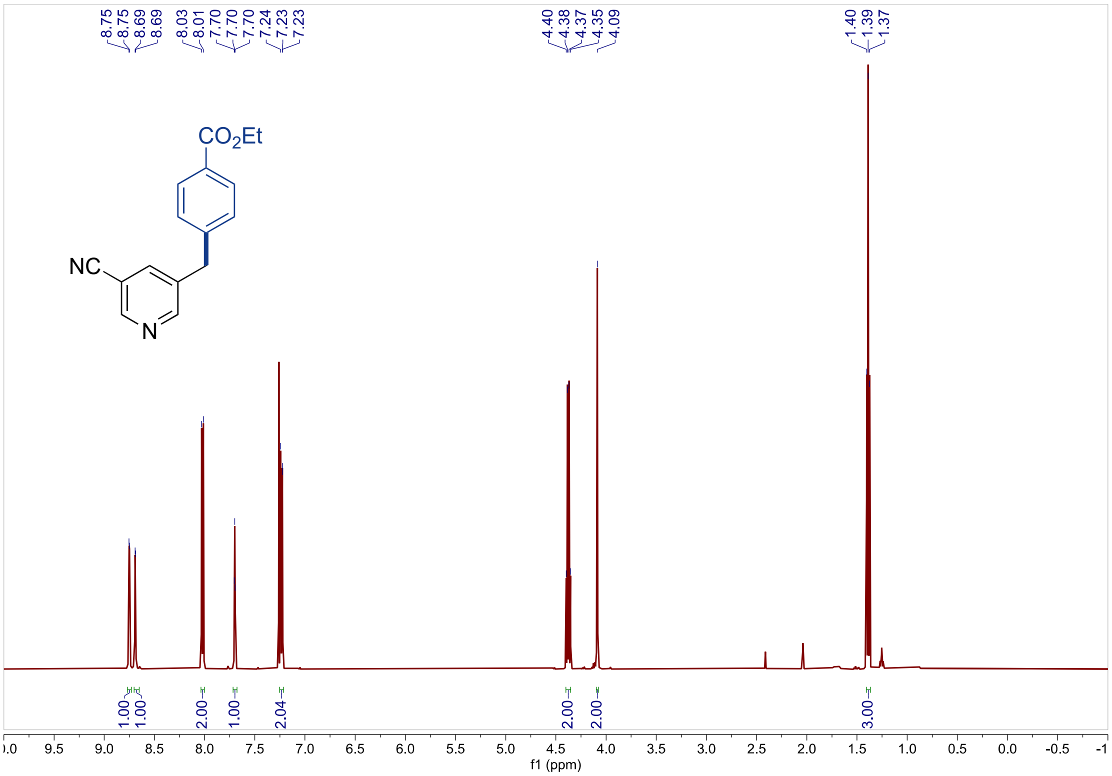

¹³C NMR: (126 MHz, $CDCl_3$) of **14**

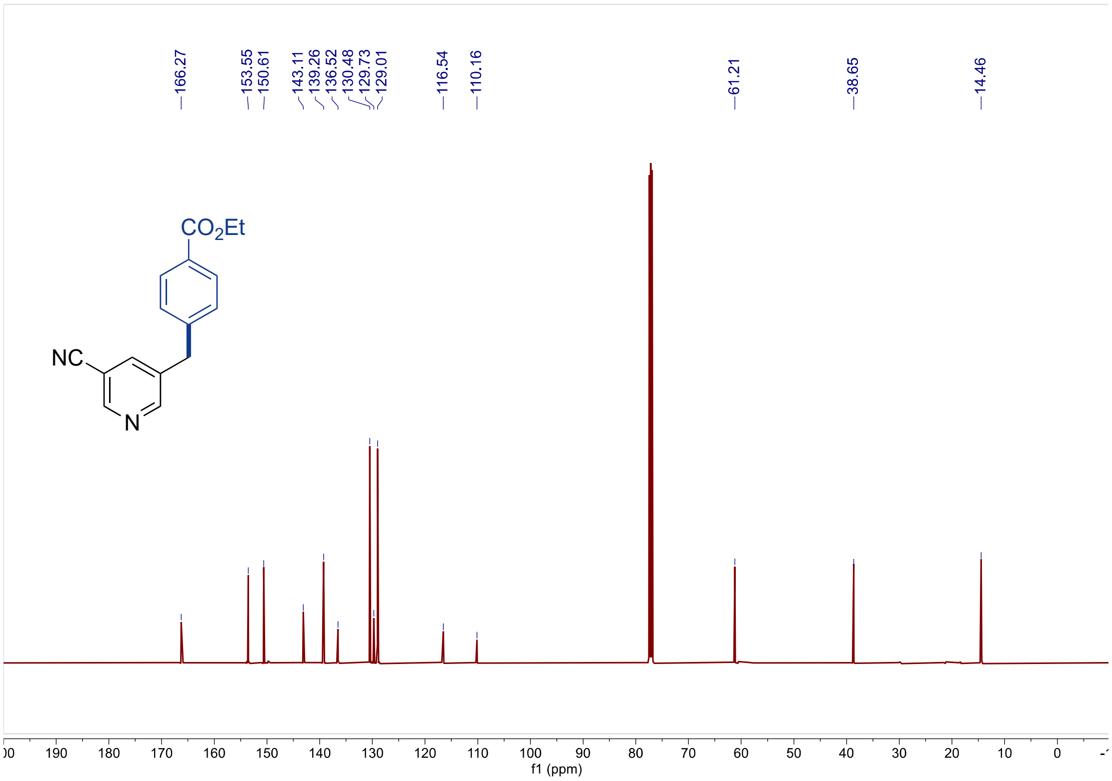

^{1}H NMR (500 MHz, $CDCl_3$) of **15**

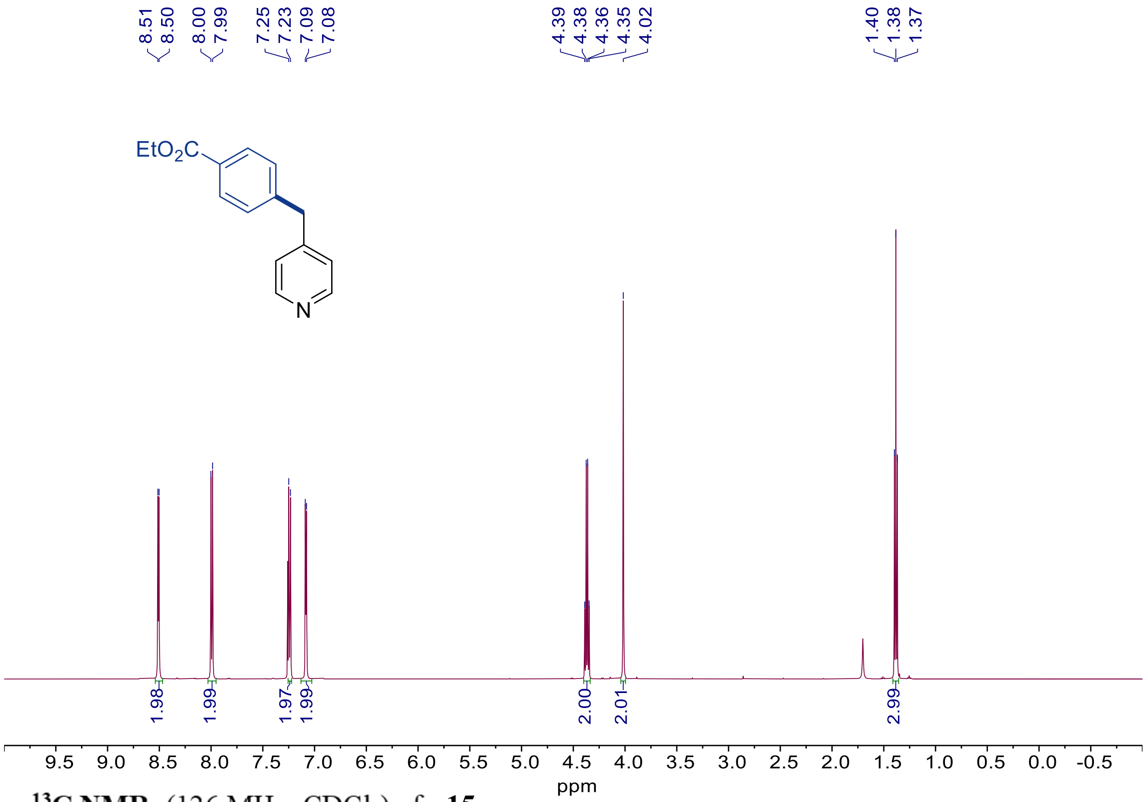

^{13}C NMR: (126 MHz, $CDCl_3$) of **15**

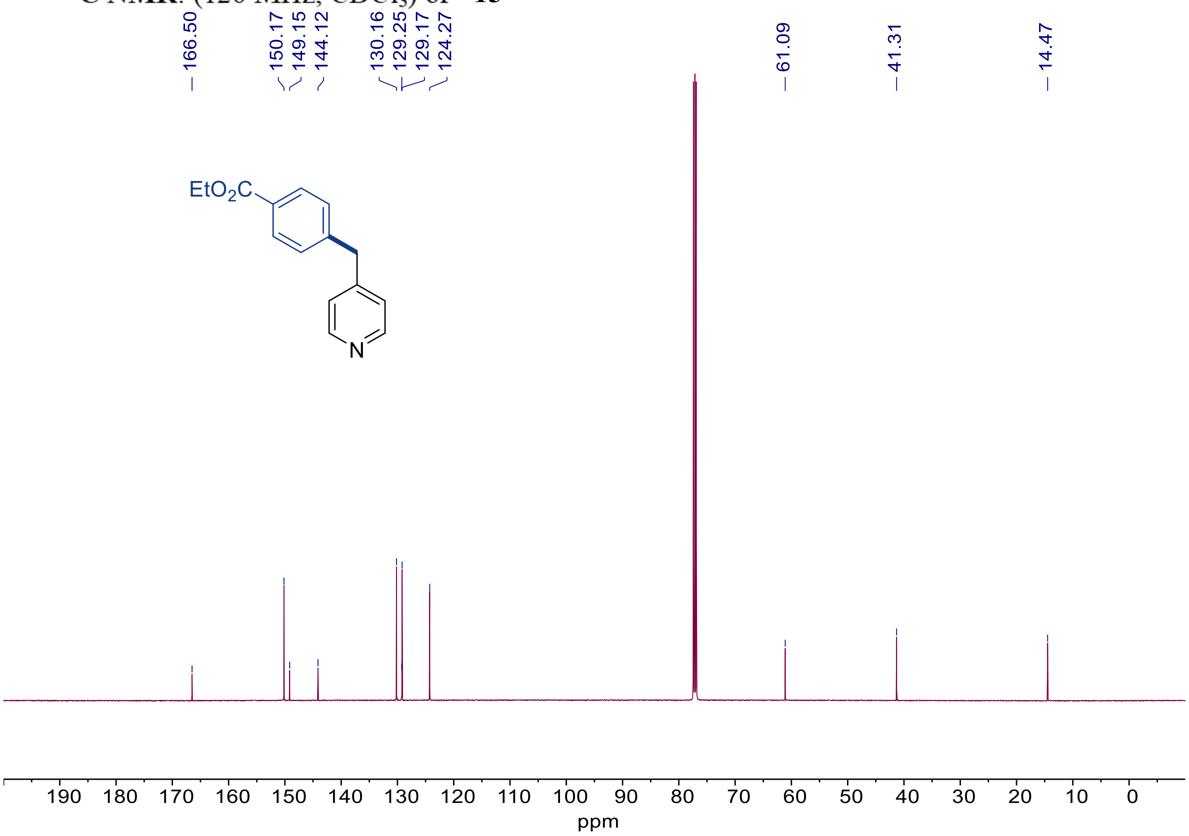

^{1}H NMR (500 MHz, $CDCl_3$) of **16**

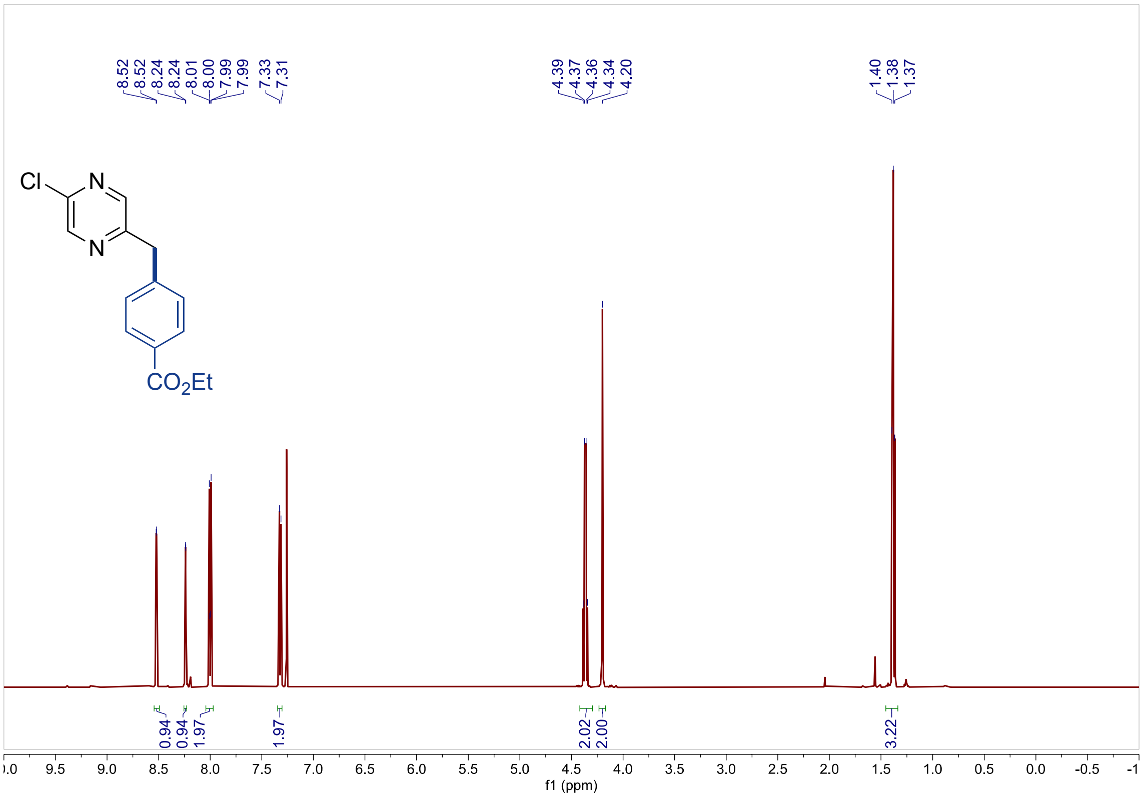

^{13}C NMR: (126 MHz, $CDCl_3$) of **16**

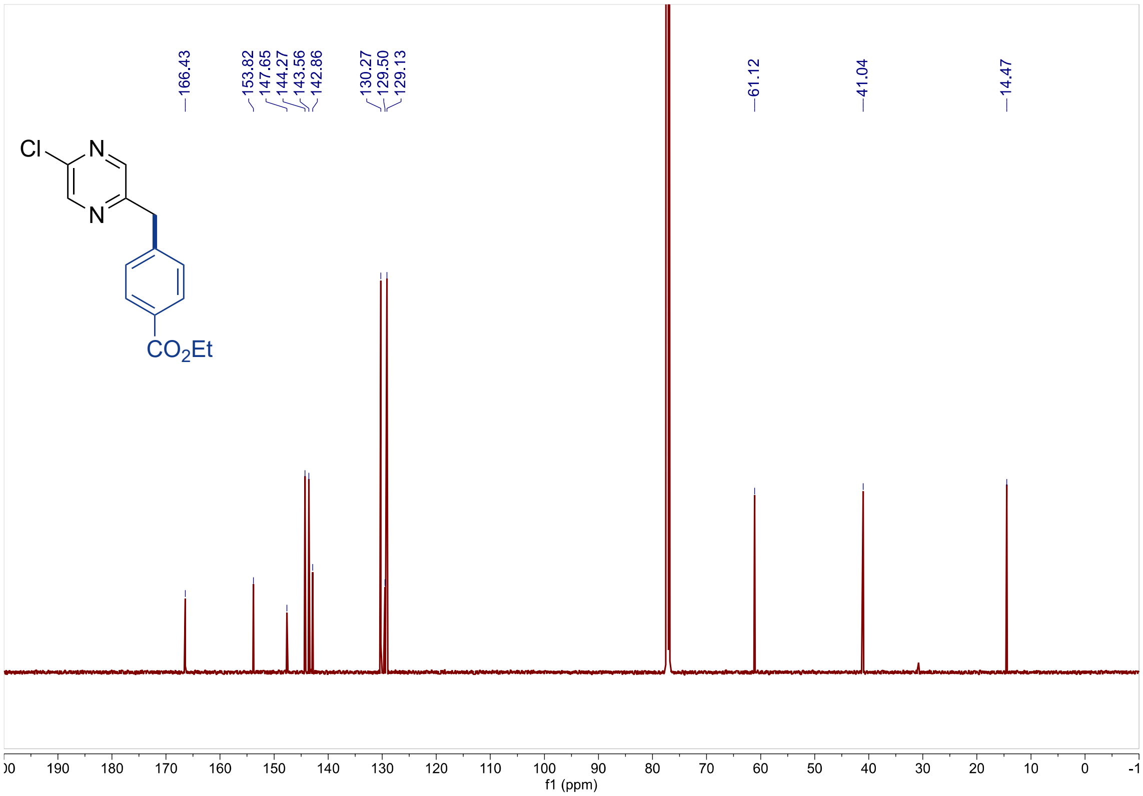

¹H NMR (500 MHz, $CDCl_3$) of **17**

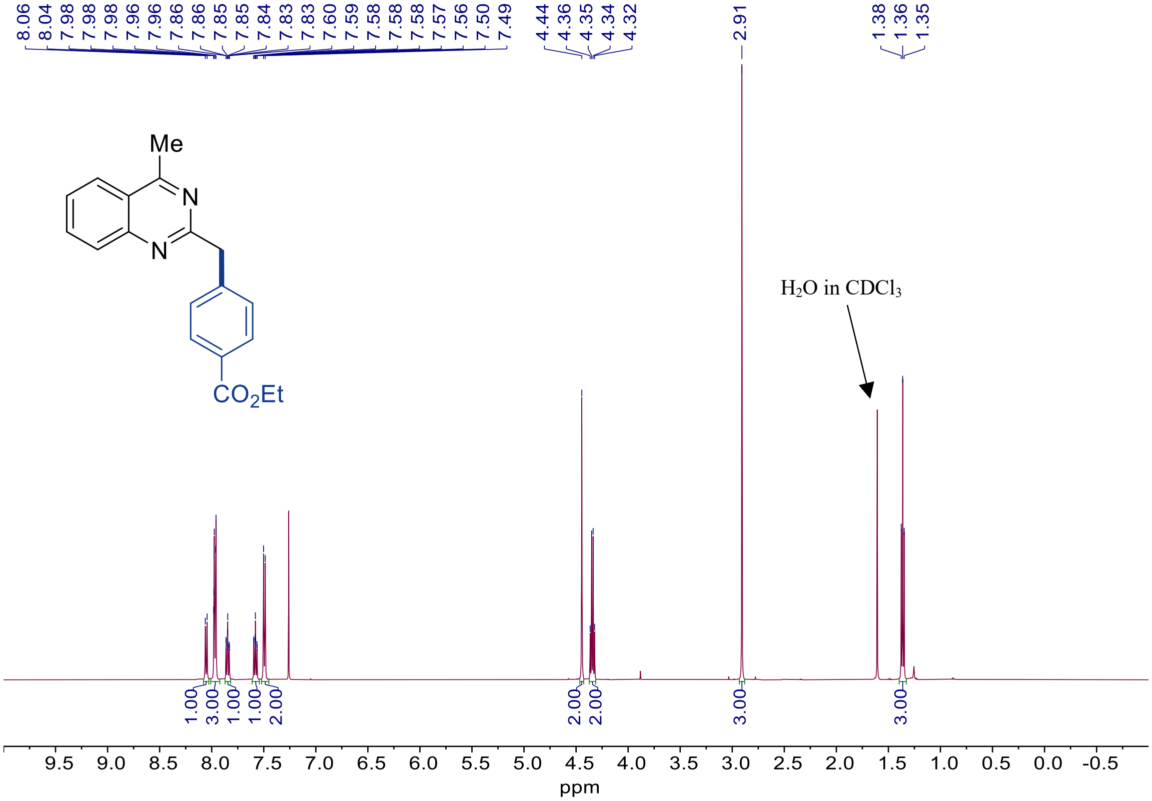

¹³C NMR: (126 MHz, $CDCl_3$) of **17**

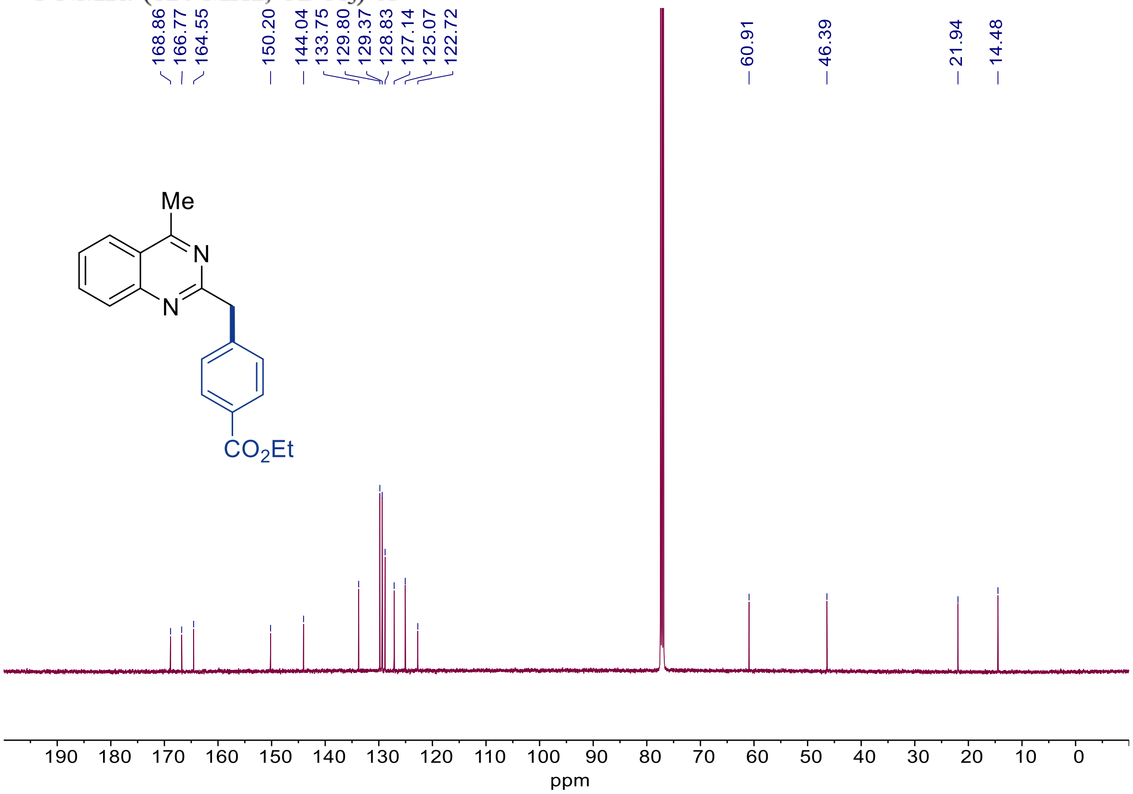

^{1}H NMR (500 MHz, $CDCl_3$) of **18**

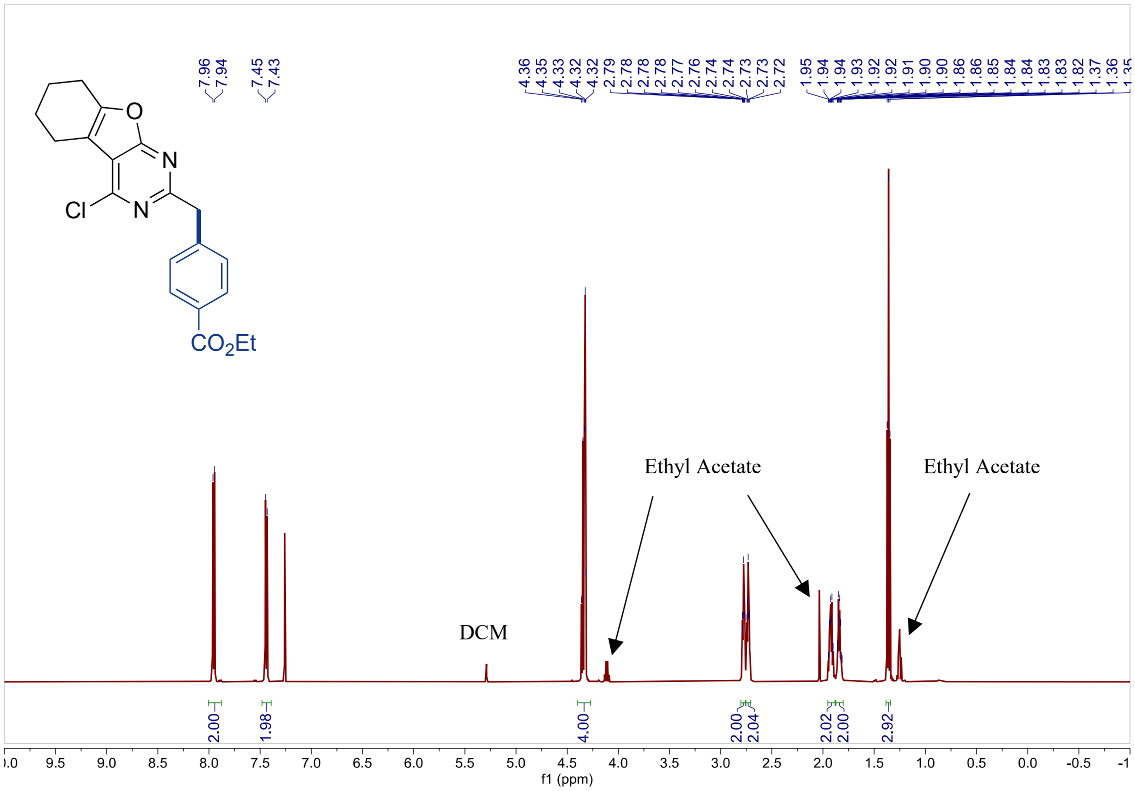

^{13}C NMR: (126 MHz, $CDCl_3$) of **18**

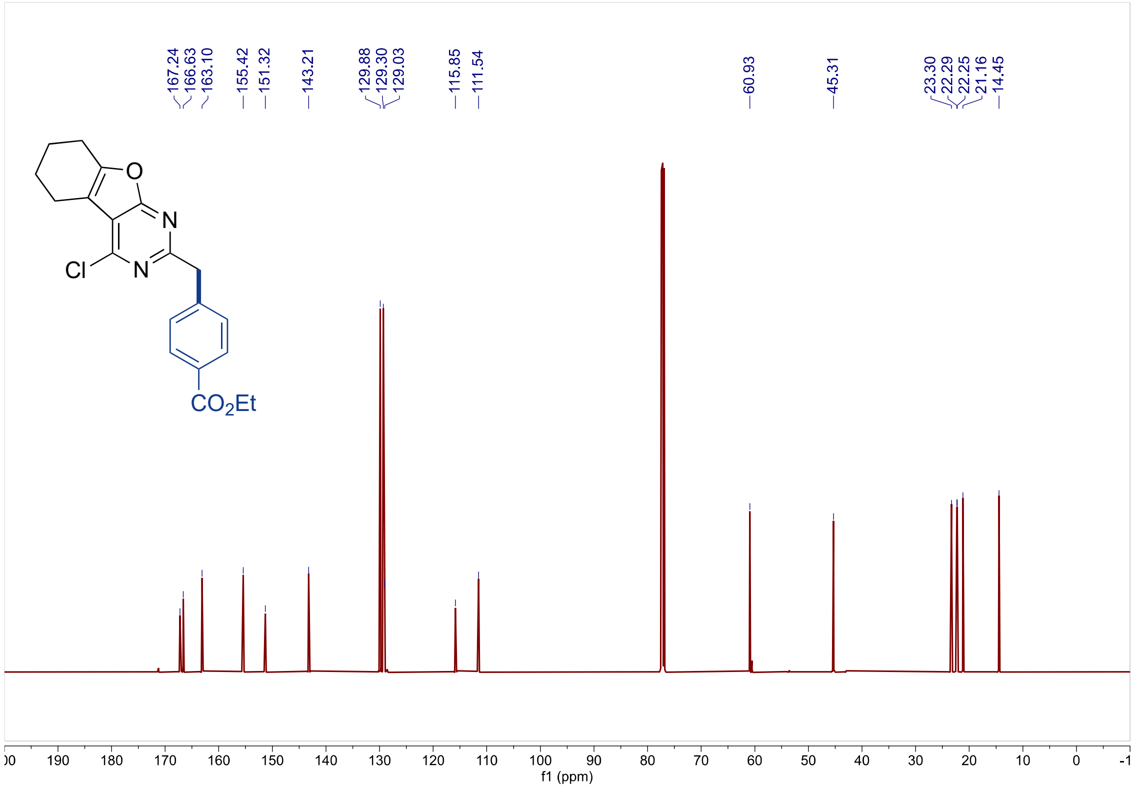

^{1}H NMR (500 MHz, $CDCl_3$) of **19**

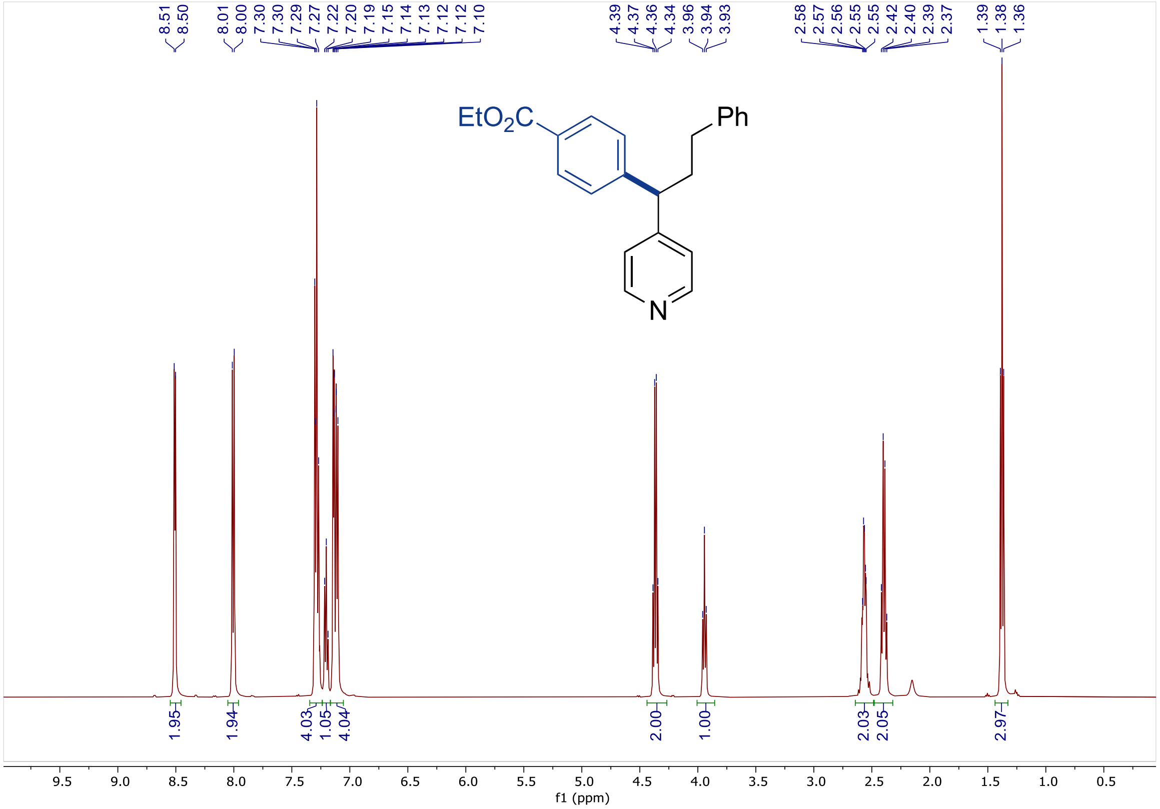

^{13}C NMR: (126 MHz, $CDCl_3$) of **19**

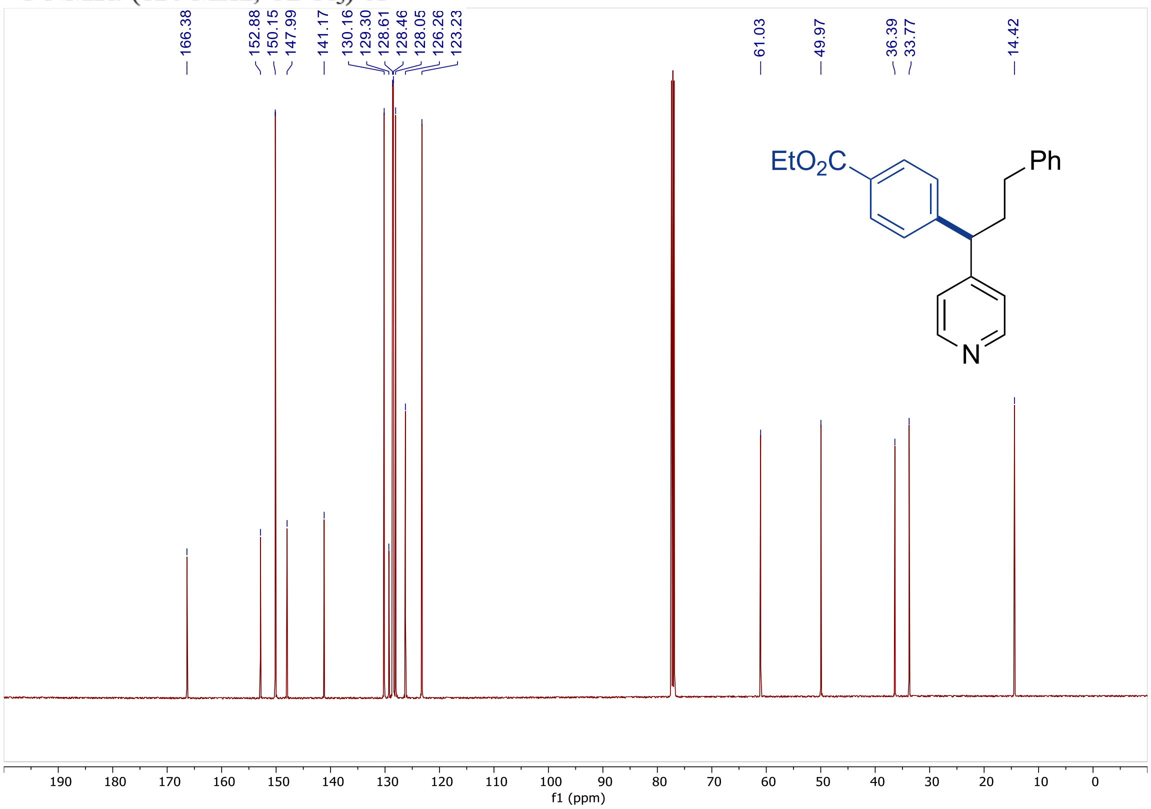

^{1}H NMR (500 MHz, $CDCl_3$) of **20**

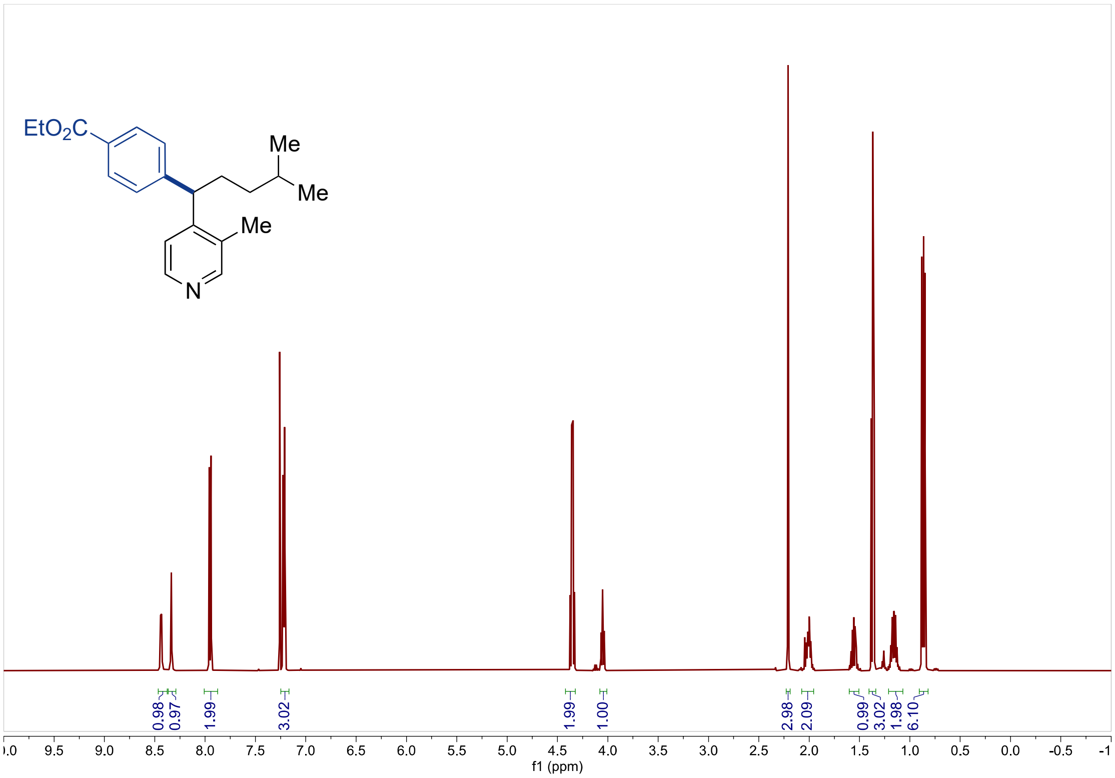

^{13}C NMR: (126 MHz, $CDCl_3$) of **20**

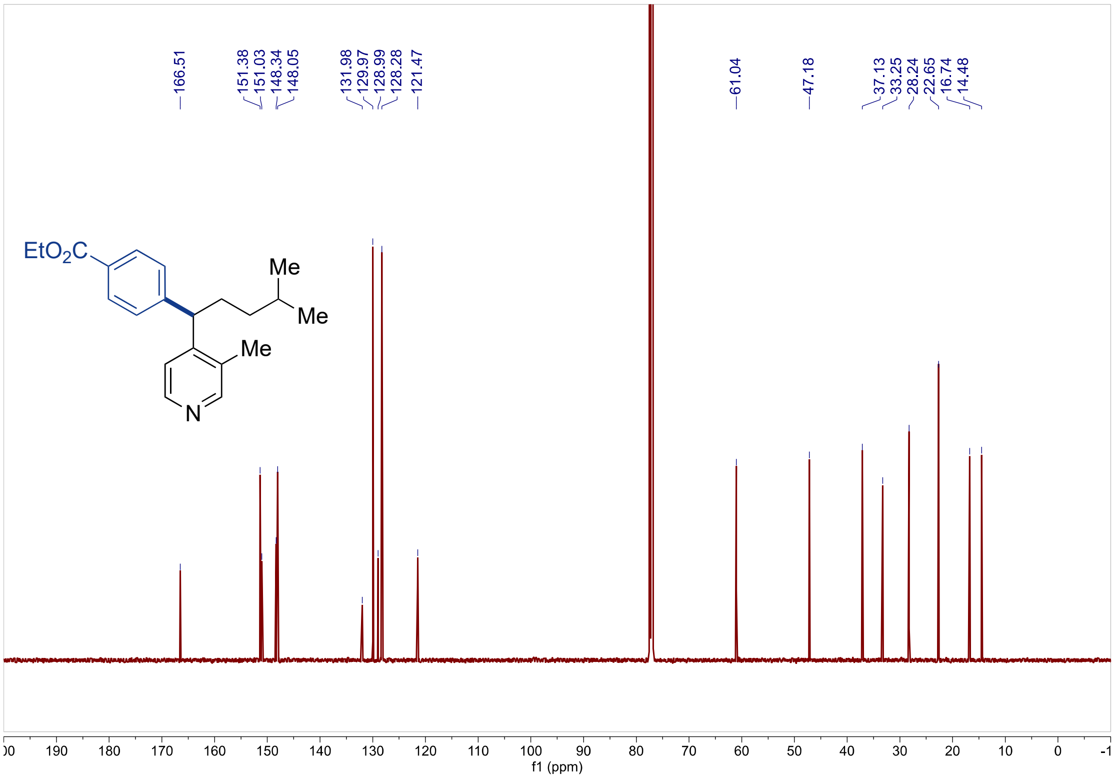

1H NMR (500 MHz, $CDCl_3$) of **21**

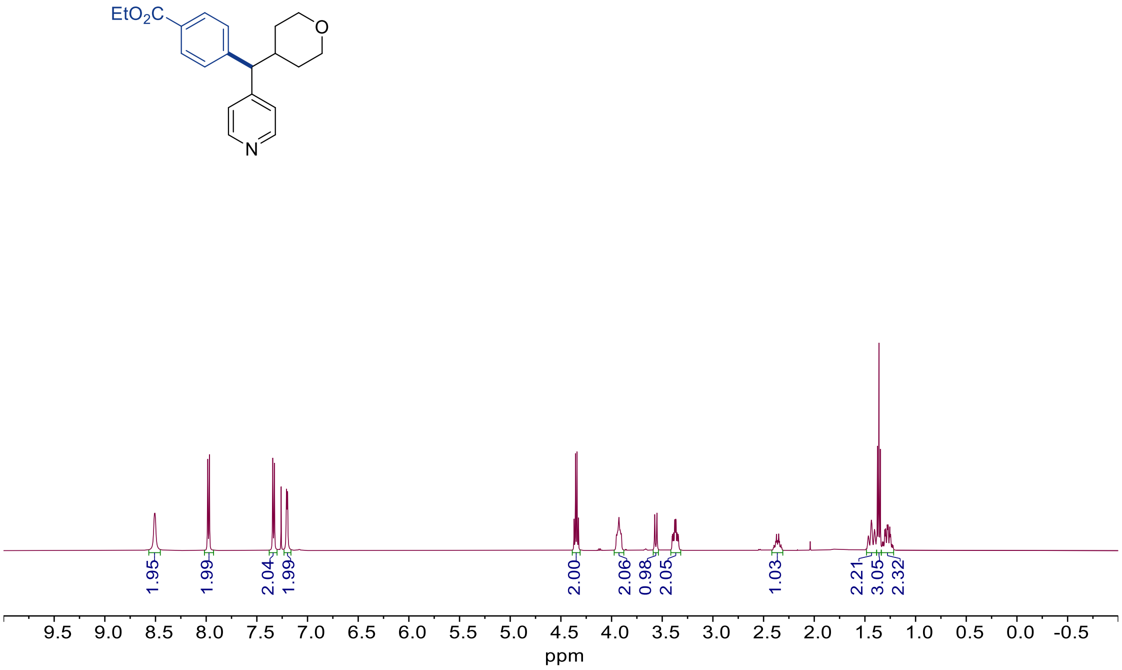

13C NMR: (126 MHz, $CDCl_3$) of **21**

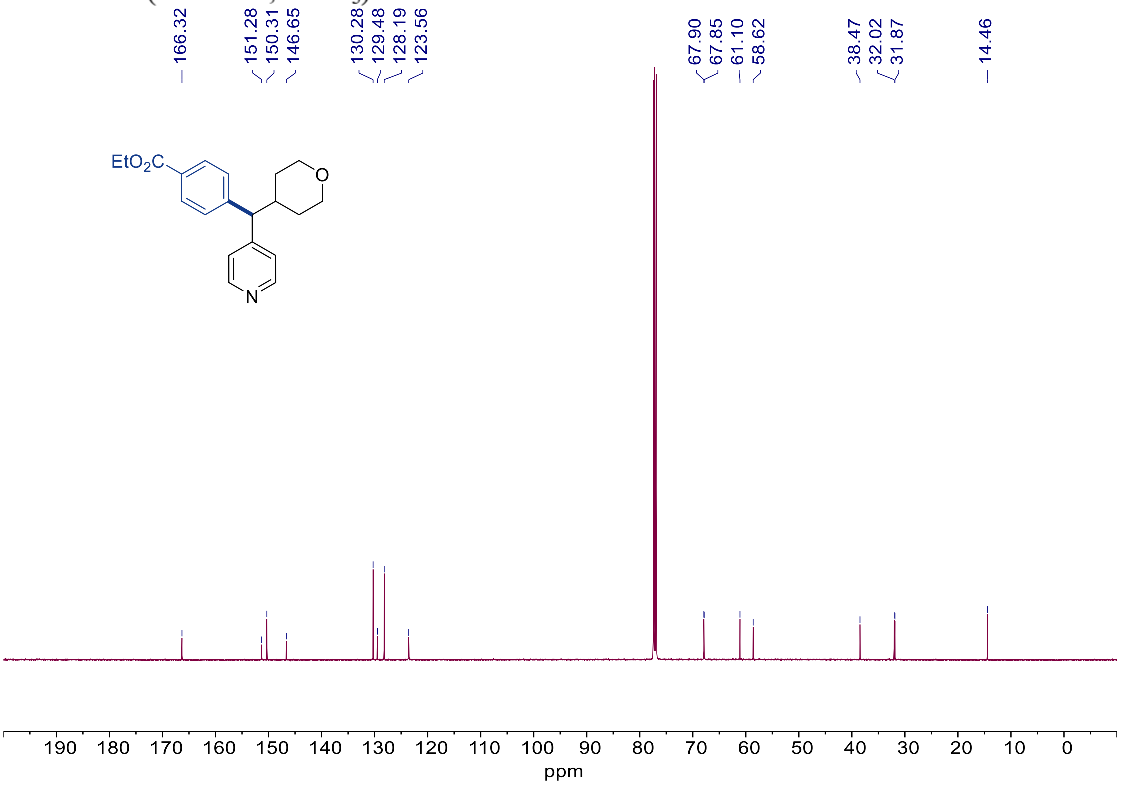

^{1}H NMR (500 MHz, $CDCl_3$) of **22**

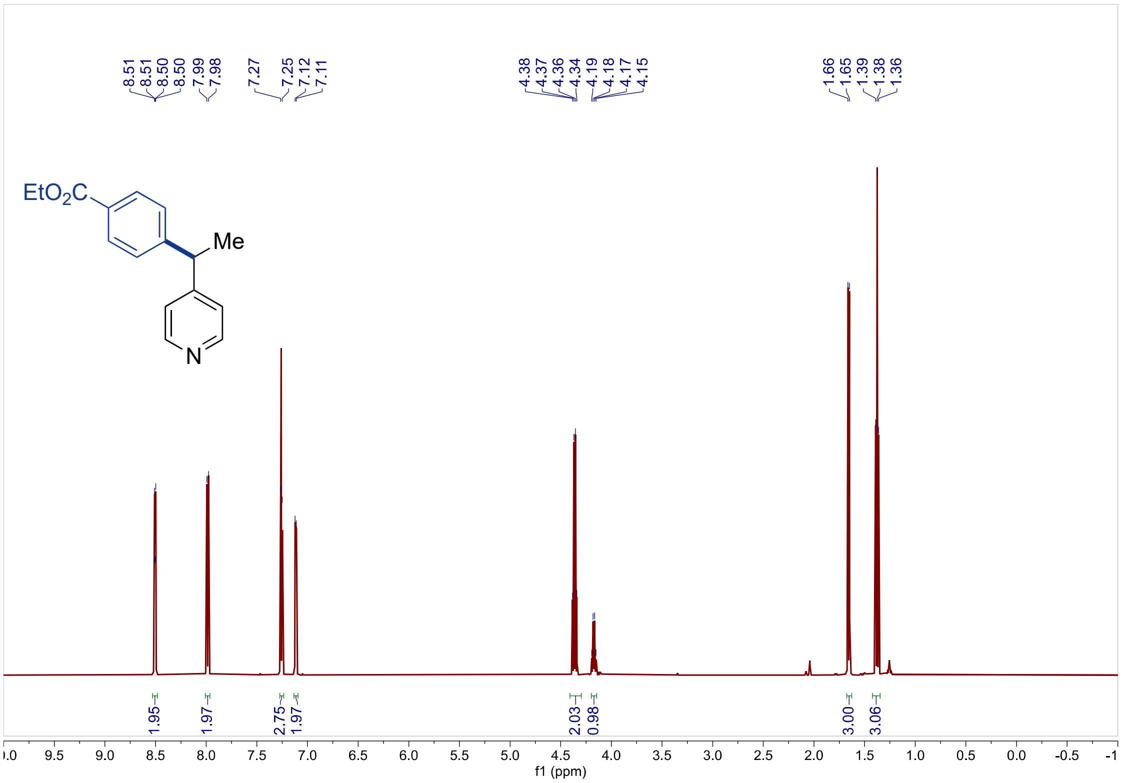

^{13}C NMR: (126 MHz, $CDCl_3$) of **22**

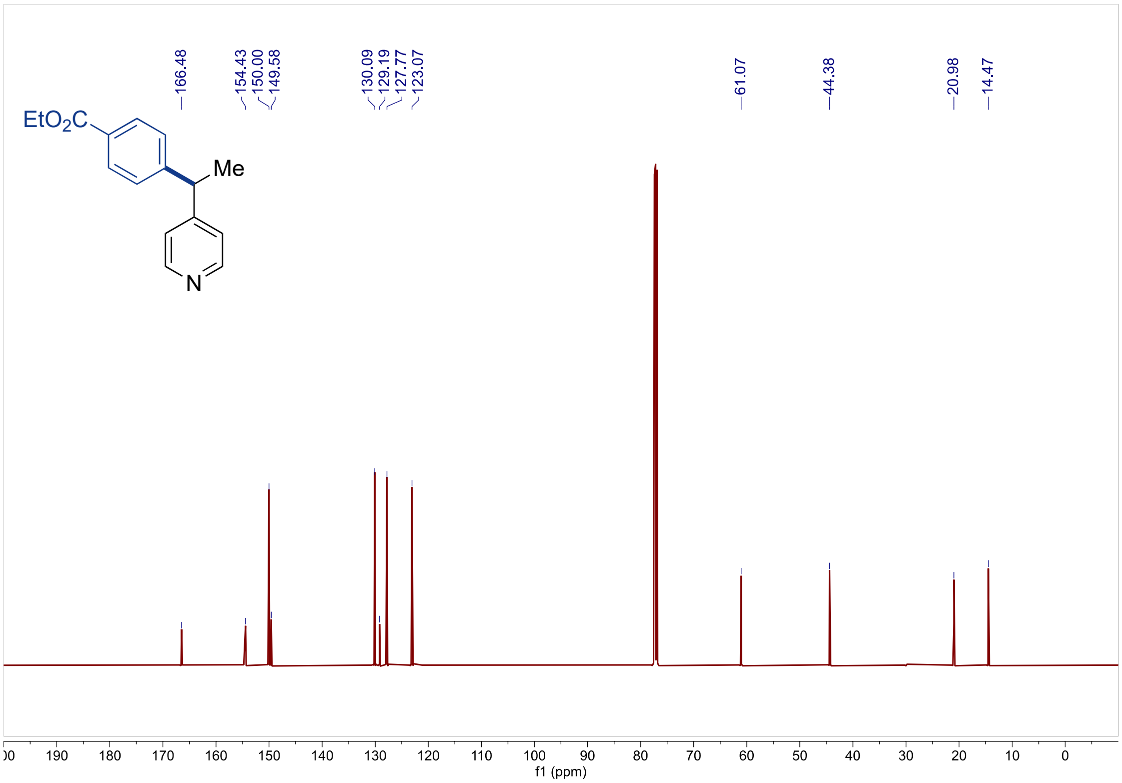

^{1}H NMR (500 MHz, $CDCl_3$) of **23**

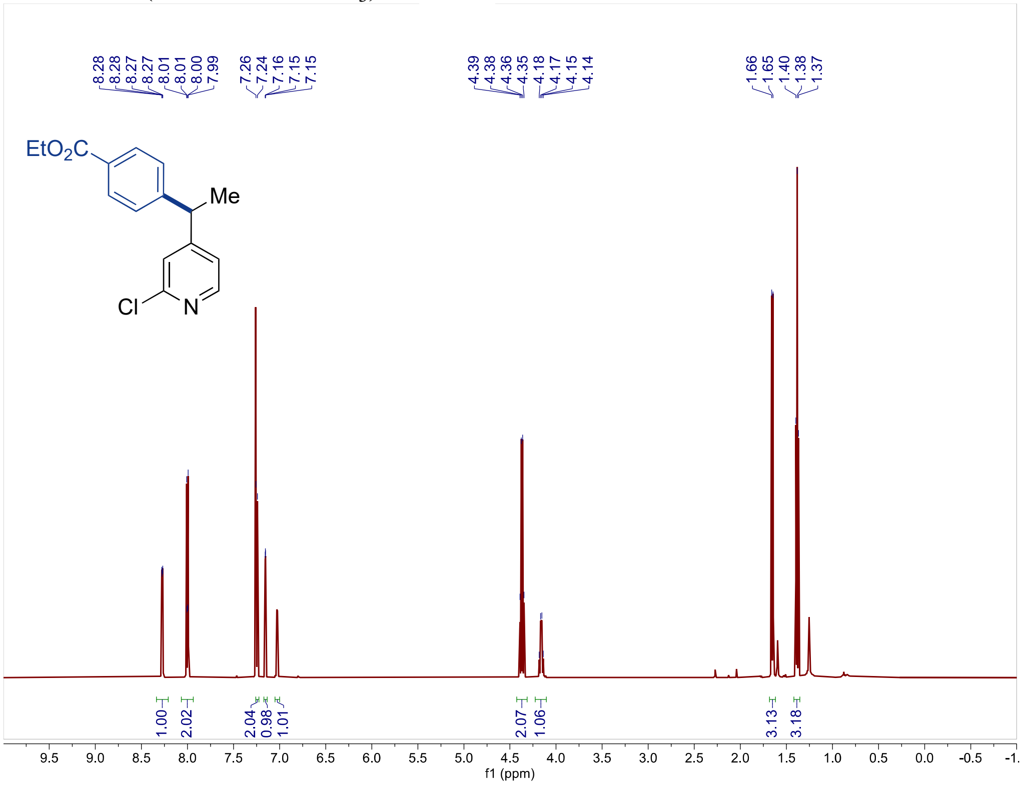

^{13}C NMR: (126 MHz, $CDCl_3$) of **23**

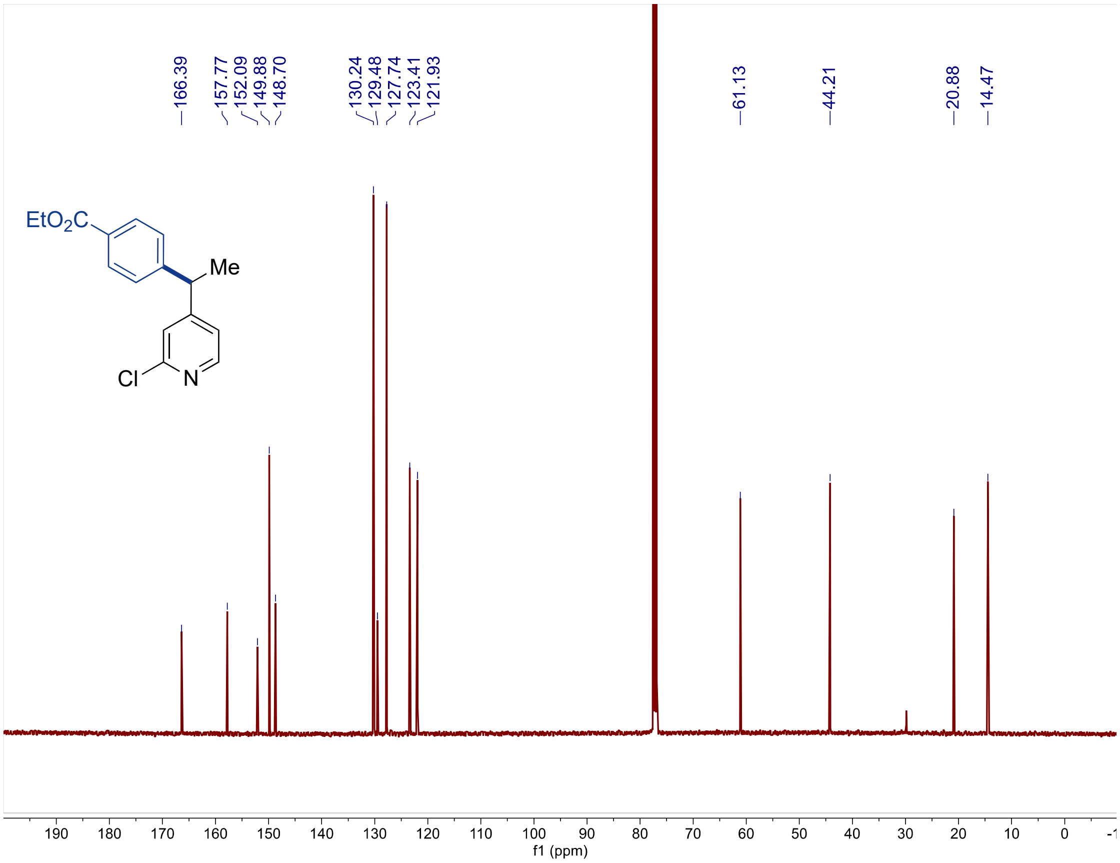

^{1}H NMR (500 MHz, $CDCl_3$) of **24**

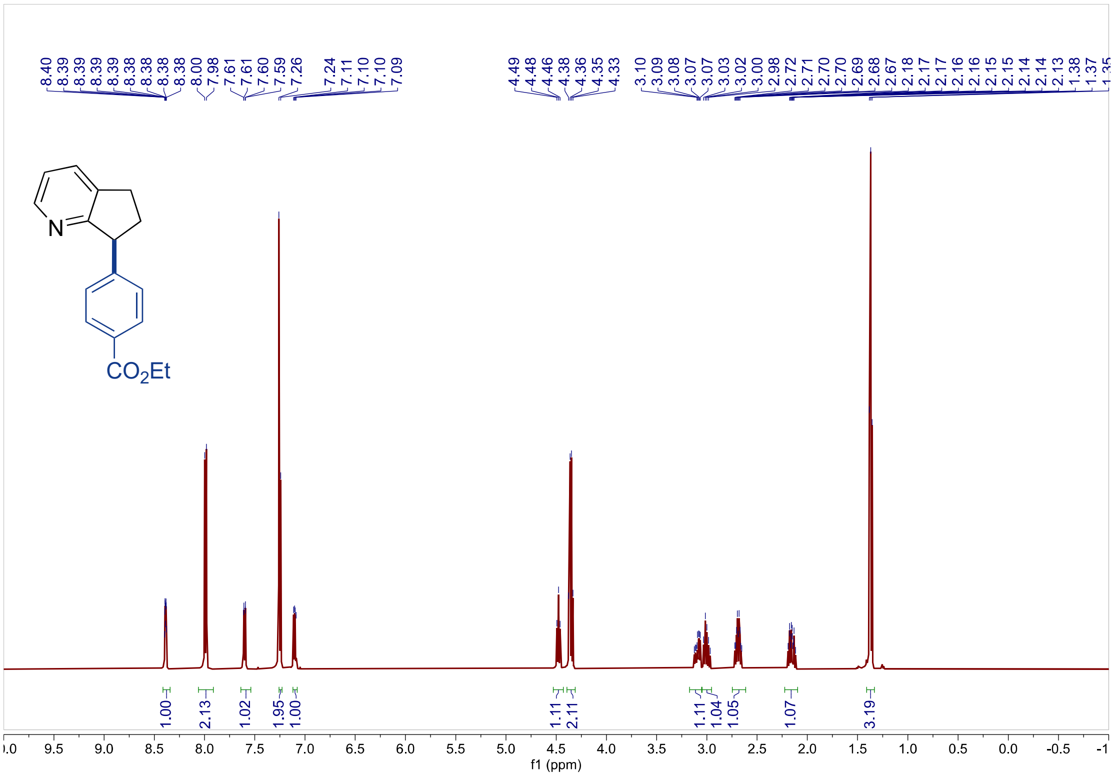

^{13}C NMR: (126 MHz, $CDCl_3$) of **24**

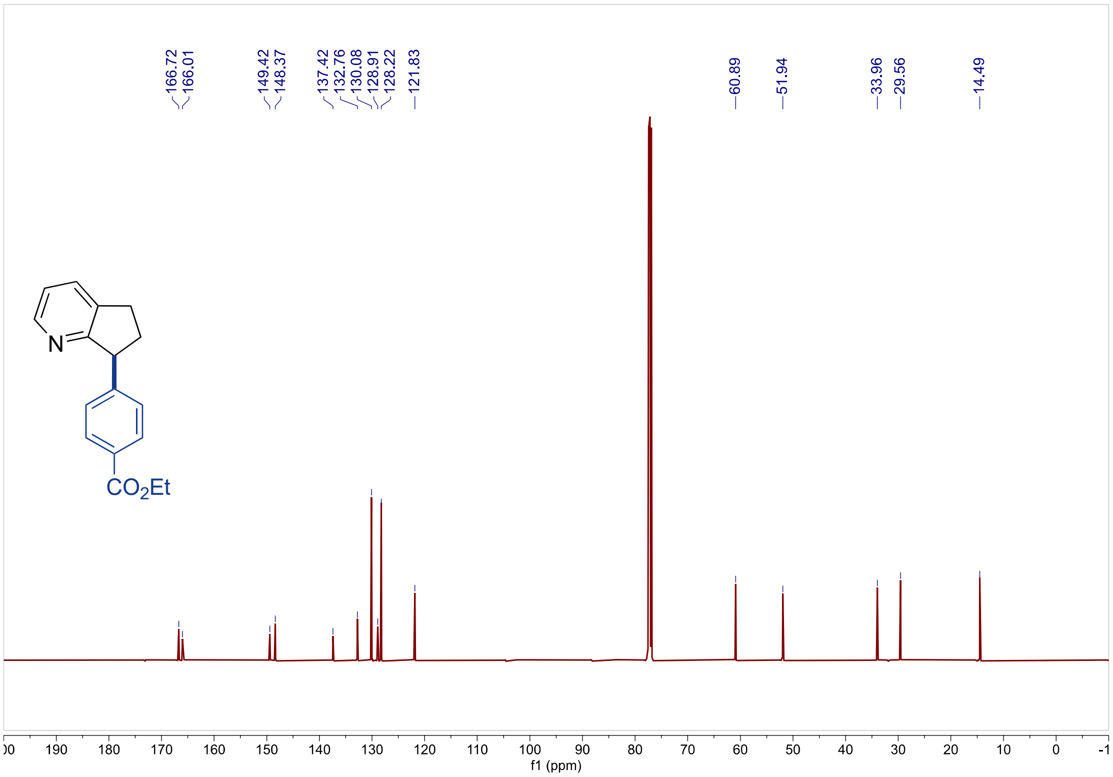

^{1}H NMR (500 MHz, $CDCl_3$) of **25**

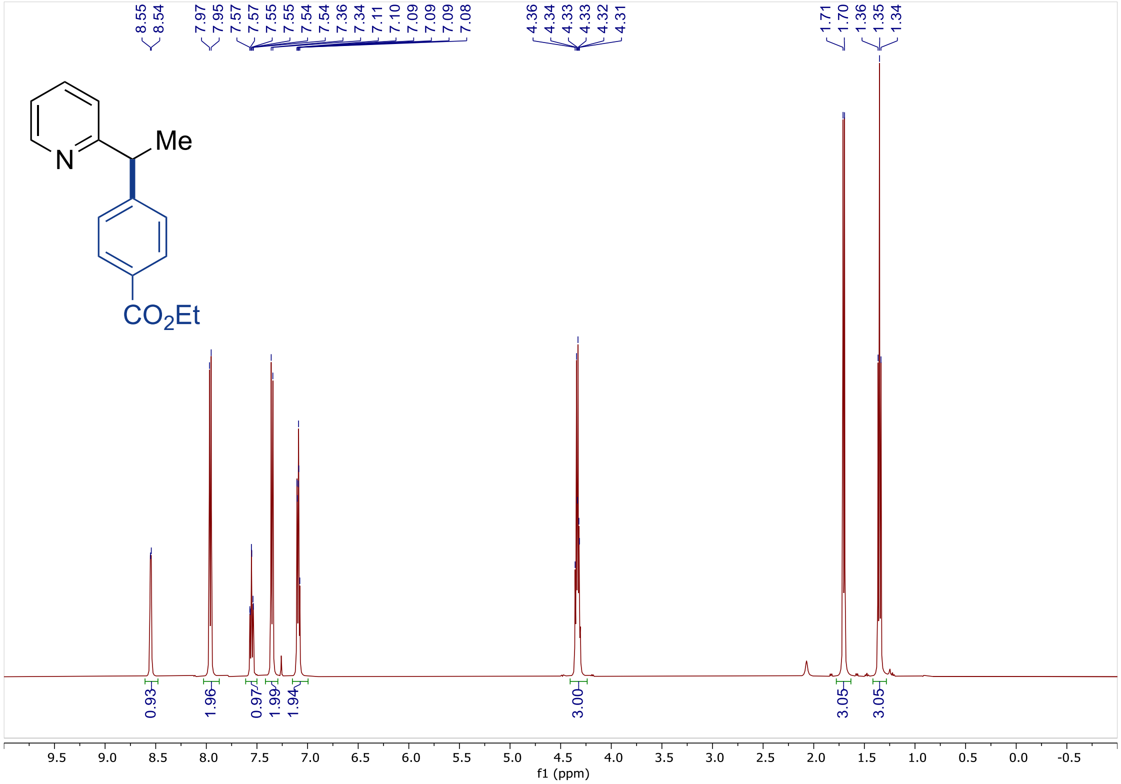

^{13}C NMR: (126 MHz, $CDCl_3$) of **25**

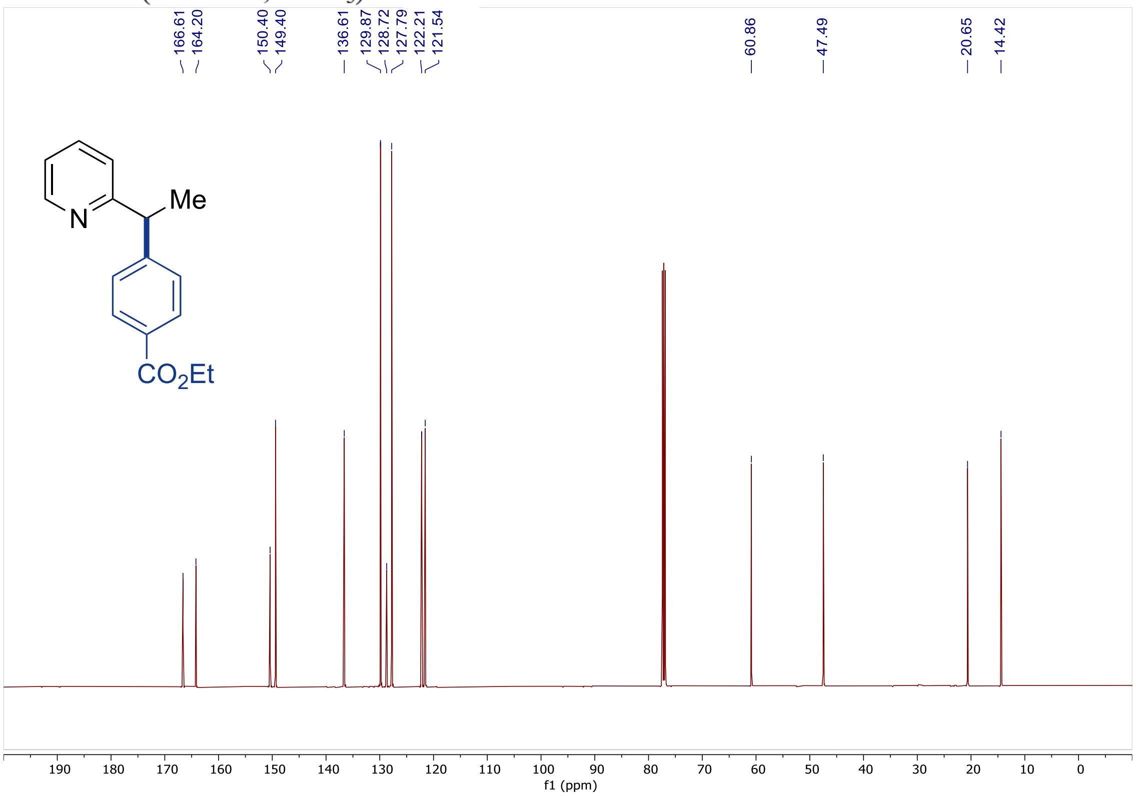

^{1}H NMR (500 MHz, $CDCl_3$) of **26**

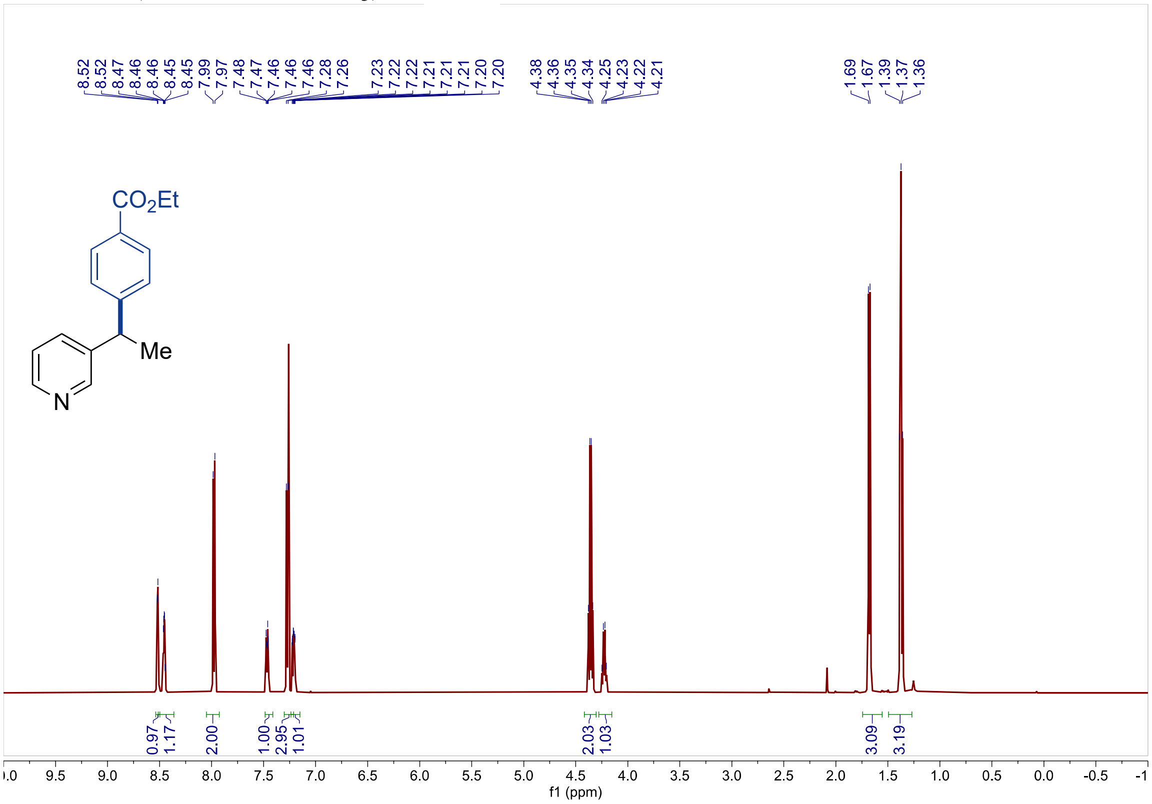

^{13}C NMR: (126 MHz, $CDCl_3$) of **26**

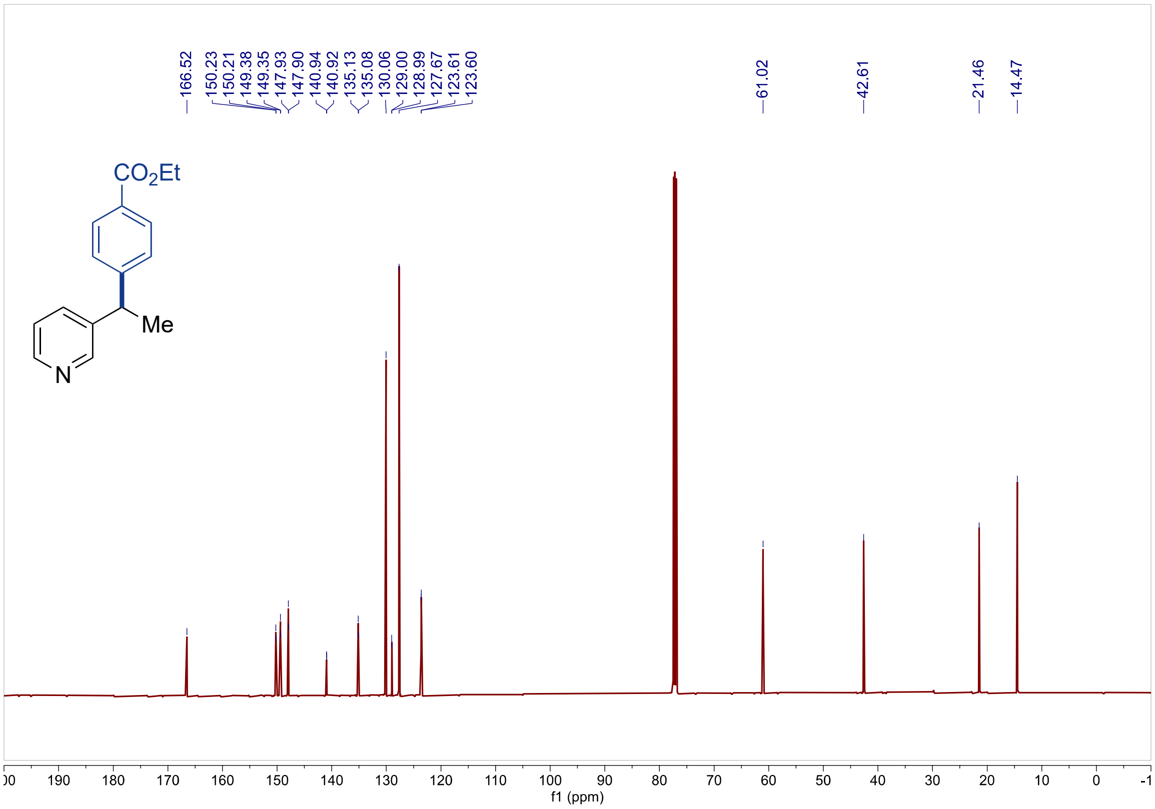

^{1}H NMR (500 MHz, $CDCl_3$) of **27**

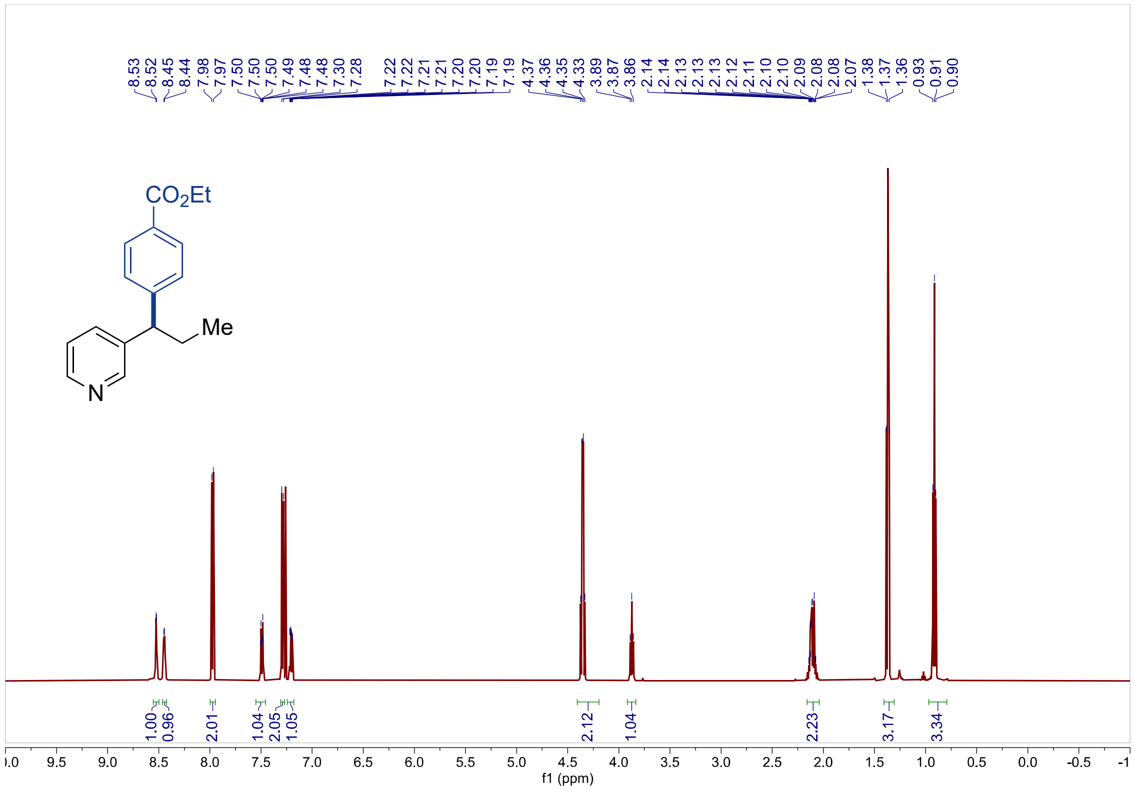

^{13}C NMR: (126 MHz, $CDCl_3$) of **27**

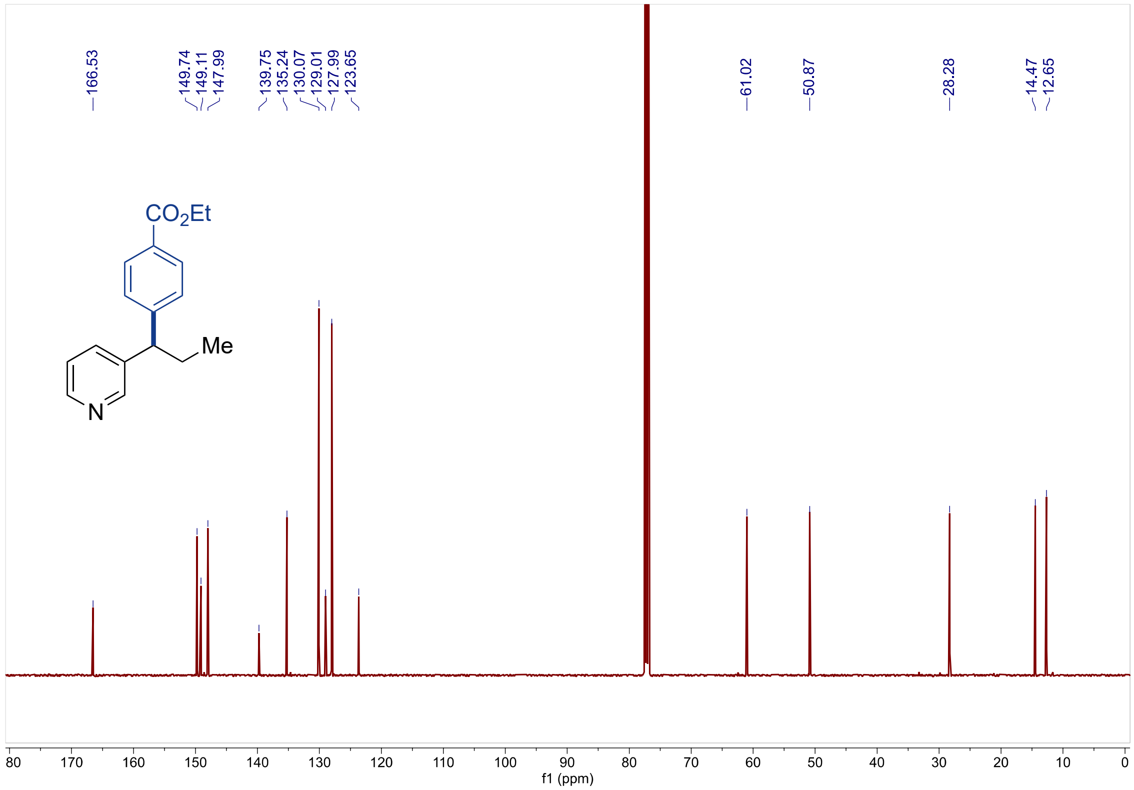

^{1}H NMR (500 MHz, $CDCl_3$) of **28**

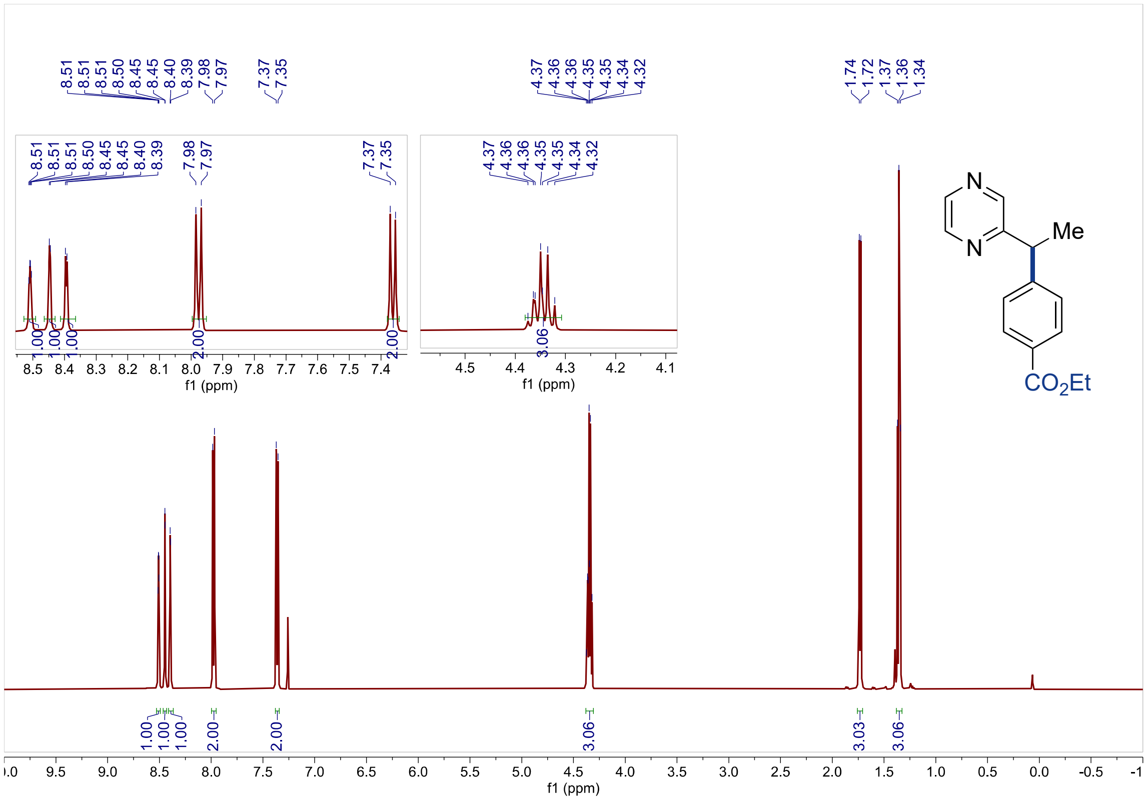

^{13}C NMR: (126 MHz, $CDCl_3$) of **28**

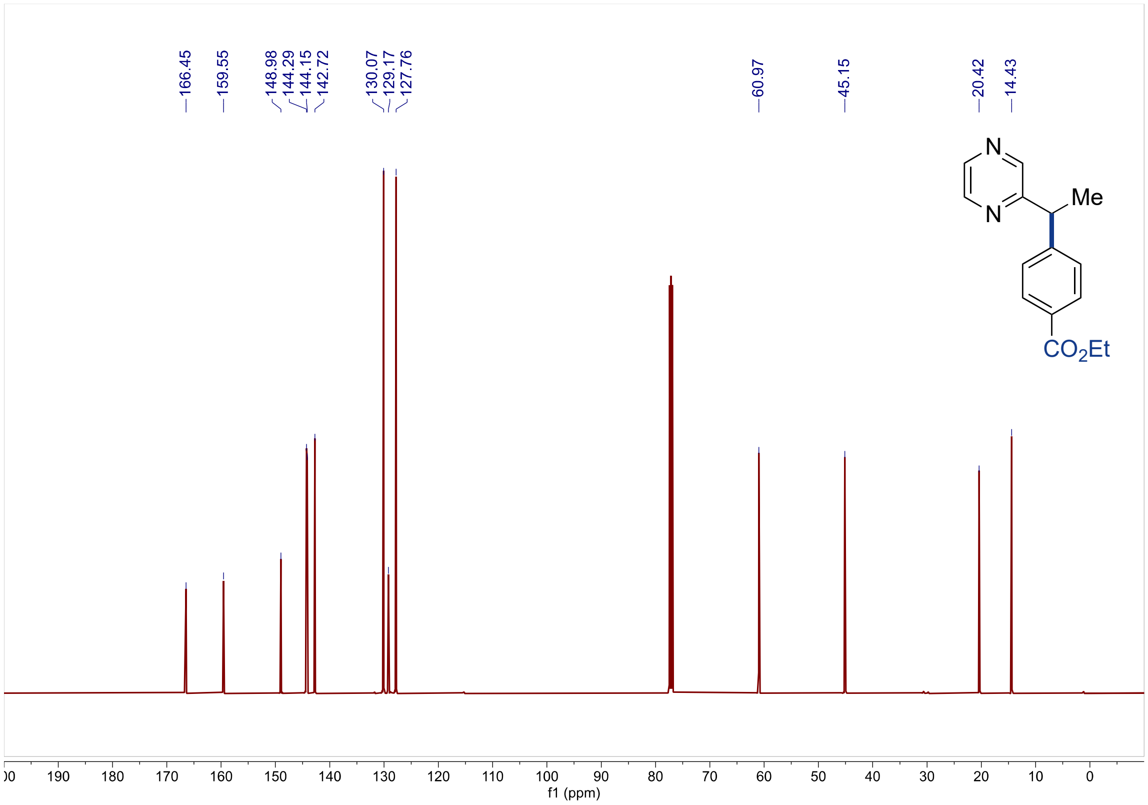

^{1}H NMR (500 MHz, $CDCl_3$) of **29**

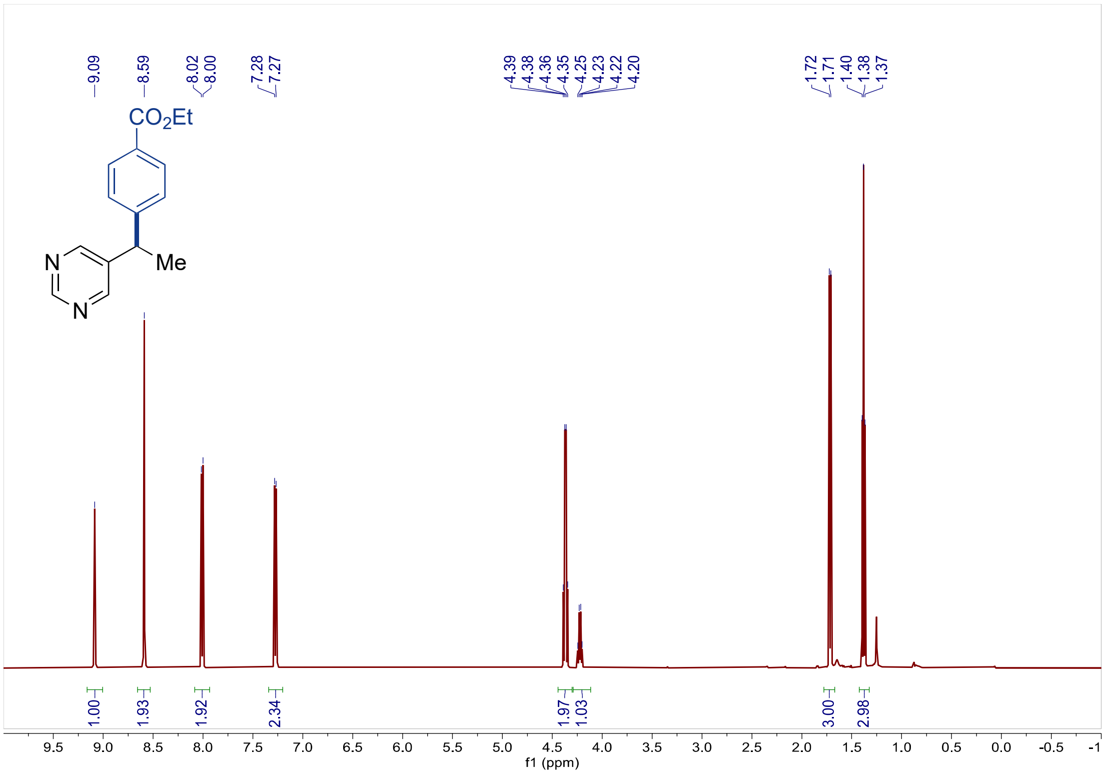

^{13}C NMR: (126 MHz, $CDCl_3$) of **29**

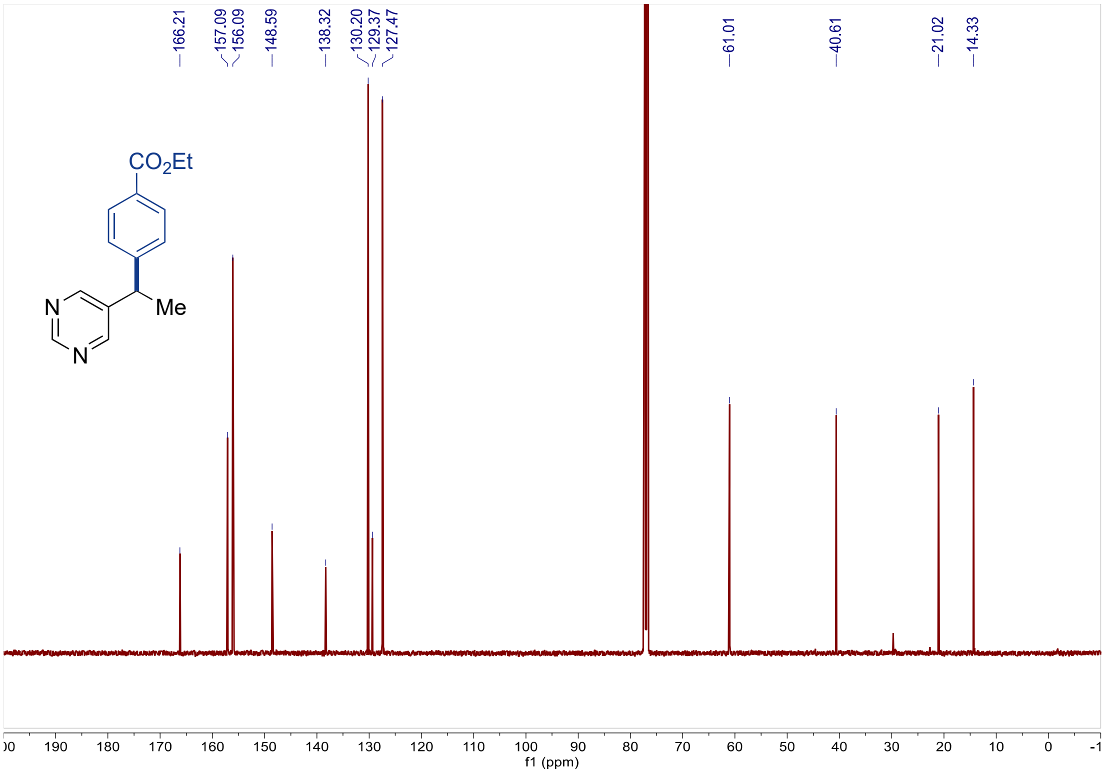

^{1}H NMR (500 MHz, $CDCl_3$) of **30**

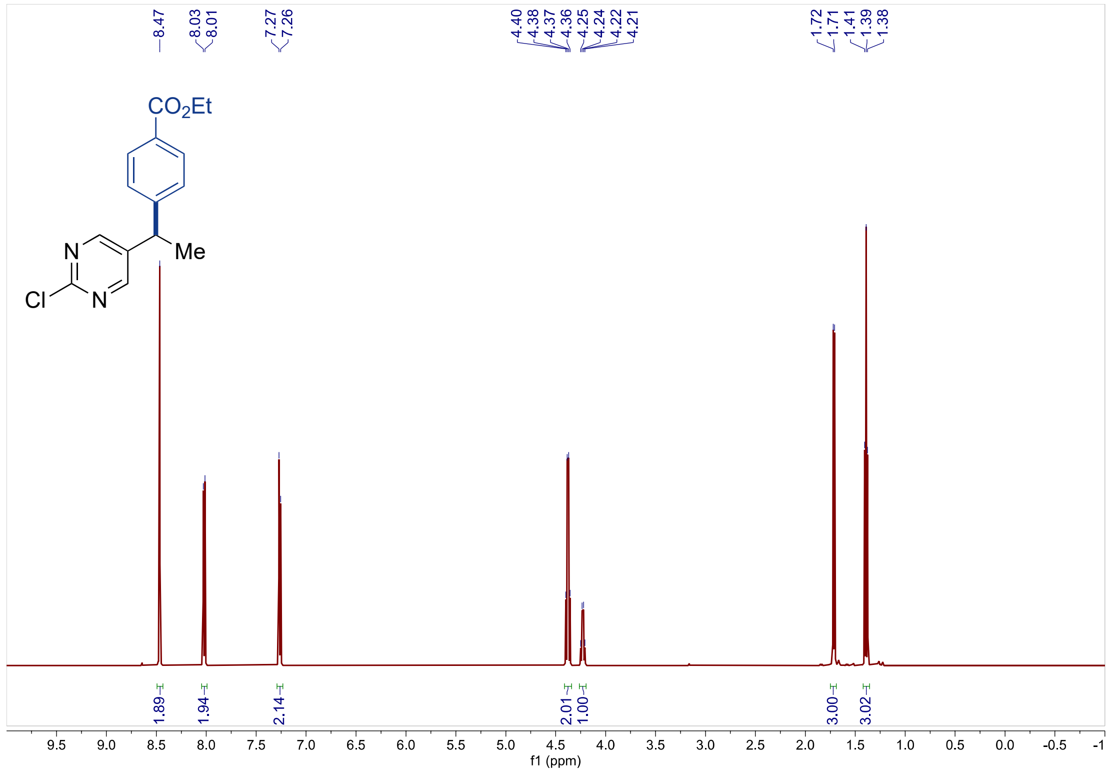

^{13}C NMR: (126 MHz, $CDCl_3$) of **30**

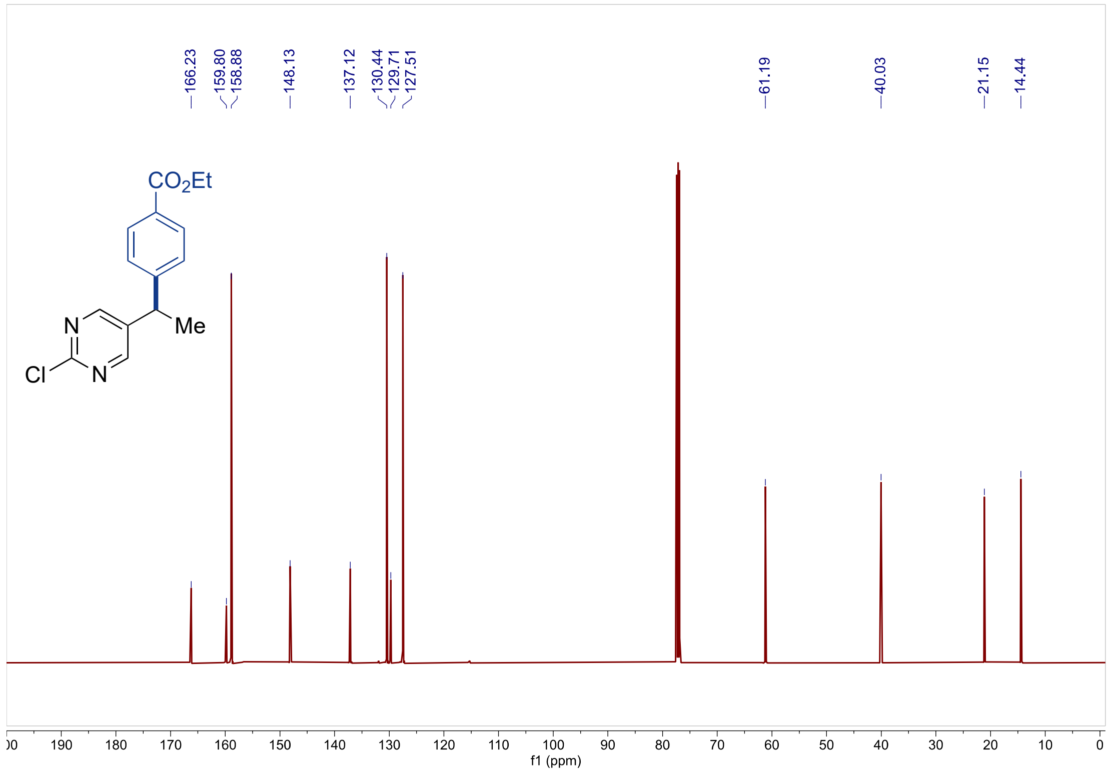

^{1}H NMR (500 MHz, $CDCl_3$) of **31**

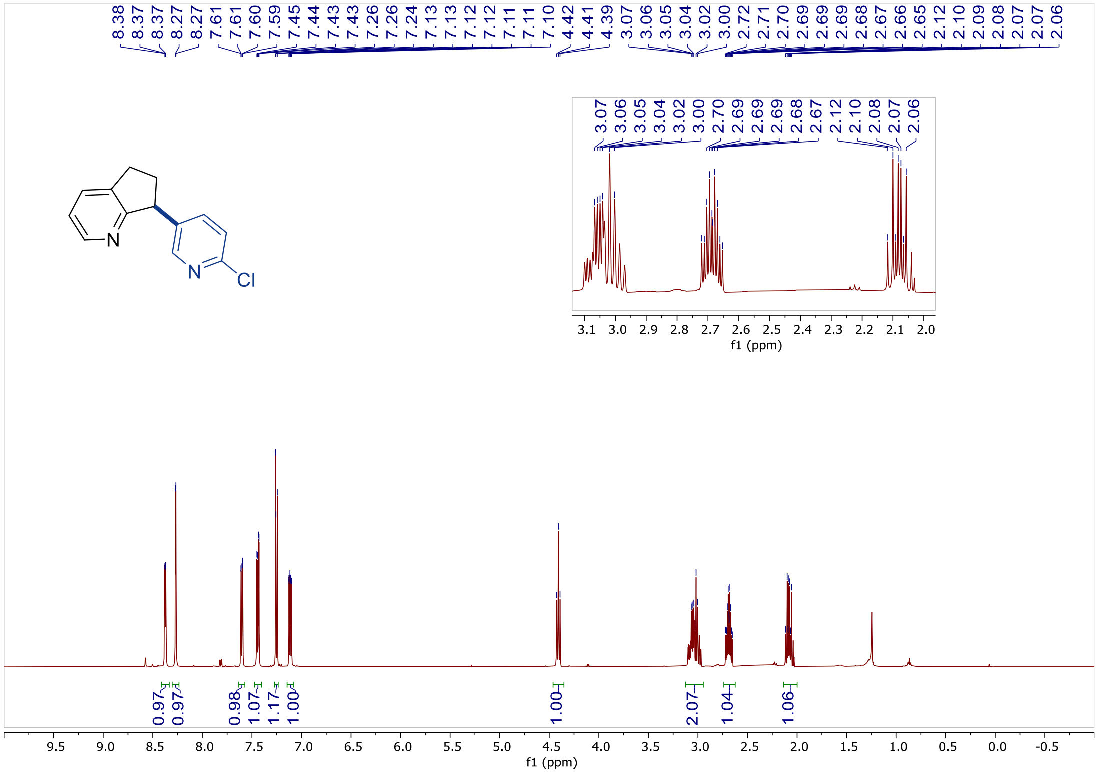

^{13}C NMR: (126 MHz, $CDCl_3$) of **31**

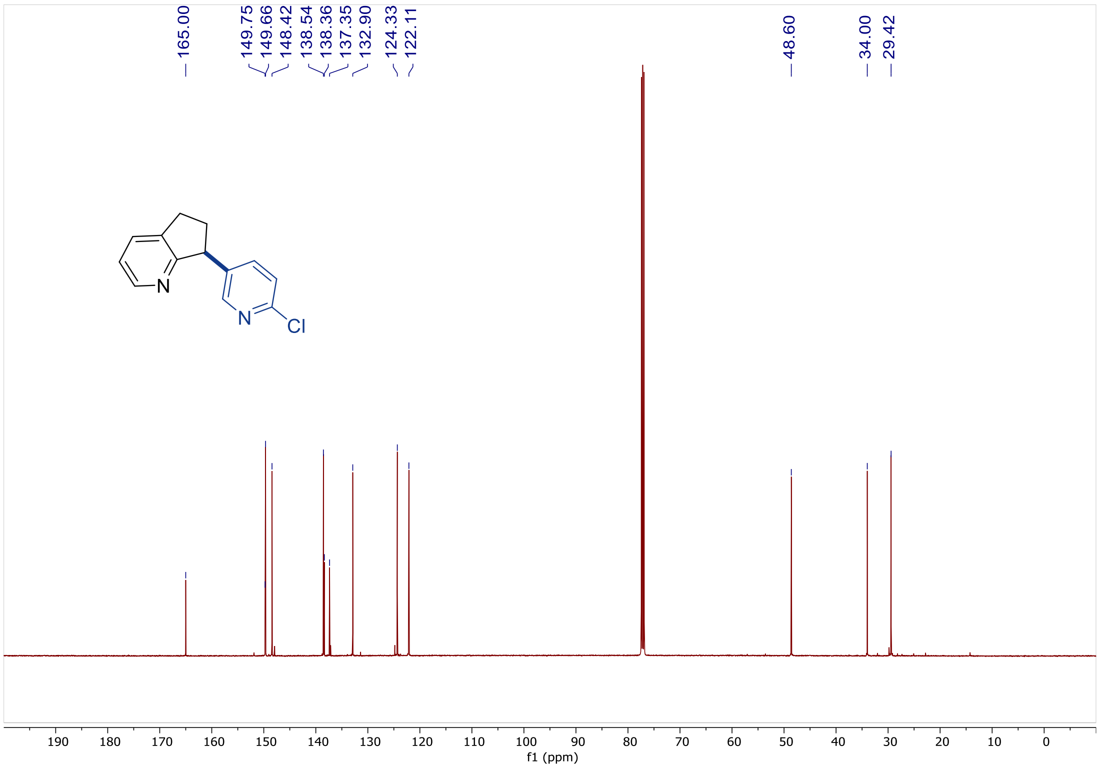

^{1}H NMR (500 MHz, $CDCl_3$) of **32**

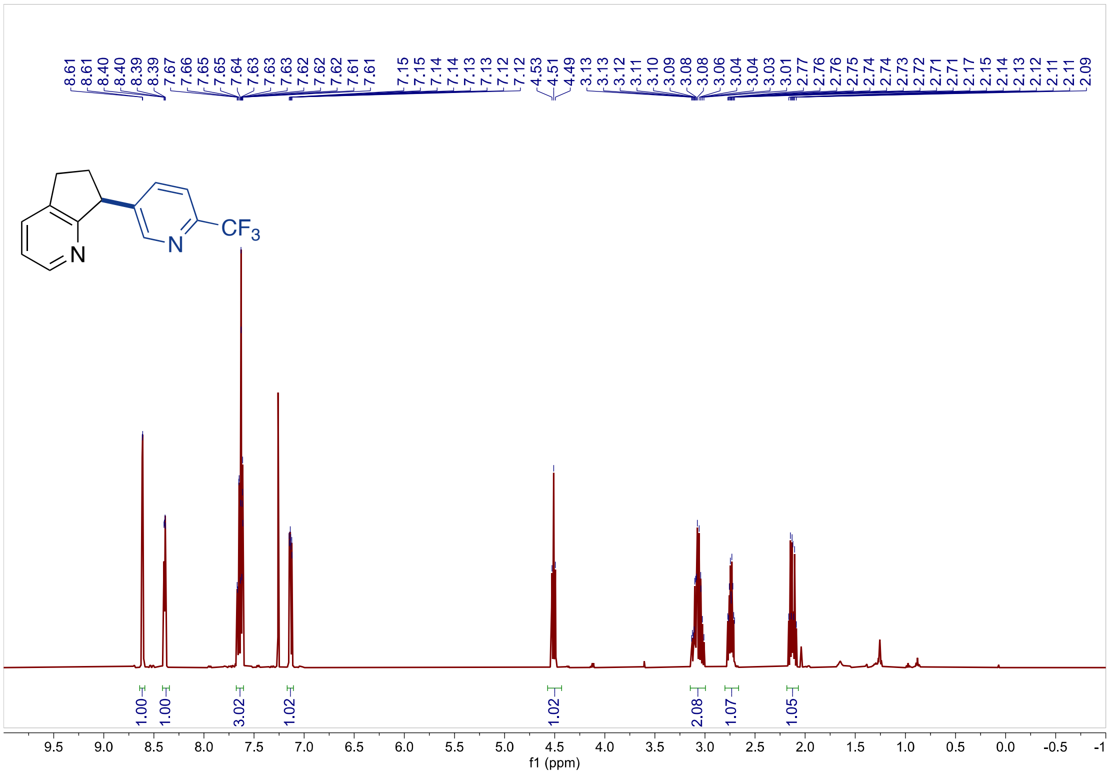

^{13}C NMR: (126 MHz, $CDCl_3$) of **32**

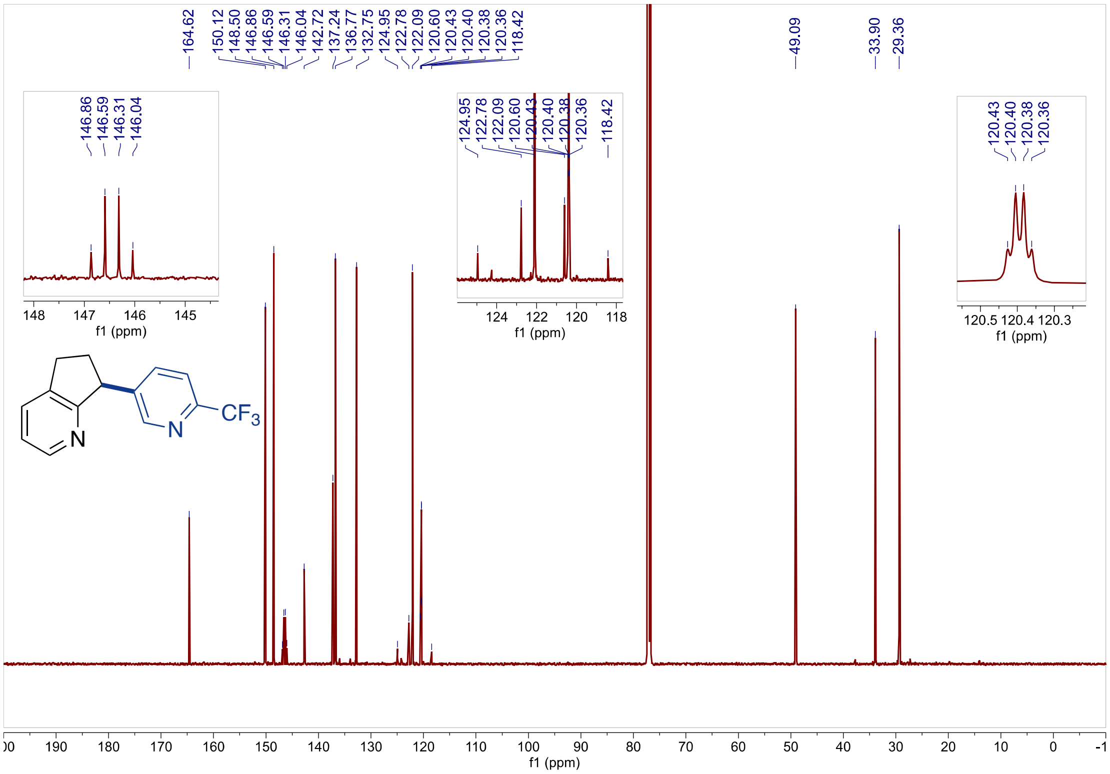

^{19}F NMR (377 MHz, $CDCl_3$) of **32**

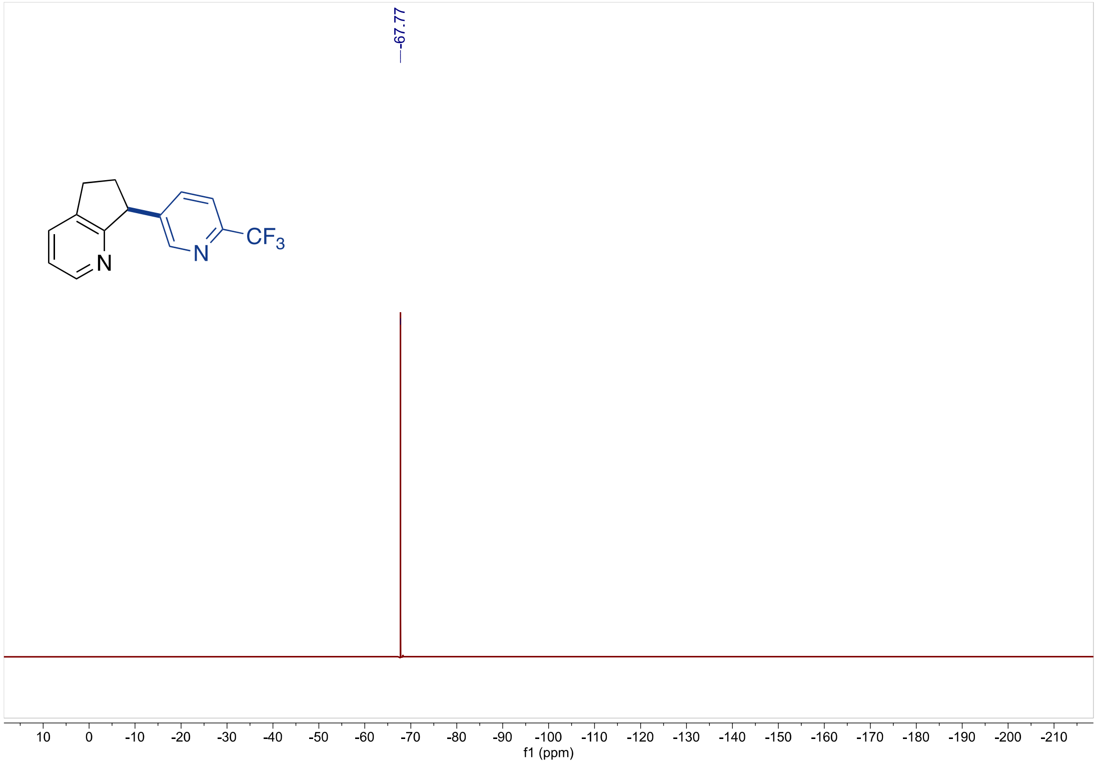

^{1}H NMR (500 MHz, $CDCl_3$) of **33**

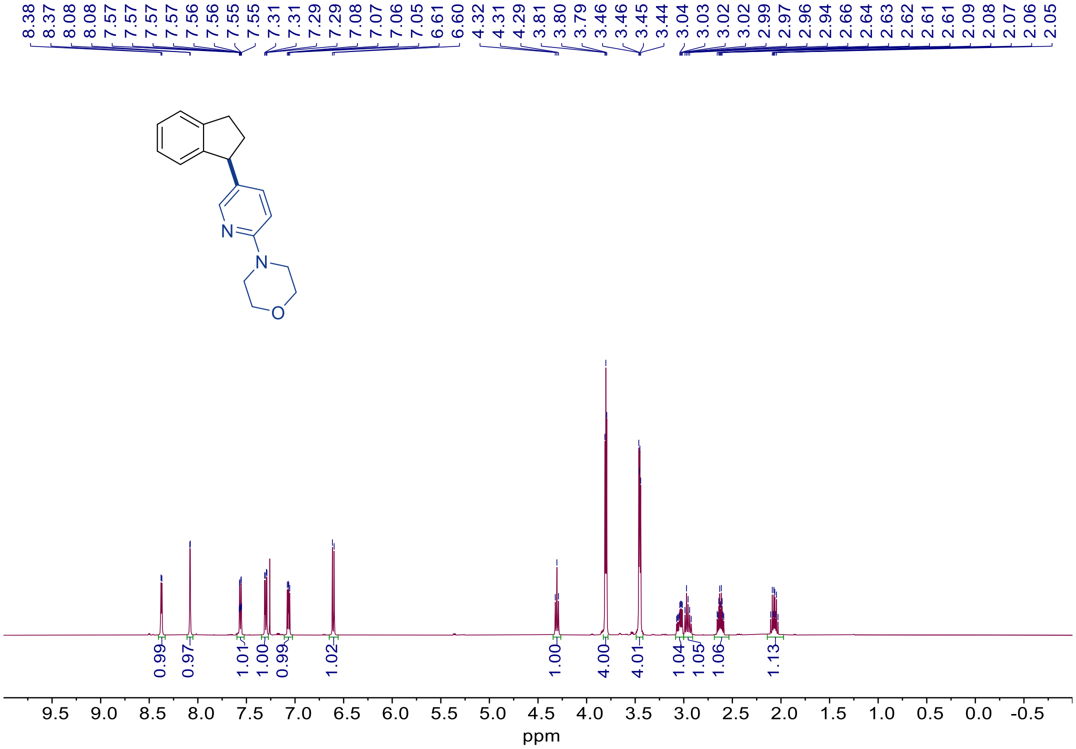

^{13}C NMR: (126 MHz, $CDCl_3$) of **33**

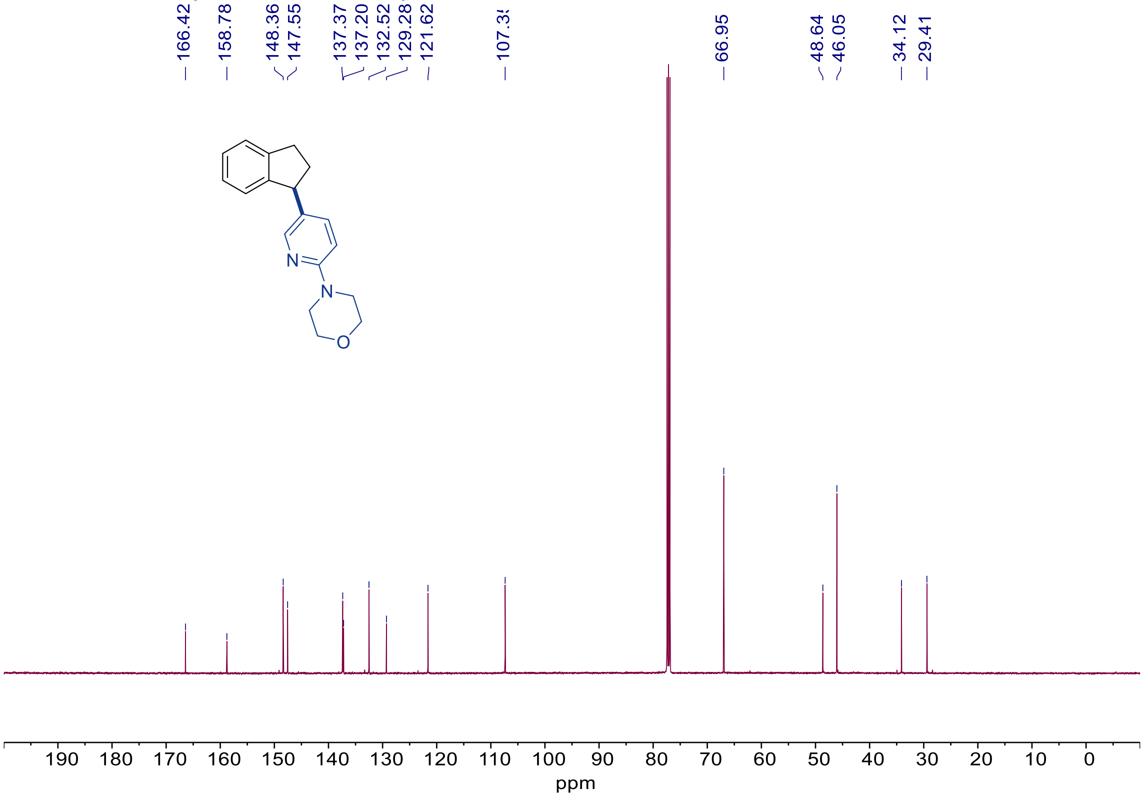

^{1}**H NMR** (500 MHz, $CDCl_3$) of **34**

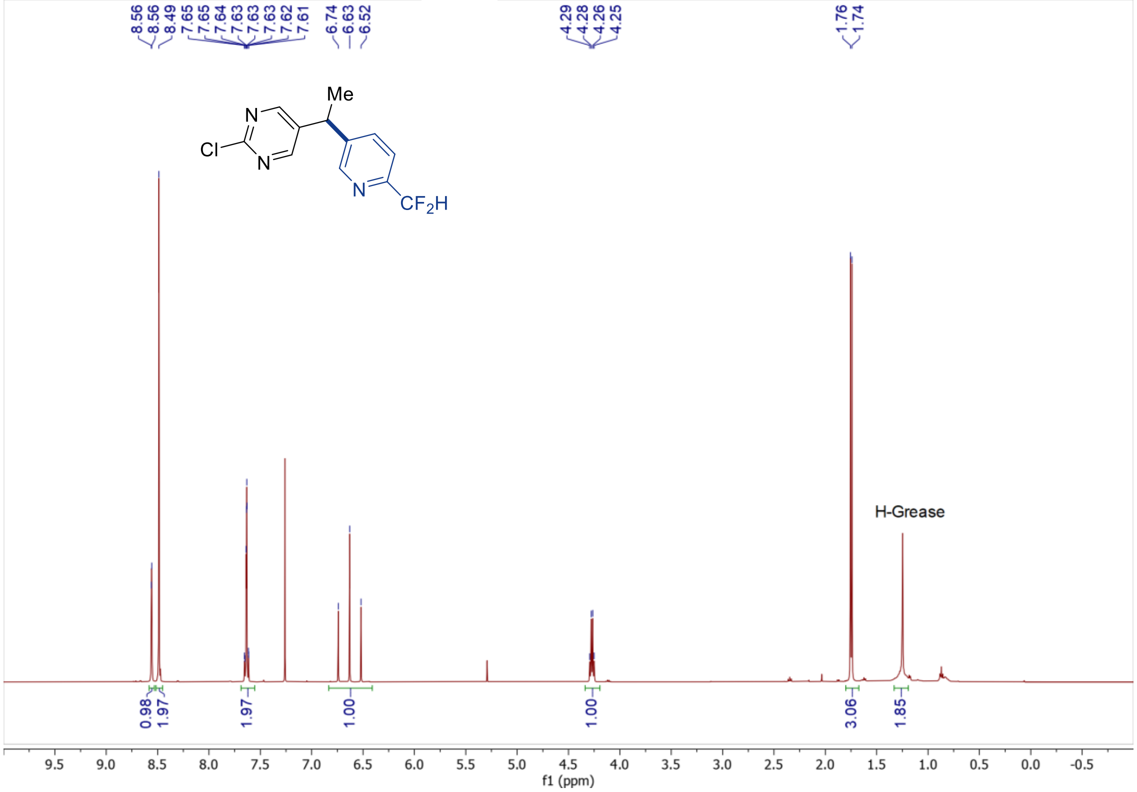

^{13}**C NMR**: (126 MHz, $CDCl_3$) of **34**

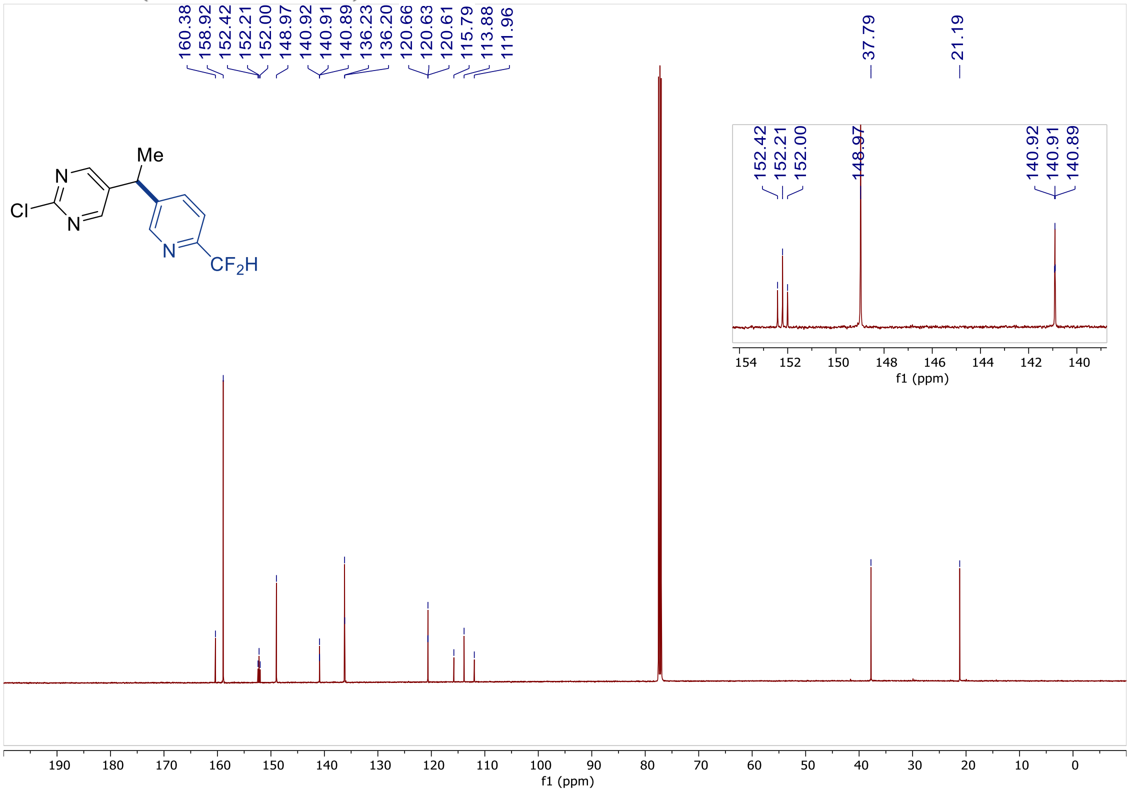

[19]**F NMR** (377 MHz, $CDCl_3$) of **34**

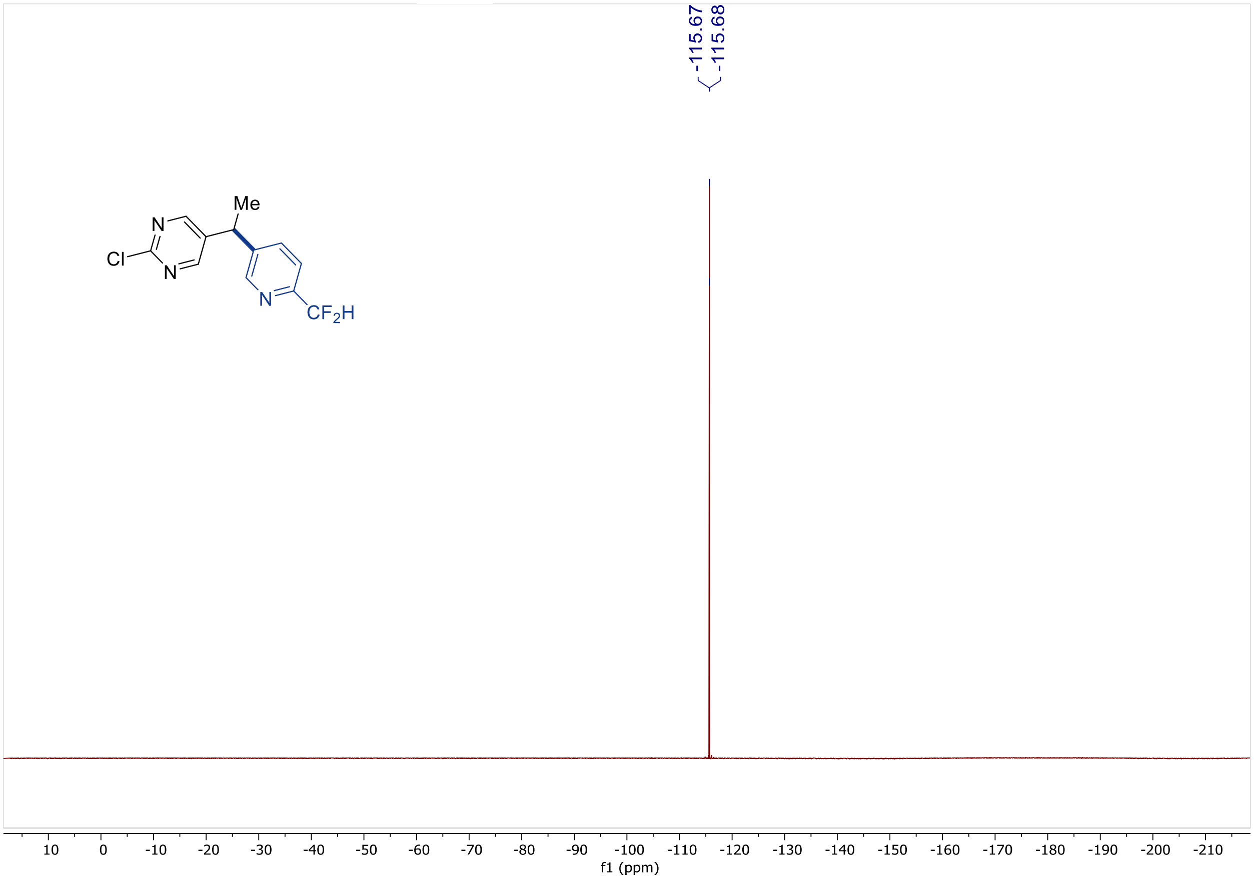

^{1}H NMR (500 MHz, $CDCl_3$) of **35**

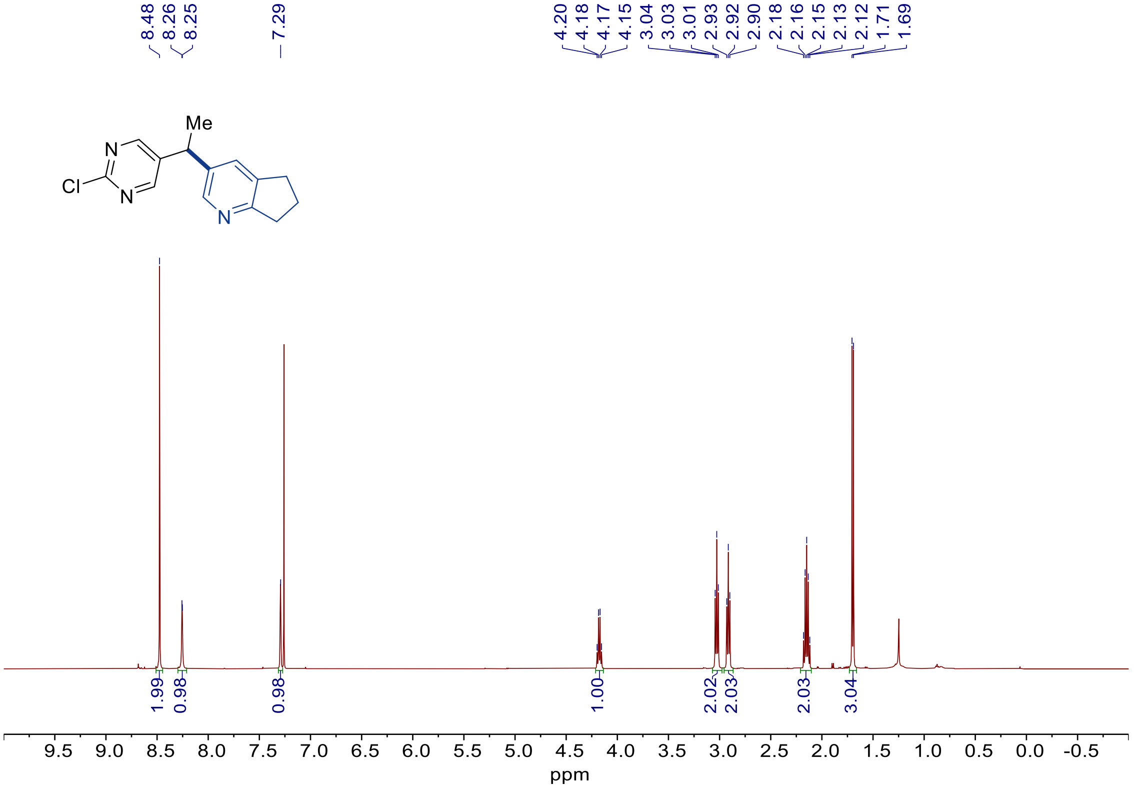

^{13}C NMR: (126 MHz, $CDCl_3$) of **35**

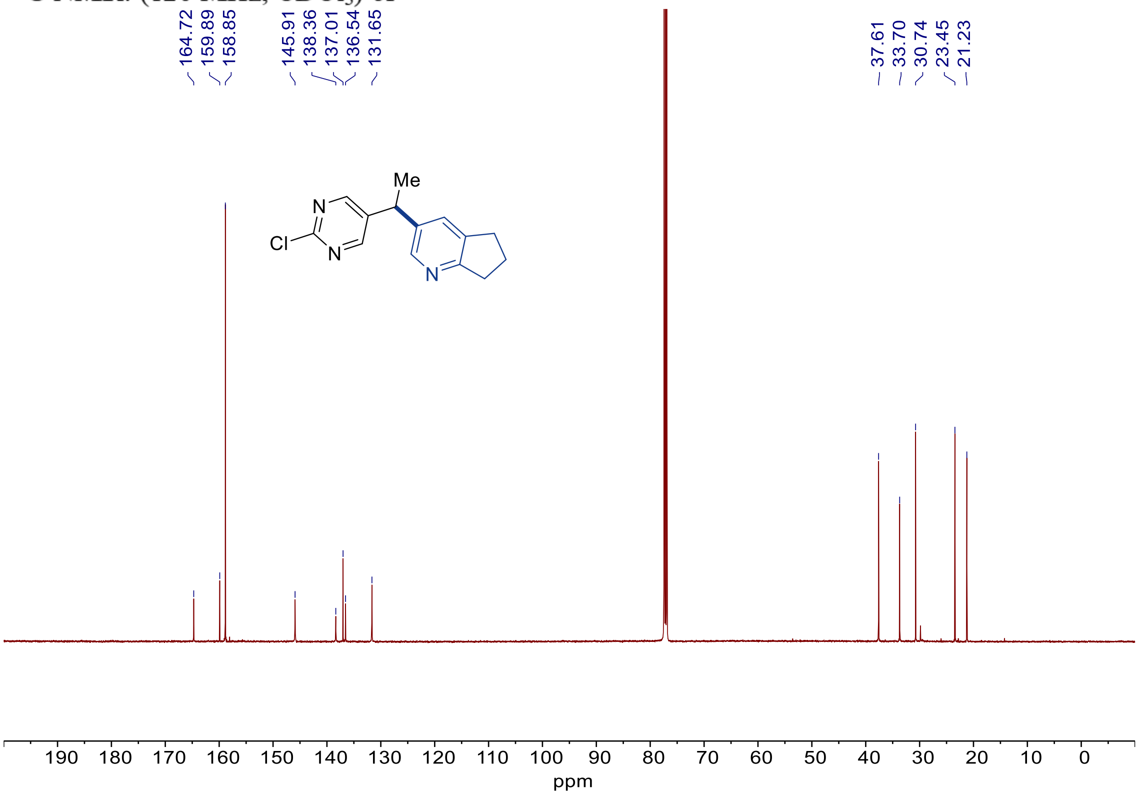

^{1}H NMR (500 MHz, $CDCl_3$) of **36**

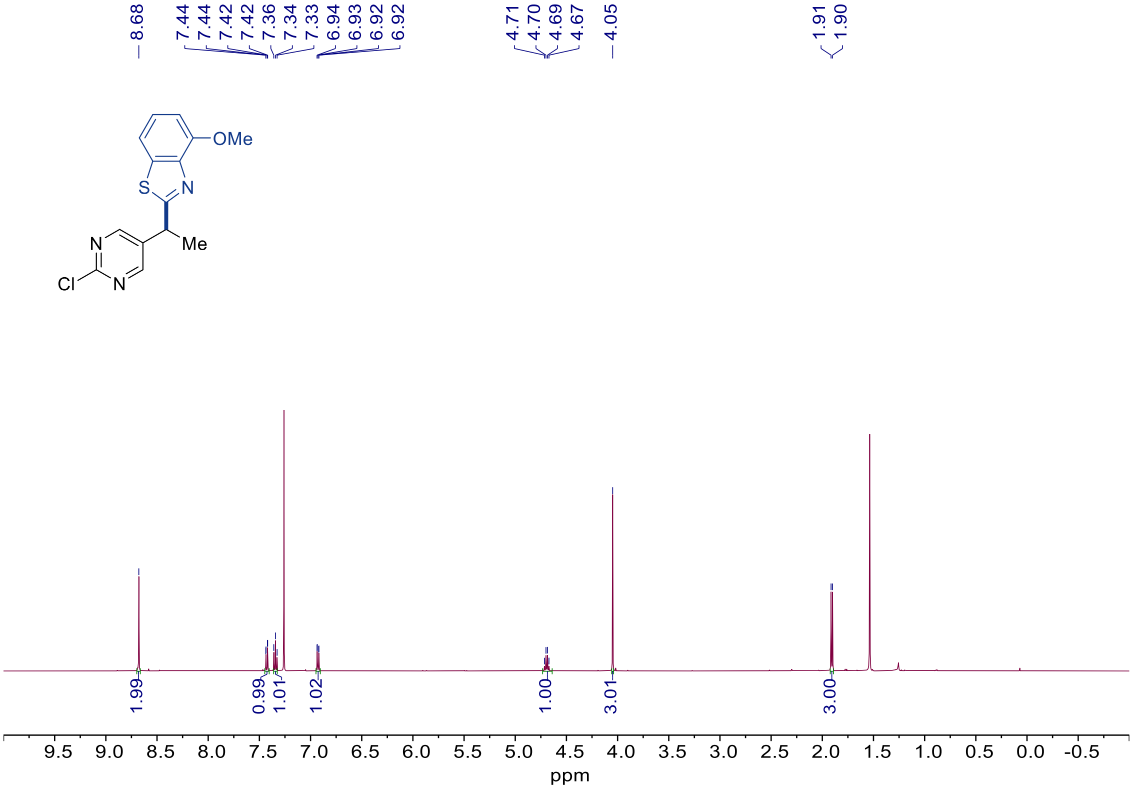

^{13}C NMR: (126 MHz, $CDCl_3$) of **36**

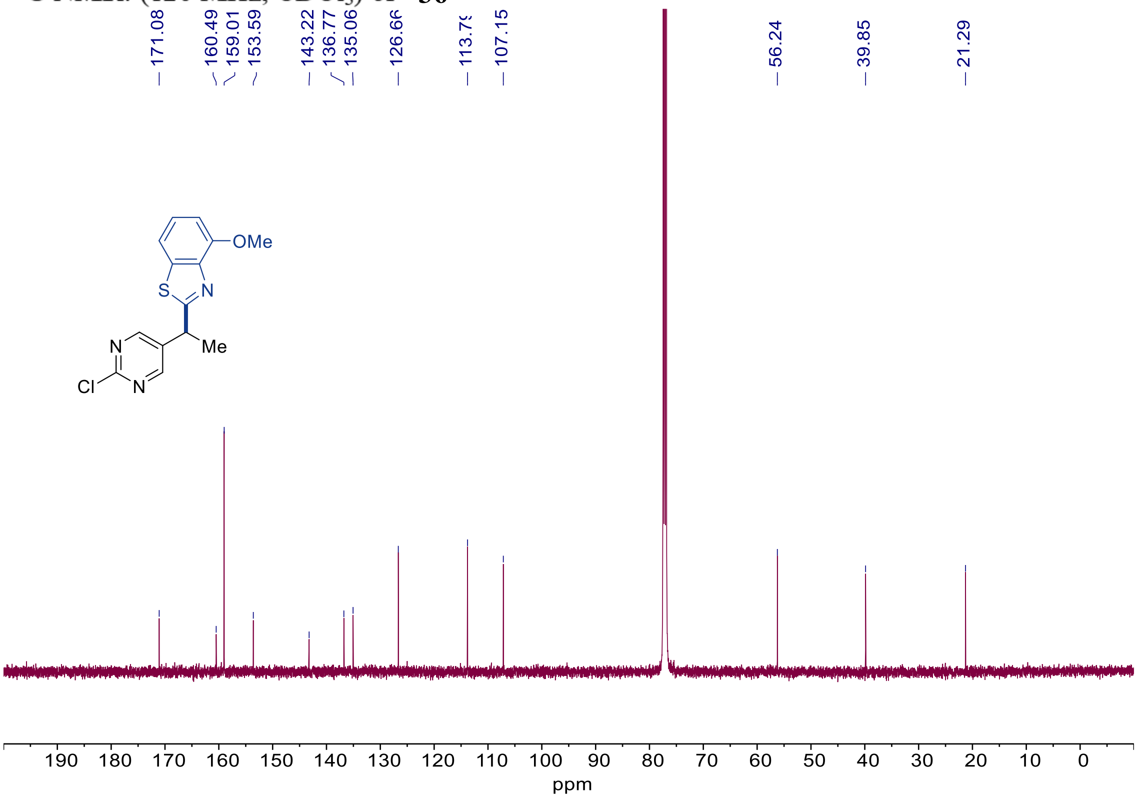

^{1}H NMR (500 MHz, $CDCl_3$) of **37**

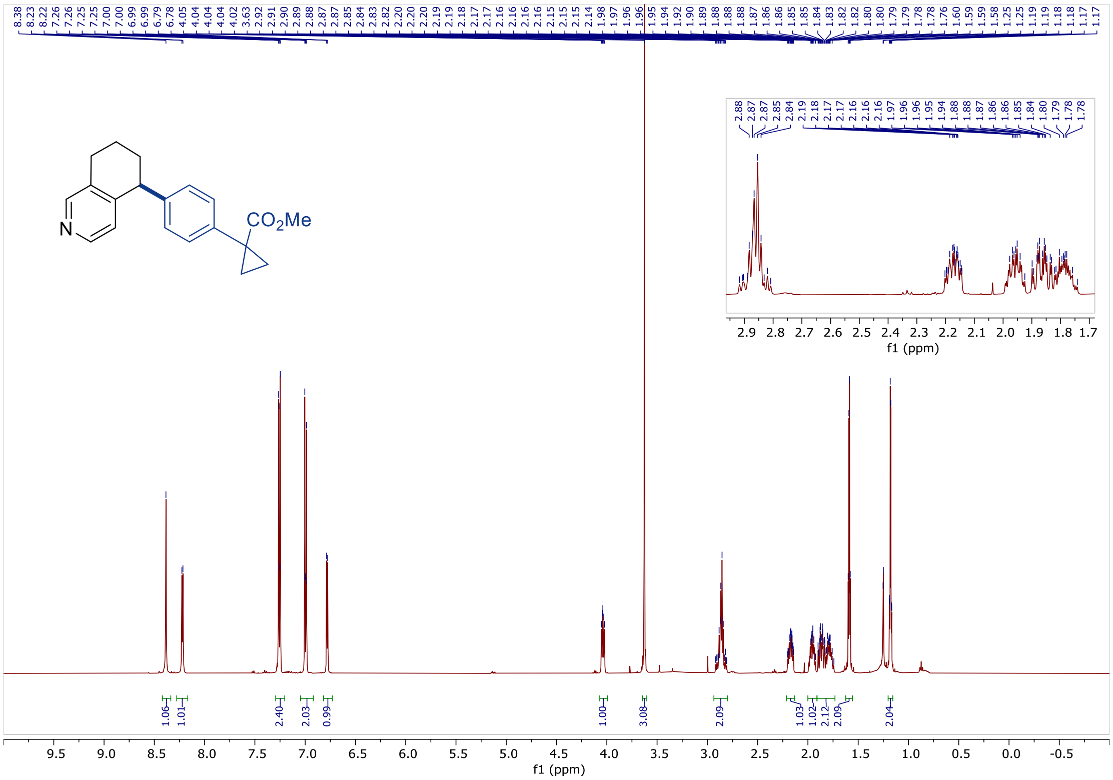

^{13}C NMR: (126 MHz, $CDCl_3$) of **37**

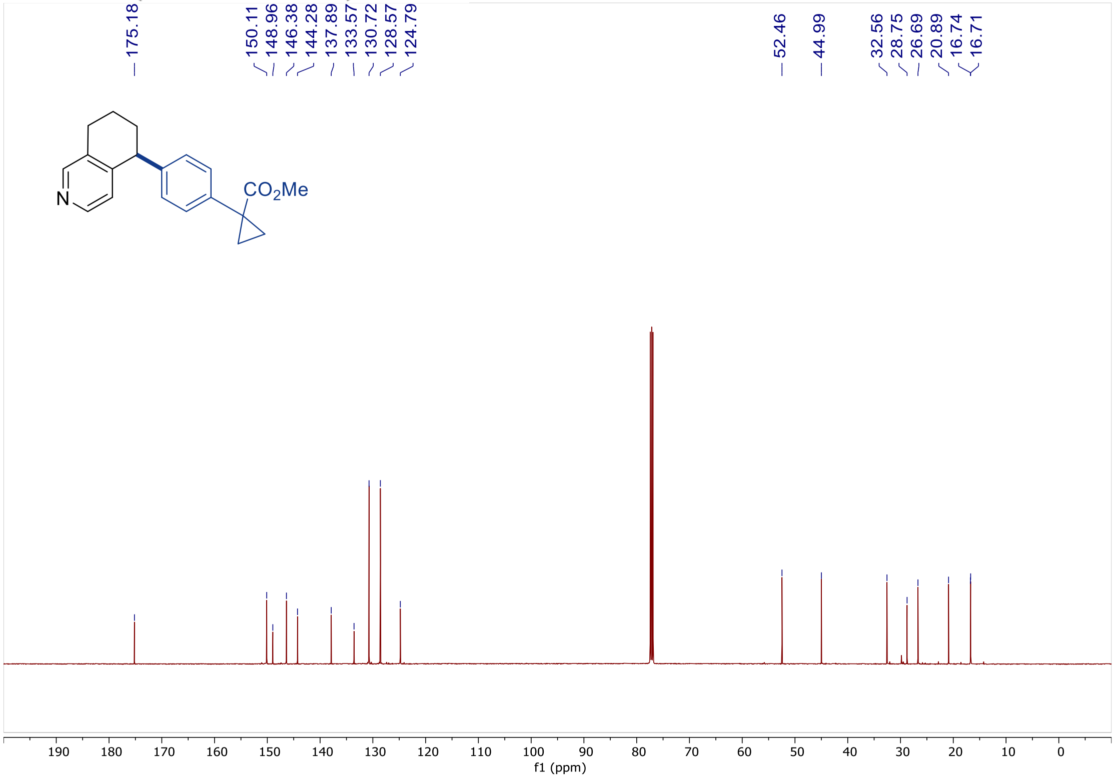

[1]**H NMR** (500 MHz, $CDCl_3$) of **38**

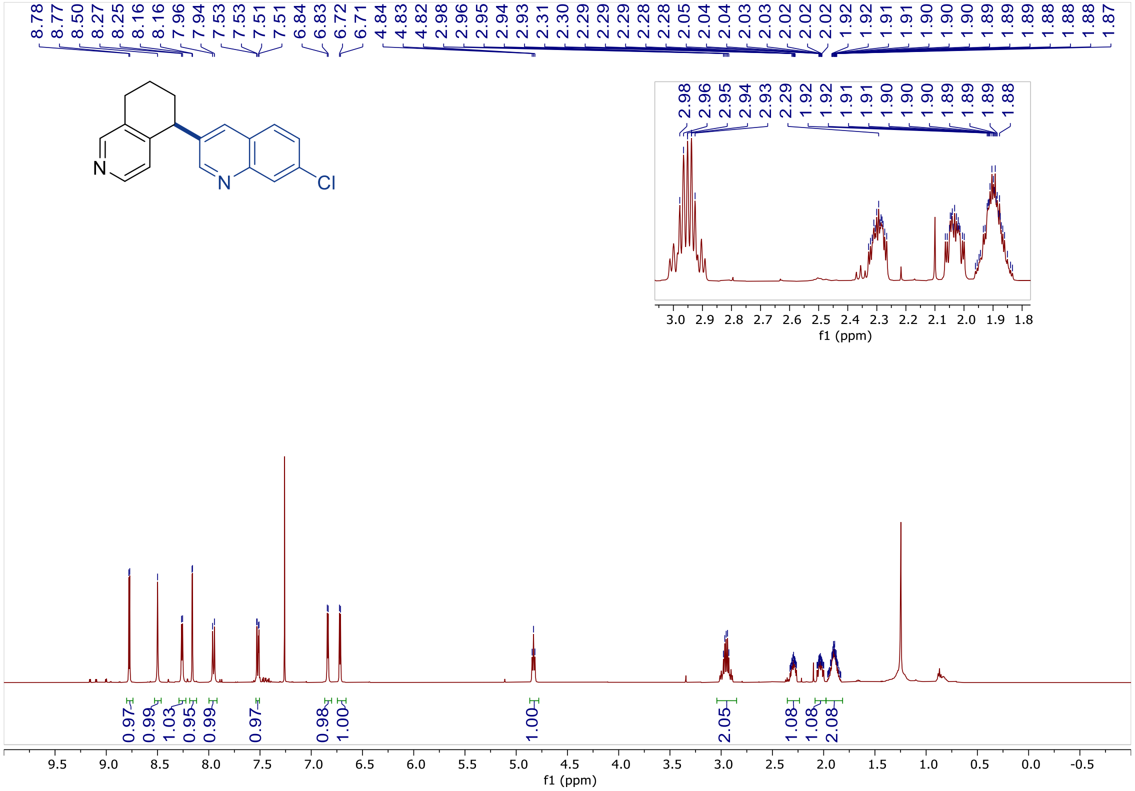

[13]**C NMR**: (126 MHz, $CDCl_3$) of **38**

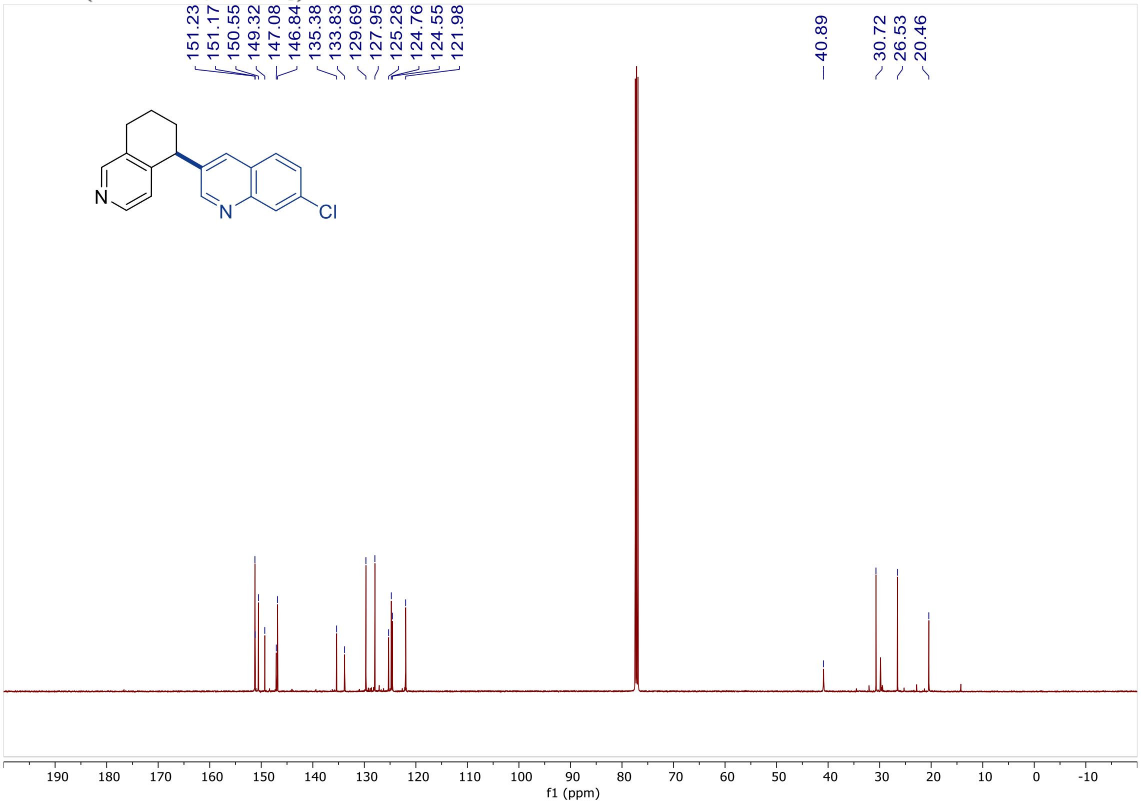

^{1}H NMR (500 MHz, $CDCl_3$) of **39**

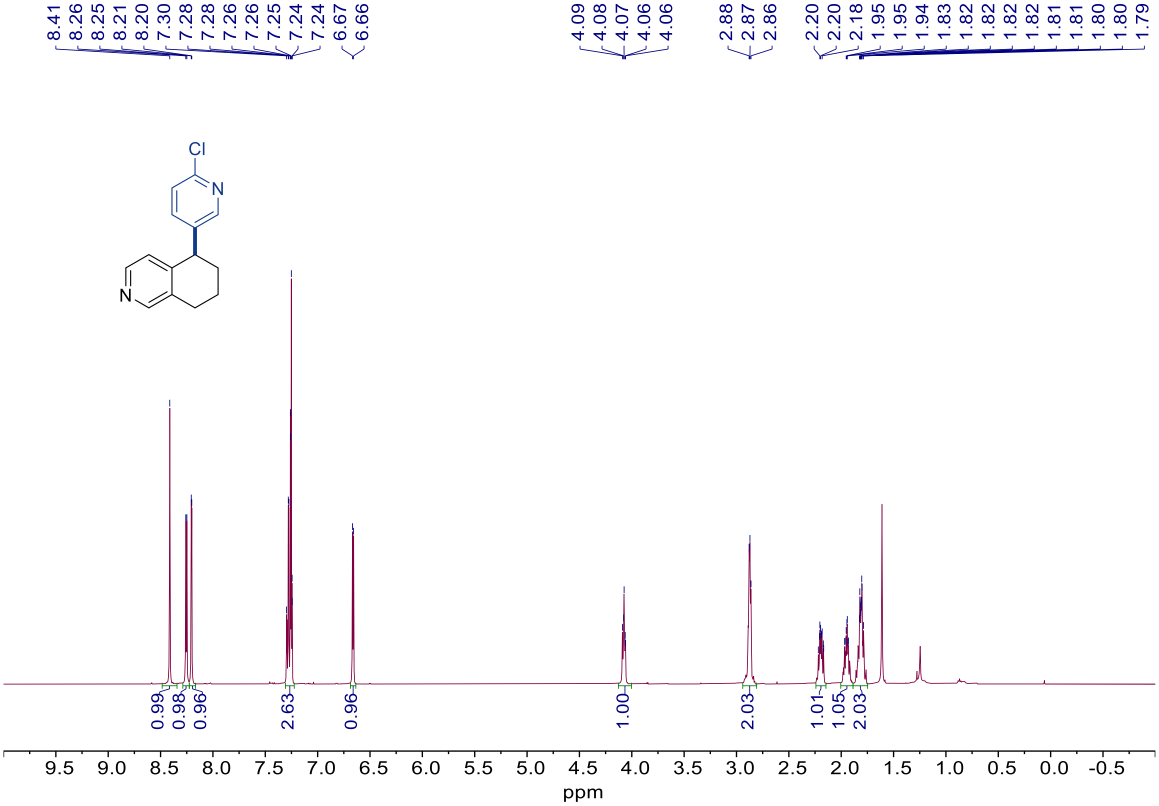

^{13}C NMR: (126 MHz, $CDCl_3$) of **39**

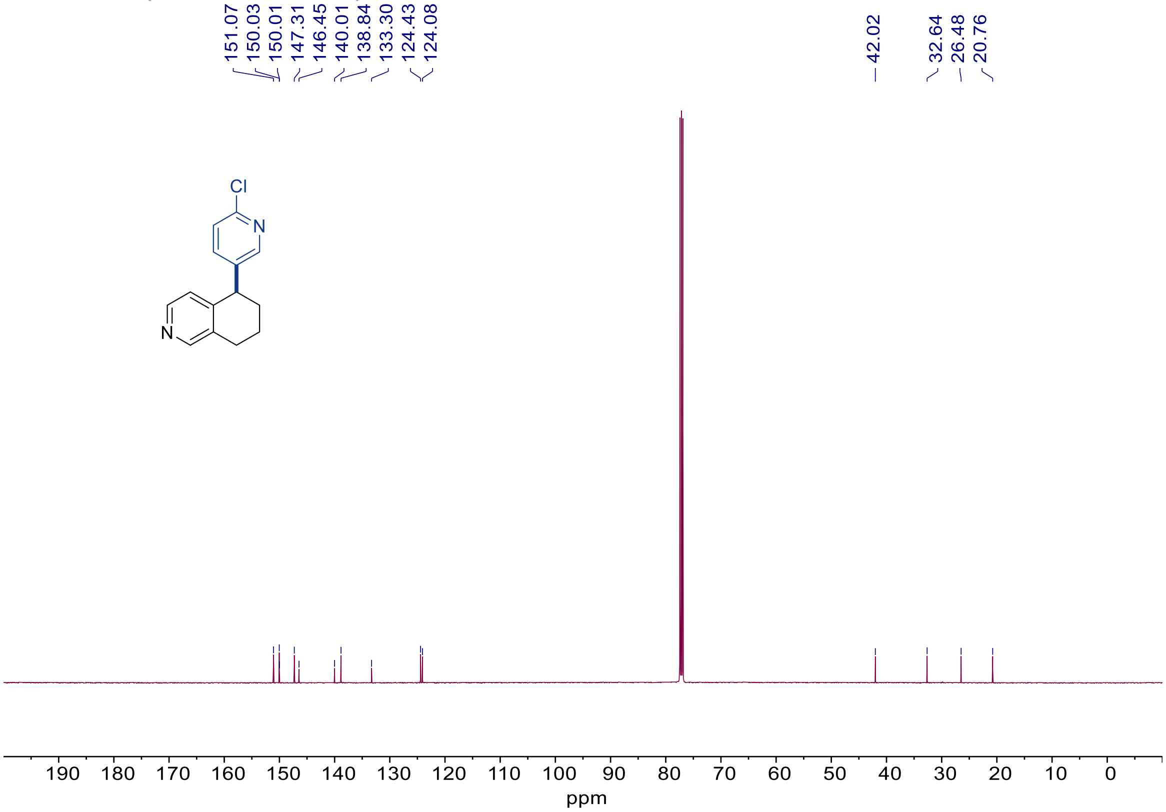

Supplement: Supplementary file 1 [file cs5c08221_si_001.pdf]
